# Diacarnusins A‑F, *nor*-Terpene Endoperoxides of the Red Sea Sponge *Diacarnus erythraeanus*

**DOI:** 10.1021/acs.jnatprod.6c00510

**Published:** 2026-07-17

**Authors:** Rania Hasib Afana, Lilach Raijman-Nagar, Micha Ilan, Shmuel Carmeli

**Affiliations:** † School of Chemistry, Raymond and Beverly Sackler Faculty of Exact Sciences, 26745 Tel-Aviv University, Ramat Aviv, Tel-Aviv 6997801, Israel; ‡ School of Zoology, George S. Wise Faculty of Life Sciences, 26745 Tel-Aviv University, Ramat Aviv, Tel-Aviv 6997801, Israel

## Abstract

Six new *nor-*terpene endoperoxides, diacarnusins A–F (**1**–**6**), along with five known ones (**7**–**11**), were isolated from the extract of the Red Sea sponge *Diacarnus erythraeanus*, which presented cytotoxic activity against the colon carcinoma cell line. The structure elucidation was performed by analysis of mass spectrometric and 1D and 2D NMR data. The relative and absolute configurations of the stereocenters of the compounds were deduced based on the Capon and Macleod empirical rule and comparison of the sign of their specific rotations with those of endoperoxides with proven absolute configurations. The cytotoxicity of the pure compounds was evaluated against a panel of cancer cell lines. The structure elucidation and biological activity of the compounds are discussed.

## Graphical abstract

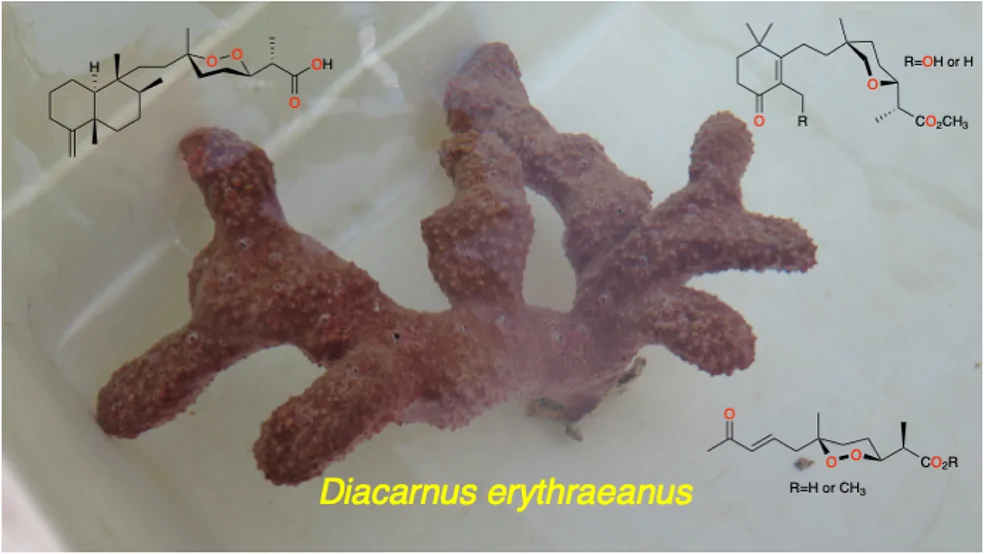

The first published *nor*-terpene endoperoxide, muqubilin, was isolated by Kashman and Rotem from a sample of the Red Sea sponge *Diacarnus erythraeanus* (Figure S1, identified at the time of publication as *Prianos* sp.).^1^ The identification of sigmospectrellin-A methyl ester from the Papua-New Guinean sponge *Sigmospectrella laevis* followed that of muqubilin.^2^ The absolute configuration of the stereocenters of sigmosceptrellins and muqubilin was established by Capon and Macleod.^3,4^ They proposed an empirical rule for determining the relative configuration of the endoperoxide end of this group of *nor*-terpenes, which is based on ^1^H and ^13^C NMR chemical shifts of selected positions of their methyl ester derivatives.^3,4^ The absolute configuration of the sigmosceptrellins was assigned by chemical transformations and comparing the sign of the products’ specific rotations.^3^ The absolute configuration of the stereocenters of muqubilin was based on a comparison of the sign of its specific rotation with those of endoperoxides with proven absolute configuration.^3^ The latter method was further used to establish the relative and absolute configuration of additional *nor*-terpene endoperoxides.^5^ Since 1979, more than 70 different *nor*-terpene endoperoxides have been isolated from sponges of the genera *Diacarnus*, *Sigmospecterella*, *Negombata*, *Latrunculia*, *Mycale*, *Hyrtios*, and their structures and biological activities against cancer cell lines, protozoans, fungi, and bacteria are discussed in a recent review.^6^ Samples of *D. erythraeanus* from various locations along the coasts of the Red Sea were collected, and their extracts yielded 15 different *nor*-terpene endoperoxides (see Figure S2 in the Supporting Information).^1,7–13^ A survey aimed at isolating cytotoxic secondary metabolites from samples of sponges collected along the Israeli coasts of the Red and Mediterranean Seas led to the discovery of a potent cytotoxic extract, active against HCT-116 colon carcinoma, from the Red Sea sponge *D. erythraeanus*, which was collected in July 2019 from the coast of Eilat, Gulf of Aqaba, Israel. Cytotoxicity-guided separation of the crude extract by reversed-phase and size-exclusion chromatography yielded six new *nor-*terpene endoperoxides, diacarnusins A–F (**1**–**6**), along with five known ones, sigmosceptrellin B (**7**) and its methyl ester (**8**),^14^ diacarperoxide A (**9**),^15^ methyl diacarnoate A (**10**),^16^ and (−)-*ent*-muqubilone (**11**).^17^ Two of the isolated metabolites, diacarnusins C (**3**) and D (**4**), were converted spontaneously, each into two isomeric products that were further transformed to complex mixtures of products. The later spontaneous transformation of **3** and **4** may enlighten the mode of action of *nor*-terpene endoperoxides. The cytotoxicity of those metabolites that were stable enough (**1**, **2**, and **5–11**) was examined against a panel of cancer cell lines. The structure elucidation of the six new secondary metabolites (**1**–**6**) and the four products of **3** and **4** are discussed below.

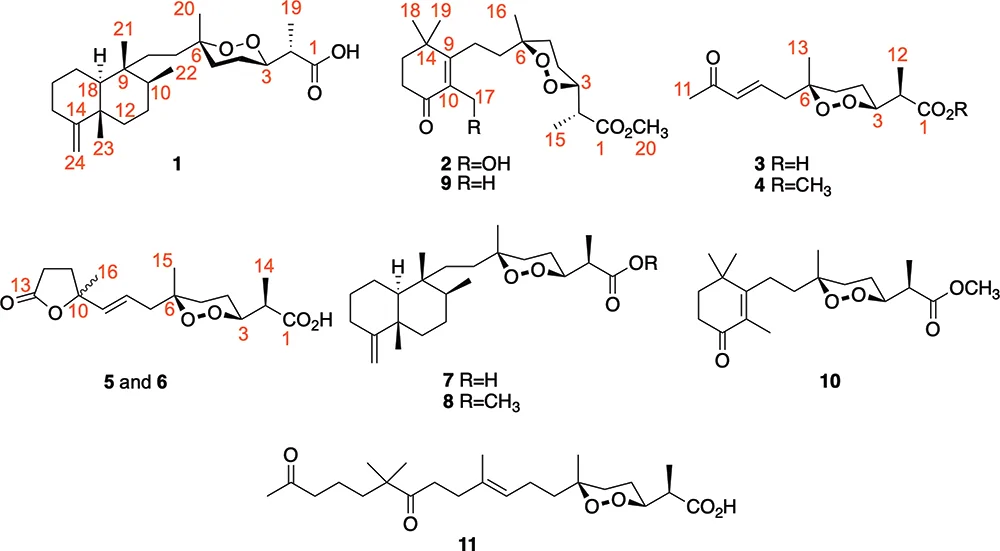



## Results and Discussion

Diacarnusin A (**1**) was isolated as an oily, transparent material. Its HRESIMS molecular cluster ion was identical with that of **7**, *m*/*z* 415.2817 [M + Na]^+^, fitting a molecular formula of C_24_H_40_O_4_ and corresponding to five degrees of unsaturation. Its ^1^H and ^13^C NMR data, in CDCl_3_ (see Tables 1 and S1), were comparable with those of **7**, presenting only small differences in the chemical shifts of the carbons and protons (see Table S2). Full analysis of the NMR data (Table S1 and Figure 1) of **1** revealed an identical planar structure to that of **7**. According to the empirical rule of Capon and Macleod,^3,4^ the chemical shifts of the doublet CH_3_-19 (δ_H_ 1.27, δ_C_ 13.2), in **1**, assign a *syn* (*threo*) relative configuration for C-2/C-3, while the singlet CH_3_-20 carbon chemical shift (δ_C_ 20.7, δ_H_ 1.25) suggests an axial orientation of the methyl attached to C-6, as in **7**. However, the positive specific rotation (α_D_ +17.5, in MeOH) measured for **1** was opposite to that of **7**, which presented a negative specific rotation (α_D_ −48.4, in MeOH). The latter comparison suggests that the cyclicperoxide-α-substituted propanoic acid moiety, which contributes the most to the specific rotation of sigmosceptrellin B (**7**) and diacarnusin A (**1**), is enantiomeric, although **1** and **7** are diastereomers due to their identical decalin units (they originate from the same sesterterpene precursor). The absolute configuration of **7** was determined by Capon and Macleod to be 2*R*,3*S*,6*R*.^3^ Based on these findings, we proposed the 2*S*,3*R*,6*S* absolute configuration of the endoperoxide moiety in diacarnusin A (**1**).

**Table 1:** NMR Data of Diacarnusin A (**1**) in CDCl_3_ ^a^

**Position**	***δ***_**C**_, **type** ^b^	***δ***_**H**_, **multiplicity,** *J* **in Hz**	**Position**	***δ***_**C**_, **type** ^b^	***δ***_**H**_, **multiplicity,** *J* **in Hz**
1	179.1, C		13	40.0, C	
2	42.8, CH	2.66, dq (6.7, 6.8)	14	160.6, C	
3	80.9, CH	4.14, brdt (6.4, 6.7)	15	33.0, CH_2_	2.27, ddd (4.3, 12.8, 13.7)
					2.09, brd (12.8)
4	23.4, CH_2_	1.74, m	16	28.6, CH_2_	1.86, brd (10.5)
					1.24, m
5	31.9, CH_2_	1.62, m	17	21.7, CH_2_	1.47, m
					1.42, m
6	80.5, C		18	48.4, CH	1.03, m
7	32.5, CH_2_	1.34, m	19	13.2, CH_3_	1.27, d (6.8)
		1.14, m			
8	30.7, CH_2_	1.30, m	20	20.7, CH_3_	1.25, s
		1.16, m			
9	38.9, C		21	18.3, CH_3_	0.73, s
10	36.4, CH	1.39, m	22	15.9, CH_3_	0.78, d (6.5)
11	27.4, CH_2_	1.48, m	23	20.8, CH_3_	1.03, s
		1.43, m			
12	37.3, CH_2_	1.58, m	24	102.5, CH_2_	4.49, s
		1.49, m			

a 500 MHz for ^1^H and 125 MHz for ^13^C.

b From HSQC.

**Figure 1:**
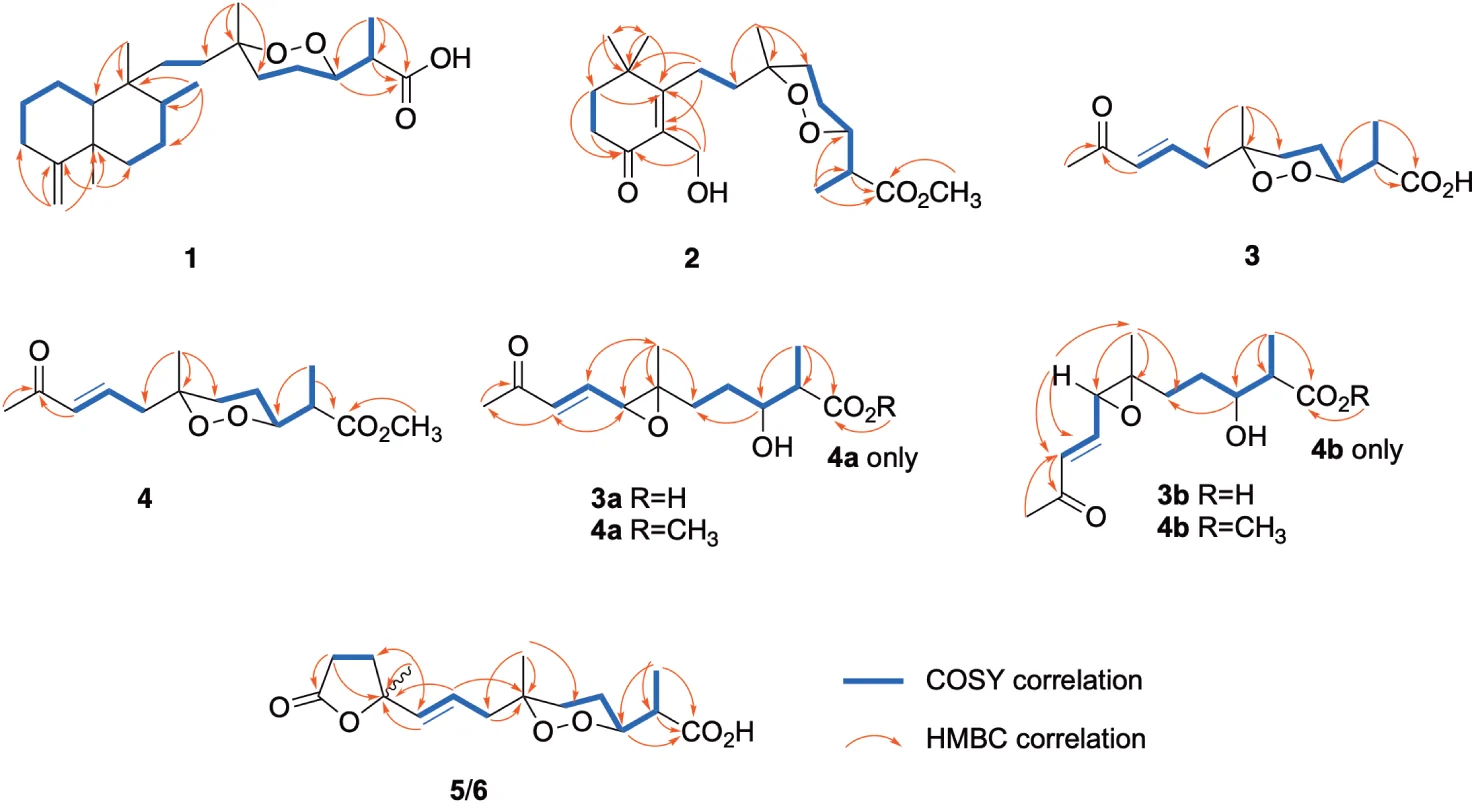
Selected COSY and HMBC correlations of compounds **1**–**6**.

Diacarnusin B (**2**) was isolated as a colorless oil. Its HRESIMS spectrum exhibited a molecular cluster ion peak at *m*/*z* 391.2093, [M + Na]^+^, corresponding to a molecular formula of C_20_H_32_O_6_ and five degrees of unsaturation. The typical ^1^H NMR and ^13^C NMR (Table 2) signals (δ_C_ 81.3, δ_H_ 4.07, CH-3, and δ_C_ 79.6, C-6) confirmed the presence of a cyclic endoperoxide common to this family of natural products. The ^1^H and ^13^C NMR data of **2**, in DMSO-*d*_6_, were comparable with those of diacarperoxide A (**9**) except for one singlet vinyl methyl that was substituted in **2** by a vinylic methylene (see Table S4). The structure of **2** was fully assigned based on analysis of the NMR data in DMSO-*d*_6_ (Table S3, Figure 1). The COSY correlations allowed to suggest three isolated spin systems: H_3_-15-H-2-H-3-H_2_-4-H_2_-5, H_2_-7-H_2_-8, and H_2_-12-H_2_-13 (Table S3 and Figure 1). HMBC correlations (Table S3 and Figure 1) enabled us to assemble the entire planar structure of **2**, which was named diacarnusin B. The relative stereochemistry of the peroxide ring was determined by applying the Capon and MacLeod empirical rule for similar terpene peroxides.^3,4^ The comparison of the ^1^H NMR and ^13^C NMR chemical shifts of **2** with those of the known diacarperoxide A (**9**) in DMSO-*d*_6_ (Table S2) revealed their identical relative configuration. The chemical shifts of the proton and carbon resonances of CH_3_-15 (δ_H_ 1.04 and δ_C_ 12.9, respectively) in **2** are comparable with those of **9** (δ_H_ 1.08 and δ_C_ 12.8, respectively) and thus indicate the *anti* (*erythro*) relative configuration that suggests either 2*R*,3*R* or 2*S*,3*S* absolute configuration. The equatorial orientation of CH_3_-16 in **2** was deduced by comparison of its ^13^C NMR chemical shift (δ_C_ 23.4) with that of **9** (δ_C_ 23.0). The measured specific rotation of **2** was +18.5, while that of **9** was +43.8. Since the absolute configuration of **9** was previously determined to be 2*R*,3*R*,6*R*, the absolute configuration of diacarnusin B (**2**) should be 2*R*,3*R*,6*R* as well.

**Table 2:** NMR Data of Diacarnusin B (**2**) in DMSO-*d*_6_ ^a^

**Position**	***δ***_**C**_, **type** ^b^	***δ***_**H**_, **multiplicity,** *J* **in Hz**	**Position**	***δ***_**C**_, **type** ^b^	***δ***_**H**_, **multiplicity,** *J* **in Hz**
1	174.0, C		11	197.5, C	
2	42.3, CH	2.45, brq (7.0)	12	34.3, CH_2_	2.36, t (6.9)
3	81.3, CH	4.07, m	13	37.0, CH_2_	1.75, t (6.9)
4	22.4, CH_2_	1.62, m	14	36.4, C	
		1.59, m			
5	32.3, CH_2_	1.76, m	15	12.9, CH_3_	1.04, d (7.0)
		1.61, m			
6	79.6, C		16	23.4, CH_3_	1.09, s
7	34.5, CH_2_	2.00, m	17	54.0, CH_2_	4.10, m
		1.60, m			4.06, m
8	23.6, CH_2_	2.27, m	17-OH		4.30, brt (4.7)
		2.24, m			
9	168.1, C		18	26.7, CH_3_	1.14, s
10	134.5, C		19	26.7, CH_3_	1.16, s
11	197.5, C		20	51.8, CH_3_	3.59, s

a 500 MHz for ^1^H, 125 MHz for ^13^C.

b From HSQC.

Diacarnusin C (**3**) and diacarnusin D (**4**) were isolated as oils and presented a positive HRESIMS molecular cluster ion [M + Na]^+^ of *m*/*z* 279.1206 and *m*/*z* 293.1353, respectively. The molecular ions corresponded to the molecular formulas, C_13_H_20_O_5_ for **3** and C_14_H_22_O_5_ for **4**, with four degrees of unsaturation each. The differences in their molecular formulas (14 mass units, CH_2_) and comparison of their ^1^H and ^13^C NMR spectral signals in DMSO-*d*_6_ clearly suggest that **4** (Table 3) is the methyl ester derivative of **3** (Table S6). While running their NMR spectra in DMSO-*d*_6_, **3** and **4** were spontaneously converted (**4** faster than **3**) into two isomeric products each (Figure 2). The structure of the natural products and their transformation products was elucidated from the spectra of the starting materials (**3** and **4**), mixtures, and end products (**3a**, **3b**, **4a**, and **4b**) in DMSO-*d*_6_ (Tables 3 and S7) and the spectrum of **3** in CDCl_3_ (Table 3).

**Table 3:** NMR Data of Diacarnusin C (**3**) and Diacarnusin D (**4**)

	**Diacarnusin C (3) in CDCl**_**3**_ ^a^		**Diacarnusin D (4) in DMSO-** ***d***_**6**_ ^a^		
**Position**	***δ***_**C**_, **type** ^b^	***δ***_**H**_, **multiplicity,** ***J*** **in Hz**	**Position**	***δ***_**C**_, **type** ^b^	***δ***_**H**_, **multiplicity,** ***J*** **in Hz**
1	178.4, C		1	173.6, C	
2	42.5, CH	2.73, m	2	41.9, CH_2_	2.70, dq (6.1, 7.1)
3	80.9, CH	4.16, dt (4.3, 7.8)	3	80.9, CH	4.06, q (6.1)
4	24.3, CH_2_	1.82, m	4	22.5, CH_2_	1.67, m
4’		1.79, m			
5	31.6, CH_2_	1.69, m	5	31.0, CH_2_	1.64, m
6	79.9, C		6	79.7, C	
7	42.4, CH_2_	2.55, m	7	41.5, CH_2_	2.46, m
7’		2.37, dd (14.3, 8.0)			2.37, dd (14.2, 7.7)
8	142.1, CH	6.76, ddd (16.0, 8.0, 7.5)	8	142.7, CH	6.72, dt (15.9, 7.5)
9	134.5, CH	6.10, d (16.0)	9	134.2, CH	6.06, dt (15.9, 1.2)
10	198.5, C		10	198.1, C	
11	26.8, CH_3_	2.26, s	11	27.0, CH_3_	2.20, s
12	13.3, CH_3_	1.29, d (7.1)	12	12.8, CH_3_	1.10, d (7.1)
13	21.6, CH_3_	1.28, s	13	21.3, CH_3_	1.20, s
			1-OCH_3_	51.9, CH_3_	3.61, s

a 500 MHz for ^1^H and 125 MHz for ^13^C.

b From HSQC.

**Figure 2:**
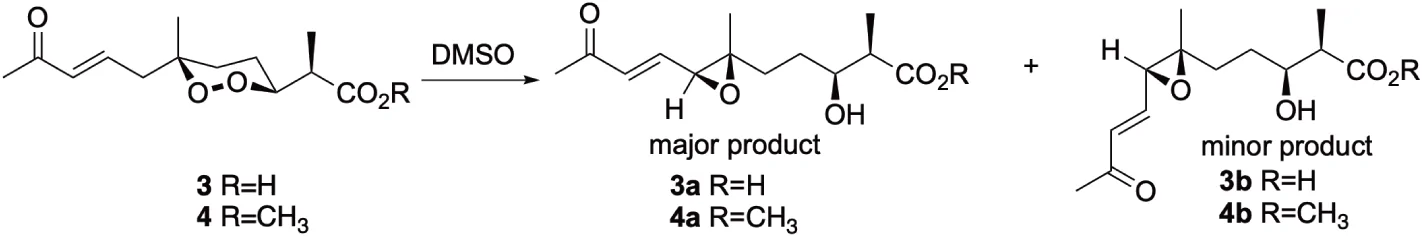
Spontaneous conversion of **3** and **4** to **3a**, **3b**, **4a,** and **4b**.

The NMR data of **3** in CDCl_3_ presented a conjugated ketone (δ_C_ 198.5), a conjugated trans-disubstituted double bond (δ_H_ 6.76, ddd, δ_C_ 142.1, and δ_H_ 6.10, d, δ_C_ 134.5), an allylic methylene (δ_H_ 2.55 and 2.37, δ_C_ 42.4), a quaternary oxygenated carbon (δ_C_ 79.9), and an oxygenated methine (δ_H_ 4.16, δ_C_ 80.9), all indicative of a cyclic endoperoxide moiety along with the two aliphatic methylene carbons (δ_C_ 31.6 and 24.3), an alpha-to-carbonyl methine (δ_H_ 2.73, δ_C_ 42.5), a carboxyl (δ_C_ 178.4), and three methyl groups: a methyl ketone (δ_H_ 2.26, δ_C_ 26.8), a tertiary methyl next to oxygen (δ_H_ 1.28, δ_C_ 21.6), and a secondary methyl (δ_H_ 1.29, δ_C_ 13.3). The latter structural elements were assembled by analysis of the 2D NMR data (COSY, HSQC, and HMBC, Figure 1 and Table 3) to the planar structure of **3**, which resembled the planar structure of negombatoperoxide B,^18^ but differed in the chemical shifts of CH_3_-12 and CH_3_-13. The chemical shifts of CH_3_-12 and CH_3_-13 of negombatoperoxide B,^13^ (δ_H_ 1.21, δ_C_ 12.3, and δ_H_ 1.12, δ_C_ 24.3, respectively) indicated a *syn* (*erythro*) relative configuration of the C-2, C-3 stereocenters and an equatorial methyl-13 on C-6, while the chemical shifts of the latter methyl groups in **3** (δ_H_ 1.29, δ_C_ 13.3, and δ_H_ 1.28, δ_C_ 21.6, respectively) indicated an *anti* (*threo*) relative configuration of the C-2, C-3 centers and an axial methyl-13 on C-6. While the specific rotation of negombatoperoxide B was positive (+80), indicating a 2*R*,3*R*,6*R* absolute configuration, which was recently verified by a total synthesis,^19^ the specific rotation of **3** was negative (−12.0), suggesting a 2*R*,3*S*,6*R* absolute configuration for diacarnusin C (**3**).

The structure of diacarnusin D (**4**) was similarly elucidated. Comparison of the 1D and 2D NMR spectra of **4** and **3** in DMSO-*d*_6_ (Tables 3 and S6, respectively), revealed differences in the chemical shifts of the signals at positions 1–4 and 12, as well as an additional signal of a methoxy group in the spectra of **4**. The remaining chemical shifts were similar. A full assignment of the NMR signals of **4** (Table S7 and Figure 2) proved that **4** is indeed the methyl ester derivative of **3**. Comparison of the chemical shifts in DMSO-*d*_6_ of CH_3_-13 in **4** (δ_H_ 1.20, δ_C_ 21.3) and **3** (δ_H_ 1.20, δ_C_ 21.4) indicates that the orientation of CH_3_-13 in **4** is axial, as in **3**. Comparison of the chemical shifts of CH_3_-12 in **4** and **3** (δ_H_ 1.10, δ_C_ 12.8 versus δ_H_ 1.07, δ_C_ 13.4, respectively) did not match, probably due to different populations of the conformers of the free acid and methyl ester in DMSO. However, comparison of the chemical shifts of CH_3_-12 in **4** with those of sigmosceptrellin B methyl ester (**8**) in DMSO-*d*_6_ (δ_H_ 1.10, δ_C_ 12.8 versus δ_H_ 1.08, δ_C_ 12.7, respectively) allowed to confirm the *anti* (*threo*) relative configuration of C-2 and C-3, in **4**. The negative specific rotation measured for **4** (−5) and **8** (−36.1) inferred the same absolute configuration for **4** and **8**. Based on these arguments, the 2*R*,3*S*,6*R* absolute configuration is indicated for diacarnusin D (**4**).

As mentioned above, compounds **3** and **4** spontaneously converted into two isomeric products each: the major (**3a** and **4a**) and minor (**3b** and **4b**) derivatives (Figure 2). The structure of both isomers could be elucidated from the mixtures. The molecular ions in the mass spectrum and molecular formulas of the transformation products remained identical with that of the natural products (HRESIMS: [M + Na]^+^, *m*/*z* 279.1204 for the mixture of **3**, **3a,** and **3b;** and *m*/*z* 293.1353 for the mixture of **4**, **4a,** and **4b**, C_14_H_22_O_5_. HRESI LCMS analysis of the mixture of **4**, **4a,** and **4b** displayed three peaks (Figure S42) with *R_t_* of 11.8 min, *m*/*z* 293.1359, *R_t_* of 11.5, *m*/*z* 293.1360, and *R_t_* of 11.2, *m*/*z* 293.1362, C_14_H_22_O_5_, for, **4**, **4b,** and **4a**, respectively. Inspection of the ^1^H and ^13^C chemical shifts of the transformation products, **3a** and **3b,** and **4a** and **4b**, of **3** and **4** (Tables S6, S6a, S6b, S7, S8a and S8b), revealed that the cyclic endoperoxide of compounds **3** and **4** (δ_H_ 4.00, δ_C_ 81.4, and δ_C_ 79.6 of **3** and δ_H_ 4.06, δ_C_ 80.9, and δ_C_ 79.7 of **4**) was transformed into hydroxy epoxides (δ_H_ 3.55, δ_C_ 70.9, δ_C_ 64.1, and δ_H_ 3.45, δ_C_ 60.9 for **3a**; δ_H_ 3.53, δ_C_ 70.9, δ_C_ 64.4, and δ_H_ 3.44, δ_C_ 61.8 for **3b**; δ_H_ 3.61, δ_C_ 70.8, δ_C_ 64.0, and δ_H_ 3.45, δ_C_ 60.8 for **4a**; and δ_H_ 3.57, δ_C_ 71.1, δ_C_ 64.3, and δ_H_ 3.44, δ_C_ 61.8 for **4b**). Full analyses of the NMR data of **3a** and **3b,** and **4a** and **4b** (Tables S6a, S6b, S8a, and S8b, respectively, and Figure 2) established the four compounds as 3-hydroxy-6,7-epoxy derivatives of **3** and **4**, respectively. Examining the ^13^C chemical shifts of CH_2_–5 and CH_3_-13 of **3a** and **3b,** and **4a** and **4b** (δ_C_ 34.5 and 16.6, respectively, for **3a**; δ_C_ 29.0 and 22.0, respectively, for **3b**; δ_C_ 34.5 and 16.6, respectively, for **4a**; and δ_C_ 29.1 and 22.0, respectively, for **4b**) and the NOE between H-7 and H_3_-13 in the NOESY spectrum of **3b** allowed to conclude that **3a** and **4a** are *E* epoxides, while **3b** and **4b** are *Z* derivatives.

Diacarnusins E and F (**5** and **6**) were eluted as a single peak from the HPLC column. They possessed an HRESIMS molecular cluster peak, [M + Na]^+^, of *m*/*z* 335.1456, corresponding to a molecular formula of C_16_H_24_O_6_, with five degrees of unsaturation. Their IR spectrum presented absorptions of two carbonyl groups at 1711 and 1736 cm^–1^, corresponding to a saturated carboxylic acid and γ-lactone, respectively, which were also evident from the ^13^C NMR signals (δ_C_ 178.3 and 176.8, respectively). Careful examination of the ^1^H and ^13^C NMR signals in CDCl_3_ of the sample (Table 4) revealed a dabbling of the carbon signals that were afterward assigned to the γ-lactone moiety and one of the vinyl carbons (C-9), suggesting that the chiral carbon (C-10) is composed of ca. 2:1 mixture of two epimers (C-9, δ_C_ 124.0 broaden; C-10 δ_C_ 85.2/84.8, 2:1; C-11 δ_C_ 34.09/34.07, 2:1 and C-16, δ_C_ 26.52/26.56 and δ_H_ 1.491/1.488, 2:1). The ^1^H NMR spectra of **5** and **6** (Table 4) resembled that of **3** (Table 3), showing similar chemical shifts and *J*-coupling of the signals of the H-2 to H-9 and of the C-2 and C-6 methyl signals. Detailed analysis of the 2D NMR data (COSY, HSQC, and HMBC, Figure 2 and Table S9) of **5** and **6** in CDCl_3_, established the identity of the C-1 to C-9 segment to that of **3**, and the γ-lactone moiety: H-9 (δ_H_ 5.61) presented

**Table 4:** NMR Data of Diacarnusins E (**5**) and F (**6**) in CDCl_3_ ^a^

**Position**	**δ**_**C**_, **type** ^b^	***δ***_**H**_, **multiplicity,** ***J*** **in Hz**	**Position**	**δ**_**C**_, **type** ^b^	***δ***_**H**_, **multiplicity,** ***J*** **in Hz**
1	178.3, C		9	135.9, CH	5.61, brd (15.8)
2	42.6, CH	2.68, brm	10	85.3, C (2)	
				84.8, C (1)	
3	81.0, CH	4.14, brdt (5.2, 7.8)	11	34.09, CH_2_	2.18, m
				34.07, CH_2_	2.07, dt (12.8, 8.6)
4	23.2, CH_2_	1.77, m	12	29.0, CH_2_	2.55, dd (7.8, 8.6)
5	31.5, CH_2_	1.64, m	13	176.8, C	
6	80.0, C		14	13.2, CH_3_	1.27, d (7.2)
7	42.2, CH_2_	2.30, m	15	21.1, CH_3_	1.24, brs
7’		2.20, m			
8	124.0, CH	5.67, dt (15.8, 6.9)	16	26.52, CH_3_	1.491, s (2)
				26.56, CH_3_	1.488, s (1)

a 500 MHz for ^1^H, 125 MHz for ^13^C.

b From HSQC.

HMBC correlations with the doubly oxygenated quaternary carbon (C-10), CH_2_–11 and CH_3_-16, CH_2_–11 (δ_H_ 2.18, 2.07) presented COSY correlations with CH_2_–12 (δ_H_ 2.55) ,and H_2_-11 and H_2_-12 presented HMBC correlations with the lactone carboxyl group C-13 (δ_C_ 176.8). The chemical shift of C-10 (85.2/84.8), IR absorption (1736 cm^–1^) and the molecular formula of (C_16_H_24_O_6_) support the existence of a γ-lactone moiety in **5** and **6**. The planar structure of **5** and **6** turned out to be identical to that of negombatoperoxides C and D, isolated from the Formosan sponge *Negombata corticata*.^18,19^ Comparison of the chemical shifts of the stereochemistry-indicative NMR signals, CH_3_-14 and CH_3_-15, of **5** and **6** (δ_H_ 1.27, δ_C_ 13.2 versus δ_H_ 1.24, δ_C_ 21.1, respectively) with those of **3** (δ_H_ 1.29, δ_C_ 13.3 and δ_H_ 1.28, δ_C_ 21.6, respectively) revealed close similarity indicating an *anti* (*threo*) relative configuration of the C-2, C-3 centers and an axial methyl-13 on C-6, but differed from those of CH_3_-13 and CH_3_-14 (δ_H_ 1.21, δ_C_ 12.8 and δ_H_ 1.12, δ_C_ 24.2, respectively) of negombatoperoxides C and D,^18,19^ which indicated a *syn* (*erythro*) relative configuration of the C-2, C-3 chiral centers and an equatorial methyl-14 on C-6. Thus, **5** and **6** are new natural products. The specific rotation of **5** and **6** was −22.3, like that of **3** (−12.0) and opposite to that of negombatoperoxides C and D (+62),^13^ inferring a 2*R*,3*S*,6*R* absolute configuration for diacarnusin E and F (**5** and **6**).

Sigmosceptrellin B (**7**) was the major endoperoxide component (309.0 mg) isolated from the sponge sample, followed by sigmosceptrellin B methyl ester (**8**, 140.0 mg), diacanusin A (**1**, 20.7, mg), (−)-*ent*-muqubilone (**11**, 20.6 mg), and the minor metabolites; methyl diacarnoate A (**10**, 4.5 mg), diacarnusin C (**3**, 3.7 mg), diacarperoxide A (**9**, 3.2 mg), diacarnusin E and F (**5** and **6**, 3.4 mg), diacarnusin B (**2**, 1.3 mg), and diacarnusin D (**4**, 0.9 mg). The isolated metabolites were tested for their toxicity against a panel of seven cancer cell lines: cervical cancer, C33A; colorectal cancer, HCT116; melanoma, A375; prostate cancer, LnCap; lung cancer, A549; cervical cancer, HELA; and colorectal cancer, HT29, and the transformed human embryonic kidney cell line, 293T (Table 5). Only the *nor*-sesterterpene acids **1** and **7** presented micromolar IC_50_’s against the entire panel of cell lines, with no selectivity. The methyl ester derivative of **7**, sigmosceptrellin B methyl ester (**8**), was 1 order of magnitude less active against the panel of cancer cell lines, and compound **10** was almost 2 orders of magnitude less active. The rest of the tested compounds were essentially inactive. This pattern of cytotoxicity is in accordance with previous studies on this group of compounds.^6^ It seems that although the endoperoxide moiety is the warhead of the cytotoxic activity of this group of compounds, the decalin moiety and, to a lesser extent, a cyclohexane ring are still essential for targeting the compounds to the site of action. The demonstrated decomposition of diacarnusins C (**3**) and D (**4**) reinforces the common notion that the endoperoxide can easily produce oxy-diradicals that isomerize to carbon-centered free radicals. These latter radicals might react with membranes, proteins, and noncoiled DNA of either mammalian cells or malaria parasites.^20^ Terpene endoperoxides have been examined over the years mainly for cytotoxicity to cancer cell lines, yeasts, malaria parasites, and bacteria.^6^ While the bicyclic *nor*-sesterterpenes were more potent against cancer cell lines, yeasts, and bacteria, monocyclic *nor*-sesterterpenes and diterpenes were more cytotoxic against malaria parasites.^6^

**Table 5:** Cytotoxicity of the Isolated Compounds (IC_50_, μΜ; ±SD)^a^

	**Cell Line Type**							
**Compound**	**C33A**	**HCT116**	**A375**	**LnCap**	**A549**	**HELA**	**293T**	**HT29**
**1**	0.95 ± *0.06*	1.31 ± *0.06*	1.21 ± *0.05*	0.82 ± *0.03*	1.09 ± *0.04*	1.47 ± *0.07*	0.99 ± *0.08*	3.12 ± *0.29*
**3**	>100	>100	>100	>100	>100	97.76 ± *17.64*	82.81 ± *6.20*	>100
**4**	>100	>100	>100	>100	>100	>100	>100	>100
**5**/**6**	>100	>100	>100	>100	>100	>100	>100	>100
**7**	1.31 ± *0.10*	1.98 ± *0.17*	1.74 ± *0.15*	1.02 ± *0.32*	1.60 ± *0.09*	2.47 ± *0.16*	1.32 ± *0.08*	4.19 ± *0.29*
**8**	9.42 ± *0.44*	15.04 ± *1.04*	12.24 ± *0.46*	7.99 ± *0.42*	11.50 ± *0.59*	15.96 ± *2.42*	7.89 ± *0.42*	17.55 ± *1.01*
**9**	>100	>100	>100	>100	>100	>100	>100	>100
**10**	33.67 ± *1.54*	43.60 ± *2.98*	46.10 ± *3.39*	60.04 ± *4.00*	52.43 ± *4.18*	32.49 ± *1.74*	27.71 ± *1.78*	79.24 ± *6.91*
**11**	>100	>100	>100	>100	>100	>100	>100	>100

a Cell lines: C33A, cervical cancer; HCT116, colorectal cancer; A375, melanoma; LNCaP, prostate cancer; A549, lung cancer; HeLa, cervical cancer; 293T, embryonic kidney; HT29, colorectal cancer.

Examining the structures of the different *nor*-terpene endoperoxides that were isolated from sponges of the genera *Diacarnus*, *Sigmospecterella*, *Negombata*, *Latrunculia*, *Mycale*, *Hyrtios*, and their biological activities suggests that geranyl farnesyl pyrophosphate (GFP, Figure 3) is the precursor of all these metabolites. It seems that the initial step of this process is the cyclization of GFP to either decalin or cyclohexane and/or the elimination of the pyrophosphate to produce three types of polyene metabolites: decalin, cyclohexane, and linear sesterterpenes (Figure 3). The latter three groups of sesterterpenes are most probably first losing a carbon by oxidation of the terminal double bond and then oxidized to the groups of the *nor*-sesterterpene endoperoxides. Alternatively, cyclohexane containing sesterterpene product is oxidized to a tetraene, and the new double bond is oxidatively cleaved to a truncated product, which after *endo*-peroxidation yields the *nor*-diterpene series of products. The four groups of biologically active metabolites are further oxidized at different positions to yield epoxide or allylic alcohol derivatives that are biologically active. However, it seems that the powerful oxidation enzymes in the sponge tissue continue to oxidize the active metabolites and degrade them to the nonactive metabolites that we isolate from these sponges, aside from the biologically active ones. The ongoing and yet unpublished investigation on the chromosomal genome sequence of the marine sponge *D. erythraeanus*, and its associated microbial metagenome sequences, may help resolve this issue.

**Figure 3:**
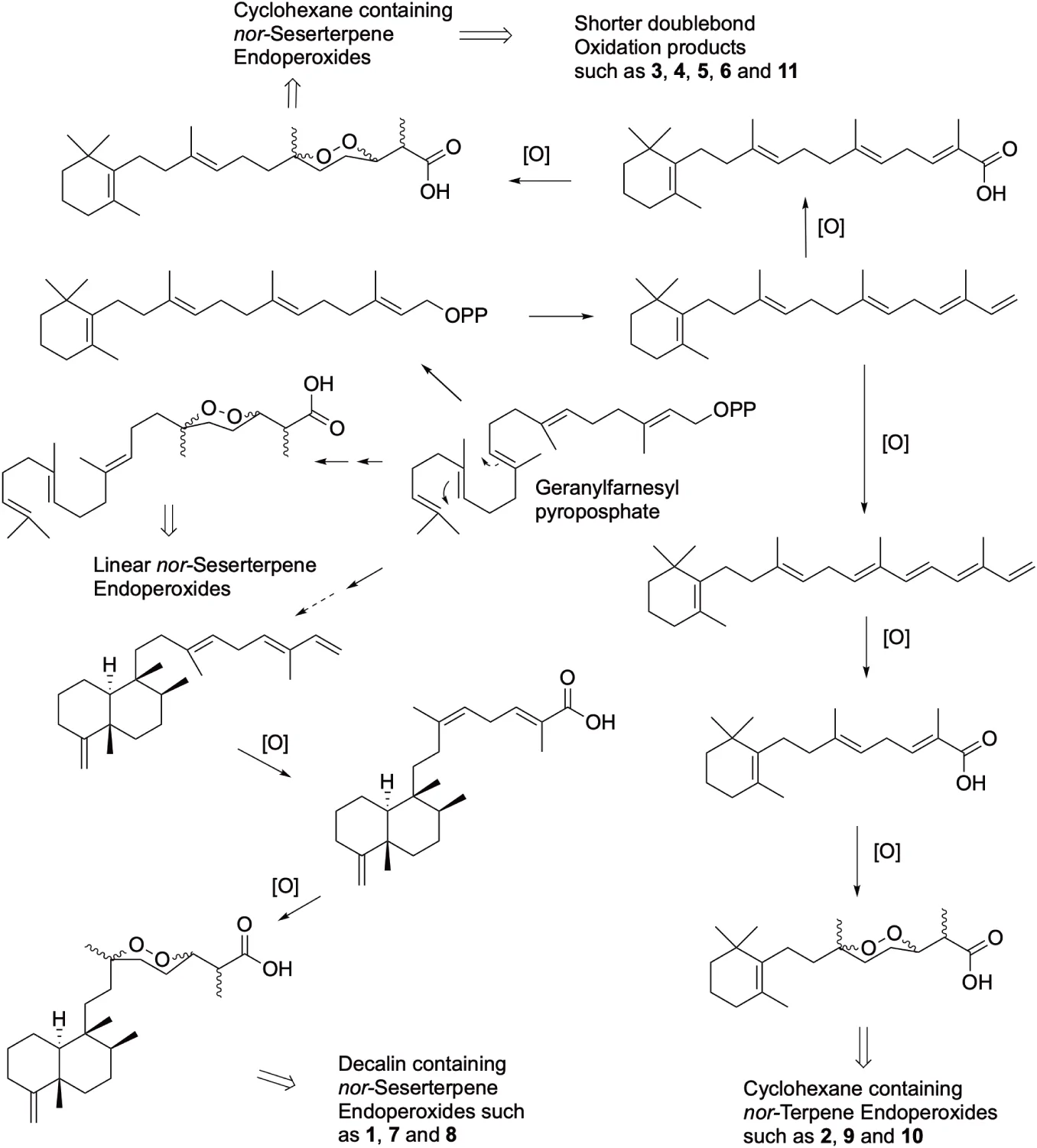
Biosynthetic routes leading to the different terpene endoperoxide metabolites.

In conclusion, six new *nor-*terpene endoperoxides, diacarnusins A–F (**1**-**6**), along with five known *nor-*terpene endoperoxides, were isolated from the extract of the Red Sea sponge *D. erythraeanus*, and their structures were elucidated. Only the new *nor*-sesterterpene diacarnusin A (**1**) exhibited significant cytotoxicity against cancer cell lines, comparable to that of the known, sigmosceptrellin B (**7**).

## Experimental Section

### General Experimental Procedures

Specific rotation values were obtained on a Jasco P-1010 polarimeter at the sodium D line (589 nm). UV spectra were recorded on an Agilent 8453 spectrophotometer (Agilent, Santa Clara, CA, USA). IR spectra were recorded on a Bruker Tensor 27 FT-IR instrument (Bruker, Billerica, MA, USA). NMR spectra were recorded on Bruker Avance III spectrometers (Bruker, Karlsruhe, Germany) at 500.13 MHz for ^1^H and 125.76 MHz for ^13^C. Chemical shifts were referenced to TMS, δ_H_ and δ_C_ = 0 ppm. DEPT, COSY-45, gTOCSY, gROESY, gHSQC, and gHMBC spectra were recorded using standard Bruker pulse sequences. ESI low- and high-resolution mass spectra and MS/MS spectra were recorded on a Waters (Waters, Milford, MA, USA) Xevo G2-XS QTOF instrument equipped with Acquity Hi-Class UPLC (binary solvent manager) with an FTN sample manager, column manager, and PDA detector, using a 2.1 × 50 mm BEH C18 (1.7 μm) column and a flow rate of 0.1–0.3 mL/min. HPLC separations were performed on an Agilent 1100 Series HPLC system (Agilent, Santa Clara, CA, USA).

### Biological Material

The sample of the sponge *D. erythraeanus* was collected in Eilat, the Red Sea, on July 16, 2019, at a depth of 6 m. The sponge was identified by Lilach Raijman-Nagar and Prof. Micha Ilan. A voucher of the sponge sample (Po-26700) is stored in the Steinhardt Museum of Natural History, Tel Aviv University.

### Extraction and Isolation Procedure

A freeze-dried sample (84.3 g) of the sponge *D. erythraeanus* was extracted using a 45:45:10 mixture of EtOAc/MeOH/H_2_O to yield 15.6 g of dry extract. The extract was then separated by polarity using a CombiFlash EZ Prep with a reverse-phase C-18 column (Teledyne ISCO, 150 g HP C18, 150 mg–3 g sample). The separation process involved a gradient elution, starting with 100% water to 100% MeOH over 60 min, followed by 15 min of 100% MeOH, and finishing with EtOAc for 15 min (repetitive 5 × 3 g, see Flow Diagram S1 in Supporting Information). This procedure resulted in 21 distinct fractions, which were collected using an autosampler collector. Following ^1^H NMR analysis, the fractions were combined into four new fractions based on their spectral similarities: Fractions 1–7 were combined and designated, fraction RA(2)-47/1; Fractions 8–11 were combined and designated, fraction RA(2)-47/2; Fractions 12–16 were combined and designated, fraction RA(2)-47/3; and fractions 17–21 were combined and designated, fraction RA(2)-47/4. Fraction RA(2)-47/3 (see Flow Diagram S1) was further purified using size-exclusion chromatography on a Sephadex LH-20 column with MeOH, resulting in 23 fractions, which were combined based on the similarity of their ^1^H NMR spectra into 4 main fractions: RA(2)-53/1, RA(2)-53/2, RA(2)-53/3, and RA(2)-53/4. The cytotoxic fraction RA(2)-53/4 was subsequently separated on a small C18 reverse-phase column (Teledyne ISCO, 15.5 g HP C18, 15.5 mg −310 mg sample) using the CombiFlash EZ Prep system (see Flow Diagram S2). The separation gradient involved 100% water to 100% MeOH over 60 min, followed by 10 min of 100% MeOH and 10 min of 100% EtOAc. This process yielded 24 fractions, which were collected by an autosampler collector. The fractions were then combined based on ^1^H NMR analysis into fractions RA(2)-157/7–13, RA(2)-157/14, RA(2)-157/18–19, and RA(2)-157/20–22. Fraction RA(2)-157/7–13 was dissolved in MeOH and subjected to separation on a semipreparative reverse-phase HPLC RP-18 column (YMC-Pack ODS-AQ, 5 μm, 250 × 10 mm) using a 35:65 ETOAC/H_2_O. This process yielded the novel compounds diacarnusin C (**3**, 3.7 mg, 14.3 min, C_13_H_20_O_5_, 0.02% yield of crude extract) and diacarnusin D (**4**, 0.9 mg, 42.3 min, C_14_H_22_O_5_, 0.006% yield). Fraction RA(2)-157/14 was also dissolved in MeOH and separated on a semipreparative reverse-phase HPLC RP-C18 column (YMC-Pack ODS-AQ, 5 μm, 250 × 10 mm) with a 50:50 MeCN/H_2_O, resulting in the novel compound diacarnusin B (**2**, 1.3 mg, 30.8 min, C_20_H_32_O_6_, 0.008% yield). Fraction RA(2)-157/18–19 was combined, dissolved in MeOH, and separated on a semipreparative reverse-phase HPLC RP-18 column (YMC-Pack ODS-AQ, 5 μm, 250 × 10 mm) using a 70:30 MeCN/H_2_O, yielding diacarperoxide A (**9**, 3.2 mg, 30.4 min, C_20_H_32_O_5_, 0.02% yield). Finally, Fractions 20–22 were combined, dissolved in MeOH, and separated on a semipreparative reverse-phase HPLC RP-18 column (YMC-Pack ODS-AQ, 5 μm, 250 × 10 mm) using a 73:27 MeCN/H_2_O, resulting in the known compound methyl dicarnoate A (**10**, 4.5 mg, 30.2 min, C_20_H_32_O_5_, 0.03% yield).

Fraction RA(2)-47/4 was further purified using size exclusion chromatography on a Sephadex LH-20 column with a 1:1 MeOH:CHCl_3_ mixture, producing 24 fractions which were combined based on their ^1^H NMR spectrum, resulting in three fractions: RA(2)-58/1, RA(2)-58/2, and RA(2)-58/3. The cytotoxic fraction RA(2)-58/3 was separated on a C18 reverse-phase column (Teledyne ISCO, 150 g HP C18, 150 mg −3 g sample) using the CombiFlash EZ Prep system, applying a gradient starting with 100% water and transitioning to 100% MeOH over 60 min, followed by 15 min of 100% MeOH and 15 min of 100% EtOAc. This process resulted in the isolation of the known compounds sigmosceptrellin B (**7**, C_24_H_40_O_4_, 309 mg, 2% yield of crude extract) and sigmosceptrellin B methyl ester (**8**, C_25_H_42_O_4_ 140 mg, 0.9% yield) among a total of 14 fractions, which were collected by an autosampler collector, fractions RA(2)-151/1–14. Fraction RA(2)-151/3 was further purified using a semipreparative reverse-phase HPLC RP-18 column (YMC-Pack ODS-AQ, 5 μm, 250 × 10 mm) with a 40:60 MeCN/H_2_O, yielding the novel compounds diacarnusins E and F as a mixture of epimers (**5** and **6**, 8.5 mg, 17.3 min, C_16_H_24_O_6_, 0.05% yield). Fraction RA(2)-151/4 was similarly separated on a semipreparative reverse-phase HPLC RP-18 column (YMC-Pack ODS-AQ, 5 μm, 250 × 10 mm) using a 60:40 MeCN/H_2_O, resulting in the known (−)-*ent*muqubilone (**11**, 7.9 mg, 20.6 min, C_24_H_40_O_6_, 0.1% yield). Fraction RA(2)-151/10 was also separated on a semipreparative reverse-phase HPLC RP-18 column (YMC-Pack ODS-AQ, 5 μm, 250 × 10 mm) with a 90:10 MeCN/H_2_O, yielding the novel compound diacarnusin A (**1**, 16.3 mg, 20.7 min, C_24_H_40_O_4_, 0.1% yield).

#### Diacarnusin A (**1**)

A colorless oil material; [α]_D_^25^ +17 (c 0.05, MeOH); UV (MeOH) λ_max_ (log *ε*) 226 nm (2.68); IR (ATR Diamond) ν_max_ 2919, 2851, 1737, 1635, 1450, 1160 cm^–1^; For NMR data see Table S1; HRESIMS *m*/*z* 415.2817 ([M + Na]^+^, calcd. for C_24_H_40_O_4_Na *m*/*z* 415.2824).

#### Diacarnusin B (**2**)

A colorless oil material; [α]_D_^25^ +18 (c 0.09, MeOH); UV (MeOH) λ_max_ (log *ε*) 244 nm (1.35); IR (ATR Diamond) ν_max_ 3431, 2948, 1737, 1660, 1455 cm^–1^; For NMR data see Table S3; HRESIMS *m*/*z* 391.2093 ([M + Na]^+^, calcd. for C_20_H_32_O_6_Na *m*/*z* 391.2097).

#### Diacarnusin C (**3**)

A colorless oil material; [α]_D_^25^ −12 (c 0.33, CHCl_3_); UV (MeOH) λ_max_ (log *ε*) 221 nm (3.20); IR (ATR Diamond) ν_max_ 2976, 2942, 2881, 2341, 2255, 1717, 1675, 1375, 1258 cm^–1^; For NMR data see Tables S5 and S6; HRESIMS *m*/*z* 279.1206 ([M + Na]^+^, calcd. for C_13_H_20_O_5_Na *m*/*z* 279.1208).

#### Diacarnusin D (**4**)

A colorless oil material; [α]_D_^25^ −5 (c 0.04, MeOH); UV (MeOH) λ_max_ (log *ε*) 221 nm (3.0); IR (ATR Diamond) ν_max_ 2953, 1732, 1674, 1570 cm^–1^; For NMR data see Table S7; HRESIMS *m*/*z* 293.1353 ([M + Na]^+^, calcd. for C_14_H_22_O_5_Na *m*/*z* 293.1365).

#### Diacarnusin E and F (**5** and **6**)

A colorless oil material; [α]_D_^25^ −22 (c 0.05, MeOH); UV (MeOH) λ_max_ (log ε) 233 nm (2.6); IR (ATR Diamond) ν_max_ 2974, 2939, 1736, 1711, 1456, 1417 cm^–1^; For NMR data see Table S9; HRESIMS *m*/*z* 335.1456 ([M + Na]^+^, calcd. for C_16_H_24_O_6_Na *m*/*z* 335.1471).

### Cell Lines and Growth Media

All human cell lines were acquired from the American Type Culture Collection (ATCC). C33A was grown in MEM-Eagle supplemented with 10%FBS, 1% penicillin-streptomycin, 1% GlutaMAX, and 1% sodium pyruvate. The 293T, HeLa, and LNCaP cells were grown in DMEM supplemented with 10%FBS and 1% penicillin-streptomycin. All other cell lines (HCT116, A375, A549, and HT29) were grown in RPMI supplemented with 10% FBS and 1% penicillin-streptomycin.

### Cellular Toxicity Assays

A 9-point, 2-fold dose–response series with 100 μM as the upper limit and a DMSO-only control point was generated using an Echo 550 liquid handler (Labcyte Inc.) in 384-well plates. Subsequently, each cell line was seeded (2,500 cells per well) using a Multidrop Combi dispenser (Thermo Fisher Scientific) on top of the compounds. Each concentration was tested in triplicate. Plates were then incubated at 37 °C and 5% CO_2_ for 48 h. Cell viability was assessed by adding CellTiter-Glo (Promega) to the reaction. The luminescence signal was measured on a PHERAstar FS multimode plate reader (BMG Labtech).

Nonlinear regression (curve fitting) was performed using the [Inhibitor] vs response – Variable slope equation within GraphPad Prism version 10.4.1 for Windows (GraphPad Software, Boston, Massachusetts, USA, www.graphpad.com)

## Supplementary Material



## Data Availability

The NMR data for diacarnusins A–F have been deposited in the Natural Products Magnetic Resonance Database (NP-MRD; www.np-mdr.org) and can be found at NP0354126 (diacarnusin A), NP0354127 (diacarnusin B), NP0354128 (diacarnusin C), NP0354129 (diacarnusin D), and NP0354130 (diacarnusins E & F).

## Notes

The sponge pictures, flow diagrams of the natural product isolation, 1D and 2D NMR spectra of all compounds at least one NMR solvent, including NMR full tables in an alternative solvent, HR ESI mass spectra of all natural products, and cytotoxicity curves of natural products (PDF)

## Notes

Dedicated to the late Prof. Yoel Kashman, our colleague and mentor that passed away on July 1, 2025, at the age of 87.
