# Supplementary material for: Diacarnusins A‑F, nor-Terpene Endoperoxides of the Red Sea Sponge Diacarnus erythraeanus
Source: J Nat Prod. 2026 Jul 17;89(8):2314–21. doi: 10.1021/acs.jnatprod.6c00510 (PMC13532193; doi:10.1021/acs.jnatprod.6c00510)

Supporting Information

Diacarnusins A-F, *nor*-Terpene Endoperoxides of the Red Sea Sponge *Diacarnus erythraeanus*

*Rania Hasib Afana[1], Lilach Raijman-Nagar[2], Micha Ilan[2], Shmuel Carmeli[1]**

[1]School of Chemistry, Raymond and Beverly Sackler Faculty of Exact Sciences, Tel-Aviv University, Ramat Aviv Tel-Aviv 69978, Israel.

[2]Department of Zoology, George S. Wise Faculty of Life Sciences, Tel-Aviv University, Ramat Aviv Tel-Aviv 69978, Israel.

Corresponding Author: *Shmuel Carmeli − Raymond and Beverly Sackler School of Chemistry, Faculty of Exact Sciences, Tel Aviv University, Tel Aviv 6997801, Israel; orcid.org/0000-0002-9641-9943; Email: carmeli@tauex.tau.ac.il.

Table of Content

Page	Content
8	Figure S1. Photographs of *D. erythreanus* in situ and after collection
9	Figure S2. *nor*-Diterpenes and *nor*-sesterterpene endoperoxides isolated from extracts *of* Red Sea *D. erythreanus* samples
11	Flow Diagram S1. Reversed phase separation of the crude extract of *D. erythreanus*
12	Flow Diagram S2. Reversed phase HPLC separation of the fraction RA(2)-53/4
13	Flow Diagram S3. Reversed phase HPLC separation of the fraction RA(2)-58/3
14	Figure S3. ^{1}H NMR (500 MHz, $CDCl_3$) spectrum of Diacarnusin A (**1**)
15	Figure S4. ^{13}C NMR (125 MHz, $CDCl_3$) spectrum of Diacarnusin A (**1**)
16	Figure S5. HSQC (500/125 MHz, $CDCl_3$) 2D spectrum of Diacarnusin A (**1**)
17	Figure S6. HMBC (500/125 MHz, $CDCl_3$) 2D spectrum of Diacarnusin A (**1**)
18	Figure S7. COSY (500 MHz, $CDCl_3$) 2D spectrum of Diacarnusin A (**1**)
19	Figure S8. NOESY (500 MHz, $CDCl_3$) 2D spectrum of Diacarnusin A (**1**)
20	Figure S9. HR ESI MS of Diacarnusin A (**1**)
21	Table S1. NMR Data of Diacarnusin A (**1**) in $CDCl_3$[a]
22	Table S2. Comparison of the NMR Data of Diacarnusin A (**1**) and Sigmosceptrellin B (**7**) in $CDCl_3$[a]
25	Figure S10. ^{1}H NMR (500 MHz, DMSO-d_6) spectrum of Diacarnusin B (**2**)
26	Figure S11. ^{13}C NMR (125 MHz, DMSO-d_6) spectrum of Diacarnusin B (**2**)

27	Figure S12. HSQC (500/125 MHz, DMSO-d_6) 2D spectrum of Diacarnusin B (**2**)
28	Figure S13. HMBC (500/125 MHz, DMSO-d_6) 2D spectrum of Diacarnusin B (**2**)
29	Figure S14. COSY (500 MHz, DMSO-d_6) 2D spectrum of Diacarnusin B (**2**)
30	Figure S15. NOESY (500 MHz, DMSO-d_6) 2D spectrum of Diacarnusin B (**2**)
31	Figure S16. HR ESI MS of Diacarnusin B (**2**)
32	Table S3. NMR data of diacarnusin B (2) in DMSO-d_6[a]
33	Table S4. Comparison of the NMR data of Diacarnusin B (**2**) and Diacarperoxide A (**9**) in DMSO-d_6[a]
34	Figure S17. ^{1}H NMR (500 MHz, $CDCl_3$) spectrum of Diacarnusin C (**3**)
35	Figure S18. ^{13}C NMR (125 MHz, $CDCl_3$) spectrum of Diacarnusin C (**3**)
36	Figure S19. HSQC (500/125 MHz, $CDCl_3$) 2D spectrum of Diacarnusin C (**3**)
37	Figure S20. HMBC (500/125 MHz, $CDCl_3$) 2D spectrum of Diacarnusin C (**3**)
38	Figure S21. COSY (500 MHz, $CDCl_3$) 2D spectrum of Diacarnusin C (**3**)
39	Figure S22. ^{1}H NMR (500 MHz, DMSO-d_6) spectrum of Diacarnusin C (**3**)
40	Figure S23. ^{1}H NMR (500 MHz, DMSO-d_6) spectrum of the mixture of Diacarnusin C (**3**) and the transformation products **3a** and **3b**
41	Figure S24. ^{13}C NMR (125 MHz, DMSO-d_6) spectrum of Diacarnusin C (**3**)
42	Figure S25. ^{13}C NMR (125 MHz, DMSO-d_6) spectrum of the mixture Diacarnusin C (**3**) and the transformation products **3a** and **3b**
43	Figure S26. HSQC (500/125 MHz, DMSO-d_6) 2D spectrum of the mixture of Diacarnusin C (**3**) and the transformation products **3a** and **3b**

44	Figure S27. HMBC (500/125 MHz, DMSO-d_6) 2D spectrum of the mixture of Diacarnusin C (**3**) and the transformation products **3a** and **3b**
45	Figure S28. COSY (500 MHz, DMSO-d_6) 2D spectrum of the mixture of Diacarnusin C (**3**) and the transformation products **3a** and **3b**
46	Figure S29. TOCSY (500 MHz, DMSO-d_6) 2D spectrum of the mixture of Diacarnusin C (**3**) and the transformation products **3a** and **3b**
47	Figure S30. NOESY (500 MHz, DMSO-d_6) 2D spectrum of the mixture of Diacarnusin C (**3**) and the transformation products **3a** and **3b**
48	Figure S31. HR ESI MS of the mixture of Diacarnusin C (**3**) and the transformation products **3a** and **3b**
49	Table S5. NMR Data of Diacarnusin C (**3**) in $CDCl_3$[a]
50	Table S6. NMR Data of diacrnusin C (**3**) in DMSO-d_6[a]
51	Table S6a. NMR Data of Diacrnusin C major product (**3a**) in DMSO-d_6[a]
52	Table S6b. NMR Data of Diacrnusin C minor product (**3b**) in DMSO-d_6[a]
53	Figure S32. ^{1}H NMR (500 MHz, DMSO-d_6) spectrum of Diacarnusin D (**4**)
54	Figure S33. ^{1}H NMR (500 MHz, DMSO-d_6) spectrum of the mixture of Diacarnusin D (**4**) and the transformation products I **4a** and **4b**
55	Figure S34. ^{1}H NMR (500 MHz, DMSO-d_6) spectrum of the mixture of Diacarnusin D (**4**) and the transformation products II **4a** and **4b**
56	Figure S35. ^{13}C NMR (125 MHz, DMSO-d_6) spectrum of the mixture Diacarnusin D (**4**) and the transformation products I **4a** and **4b**
57	Figure S36. ^{13}C NMR (125 MHz, DMSO-d_6) spectrum of the mixture Diacarnusin D (**4**) and the transformation products II **4a** and **4b**

58	Figure S37. HSQC (500/125 MHz, DMSO-d_6) 2D spectrum of the mixture of Diacarnusin D (**4**) and the transformation products I **4a** and **4b**
59	Figure S38. HSQC (500/125 MHz, DMSO-d_6) 2D spectrum of the mixture of Diacarnusin D (**4**) and the transformation products II **4a** and **4b**
60	Figure S39. HMBC (500/125 MHz, DMSO-d_6) 2D spectrum of the mixture of Diacarnusin D (**4**) and the transformation products I **4a** and **4b**
61	Figure S40. COSY (500 MHz, DMSO-d_6) 2D spectrum of the mixture of Diacarnusin D (**4**) and the transformation products I **4a** and **4b**
62	Figure S41. COSY (500 MHz, DMSO-d_6) 2D spectrum of the mixture of Diacarnusin D (**4**) and the transformation products II **4a** and **4b**
63	Figure S42. HR ESI MS of the mixture of Diacarnusin D (**4**) and the transformation products **4a** and **4b**
64	Figure S43. HR ESI LCMS of the mixture of Diacarnusin D (**4**) and the transformation products **4a** and **4b**
65	Table S7. NMR data of Diacarnusin D (**4**) in DMSO-d_6[a]
66	Table S8a. NMR data of Diacarnusin D major product (**4a**) in DMSO-d_6[a]
67	Table S8b. NMR data of Diacarnusin D minor product (**4b**) in DMSO-d_6[a]
68	Figure S44. ^{1}H NMR (500 MHz, $CDCl_3$) spectrum of the mixture of Diacarnusins E (**5**) and F (**6**)
69	Figure S44. ^{13}C NMR (125 MHz, $CDCl_3$) spectrum of the mixture of Diacarnusins E (**5**) and F (**6**)
70	Figure S46. HSQC (500/125 MHz, $CDCl_3$) 2D spectrum of the mixture of Diacarnusins E (**5**) and F (**6**)
71	Figure S46. HMBC (500/125 MHz, $CDCl_3$) 2D spectrum of the mixture of Diacarnusins E (**5**) and F (**6**)
72	Figure S48. COSY (500 MHz, $CDCl_3$) 2D spectrum of the mixture of Diacarnusins E (**5**) and F (**6**)

73	Figure S49. HR ESI MS of the mixture of Diacarnusins E (**5**) and F (**6**)
74	Table S9. NMR data of diacarnusins E (**5**) and F (**6**) in $CDCl_3$[a]
75	Figure S50. ^{1}H NMR (500 MHz, $CDCl_3$) spectrum of Sigmosceptrellin B (**7**)
76	Figure S51. ^{1}H NMR (500 MHz, DMSO-d_6) spectrum of Sigmosceptrellin B (**7**)
77	Figure S52. ^{13}C NMR (500 MHz, $CDCl_3$) spectrum of Sigmosceptrellin B (**7**)
78	Figure S53. ^{13}C NMR (125 MHz, DMSO-d_6) spectrum of Sigmosceptrellin B (**7**)
79	Figure S54. HSQC (500/125 MHz, $CDCl_3$) 2D spectrum of Sigmosceptrellin B (**7**)
80	Figure S55. HSQC (500 MHz, DMSO-d_6) 2D spectrum of Sigmosceptrellin B (**7**)
81	Figure S56. HMBC (500/125 MHz, $CDCl_3$) 2D spectrum of Sigmosceptrellin B (**7**)
82	Figure S57. COSY (500 MHz, $CDCl_3$) 2D spectrum of Sigmosceptrellin B (**7**)
83	Figure S58. COSY (500 MHz, DMSO-d_6) 2D spectrum of Sigmosceptrellin B (**7**)
84	Figure S59. HR ESI MS of Sigmosceptrellin B (**7**)
85	Table S10a. NMR data of Sigmosceptrellin B (**7**) in $CDCl_3$[a]
87	Table S10b. NMR data of Sigmospterellin B (**7**) in DMSO-d_6[a]
89	Figure S60. ^{1}H NMR (500 MHz, $CDCl_3$) spectrum of Sigmosceptrellin B Methyl Ester (**8**)
90	Figure S61. ^{13}C NMR (125 MHz, $CDCl_3$) spectrum of Sigmosceptrellin B Methyl Ester (**8**)
91	Figure S62. HSQC (500/125 MHz, $CDCl_3$) 2D spectrum of Sigmosceptrellin B Methyl Ester (**8**
92	Figure S63. HMBC (500/125 MHz, $CDCl_3$) 2D spectrum of Sigmosceptrellin B Methyl Ester (**8**)

93	Figure S64. COSY (500 MHz, $CDCl_3$) 2D spectrum of Sigmosceptrellin B Methyl Ester (**8**)
94	Figure S65. HR ESI MS of Sigmosceptrellin B Methyl Ester (**8**)
95	Table S11. NMR data of Sigmosceptrellin B methyl ester (**8**) in $CDCl_3$[a]
97	Figure S66. ^1H NMR (500 MHz, DMSO-d_6) spectrum of Diacarperoxide A (**9**)
98	Figure S67. ^{13}C NMR (125 MHz, DMSO-d_6) spectrum of Diacarperoxide A (**9**)
99	Figure S68. HSQC (500/125 MHz, DMSO-d_6) 2D spectrum of Diacarperoxide A (**9**)
100	Figure S69. HMBC (500/125 MHz, DMSO-d_6) 2D spectrum of Diacarperoxide A (**9**)
101	Figure S70. COSY (500 MHz, DMSO-d_6) 2D spectrum of Diacarperoxide A (**9**)
102	Figure S71. HR ESI MS of Diacarperoxide A (**9**)
103	Table S12. NMR Data of Diacarperoxide A (**9**) in DMSO-d_6[a]
104	Figure S72. ^1H NMR data (500 MHz, $CDCl_3$) spectrum of Methyl Diacarnoate A (**10**)
105	Figure S73. ^{13}C NMR data (125 MHz, $CDCl_3$) spectrum of Methyl Diacarnoate A (**10**)
106	Figure S74. HSQC data (500/125 MHz, $CDCl_3$) 2D spectrum of Methyl Diacarnoate A (**10**)
107	Figure S75. COSY data (500 MHz, $CDCl_3$) 2D spectrum of Methyl Diacarnoate A (**10**)
108	Figure S76. HR ESI MS of Methyl Diacarnoate A (**10**)
109	Table S13. NMR data of Methyl Diacarnoate A (**10**) in $CDCl_3$[a]
110	Figure S77. ^1H NMR data (500 MHz, $CDCl_3$) spectrum of (-)-*ent*-Muqubilone (**11**)
111	Figure S78. ^{13}C NMR data (125 MHz, $CDCl_3$) spectrum of (-)-*ent*-Muqubilone (**11**)

112	Figure S79. HSQC data (500/125 MHz, $CDCl_3$) 2D spectrum of (-)-*ent*-Muqubilone (**11**)
113	Figure S80. HMBC data (500/125 MHz, $CDCl_3$) 2D spectrum of (-)-*ent*-Muqubilone (**11**)
114	Figure S81. COSY data (500 MHz, $CDCl_3$) 2D spectrum of (-)-*ent*-Muqubilone (**11**)
115	Figure S82. HR ESI MS of (-)-*ent*-Muqubilone (**11**)
116	Table S14. NMR Data data of (-)-*ent*-Muqubilone (**11**) in $CDCl_3$[a]
117	Figure S83. Concentration-response cytotoxicity curves of Diacarnusin A (**1**) on the different cell lines
118	Figure S84. Concentration-response cytotoxicity curves of Methyl Diacarnoate A (**10**) on the different cell lines
119	Figure S85. Concentration-response cytotoxicity curves of Diacarnusins E&F (**5&6**) on the different cell lines
120	Figure S86. Concentration-response cytotoxicity curves of Sigmosceptrellin B (**7**) on the different cell lines
121	Figure S87. Concentration-response cytotoxicity curves of Sigmosceptrellin B methyl ester (**8**) on the different cell lines
122	Figure S88. Concentration-response cytotoxicity curves of Diacarperoxide A (**9**) on the different cell lines
123	Figure S89. Concentration-response cytotoxicity curves of Methyl Diacarnoate A (**10**) on the different cell lines
124	Figure S90. Concentration-response cytotoxicity curves of (-)-*ent*-Muqubilone (**11**) on the different cell lines

Figure S1. Photographs of *D. erythreanus* in situ

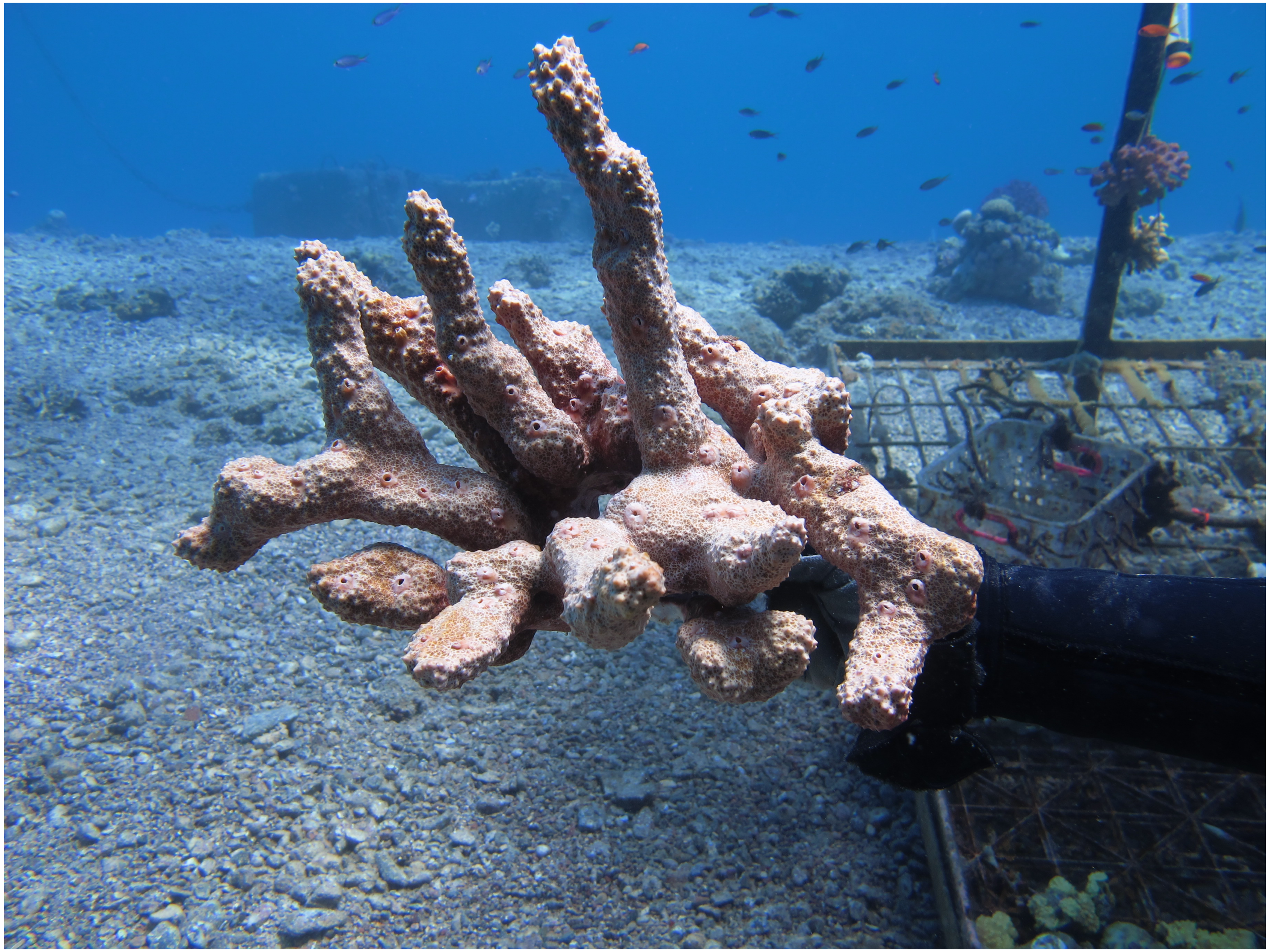

Figure S2. *nor*-Diterpenes and *nor*-sesterterpene endoperoxides isolated from extracts of Red Sea *D. erythreanus* samples

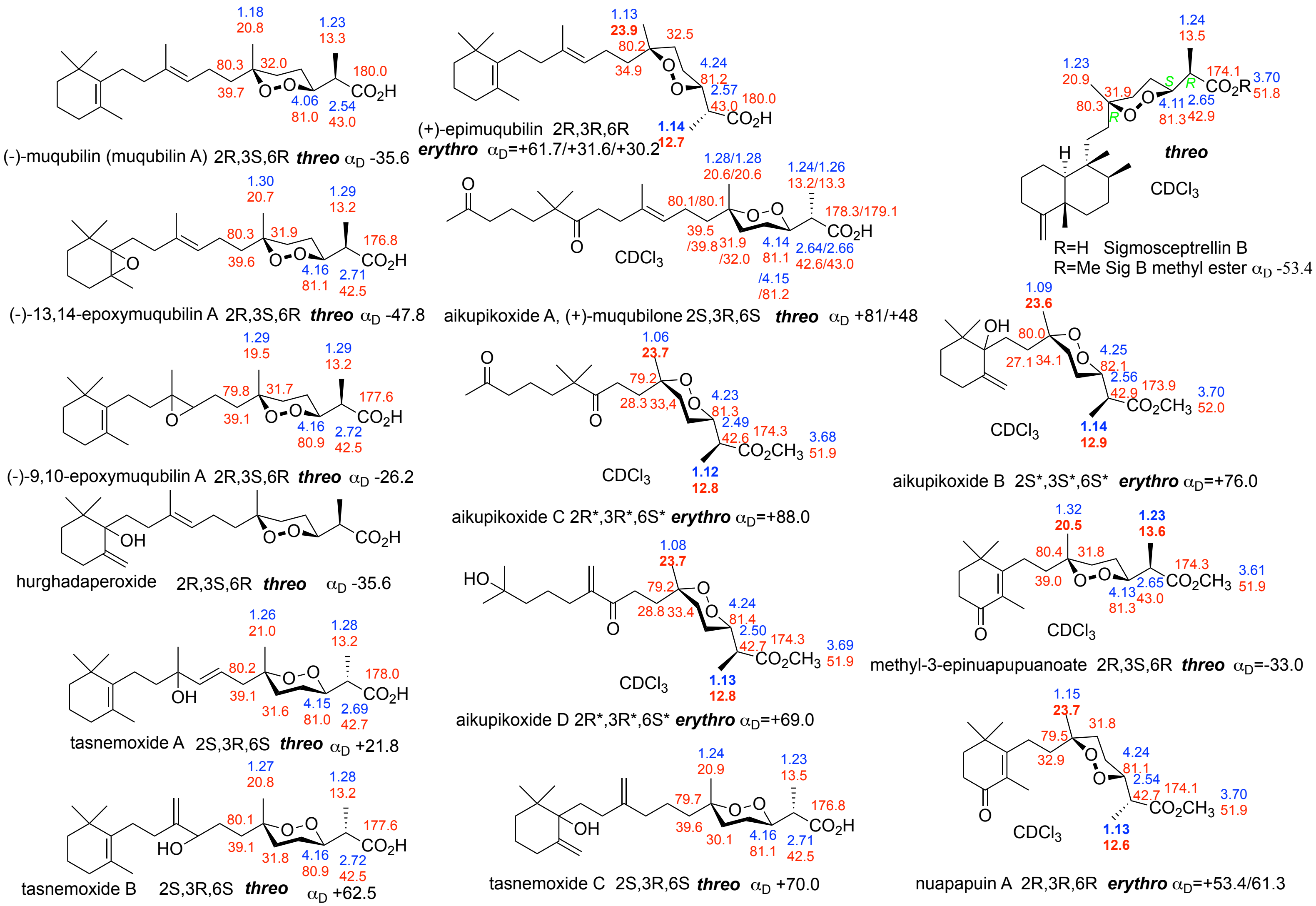

The first sample of *D. erythraeanus* was collected at Marsa el Muqubila, the Sainai Peninsula, Gulf of Aqaba in the end of the 1970's and yielded muqubilin.[1] A second sample was collected in Eilat, Israel at the northern Gulf of Aqaba in the beginning of 1980's and yielded (although given the names prianicins A and B) muqubilin and sigmosceptrellin B and their antimicrobial activity was characterized.[7] The structure elucidation of hurghaperoxide from a sample of *D. erythraeanus* that was collected in Hurghada, the Egyptian Red Sea coast, was published on 1996.[8] El Sayed *et al.* described the structure elucidation and biological activity of the new muqubilone and the known muqubilin and sigmosceptrellin B from a sample of the *D. erythraeanus* collected northeast of Hurghada, Egypt, on 1997.[9] Muqubilone (aikupikoxide A), aikupikoxides B-D, and the known (+)-epimuqubilin and nuapapuin A were isolated from a sample of *D. erythraeanus* collected on 1999 at El Quster, 120 km south of Hurghada, Egypt.[10] Tasnemoxides A-C and the known sigmosceptrellin B were isolated from a sample of *D. erythraeanus* collected off Hurghada, Egypt on 2003.[11] Additional sample of *D. erythraeanus* that was collected in Elfanadir, near Hurghada, Egypt on 2009 yielding two new epoxy-derivatives of muqubilin, (-)-13,14-epoxy- and (-)-9,10-epoxy-muqubilin A along with the known muqubilin, hurghaperoxide, sigmosceptrellin B and its methyl ester, nuapapuin A and methyl-3-epinupapuanoate, and their cytotoxicity was evaluated on a panel of cancer cell-lines.[12] The latest report on *D. erythraeanus* metabolites from the Red Sea comes from a 2011 collection of a sponge sampled at the Jordanian coast of the Gulf of Aqaba which yielded muqubilone, sigmosceptrellin B and its methyl ester, methyl-3-epinuapapuanote and its enantiomer (probably, nuapupuin A).[13]

Flow Diagram S1. Reversed phase separation of the crude extract of *D. erythreanus*

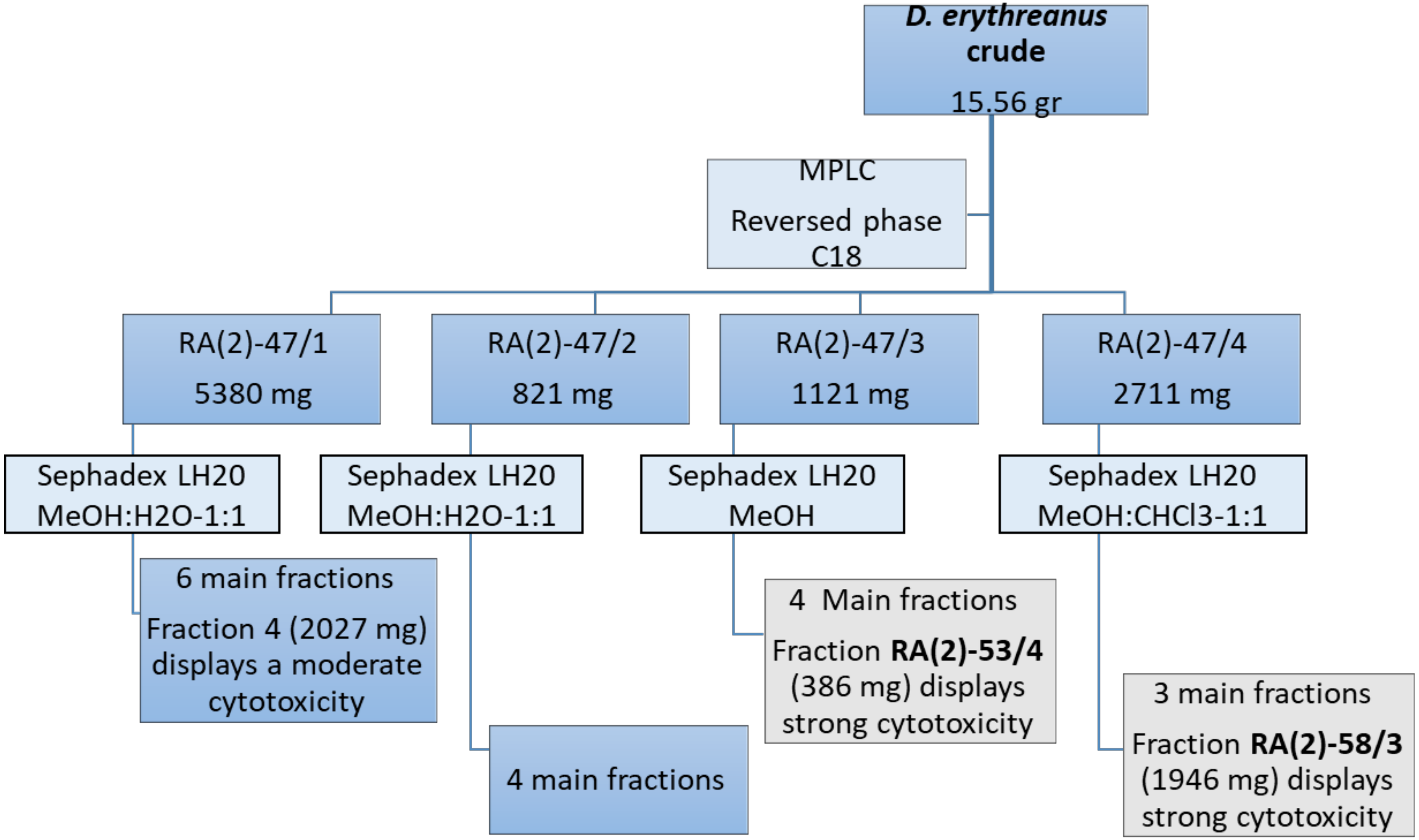

Flow Diagram S2. Reversed phase HPLC separation of the fraction RA(2)-53/4

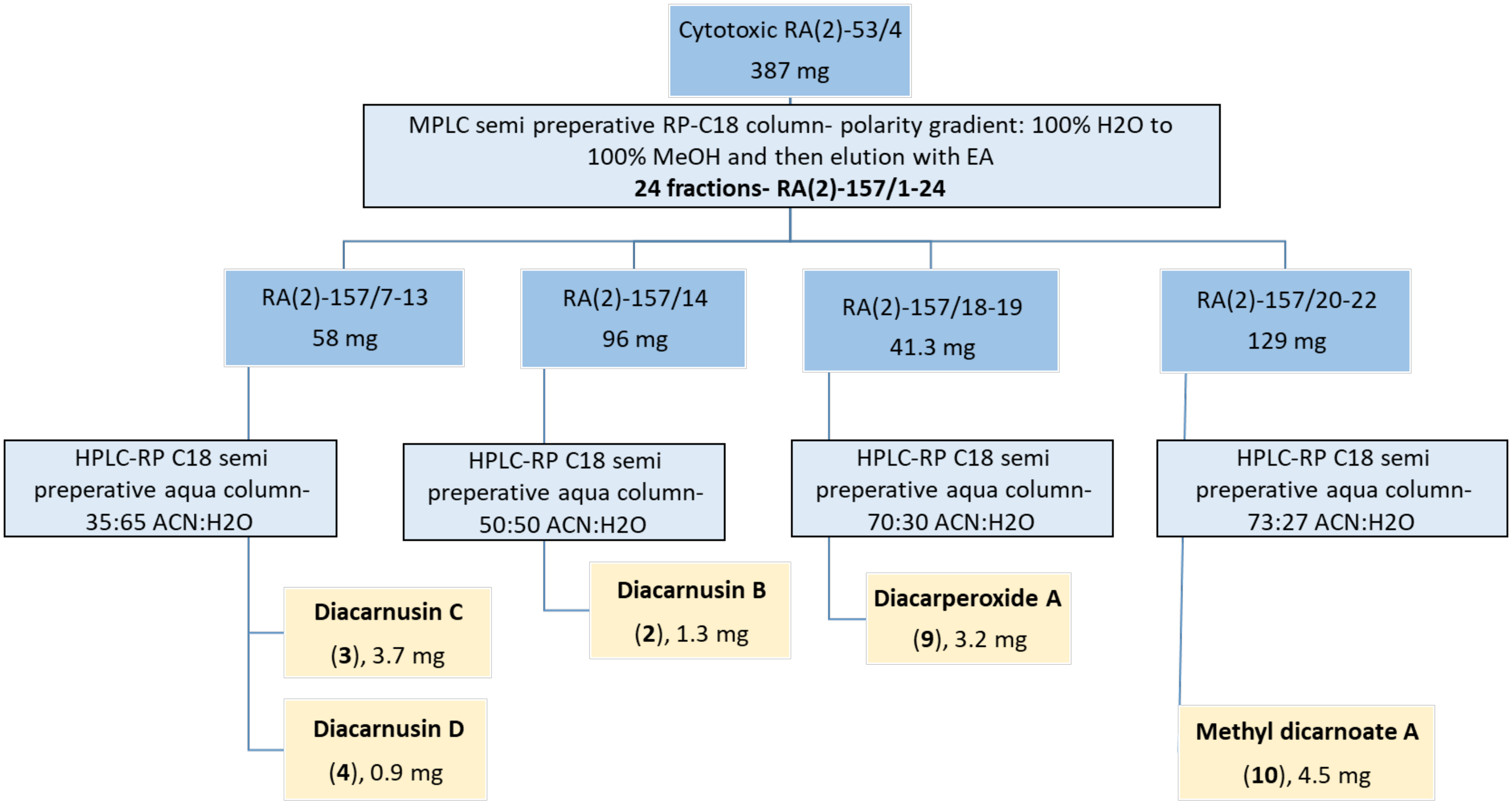

Flow Diagram S3. Reversed phase HPLC separation of the fraction RA(2)-58/3

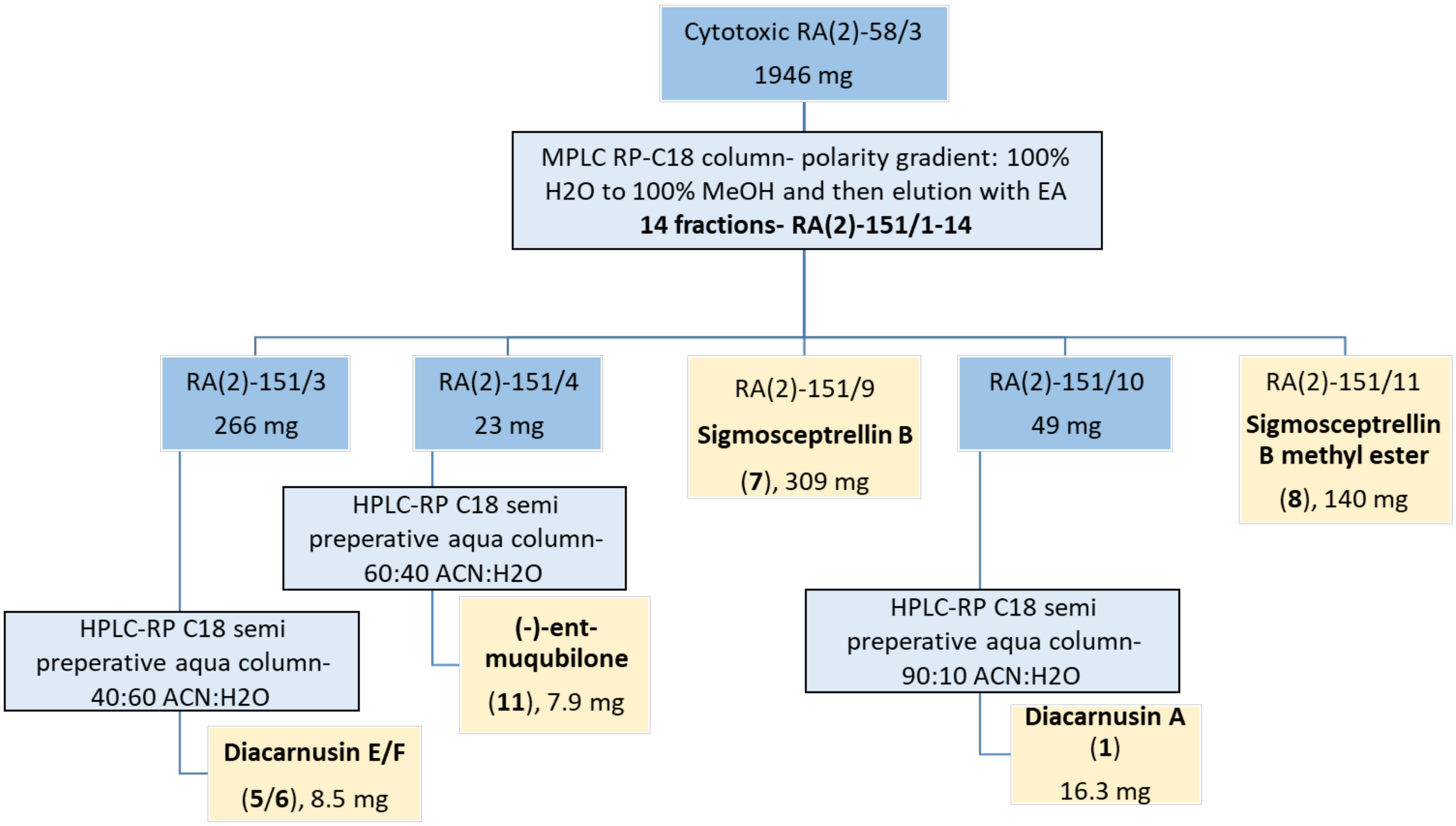

Figure S3. ^{1}H NMR data (500 MHz, $CDCl_3$) spectrum of Diacarnusin A (**1**)

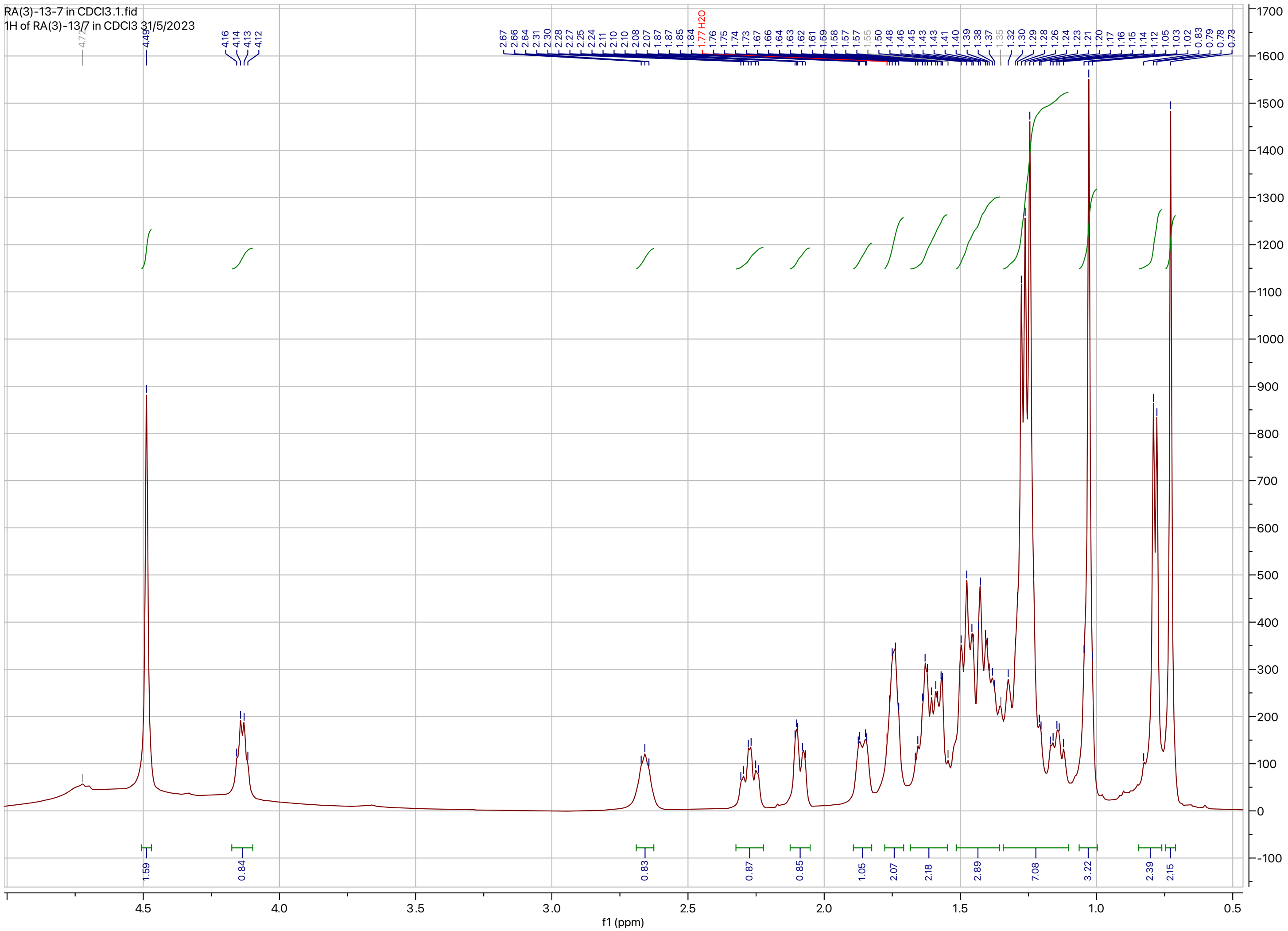

Figure S4. ^{13}C NMR data (125 MHz, $CDCl_3$) spectrum of Diacarnusin A (**1**)

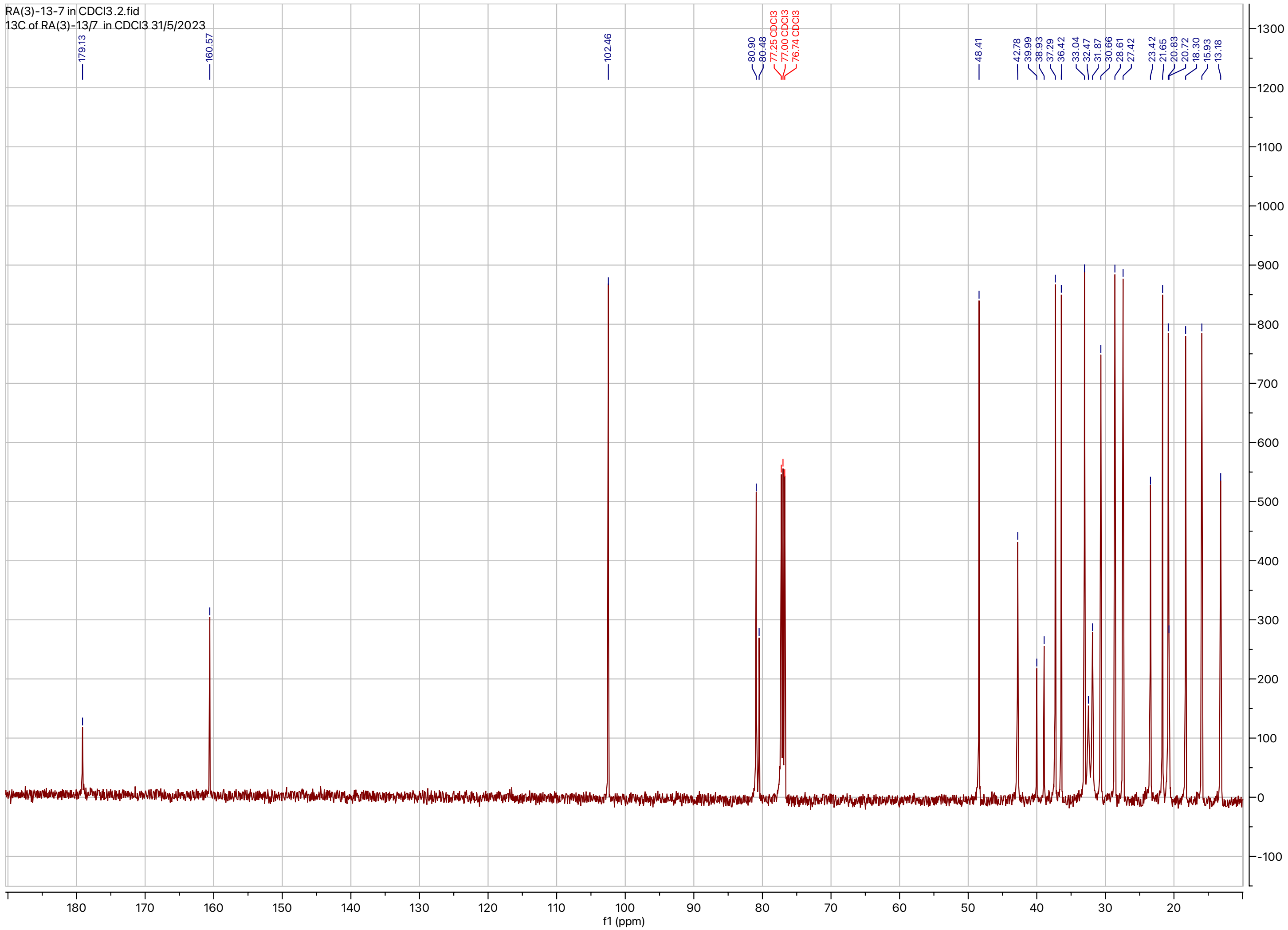

Figure S5. HSQC data (500/125 MHz, $CDCl_3$) 2D spectrum of Diacarnusin A (**1**)

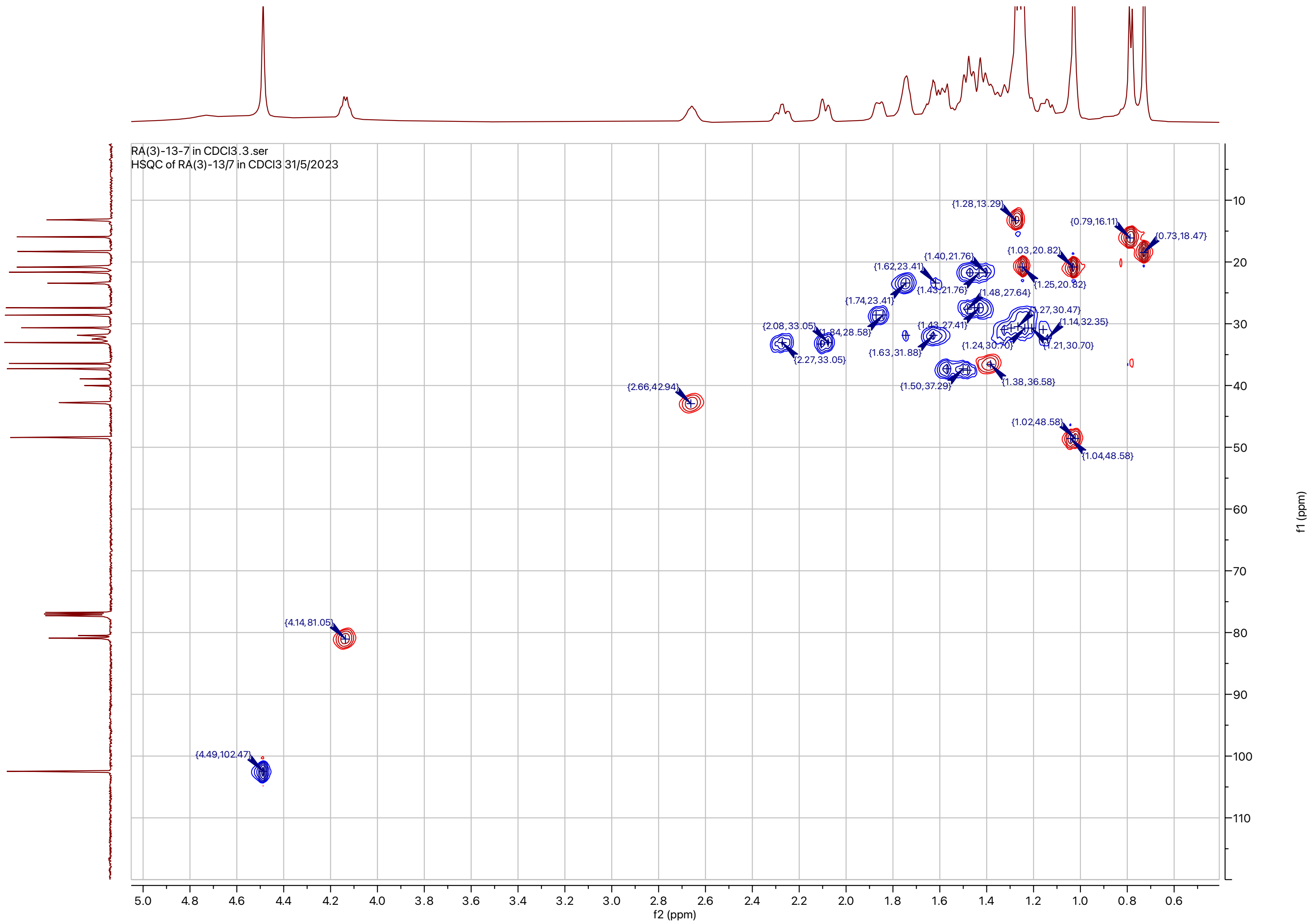

Figure S6. HMBC data (500/125 MHz, $CDCl_3$) 2D spectrum of Diacarnusin A (**1**)

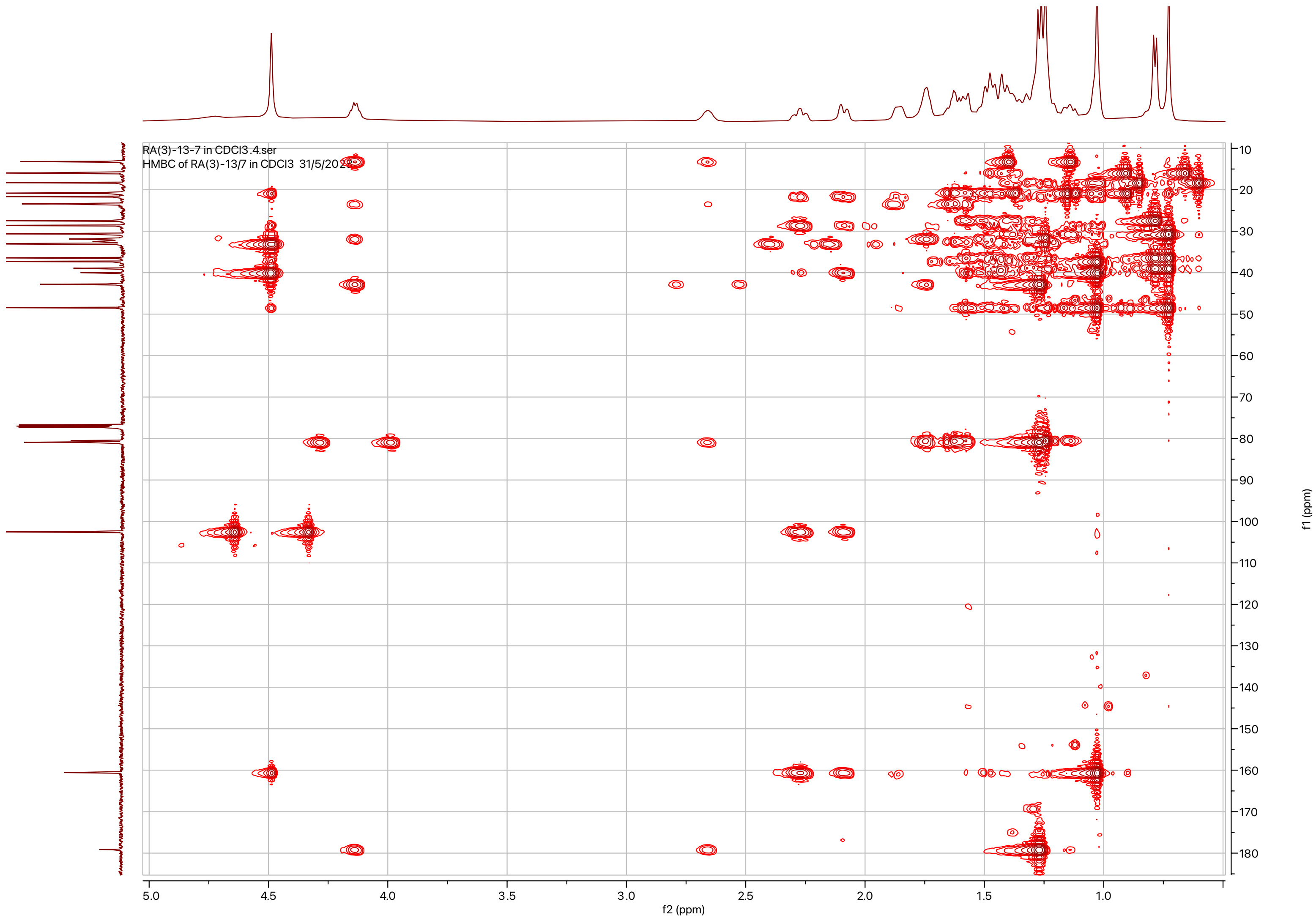

Figure S7. COSY data (500 MHz, $CDCl_3$) 2D spectrum of Diacarnusin A (**1**)

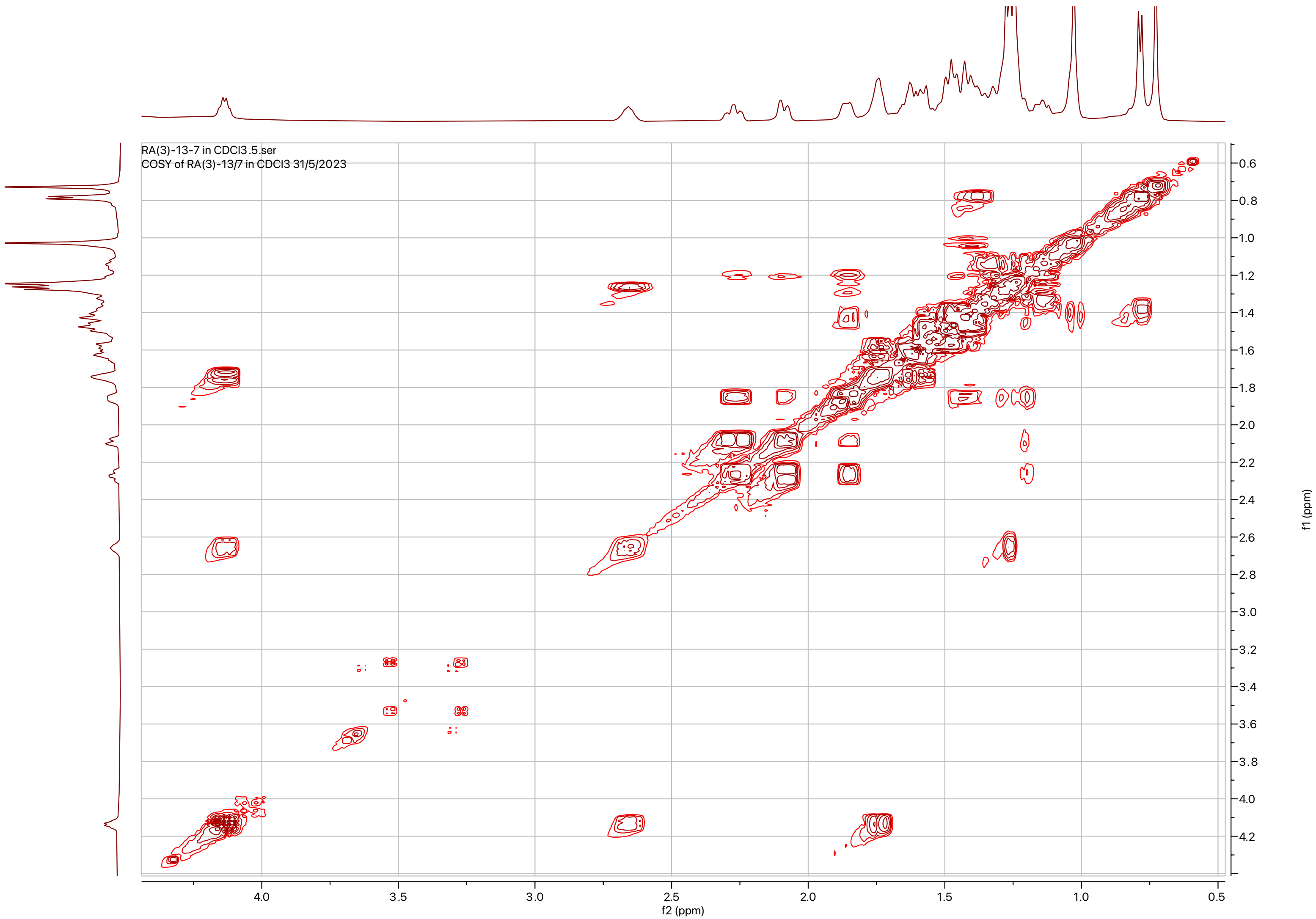

Figure S8. NOESY data (500 MHz, $CDCl_3$) 2D spectrum of Diacarnusin A (**1**)

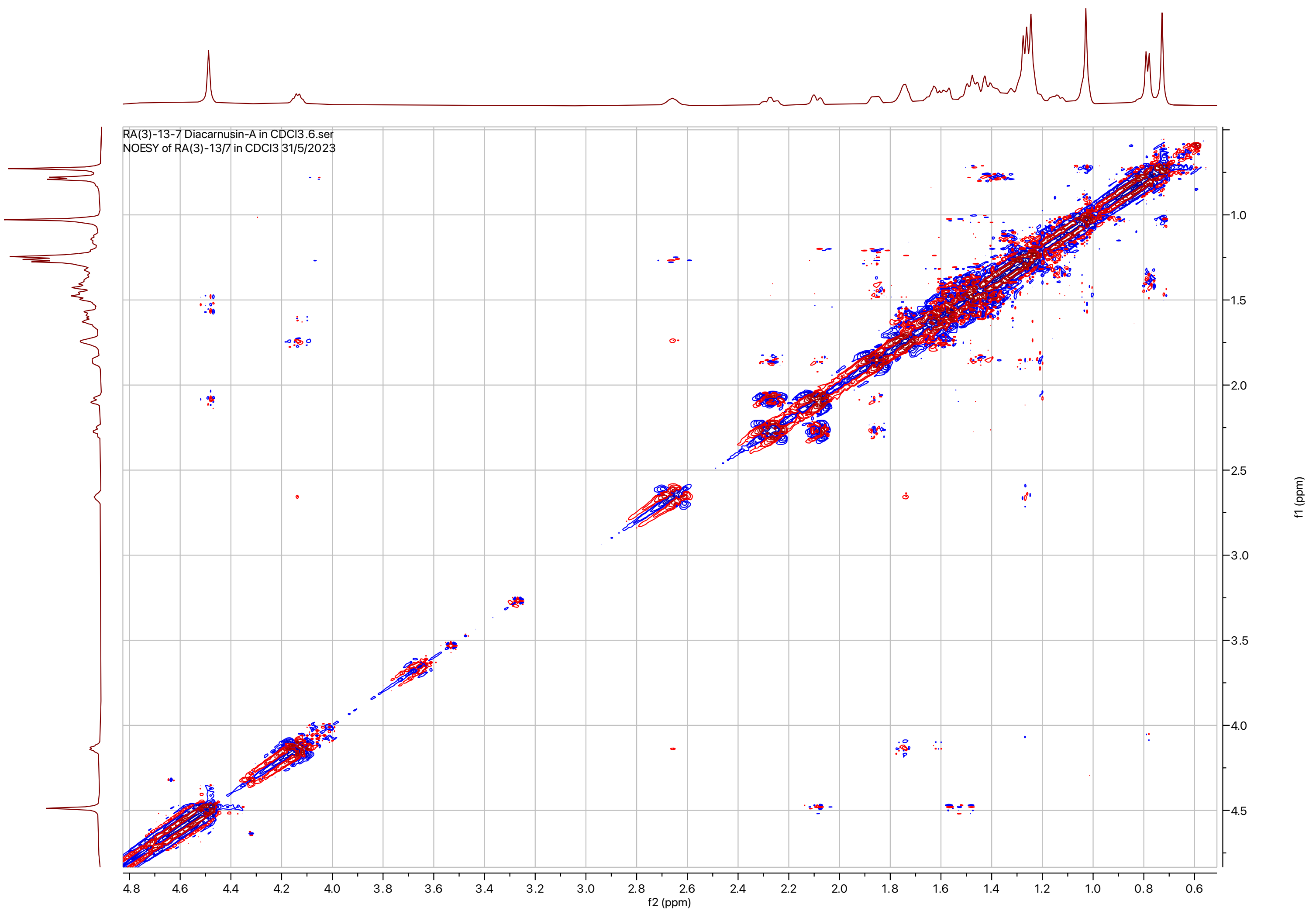

Figure S9. HR ESI MS of Diacarnusin A (**1**)

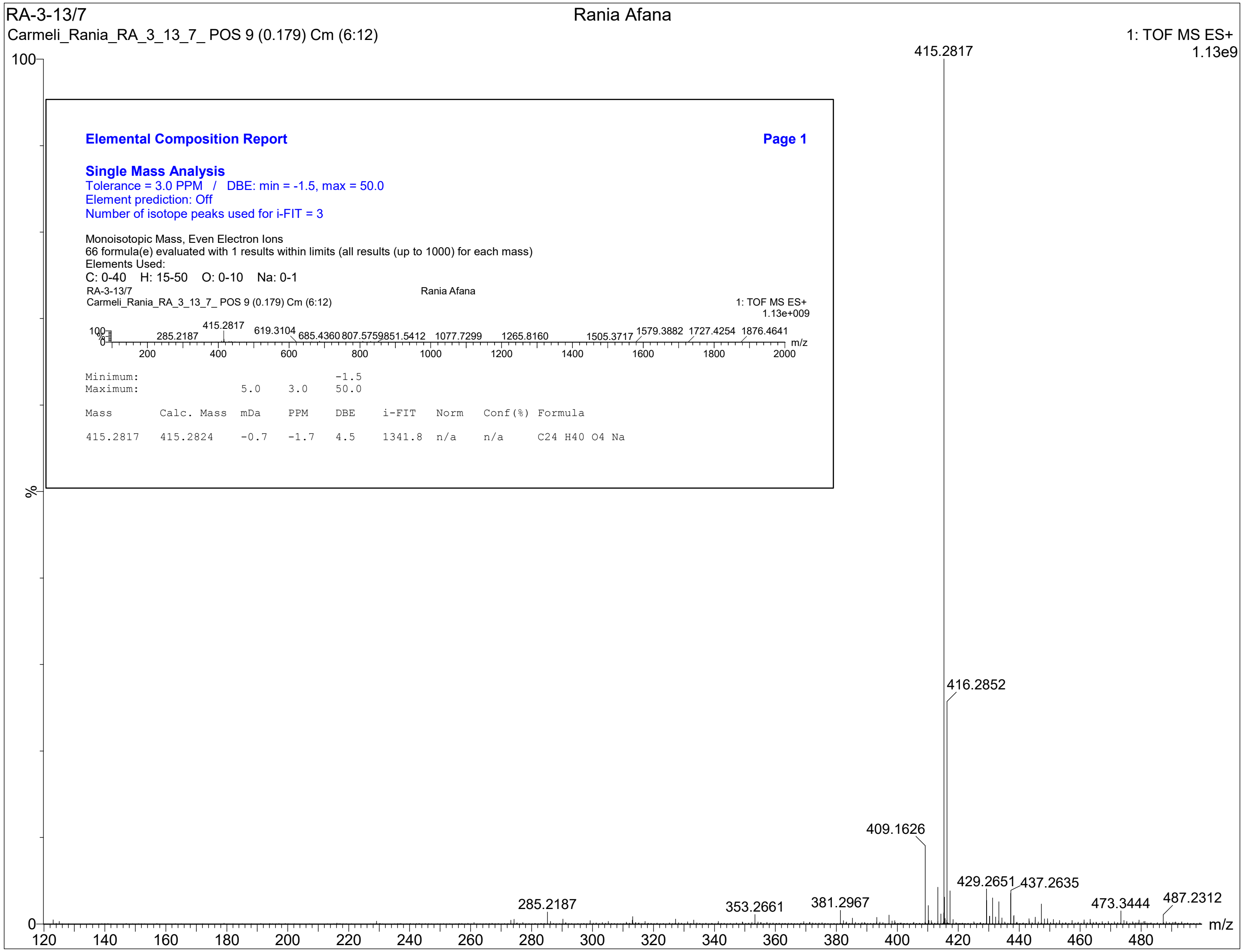

Minimum:				-1.5					
Maximum:		5.0	3.0	50.0					
Mass	Calc. Mass	mDa	PPM	DBE	i-FIT	Norm	Conf(%)	Formula	
415.2817	415.2824	-0.7	-1.7	4.5	1341.8	n/a	n/a	C24 H40 O4 Na	

Table S1. NMR Data of Diacarnusin A (**1**) in $CDCl_3$[a]

Position	δ_C, type[b]	δ_H, multiplicity, *J* in Hz	HMBC correlations[c]	COSY correlations	ROESY correlations
1	179.1, C		H-2,3, H3-19		
2	42.8, CH	2.66, dq (6.7, 6.8)	H-3, H2-4, H3-19	H-3, H3-19	
3	80.9, CH	4.14, brdt (6.4, 6.7)	H-2, H2-4,5, H3-19	H-2, H2-4	H2-4, H3-19
4	23.4, CH2	1.74, m	H-2,3, H2-5	H-3, H2-5	
5	31.9, CH2	1.62, m	H-3, H2-4, H3-20	H2-4	
6	80.5, C		H2-4,5,7, H3-20		
7	32.5, CH2	1.34, m	H2-5, H-8		
		1.14, m			
8	30.7, CH2	1.30, m	H-7',10, H3-21	H-7',8'	
		1.16, m			
9	38.9, C		H-8, H3-21,22		
10	36.4, CH	1.39, m	H-12, H2-11, H3-21,22	H2-11, H3-22	H3-22
11	27.4, CH2	1.48, m	H2-12, H3-22	H-10, H2-12	
		1.43, m		H-10, H2-12	
12	37.3, CH2	1.58, m	H-11',18	H-12', H2-11	
		1.49, m		H-12'	
13	40.0, C		H-18, H2-11,12,15, 24		
14	160.6, C		H-11,18, H2-15,24		
15	33.0, CH2	2.27, ddd (4.3, 12.8, 13.7)	H-16', H2-17,24	H2-24, H-15', 16, 18	H-15',16,17
		2.09, brd (12.8)		H-15,16	H2-24, H-15,16,17
16	28.6, CH2	1.86, brd (10.5)	H2-15,17,24	H2-15, H-16',17	H2-15,17, H-16'
		1.24, m		H-16	H-16
17	21.7, CH2	1.47, m	H2-15, H-18		H-16
		1.42, m			H-16
18	48.4, CH	1.03, m	H2-24. H2-12, H-17',16',18, H3-21	H2-17	H3-21
19	13.2, CH3	1.27, d (6.8)	H-2,3	H-2	
20	20.7, CH3	1.25, s	H2-5, H-7'		
21	18.3, CH3	0.73, s	H-7',8,10, H3-23		H3-23
22	15.9, CH3	0.78, d (6.5)	H2-11	H-10	
23	20.8, CH3	1.03, s	H-18, H2-12,24,		H3-21

24	102.5, CH2	4.49, s	H2-15	H-15	H-15’, H2-12

[a]500 MHz for ^{1}H, 125 MHz for ^{13}C; [b]From HSQC; [c]HMBC correlations, optimized for J_{C-H}=8 Hz, are from carbon to the indicated proton(s).

Table S2. Comparison of the NMR Data of Diacarnusin A (**1**) and Sigmosceptrellin B (**7**) in $CDCl_3$[a]

	Diacarnusin A (**1**)		Sigmosceptrellin B (**7**)	
Position	δ_C, multiplicity	δ_H, multiplicity, *J* in Hz	δ_C, multiplicity	δ_H, multiplicity
1	179.1, C		174.8, C	
2	42.8, CH	2.66, dq (6.7, 6.8)	42.4, CH	2.47, brt
3	80.9, CH	4.14, brdt (6.4, 6.7)	81.1, CH	3.99, dt
4	23.4, CH_2	1.74, m	23.1, CH_2	1.66, m
				1.62, m
5	31.9, CH_2	1.62, m	31.7, CH_2	1.61, m
				1.49, m
6	80.5, C		79.7, C	
7	32.5, CH_2	1.34, m	32.2, CH_2	1.24, m
		1.14, m		1.04, m
8	30.7, CH_2	1.30, m	30.4, CH_2	1.24, m
		1.16, m		1.18, m
9	38.9, C		38.6, C	
10	36.4, CH	1.39, m	36.1, CH	1.34, m
11	27.4, CH_2	1.48, m	27.2, CH_2	1.44, m
		1.43, m		1.39, m

12	37.3, CH_2	1.58, m	37.1, CH_2	1.50, m
		1.49, m		1.45, m
13	40.0, C		39.7, C	
14	160.6, C		159.7, C	
15	33.0, CH_2	2.27, ddd (4.3, 12.8, 13.7)	32.7, CH_2	2.22, dt
		2.09, brd (12.8)		2.04, brd
16	28.6, CH_2	1.86, brd (10.5)	28.3, CH_2	1.83, brd
		1.24, m		1.16, m
17	21.7, CH_2	1.47, m	21.3, CH_2	1.44, m
		1.42, m		1.39, m
18	48.4, CH	1.03, m	48.2, CH	0.95, brd
19	13.2, CH_3	1.27, d (6.8)	12.9, CH_3	1.06, d
20	20.7, CH_3	1.25, s	20.6, CH_3	1.15, s
21	18.3, CH_3	0.73, s	18.4, CH_3	0.70, s
22	15.9, CH_3	0.78, d (6.5)	16.0, CH_3	0.74, d
23	20.8, CH_3	1.03, s	20.7, CH_3	0.98, s
24	102.5, CH_2	4.49, s	103.0, CH_2	4.46, s

[a]500 MHz for ^{1}H, 125 MHz for ^{13}C.

Figure S10. ^{1}H NMR data (500 MHz, DMSO-d_6) spectrum of Diacarnusin B (**2**)

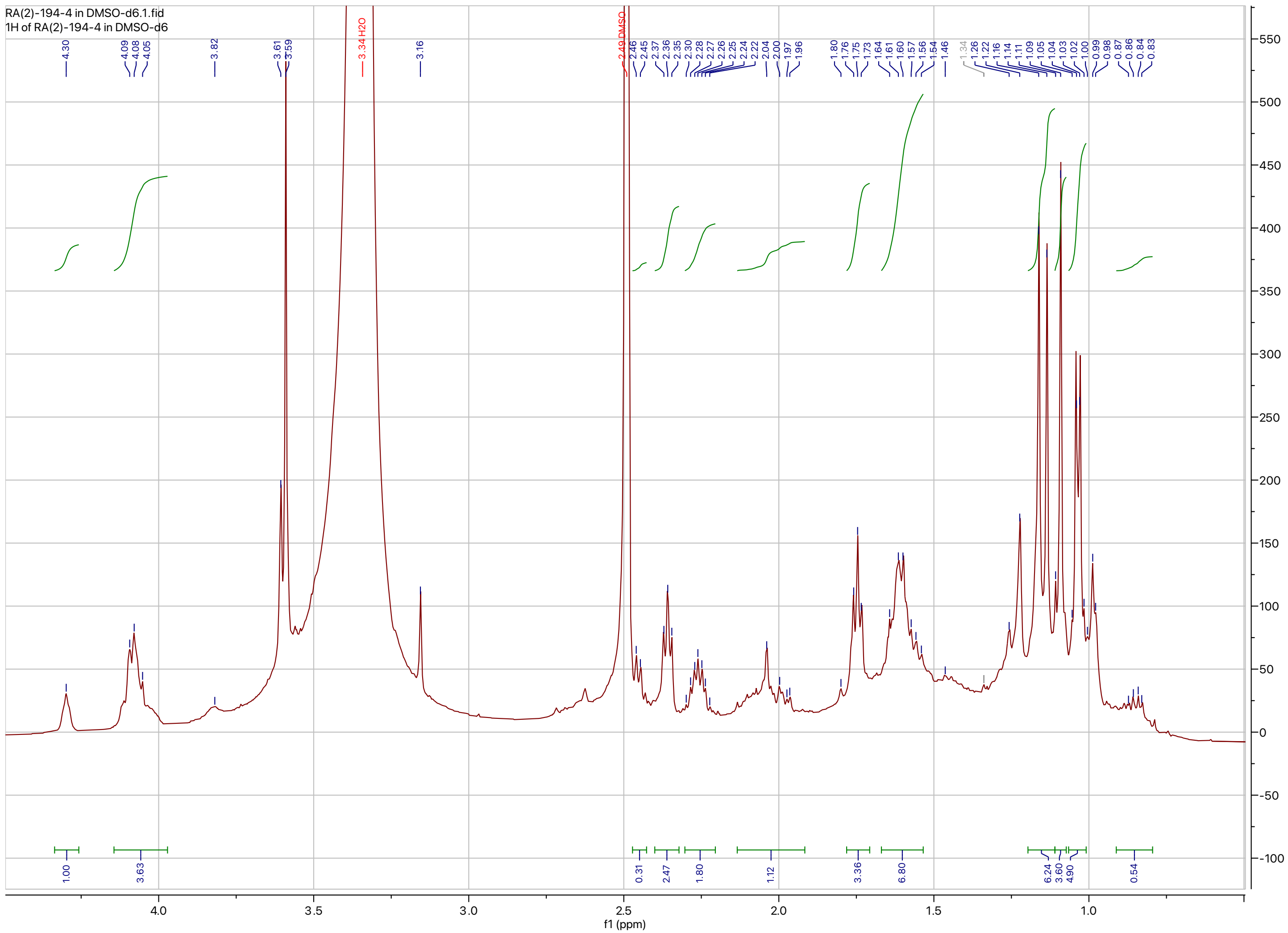

Figure S11. ^{13}C NMR (125 MHz, DMSO-d_6) spectrum of Diacarnusin B (**2**)

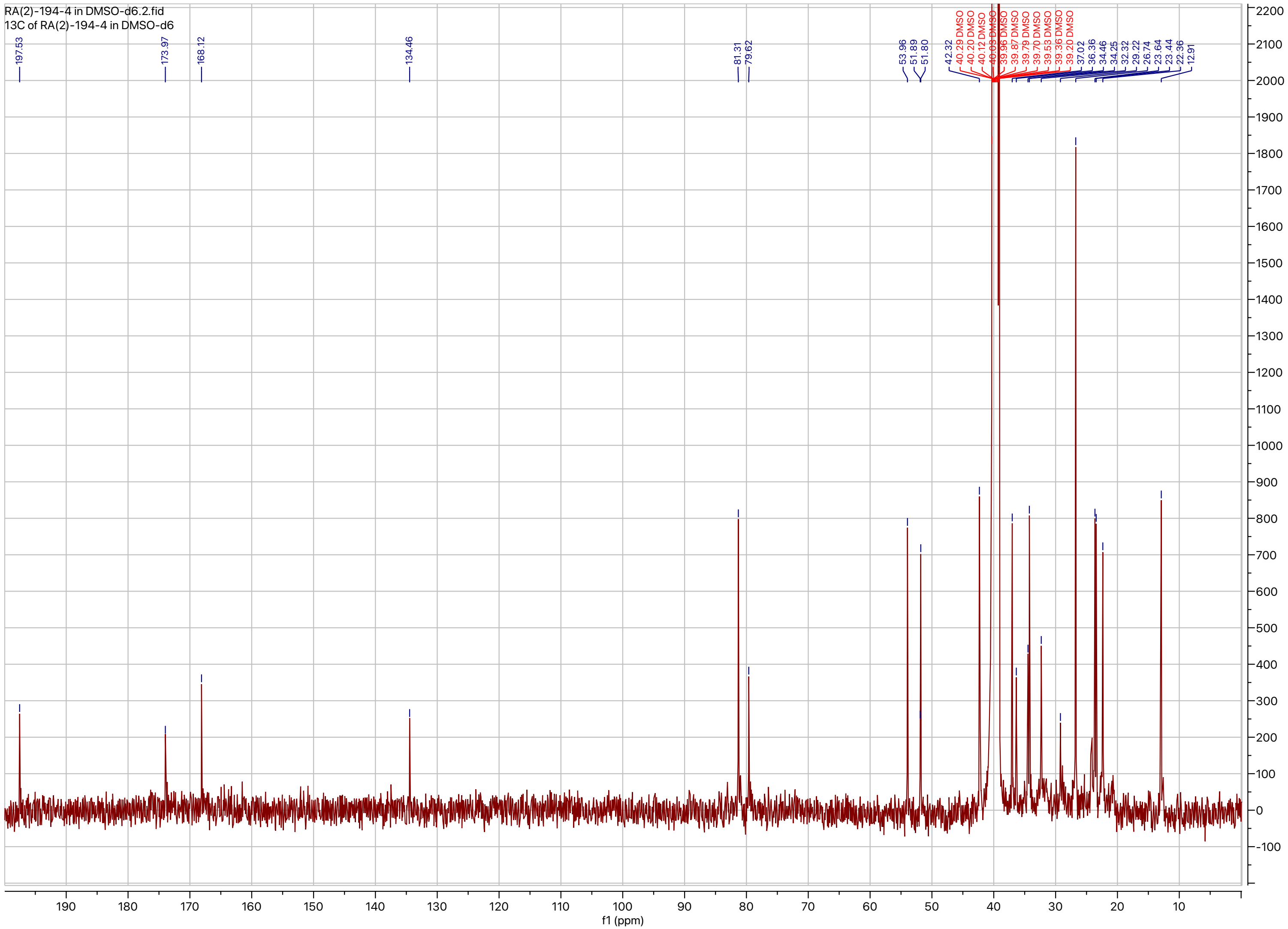

Figure S12. HSQC (500/125 MHz, DMSO-d_6) 2D spectrum of Diacarnusin B (**2**)

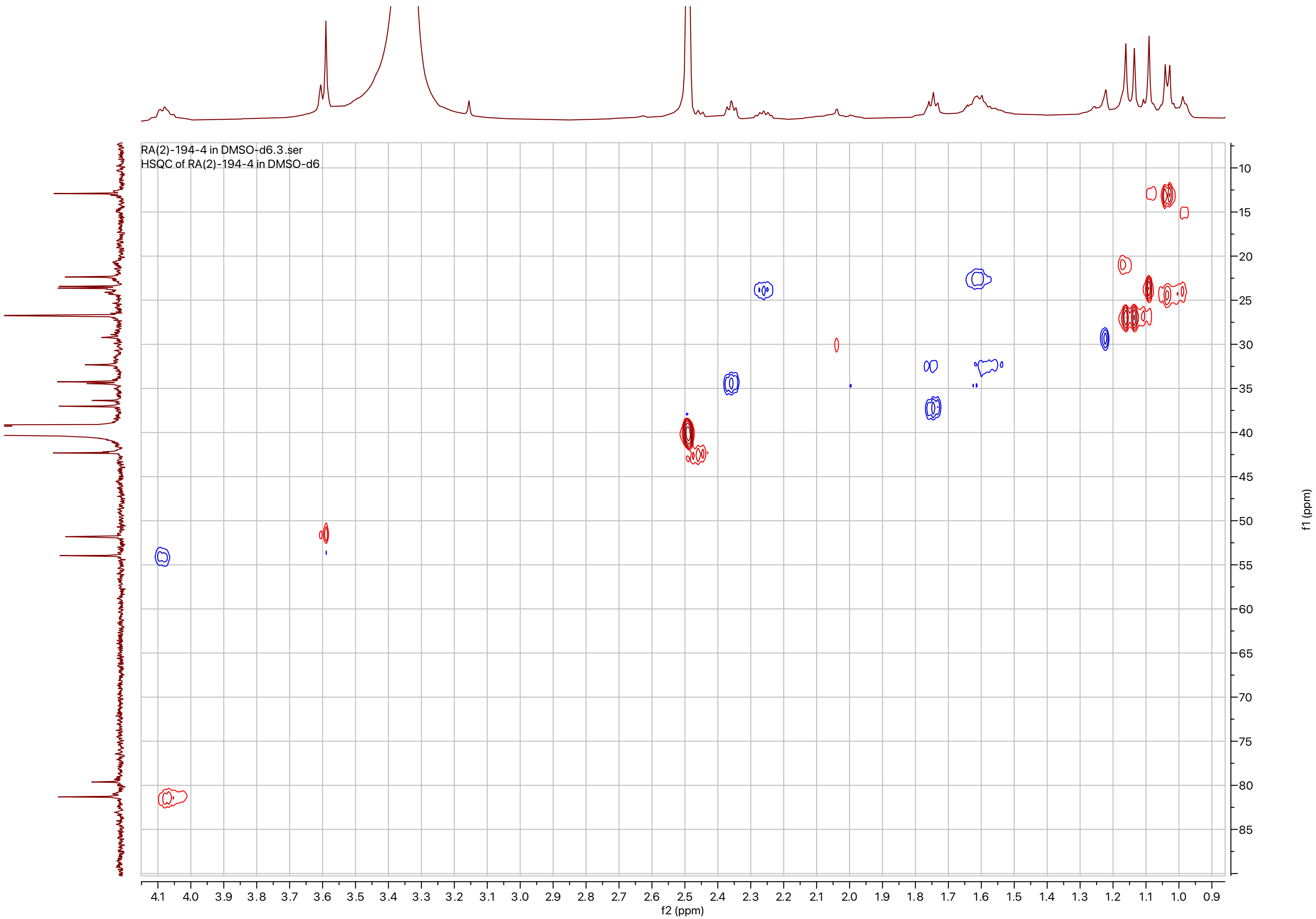

Figure S13. HMBC (500/125 MHz, DMSO-d_6) 2D spectrum of Diacarnusin B (**2**)

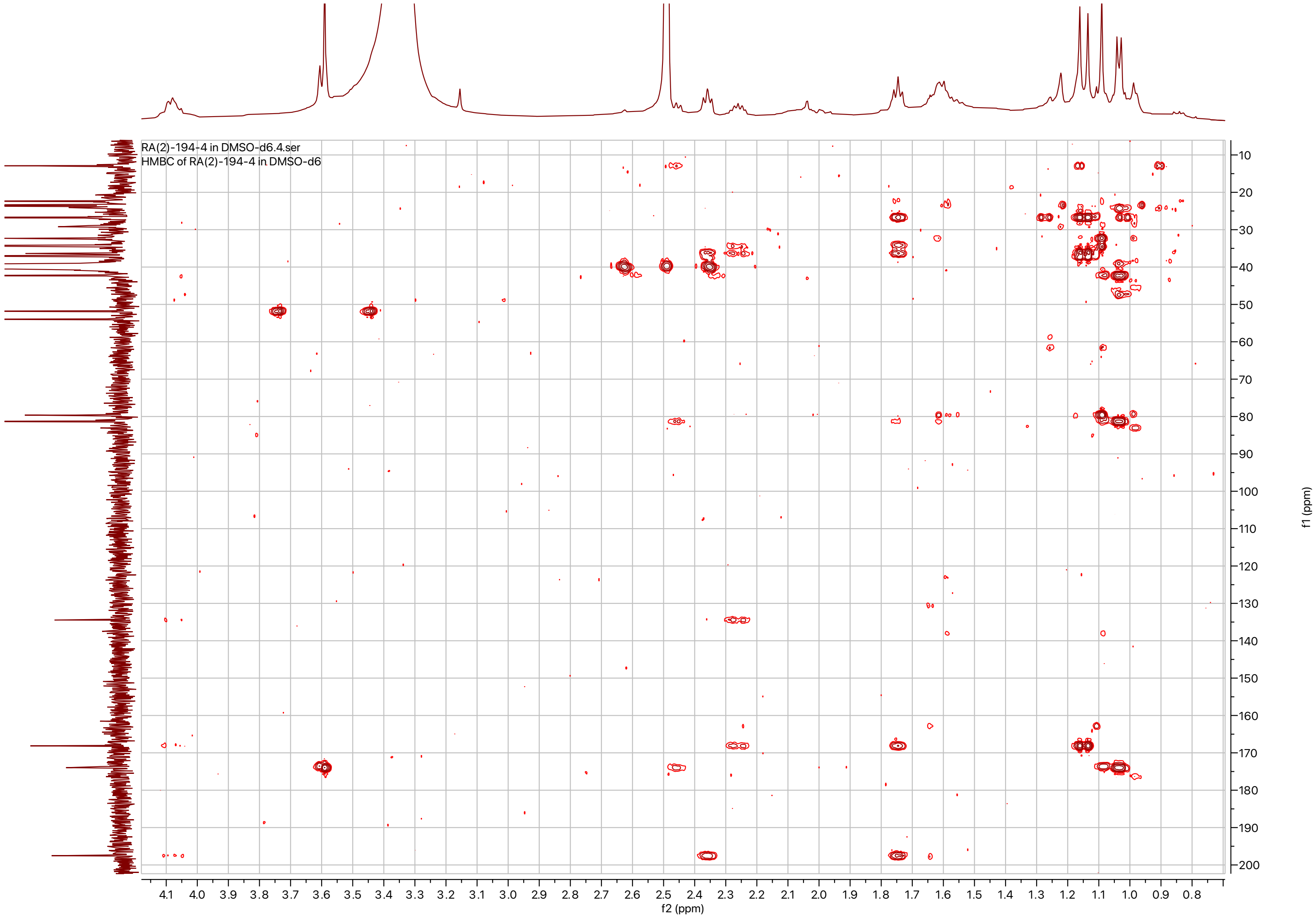

Figure S14. COSY (500 MHz, DMSO-d_6) 2D spectrum of Diacarnusin B (**2**)

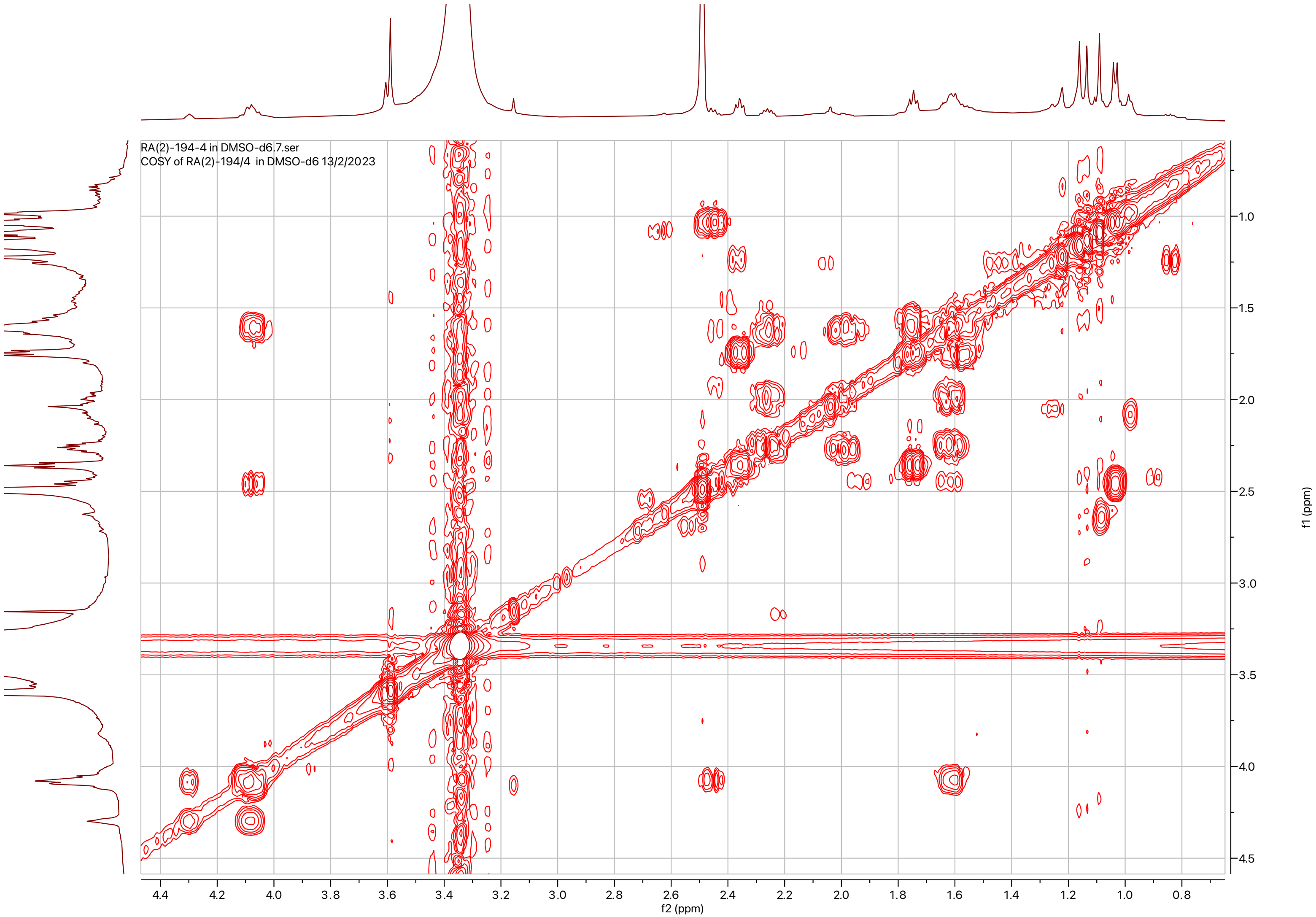

Figure S15. NOESY (500 MHz, DMSO-d_6) 2D spectrum of Diacarnusin B (**2**)

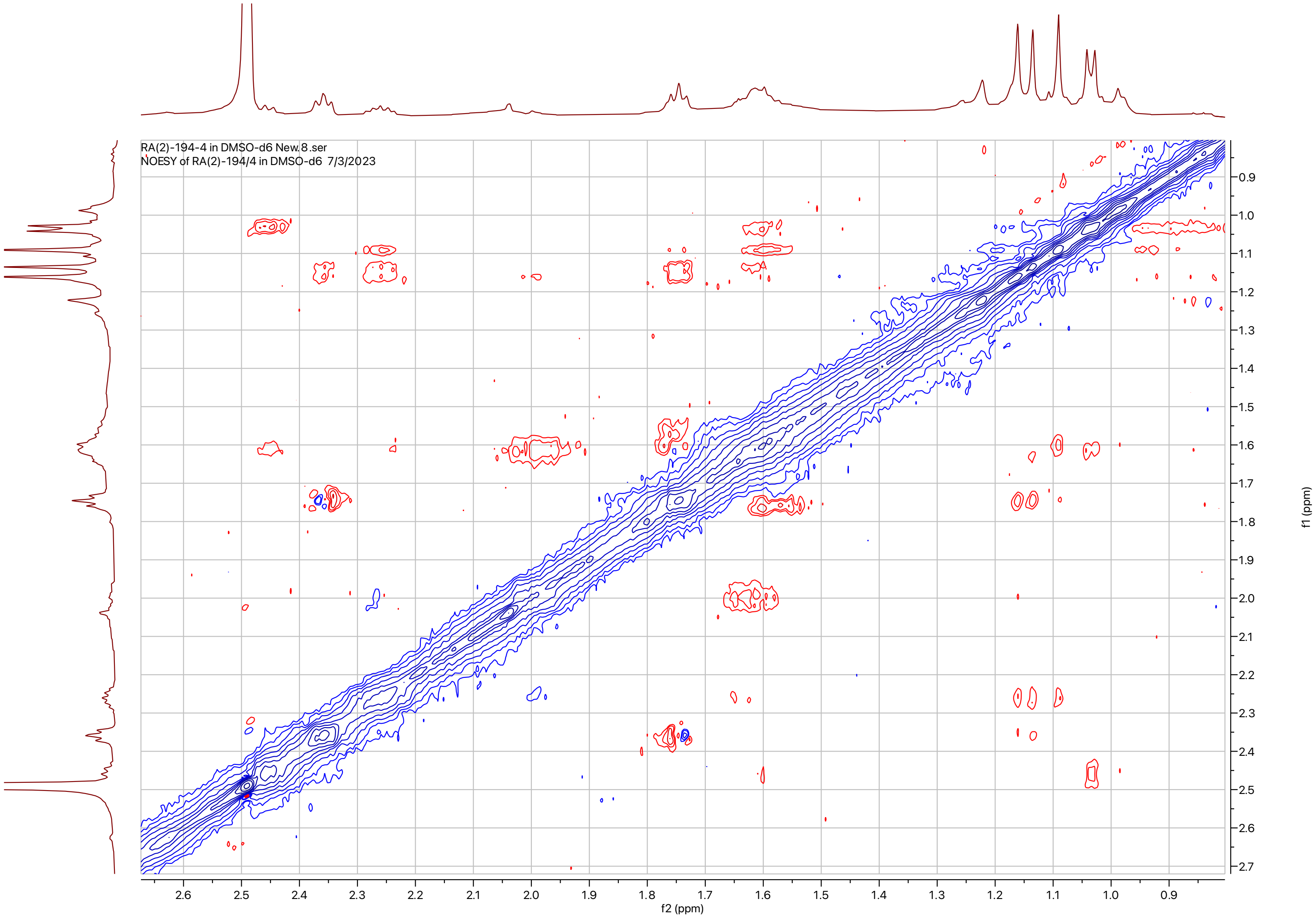

Figure S16. HR ESI MS of Diacarnusin B (**2**)

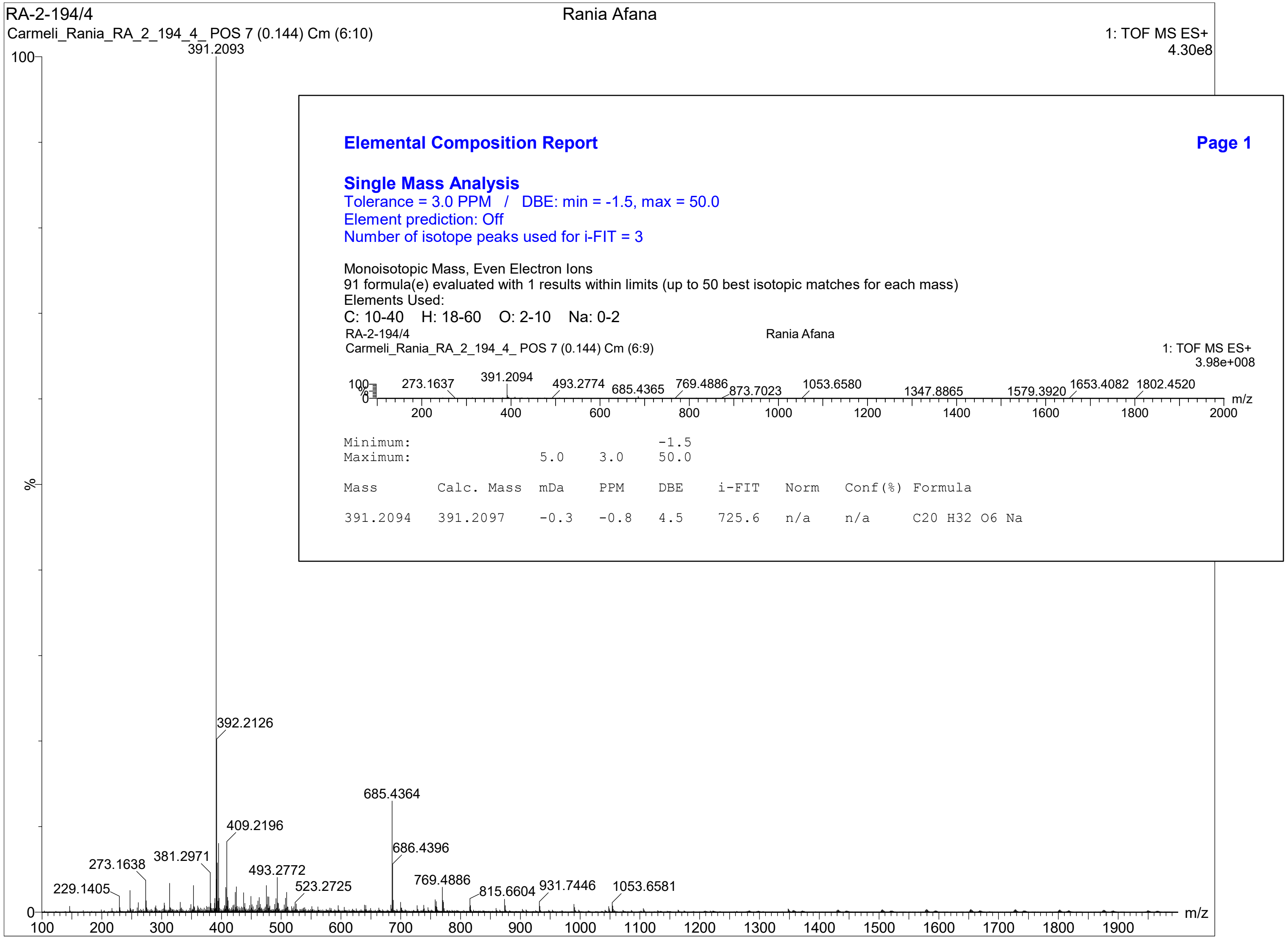

Table S3. NMR data of diacarnusin B (**2**) in DMSO-d_6[a]

Position	δ_C, type[b]	δ_H, multiplicity, J in Hz	HMBC correlations[c]	COSY correlations	ROESY correlations
1	174.0, C		H-2, H_3-15,20		
2	42.3, CH	2.45, brq (7.0)	H-3, H_3-15	H-3, H_2-4, H_3-15	H-3, H_2-4, H_3-15
3	81.3, CH	4.07, m	H-2, H_2-5, H_3-15	H-2, H_2-4	H-2, H_2-4, H_3-15
4	22.4, CH_2	1.62, m	H_2-5, H_3-16	H-3, H-2	H_3-15
		1.59, m		H-3, H-2	
5	32.3, CH_2	1.76, m	H-4, H_3-16	H-4',5'	H-4',5'
		1.61, m		H-4',5	H-4',5
6	79.6, C		H_2-5,7, H_3-16		
7	34.5, CH_2	2.00, m	H_2-8, H_3-16	H-7', H_2-8	H-7'
		1.60, m		H-7, H_2-8	H-7, H_3-16
8	23.6, CH_2	2.27, m	H_2-7	H-8', H_2-7	H_2-17, H_3-16,18,19
					H_2-17, H_3-16,18,19
		2.24, m		H-8, H_2-7	
9	168.1, C		H_2-8,13,17, H_3-18,19		
10	134.5, C		H_2-12,13,17		
11	197.5, C		H_2-12,13,17		
12	34.3, CH_2	2.36, t (6.9)	H_2-13	H_2-13	H_2-13
13	37.0, CH_2	1.75, t (6.9)	H_2-12, H_3-18,19	H_2-12	H_2-12, H_3-18,19
14	36.4, C		H_2-12,13,17, H_3-18,19		
15	12.9, CH_3	1.04, d (7.0)	H-2		H-3, H_2-4
16	23.4, CH_3	1.09, s	H-7'		H_2-8, H-7'
17	54.0, CH_2	4.10, m		17-OH	H_2-8
		4.06, m		17-OH	H_2-8
17-OH		4.30, brt (4.7)		H_2-8	
18	26.7, CH_3	1.14, s	H_2-13, H_3-19		H_2-8
19	26.7, CH_3	1.16, s	H_2-13, H_3-18		H_2-8,
20	51.8, CH_3	3.59, s			

[a]500 MHz for ^{1}H, 125 MHz for ^{13}C; [b]From HSQC; [c]HMBC correlations, optimized for $J_{C\text{-}H}$=8 Hz, are from carbon to the indicated proton(s).

Table S4. Comparison of the NMR data of Diacarnusin B (**2**) and Diacarperoxide A (**9**) in DMSO-d_6[a]

	Diacarnusin B (**2**)		Diacarperoxide A (**9**)	
Position	δ_C, multiplicity	δ_H, multiplicity, *J* in Hz	δ_C, multiplicity	δ_H, multiplicity, *J* in Hz
1	174.0, C		173.7, C	
2	42.3, CH	2.45, brq (7.0)	42.0, CH	2.69, dq, (7.0, 6.8)
3	81.3, CH	4.07, m	80.9, CH	4.05, m
4	22.4, CH_2	1.62, m	22.6, CH_2	1.66, m
		1.59, m		
5	32.3, CH_2	1.76, m	31.2, CH_2	1.69, m
		1.61, m		
6	79.6, C		79.9, C	
7	34.5, CH_2	2.00, m	37.4, CH_2	1.55, m
		1.60, m		1.49, m
8	23.6, CH_2	2.27, m	23.9, CH_2	2.19, t (8.4)
		2.24, m		
9	**168.1, C**		**164.9, C**	
10	**134.5, C**		**130.2, C**	
11	197.5, C		197.7, C	
12	34.3, CH_2	2.36, t (6.9)	34.0, CH_2	2.35, t (6.8)
13	37.0, CH_2	1.75, t (6.9)	37.0, CH_2	1.72, t (6.8)
14	36.4, C		36.4, C	
15	12.9, CH_3	1.04, d (7.0)	12.8, CH_3	1.10, d (6.8)
16	23.4, CH_3	1.09, s	20.5, CH_3	1.25, s
17	**54.0, CH_2**	**4.10, m**	**11.4, CH_3**	**1.64, s**
		**4.06, m**		
17-OH		**4.30, brt (4.7)**		
18	26.7, CH_3	1.14, s	26.7, CH_3	1.11, s
19	26.7, CH_3	1.16, s	26.8, CH_3	1.11, s
20	51.8, CH_3	3.59, s	51.9, CH_3	3.61, s

[a]500 MHz for ^{1}H, 125 MHz for ^{13}C.

Figure S17. ^{1}H NMR (500 MHz, $CDCl_3$) spectrum of Diacarnusin C (**3**)

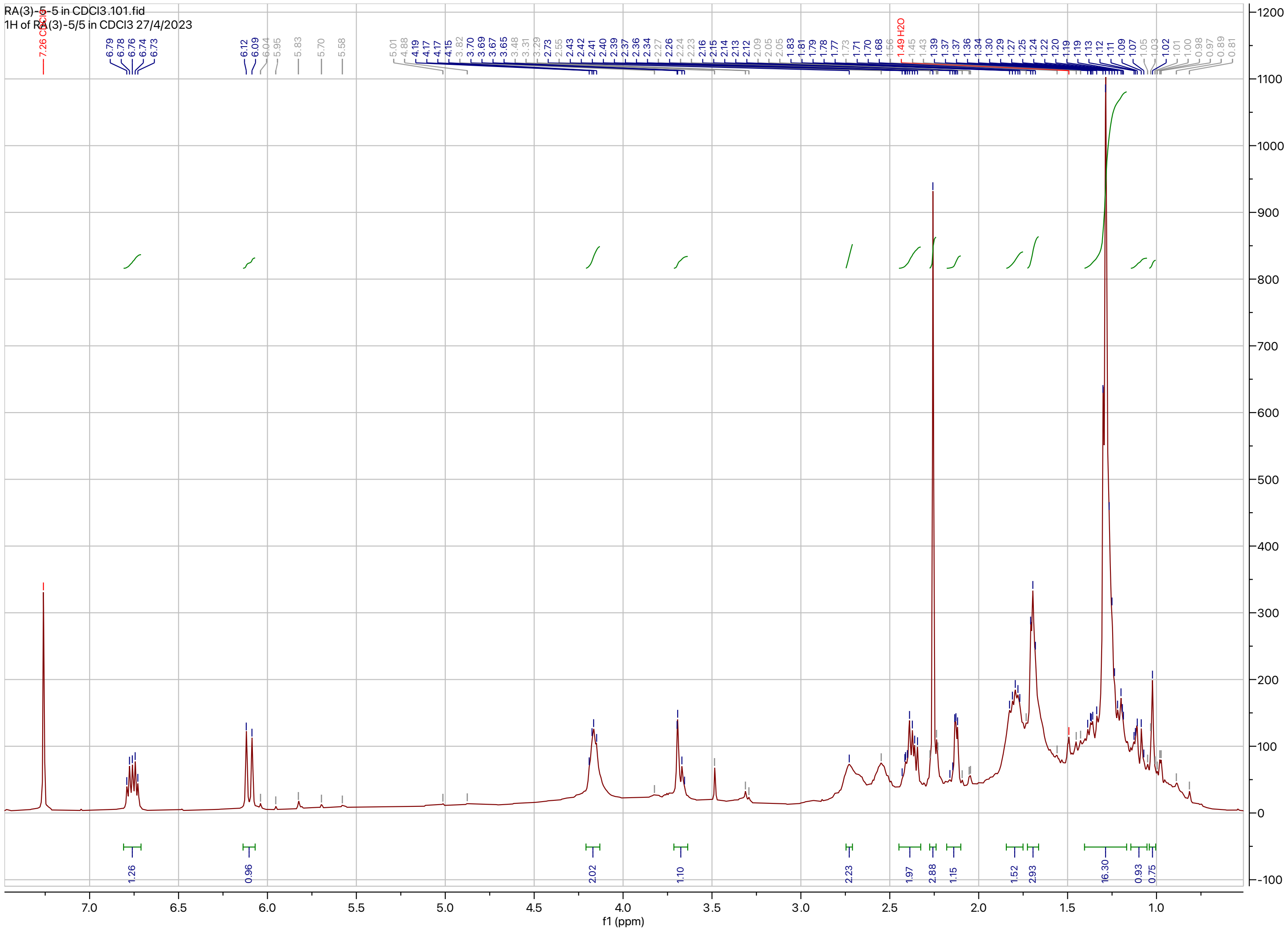

Figure S18. ^{13}C NMR (125 MHz, $CDCl_3$) spectrum of Diacarnusin C (**3**)

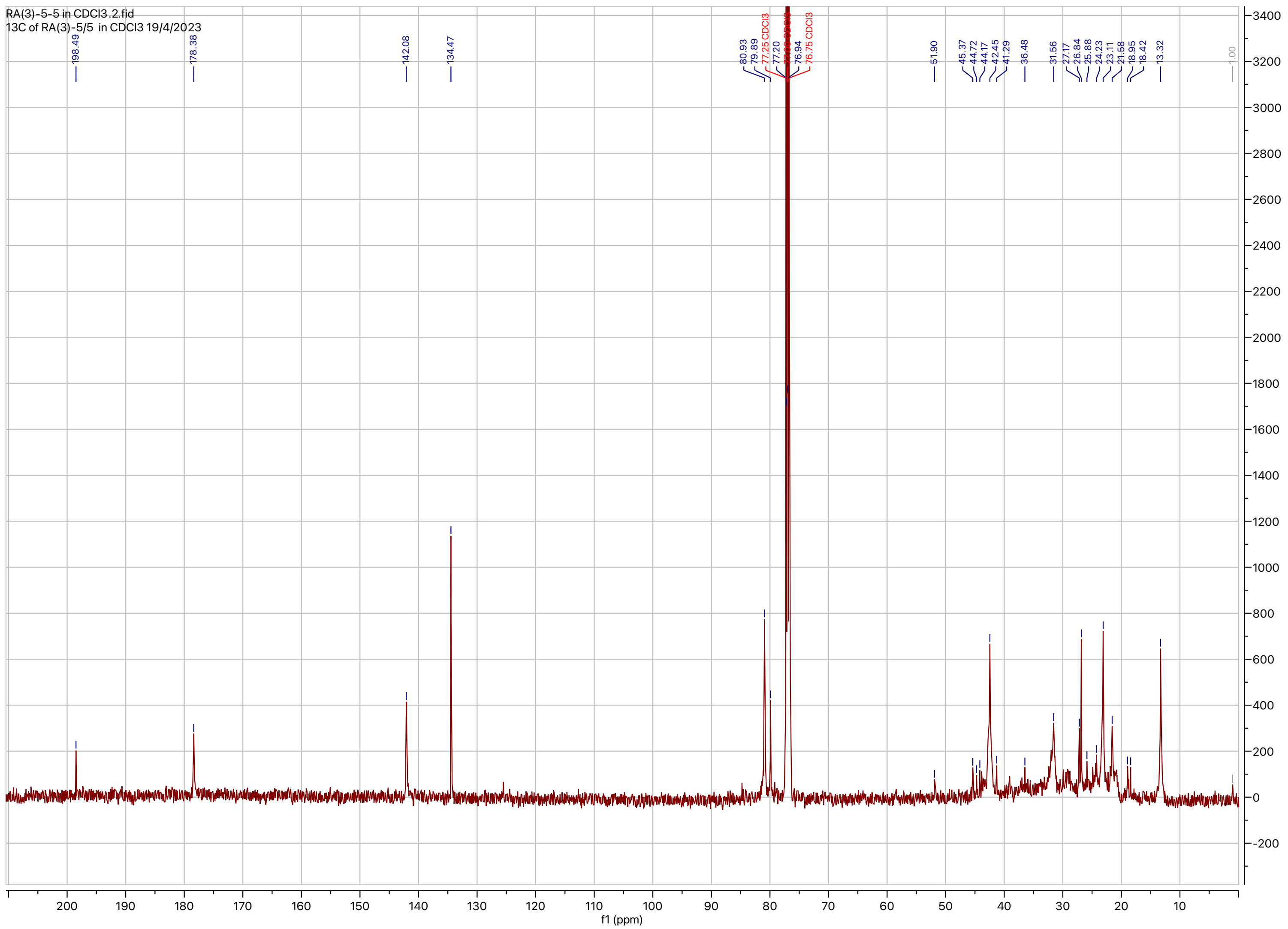

Figure S19. HSQC (500/125 MHz, $CDCl_3$) 2D spectrum of Diacarnusin C (**3**)

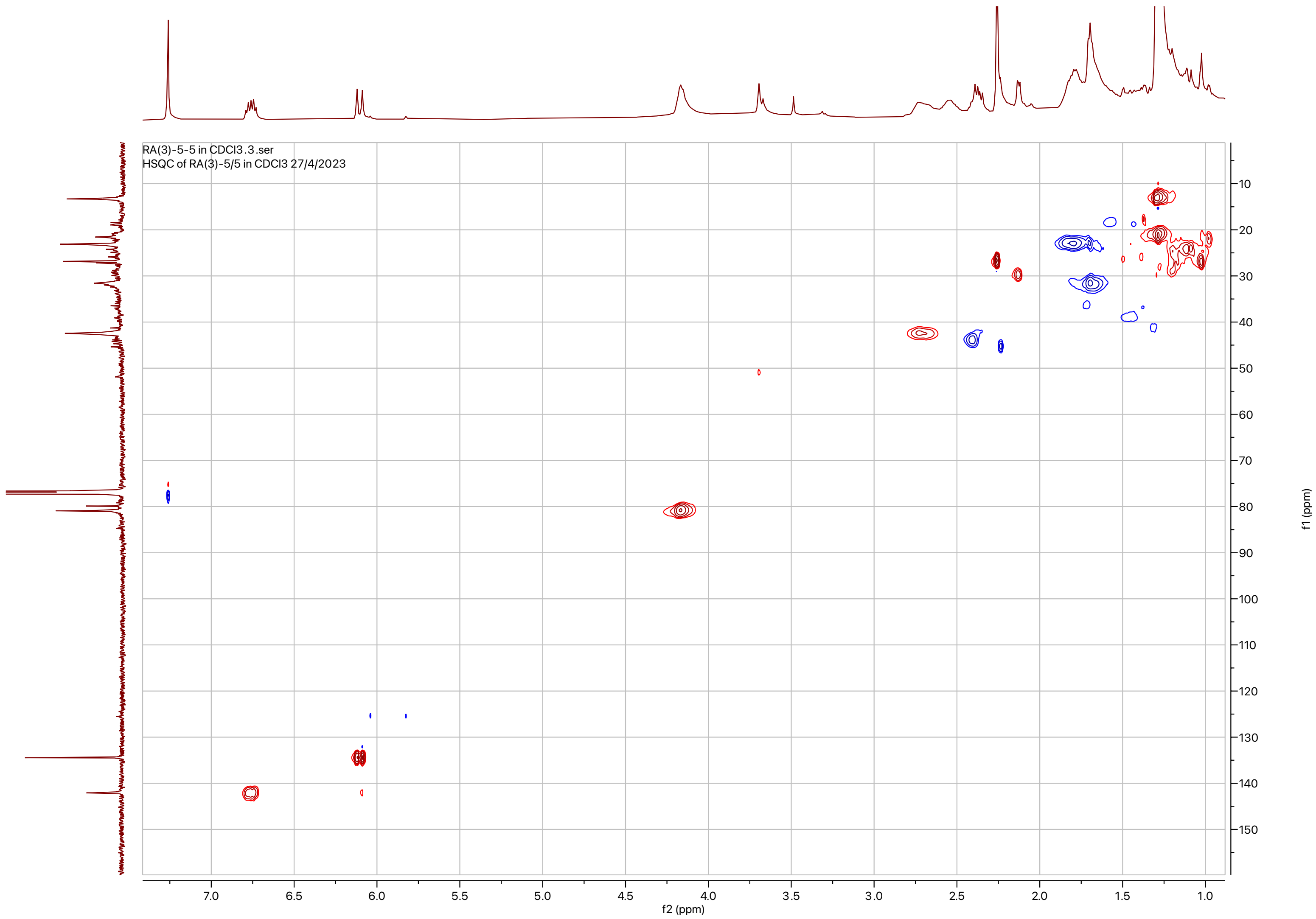

Figure S20. HMBC (500/125 MHz, $CDCl_3$) 2D spectrum of Diacarnusin C (**3**)

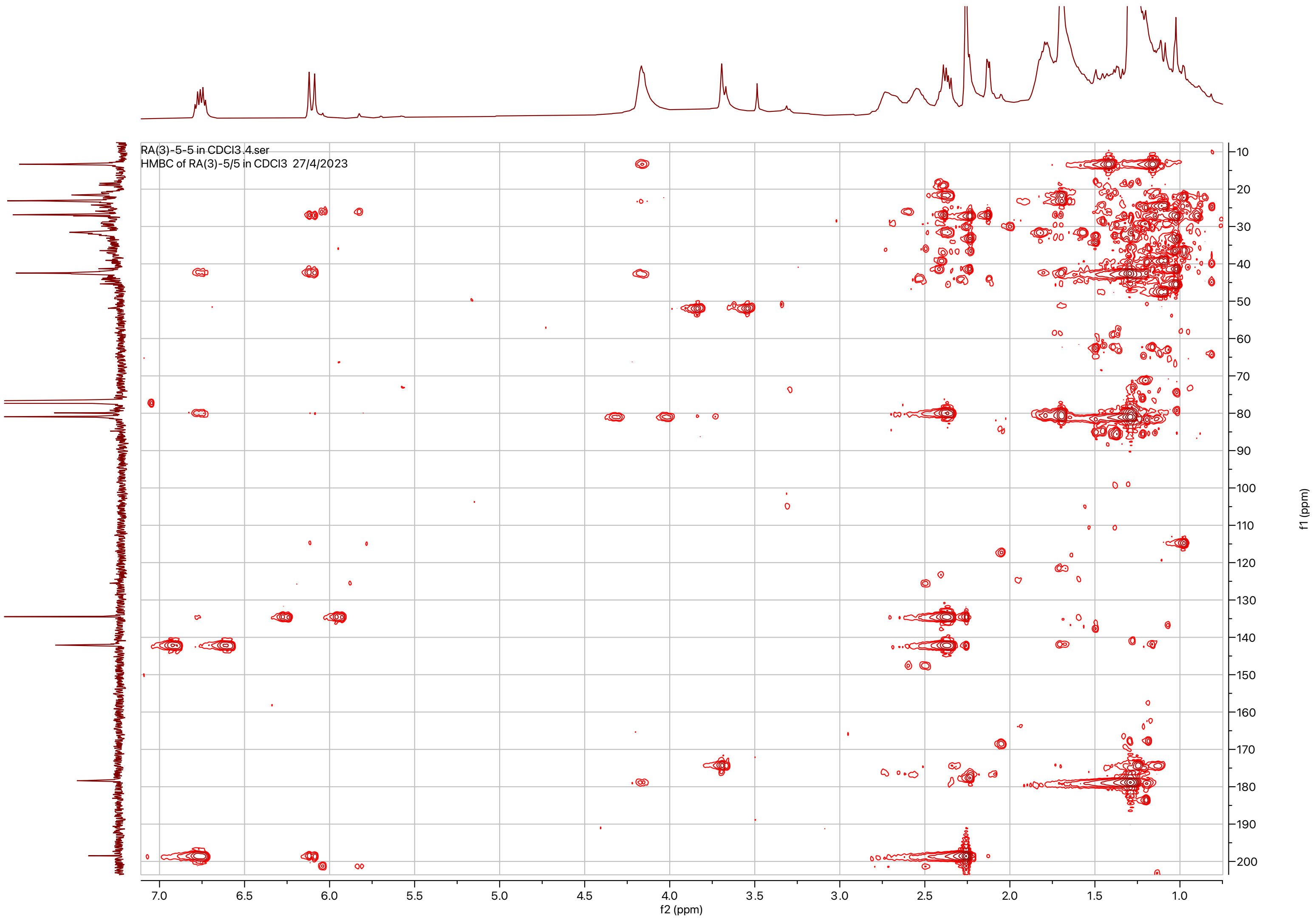

Figure S21. COSY (500 MHz, $CDCl_3$) 2D spectrum of Diacarnusin C (**3**)

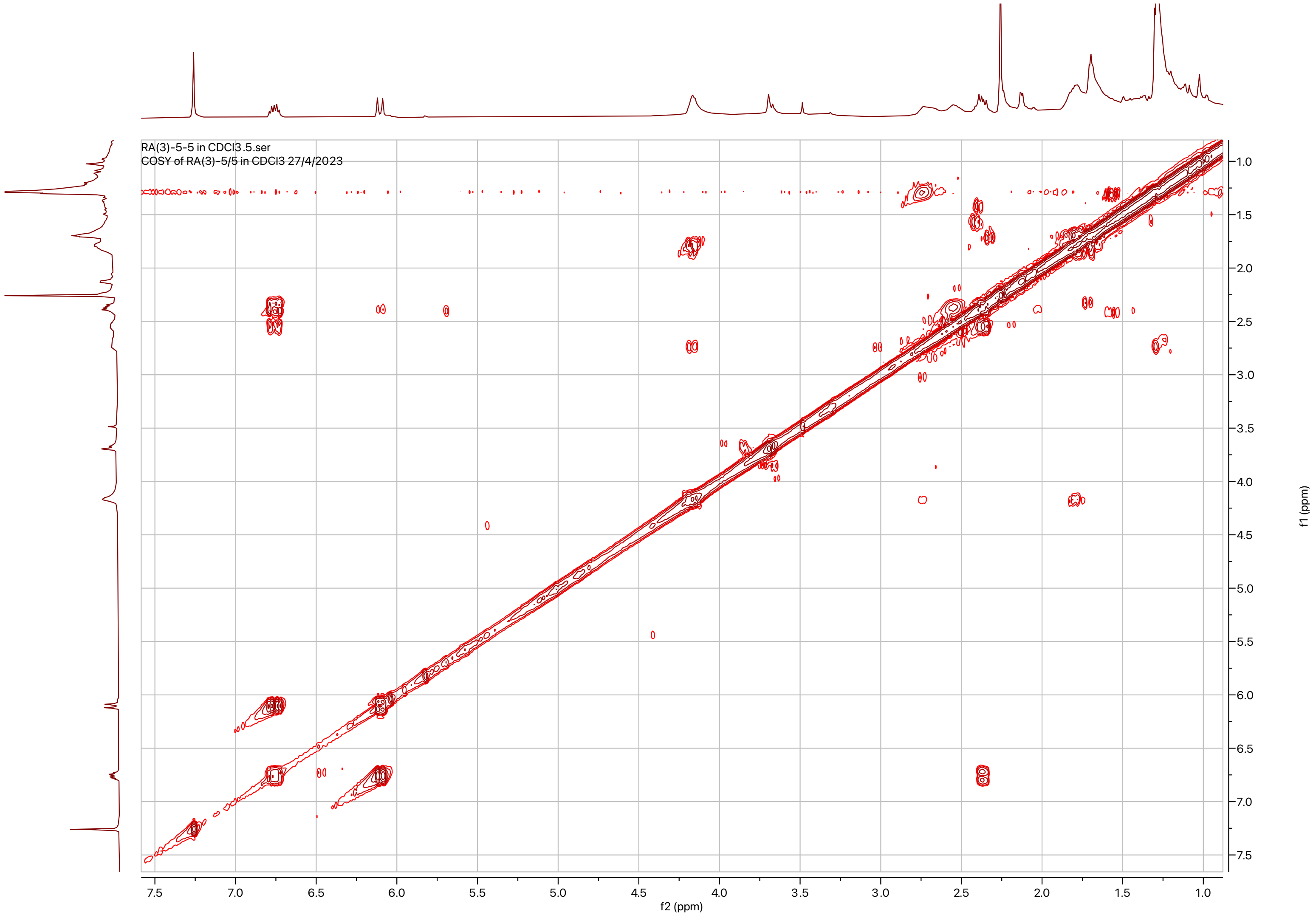

Figure S22. ^{1}H NMR (500 MHz, DMSO-d_6) spectrum of Diacarnusin C (**3**)

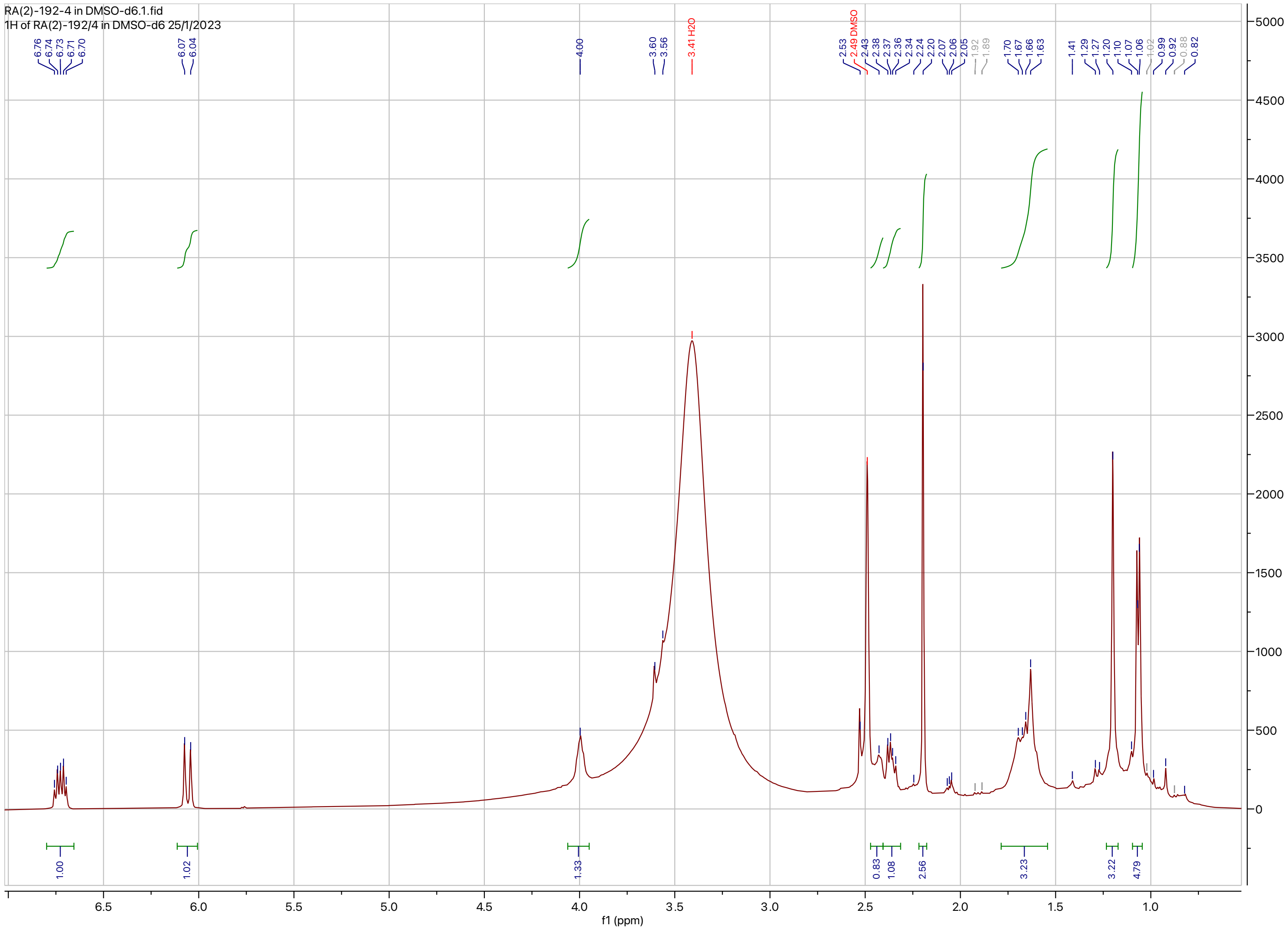

Figure S23. ^{1}H NMR (500 MHz, DMSO-d_6) spectrum of the mixture of Diacarnusin C (**3**) and the transformation products **3a** and **3b**

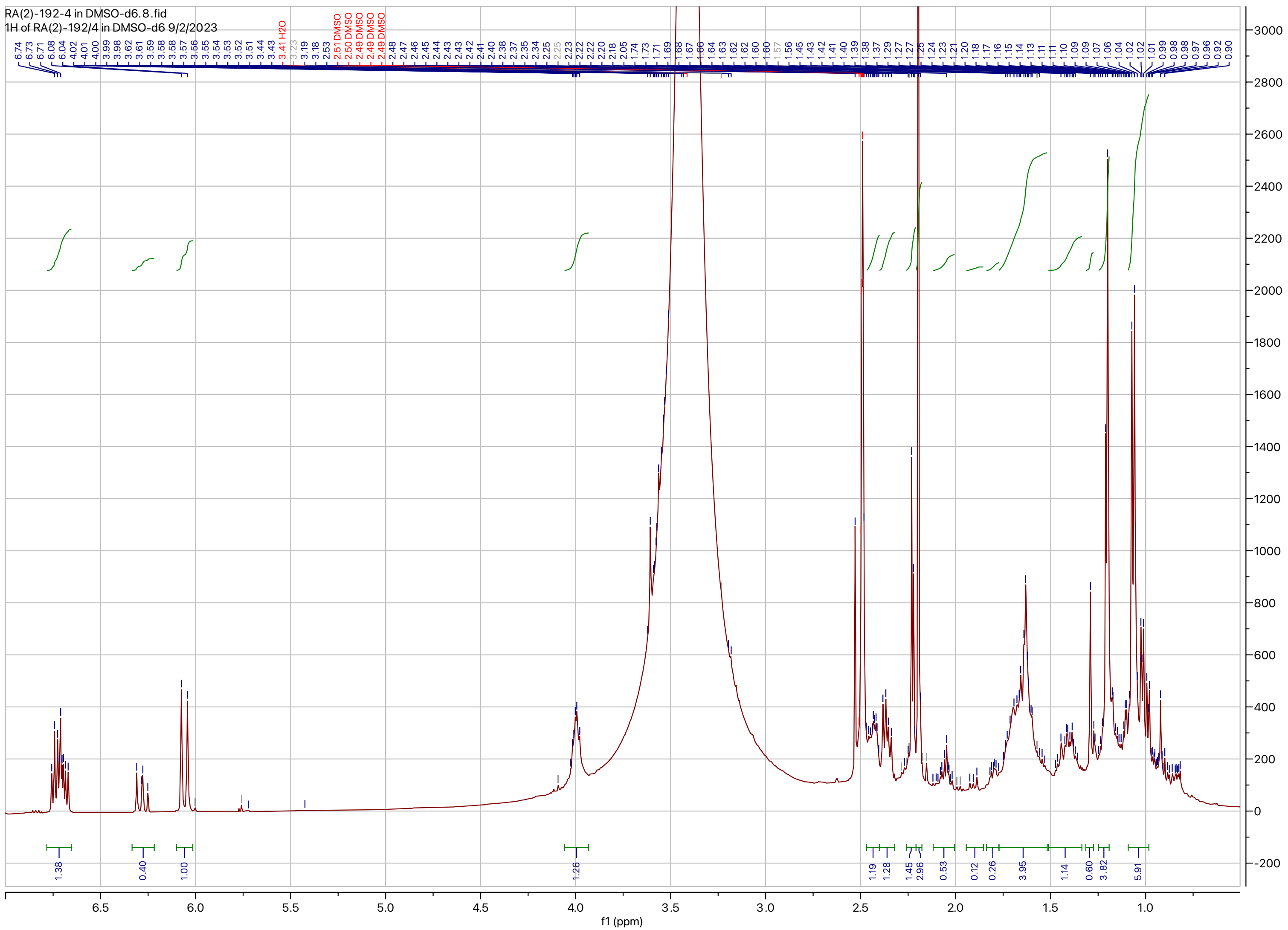

Figure S24. ^{13}C NMR (125 MHz, DMSO-d_6) spectrum of Diacarnusin C (**3**)

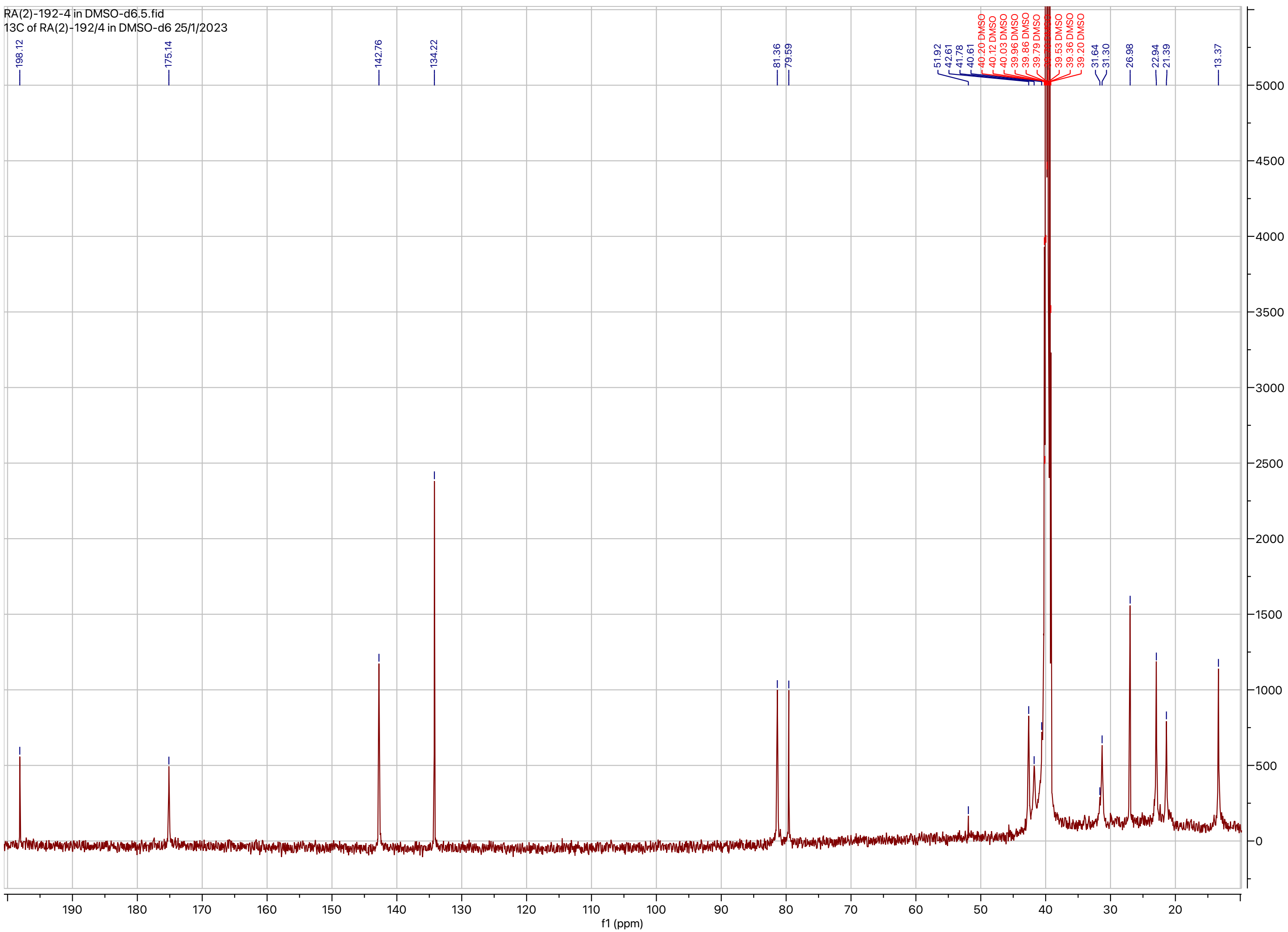

Figure S25. ^{13}C NMR (125 MHz, DMSO-d_6) spectrum of the mixture Diacarnusin C (**3**) and the transformation products **3a** and **3b**

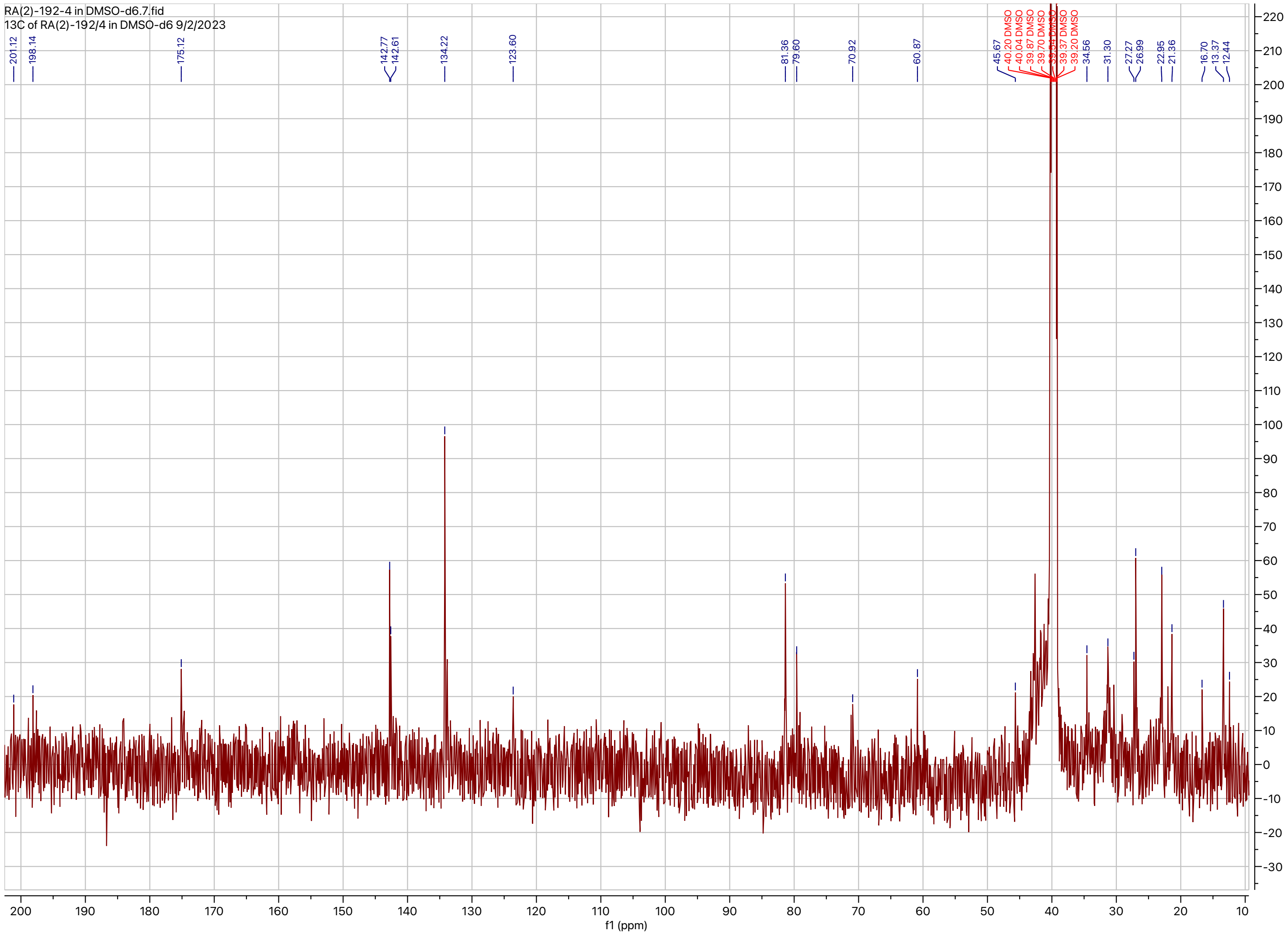

Figure S26. HSQC (500/125 MHz, DMSO-d_6) 2D spectrum of the mixture of Diacarnusin C (**3**) and the transformation products **3a** and **3b**

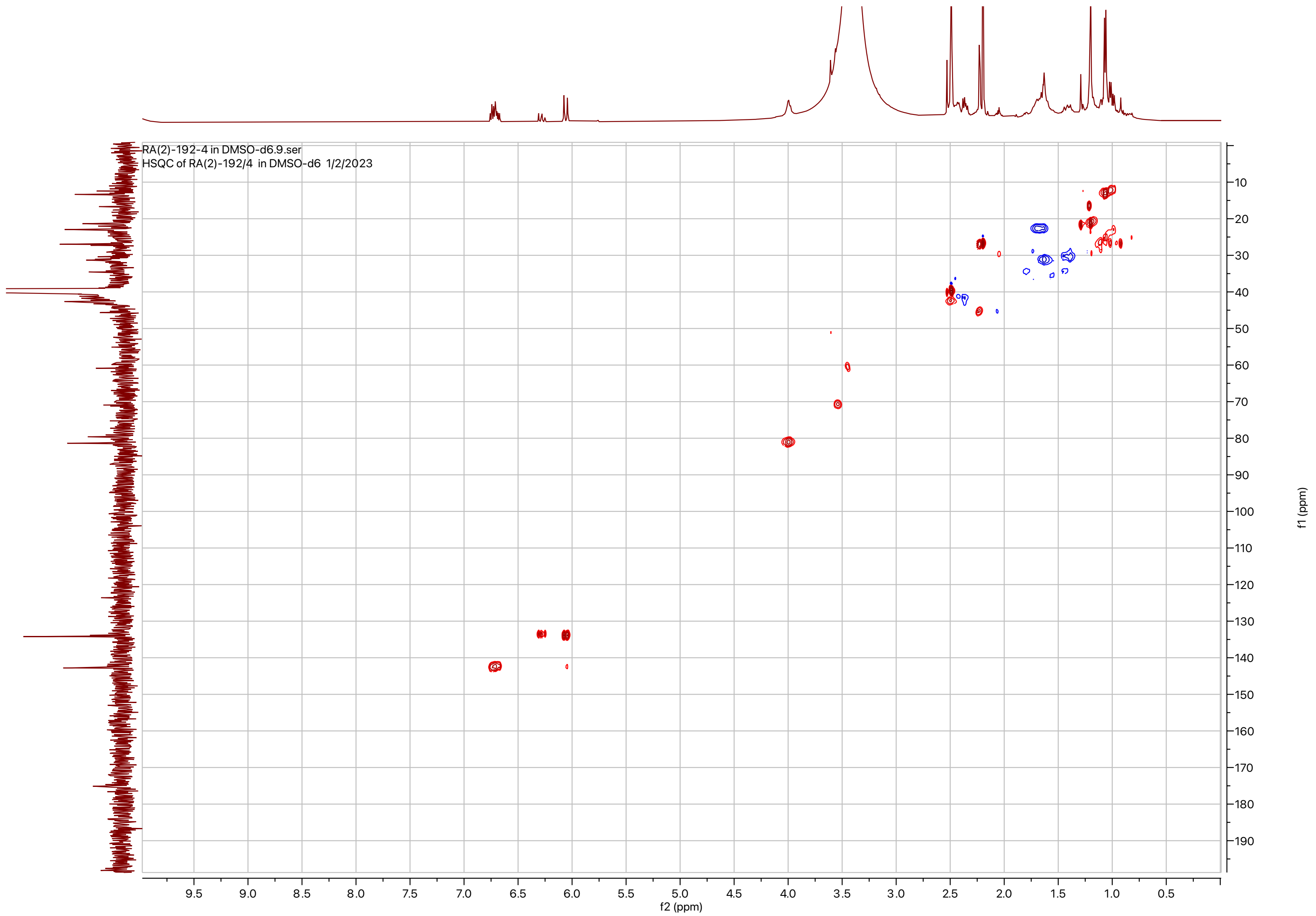

Figure S27. HMBC (500/125 MHz, DMSO-d_6) 2D spectrum of the mixture of Diacarnusin C (**3**) and the transformation products **3a** and **3b**

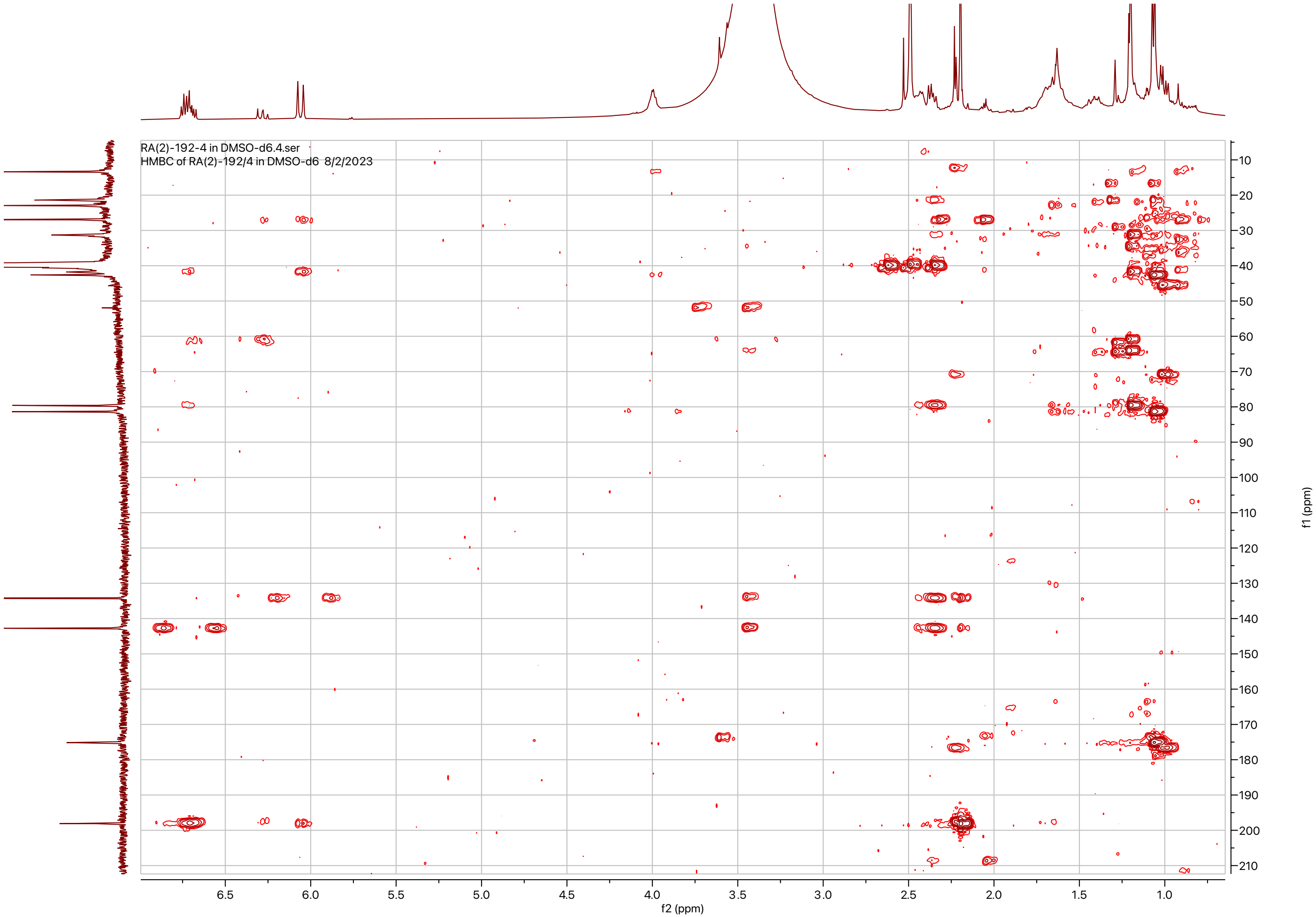

Figure S28. COSY (500 MHz, DMSO-d_6) 2D spectrum of the mixture of Diacarnusin C (**3**) and the transformation products **3a** and **3b**

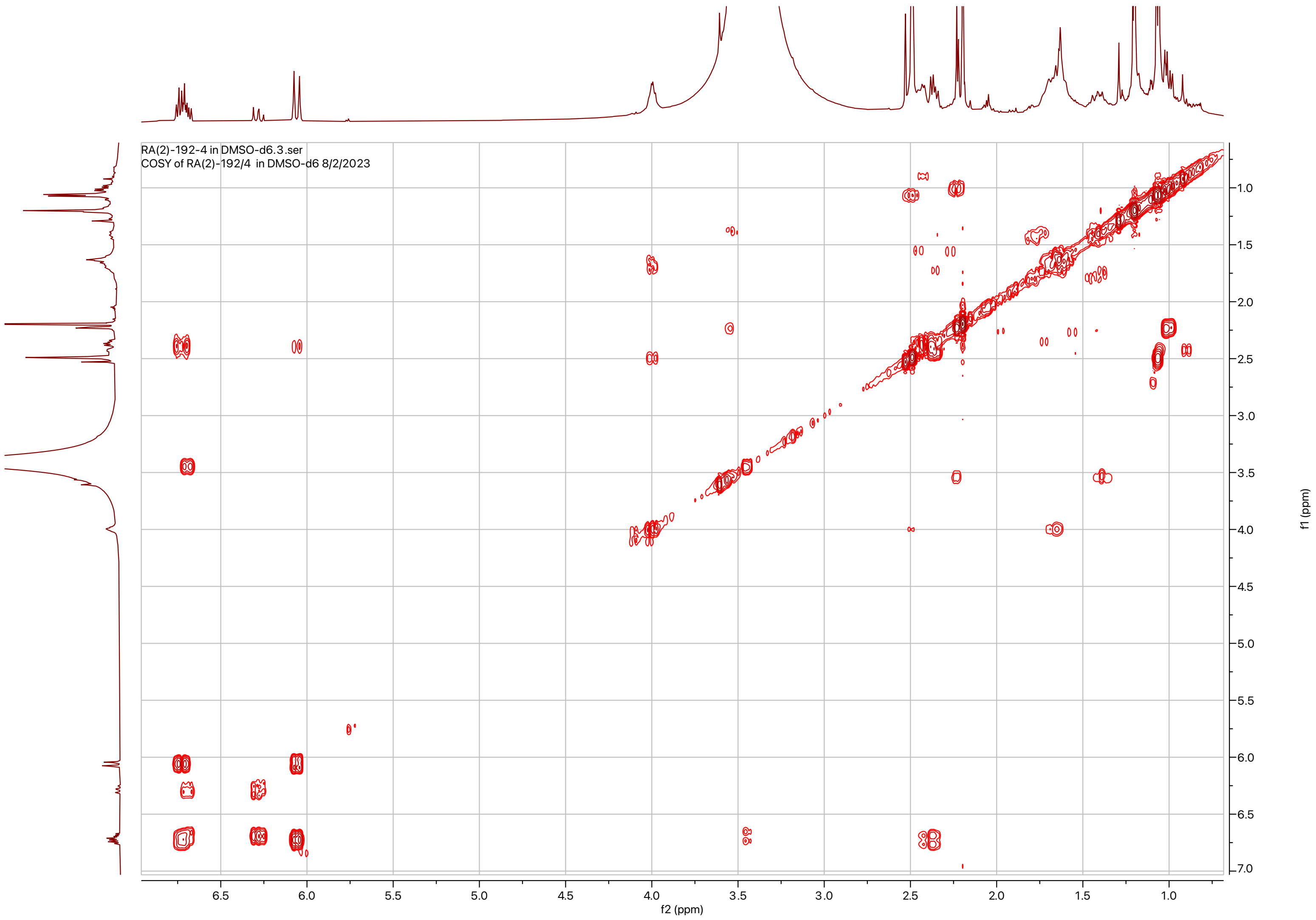

Figure S29. TOCSY (500 MHz, DMSO-d_6) 2D spectrum of the mixture of Diacarnusin C (**3**) and the transformation products **3a** and **3b**

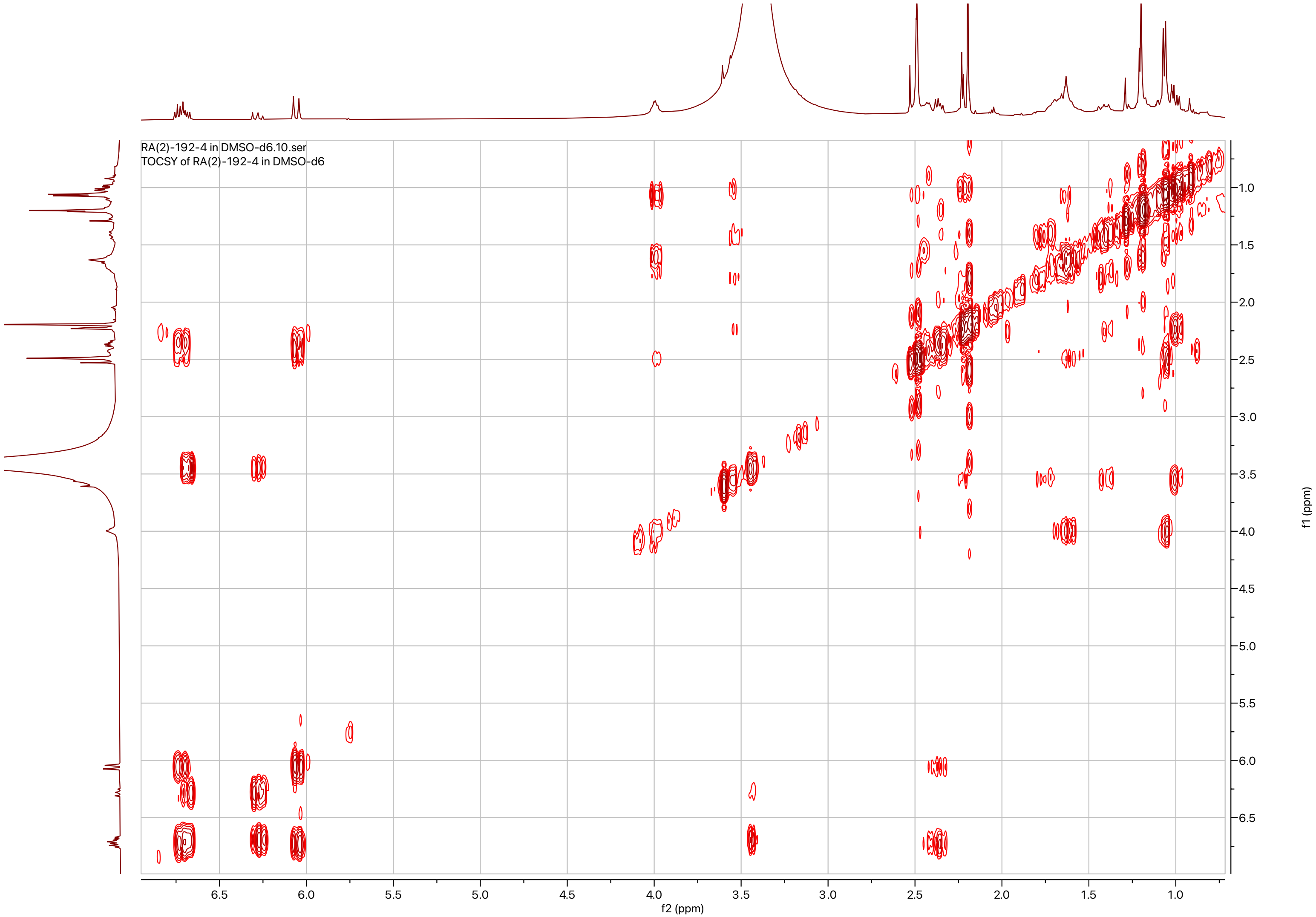

Figure S30. NOESY (500 MHz, DMSO-d_6) 2D spectrum of the mixture of Diacarnusin C (**3**) and the transformation products **3a** and **3b**

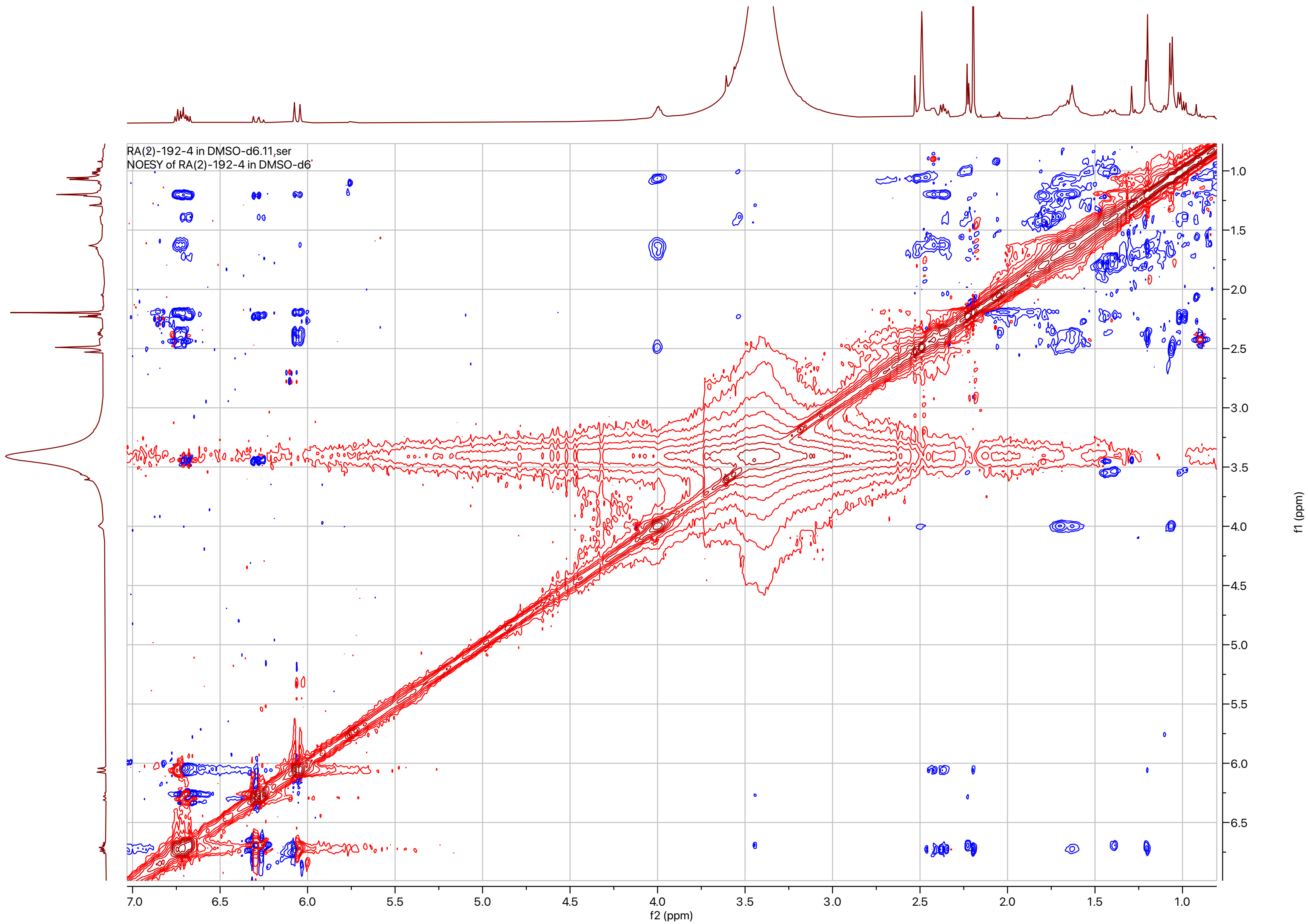

Figure S31. HR ESI MS of the mixture of Diacarnusin C (**3**) and the transformation products **3a** and **3b**

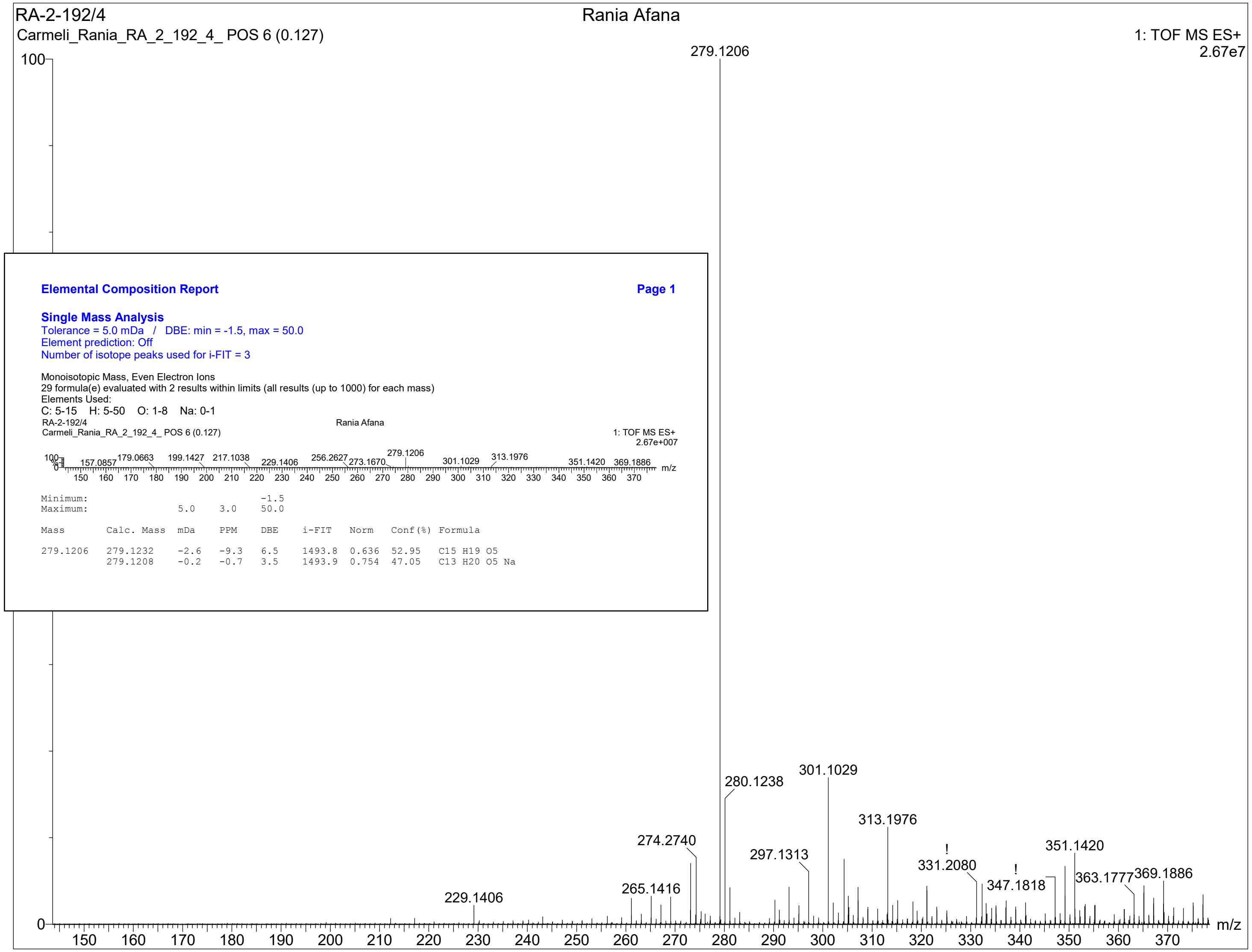

Elemental Composition Report Page 1

Single Mass Analysis
Tolerance = 5.0 mDa / DBE: min = -1.5, max = 50.0
Element prediction: Off
Number of isotope peaks used for i-FIT = 3

Monoisotopic Mass, Even Electron Ions
29 formula(e) evaluated with 2 results within limits (all results (up to 1000) for each mass)
Elements Used:
C: 5-15 H: 5-50 O: 1-8 Na: 0-1

RA-2-192/4 Rania Afana
Carmeli_Rania_RA_2_192_4_ POS 6 (0.127) 1: TOF MS ES+ 2.67e+007

157.0857 179.0663 199.1427 217.1038 229.1406 256.2627 273.1670 279.1206 301.1029 313.1976 351.1420 369.1886 m/z

Mass	Calc. Mass	mDa	PPM	DBE	i-FIT	Norm	Conf(%)	Formula
Minimum:				-1.5				
Maximum:		5.0	3.0	50.0				
279.1206	279.1232	-2.6	-9.3	6.5	1493.8	0.636	52.95	C15 H19 O5
	279.1208	-0.2	-0.7	3.5	1493.9	0.754	47.05	C13 H20 O5 Na

Table S5. NMR Data of Diacarnusin C (**3**) in $CDCl_3$[a]

Position	δ_C, type[b]	δ_H, multiplicity, *J* in Hz	HMBC correlations[c]	COSY correlations
1	178.4, C		H-3, H3-12	
2	42.5, CH	2.73, m		H-3, H3-12
3	80.9, CH	4.16, dt (4.3, 7.8)	H-4,4’, H2-5 H3-12	H-2, H-4,4’
4	24.3, CH2	1.82, m	H2-5	H-3, H2-5
4’		1.79, m		H-3, H2-5
5	31.6, CH2	1.69, m	H-4, H-7’, H3-13	H2-4
6	79.9, C		H-4,4’, H2-5, H-7’, H-8, H-9	
7	42.4, CH2	2.55, m	H-3,8,9, H2-4,5, H3-13	H-7’, H-8
7’		2.37, dd (14.3, 8.0)		H-7, H-8
8	142.1, CH	6.76, ddd (16.0, 8.0, 7.5)	H-7’ H2-5,	H2-7, H-9
9	134.5, CH	6.10, d (16.0)	H-7’, H-8, H3-11	H2-7, H-8
10	198.5, C		H-8, H-9, H3-11	
11	26.8, CH3	2.26, s	H-9	
12	13.3, CH3	1.29, d (7.1)	H-3	
13	21.6, CH3	1.28, s	H2-5	

[a]500 MHz for ^{1}H, 125 MHz for ^{13}C; [b]From HSQC; [c]HMBC correlations, optimized for $J_{C\text{-}H}$=8 Hz, are from carbon to the indicated proton(s).

Table S6. NMR Data of Diacrnusin C (**3**) in DMSO-d_6[a]

Position	δ_C, multiplicity[b]	δ_H, multiplicity, *J* in Hz	HMBC correlations[c]	COSY correlations	NOESY correlations
1	175.1, C		H-3, H_3-12		
2	42.6, CH	2.50, m	H-3, H_3-12	H-3, H_3-12	H-3, H_2-4, H_3-12
3	81.4, CH	4.00, dt (4.0, 7.6)	H_2-5	H-2,4,4’	H-2, H_2-4, H_3-12
4	22.9, CH_2	1.70, m	H_2-5	H-3,4’, H_2-5	H-2,3, H_3-13
		1.64, m		H-3,4, H_2-5,	H-2,3
5	31.3, CH_2	1.63, m	H-4,7’, H_3-13	H_2-4	H-8, H_2-7, H_3-13
6	79.6, CH		H-4’,8, H_2-7, H_3-13		
7	41.8, CH_2	2.44, brdd (14.2, 6.4)	H_2-5, H-8,9 H_3-13	H-7’,8	H-8,9, H_2-5
		2.36, dd (14.2, 7.7)		H-7,8	H-8,9, H_2-5
8	142.8, CH	6.73, dt (15.9, 7.9)	H_2-7, H_3-11	H-9, H_2-7	H_2-,7, H_3-11, 13
9	134.2, CH	6.06, d (15.9)	H_2-7, H_3-11	H-8	H_2-7, H_3-11
10	198.1, C		H-8,9, H_3-11		
11	27.0, CH_3	2.20, s	H-9		H-8,9
12	13.4, CH_3	1.07, d (6.9)	H-3	H-2	H-2,3
13	21.4, CH_3	1.20, s	H-7’		H-8, H_2-4,7

[a]500 MHz for ^{1}H, 125 MHz for ^{13}C; [b]From HSQC; [c]Optimized for $J_{C\text{-}H}$=8 Hz.

Table S6a. NMR Data of Diacrnusin C major product (**3a**) in DMSO-d_6[a]

Position	δ_C, multiplicity[b]	δ_H, multiplicity, J in Hz	HMBC correlations[c]	COSY correlations	NOESY correlations
1	176.6, C		H-2, H_3-12		
2	45.5, CH	2.24, m		H-3, H_3-12	
3	70.9, CH	3.55, m	H-2, H_3-12	H-2, H_2-4	H-4, H_3-12
4	30.3, CH_2	1.44, m		H-3	H-3
		1.35, m		H-3	
5	34.5, CH_2	1.79, m			
		1.42, m			
6	64.1, C		H-4', H_3-13		
7	60.9, CH	3.45, d (6.7)	H-8,9, H_3-13	H-8,9	H-4',8,9
8	142.6, CH	6.69, dd (16.0, 6.7)	H-7	H-7,9	H-7, H_3-11, H_3-13
9	133.8, CH	6.29, d (16.0)	H-7, H_3-11	H-8,7	H-7, H_3-11, H_3-13
10	197.6, C		H-8, H_3-11		
11	27.3, CH_3	2.23, s			H-8,9
12	12.4, CH_3	1.02, d (6.9)		H-2	H-3
13	16.6, CH_3	1.21, s			H-8,9

[a]500 MHz for ^{1}H, 125 MHz for ^{13}C; [b]From HSQC; [c]Optimized for $J_{C\text{-}H}$=8 Hz.

Table S6b. NMR Data of Diacrnusin C minor product (**3b**) in DMSO-d_6[a]

Position	δ_C, multiplicity[b]	δ_H, multiplicity, J in Hz	HMBC correlations[c]	COSY correlations	NOESY correlations
1	176.7, C		H-2, H_3-12		
2	45.5, CH	2.23, m		H-3, H_3-12	
3	70.9, CH	3.53, m	H-2, H_3-12	H-2, H_2-4	H-4', H_3-12
4	34.2, CH_2	1.72, m		H-3	
		1.39, m		H-3	
5	29.0, CH_2	1.73, m			H-8,9
		1.39, m			H-3,8
6	64.4, C		H-5', H_3-13		
7	61.8, CH	3.44, d (6.7)	H-8,9 H_3-13	H-8,9	H-8,9, H_3-11, H_3-13
8	142.6, CH	6.69, dd (16.0, 6.7)	H-7	H-7,9	H-7, H_2-5
9	133.9, CH	6.27, d (16.0)	H-7, H_3-11	H-7,8	H-5,7, H_3-11,
10	197.6, C		H-9, H_3-11		H-8,9
11	27.1, CH_3	2.22, s			
12	12.2, CH_3	0.99, d (6.9)		H-2	H-2
13	22.0, CH_3	1.29, s			H-7

[a]500 MHz for ^{1}H, 125 MHz for ^{13}C; [b]From HSQC; [c]Optimized for $J_{C\text{-}H}$=8 Hz.

Figure S32. ^{1}H NMR (500 MHz, DMSO-d_6) spectrum of Diacarnusin D (**4**)

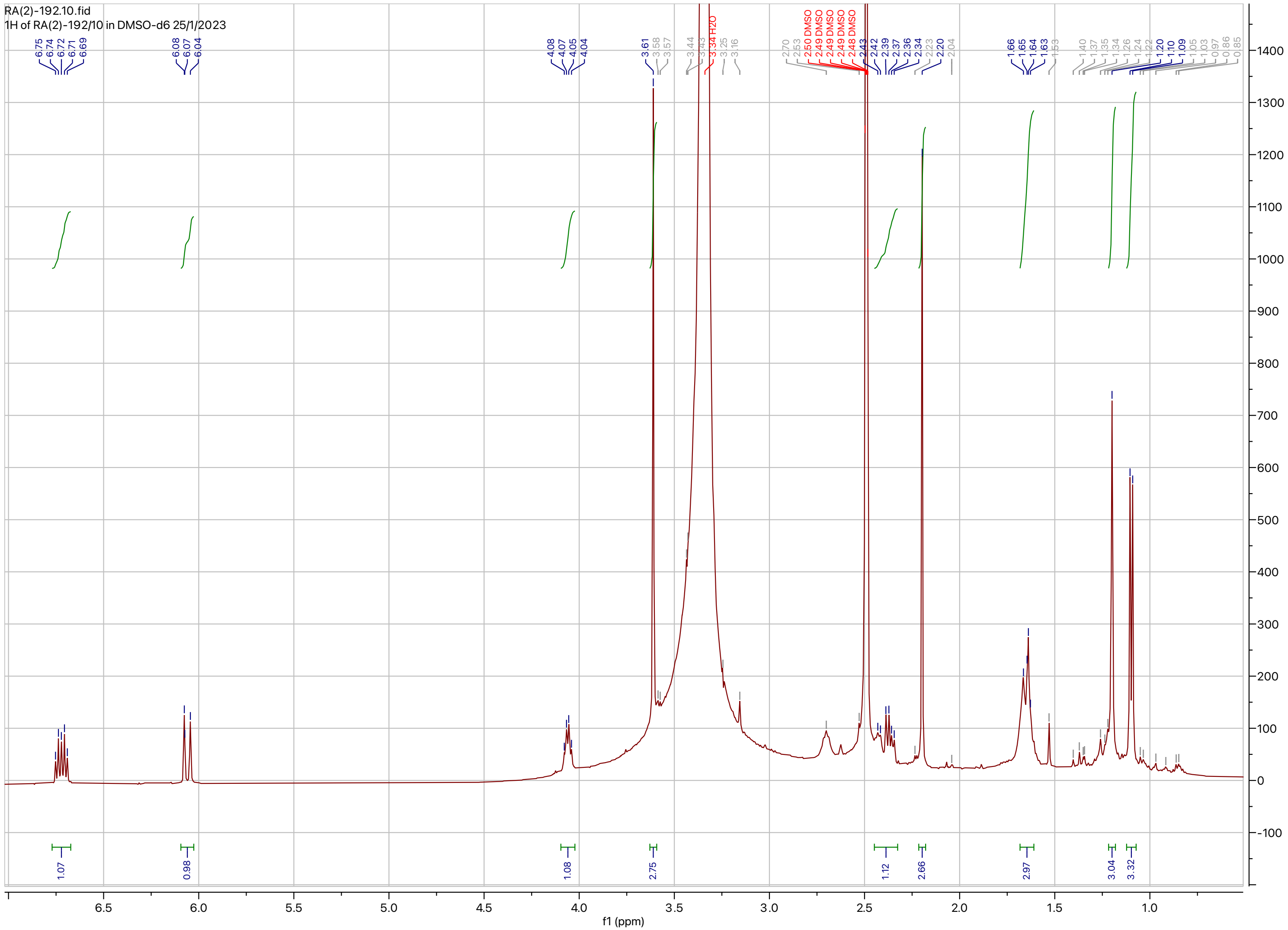

Figure S33. ^{1}H NMR (500 MHz, DMSO-d_6) spectrum of the mixture of Diacarnusin D (**4**) and the transformation products I **4a** and **4b**

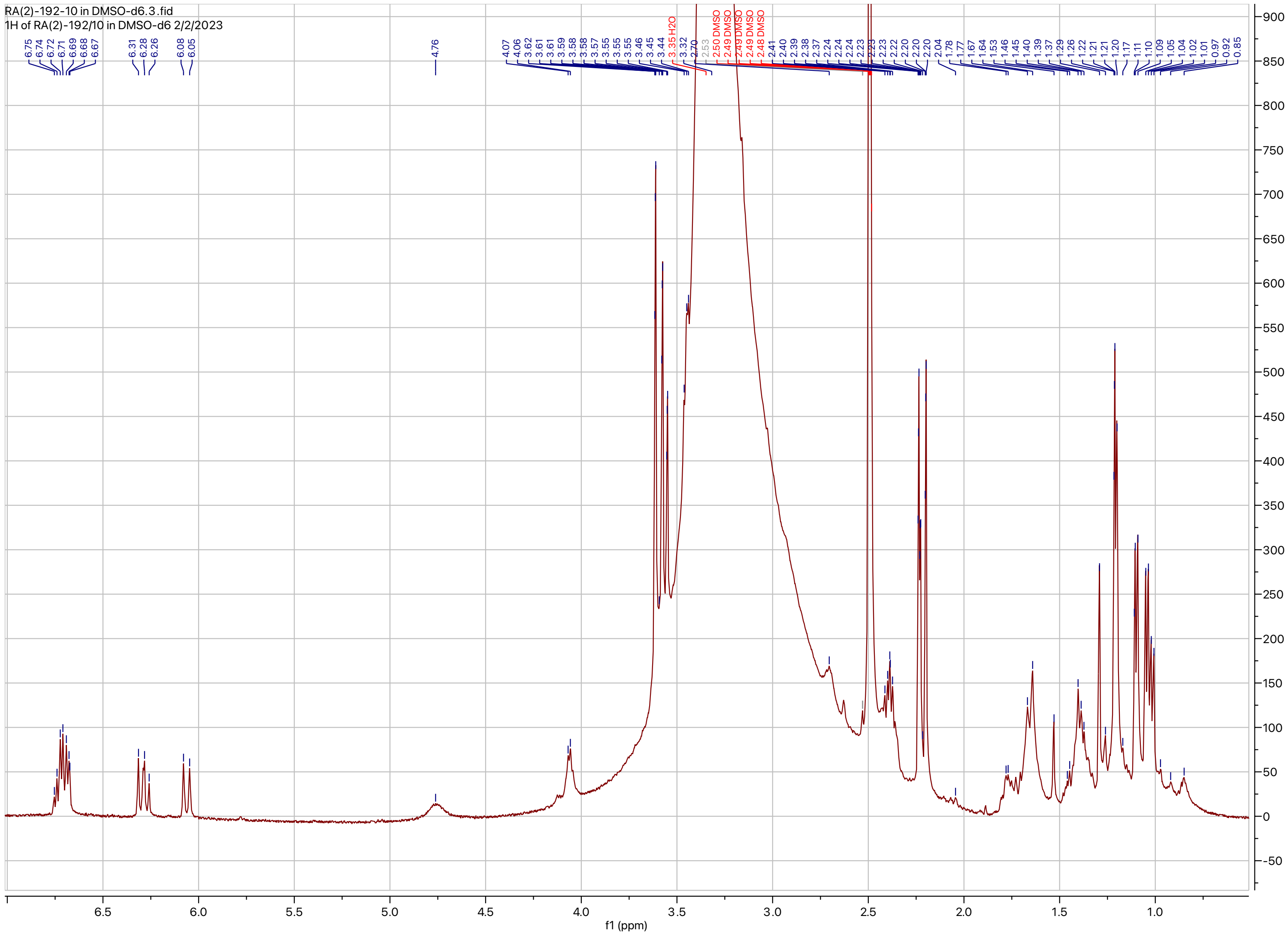

Figure S34. ^{1}H NMR (500 MHz, DMSO-d_6) spectrum of the mixture of Diacarnusin D (**4**) and the transformation products II **4a** and **4b**

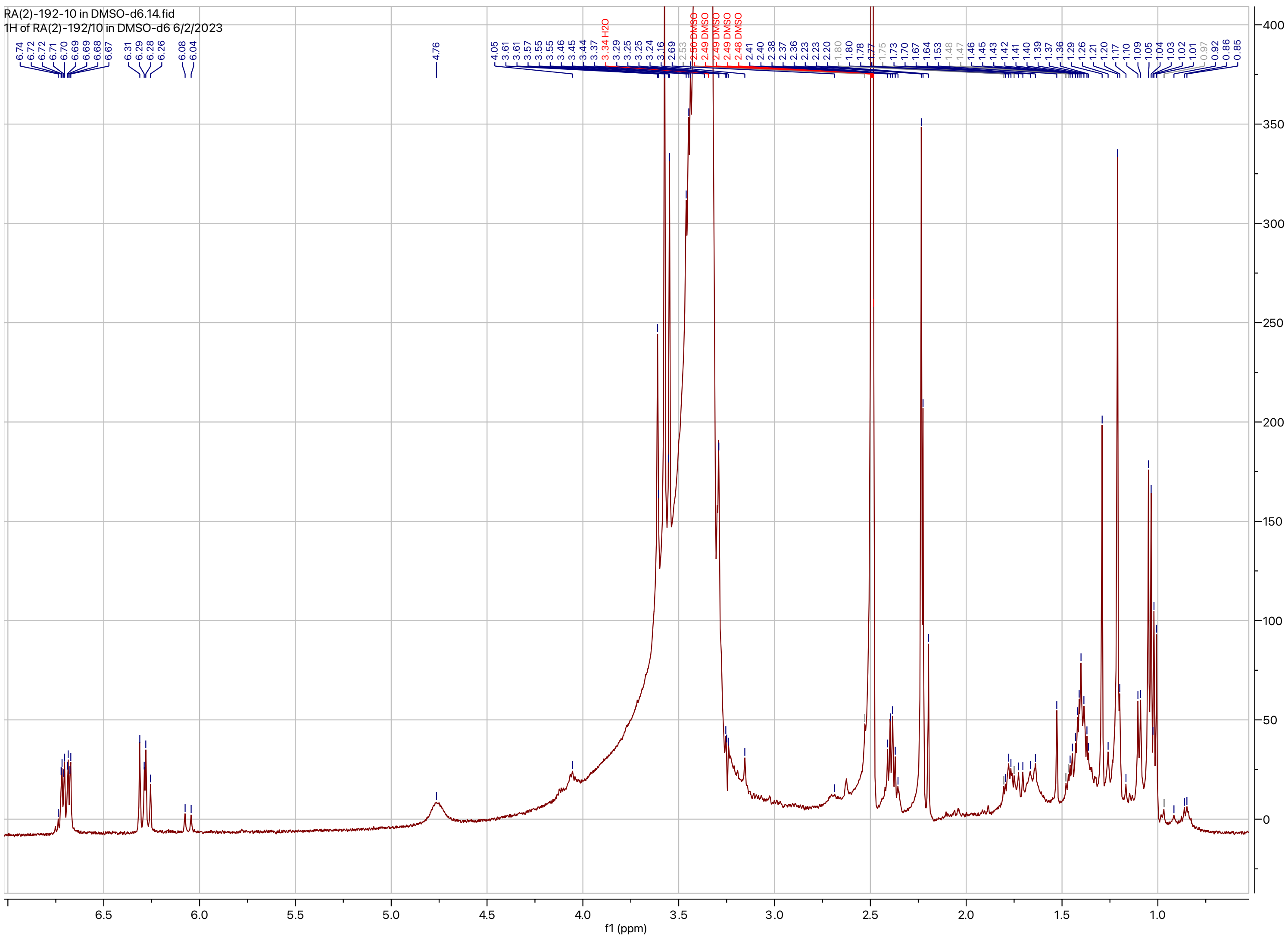

Figure S35. ^{13}C NMR (125 MHz, DMSO-d_6) spectrum of the mixture Diacarnusin D (**4**) and the transformation products I **4a** and **4b**

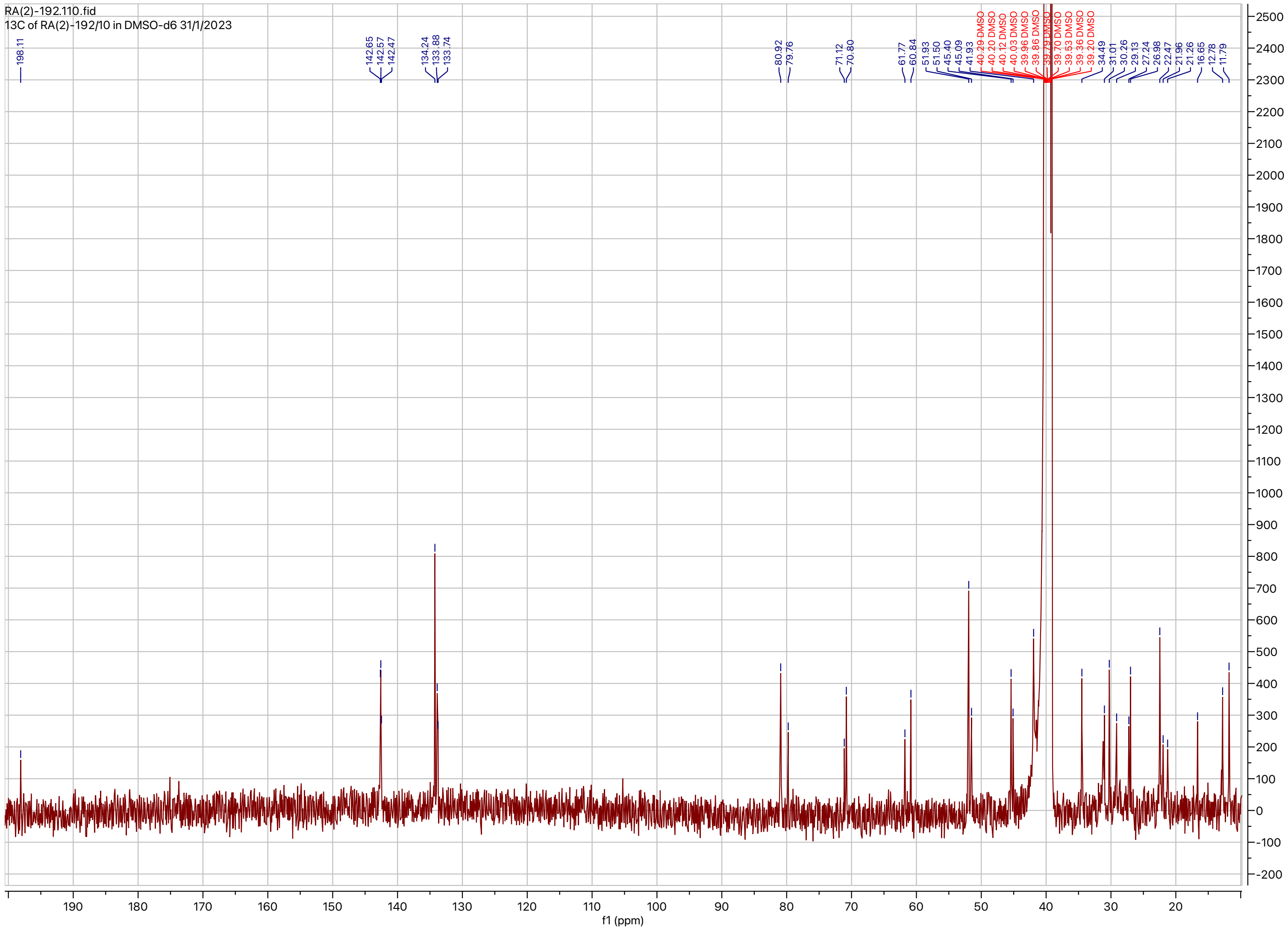

Figure S36. ^{13}C NMR (125 MHz, DMSO-d_6) spectrum of the mixture Diacarnusin D (**4**) and the transformation products II **4a** and **4b**

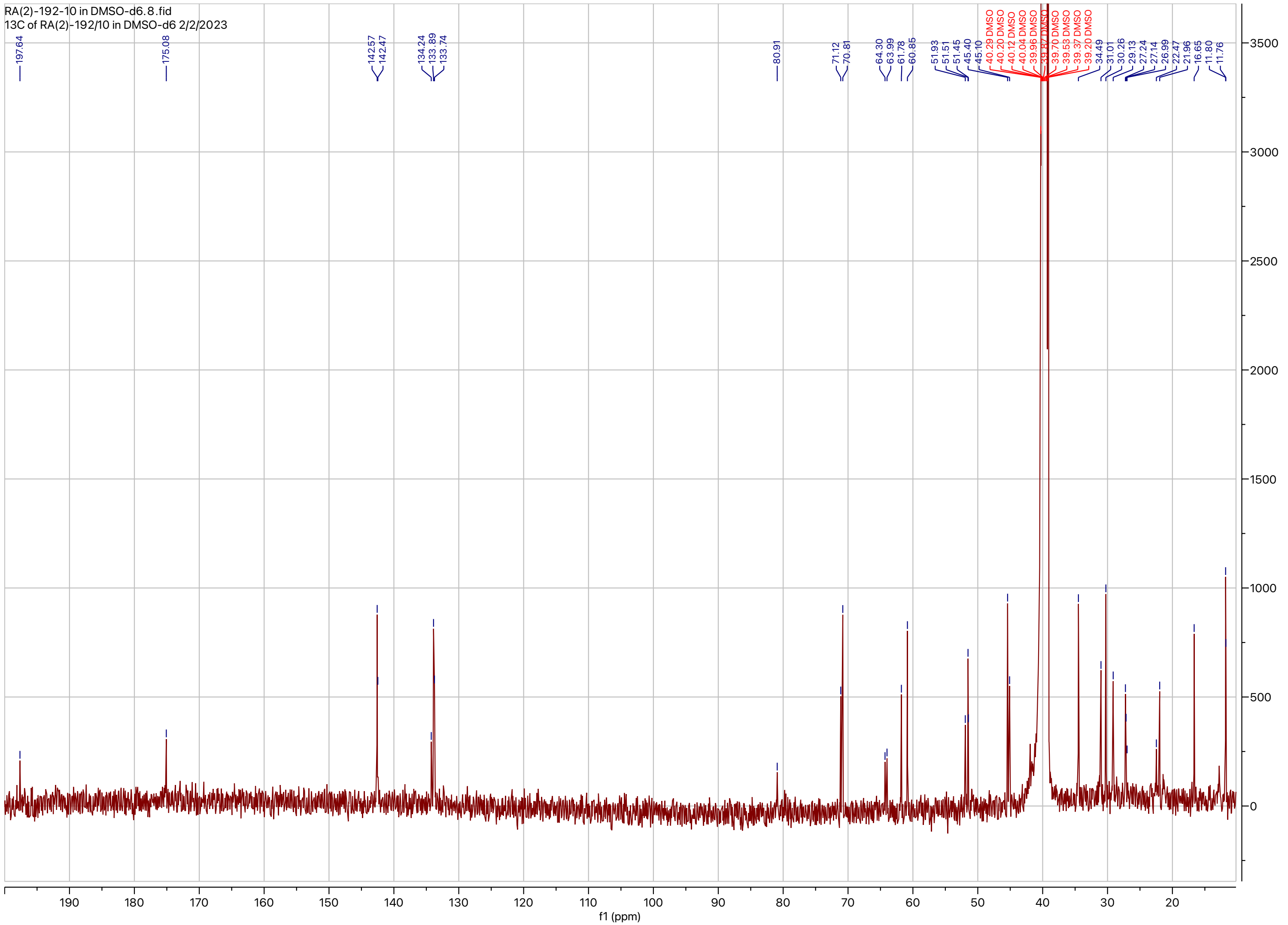

Figure S37. HSQC (500/125 MHz, DMSO-d_6) 2D spectrum of the mixture of Diacarnusin D (**4**) and the transformation products I **4a** and **4b**

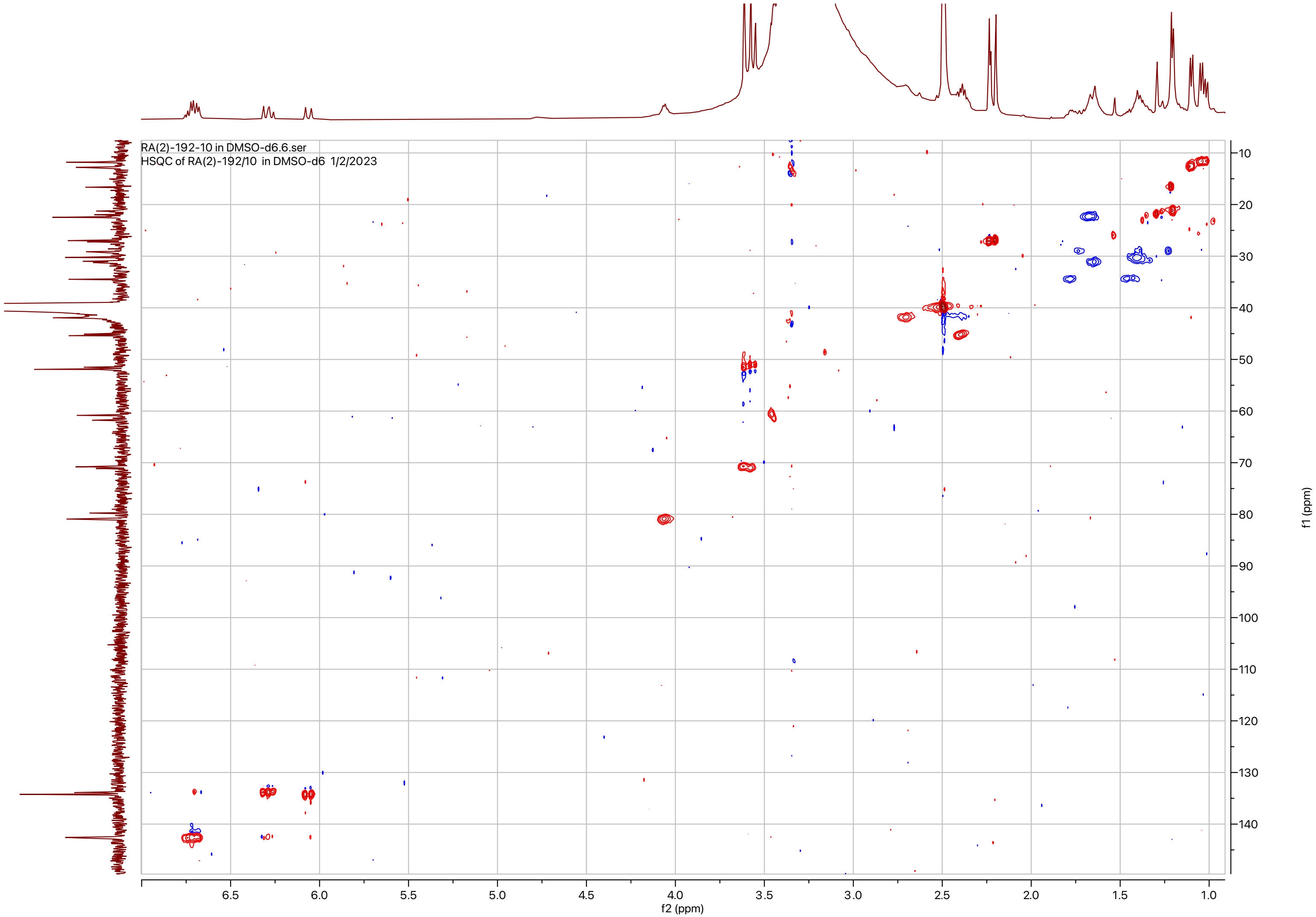

Figure S38. HSQC (500/125 MHz, DMSO-d_6) 2D spectrum of the mixture of Diacarnusin D (**4**) and the transformation products II **4a** and **4b**

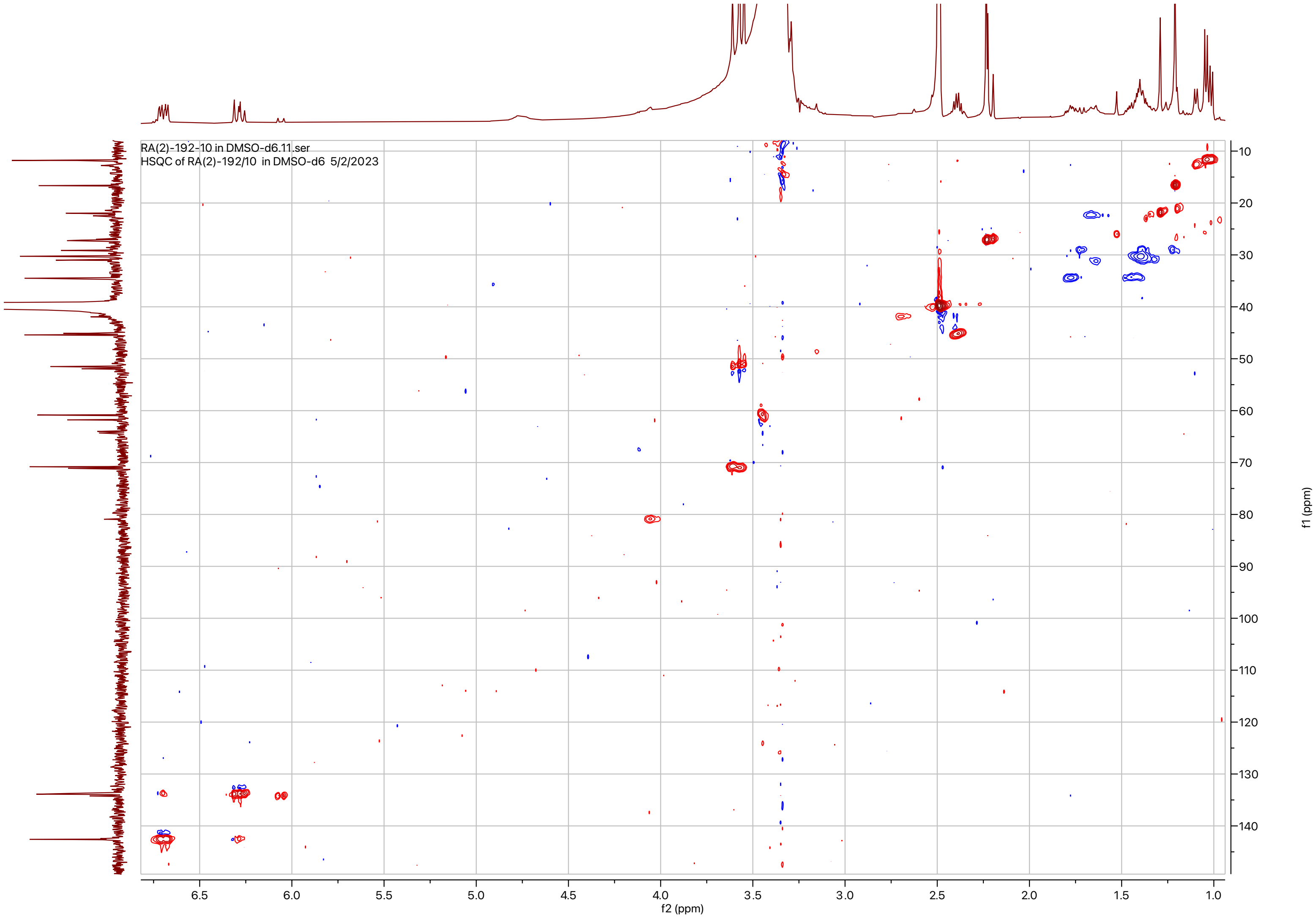

Figure S39. HMBC (500/125 MHz, DMSO-d_6) 2D spectrum of the mixture of Diacarnusin D (**4**) and the transformation products I **4a** and **4b**

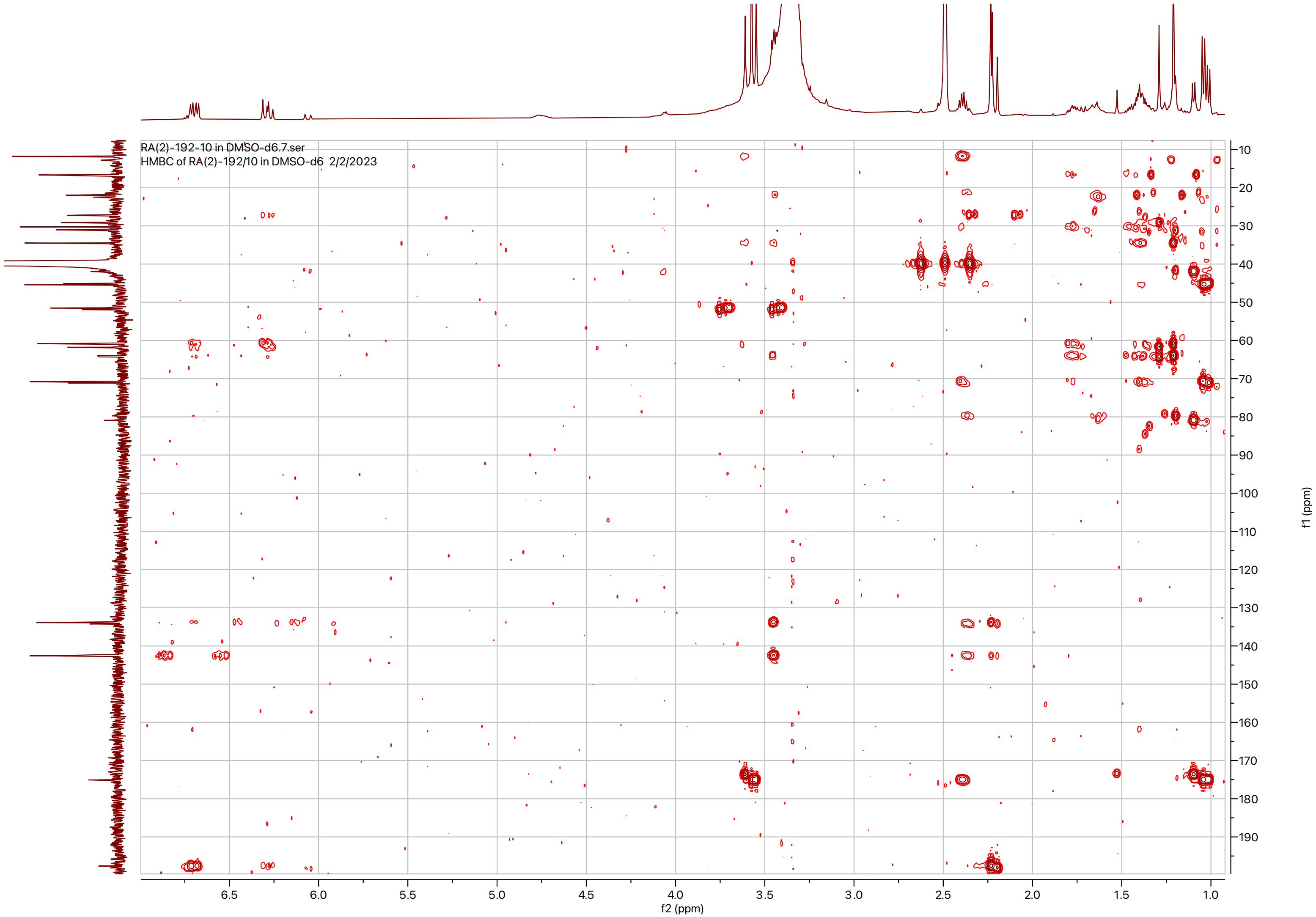

Figure S40. COSY (500 MHz, DMSO-d_6) 2D spectrum of the mixture of Diacarnusin D (**4**) and the transformation products I **4a** and **4b**

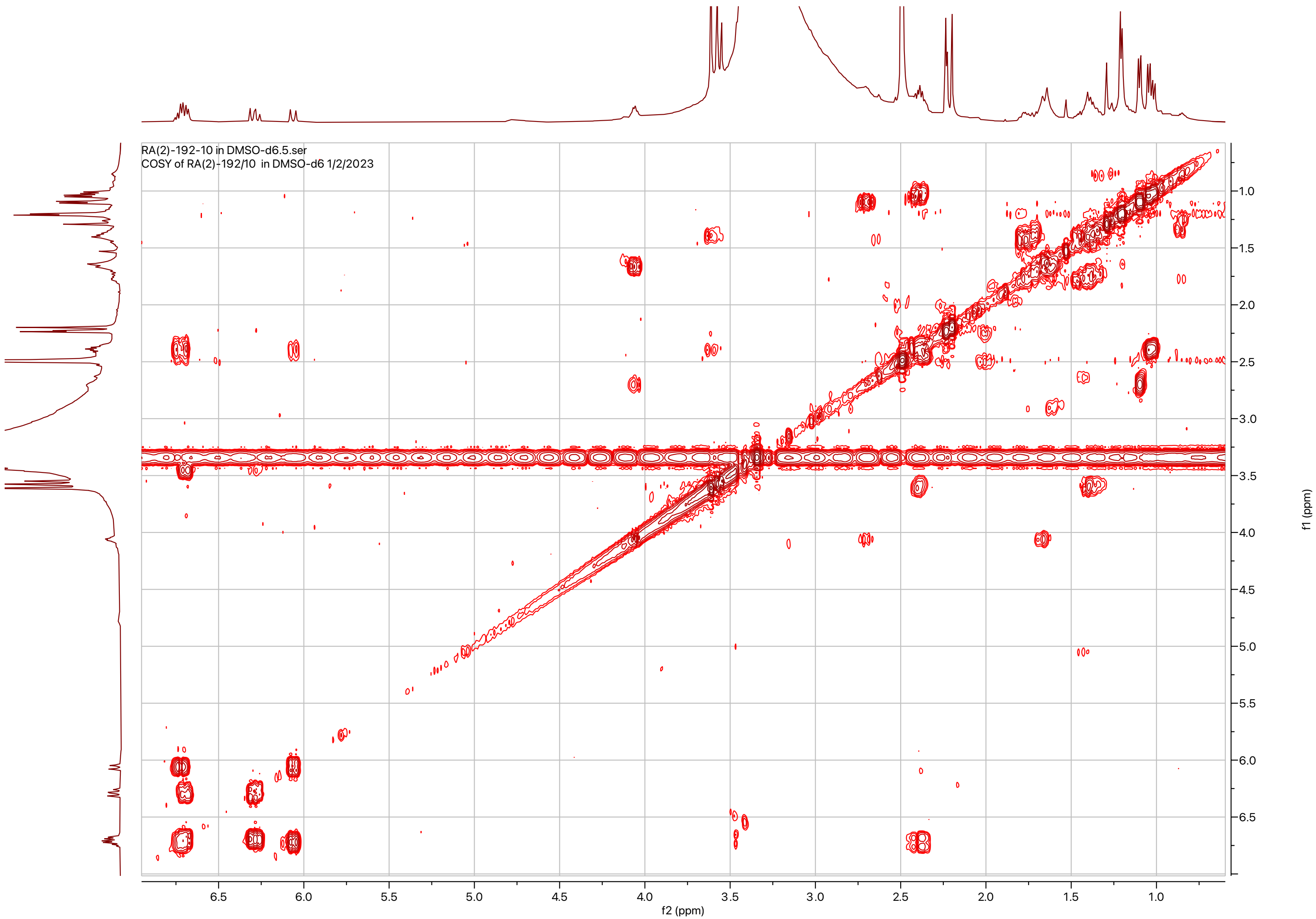

Figure S41. COSY (500 MHz, DMSO-d_6) 2D spectrum of the mixture of Diacarnusin D (**4**) and the transformation products II **4a** and **4b**

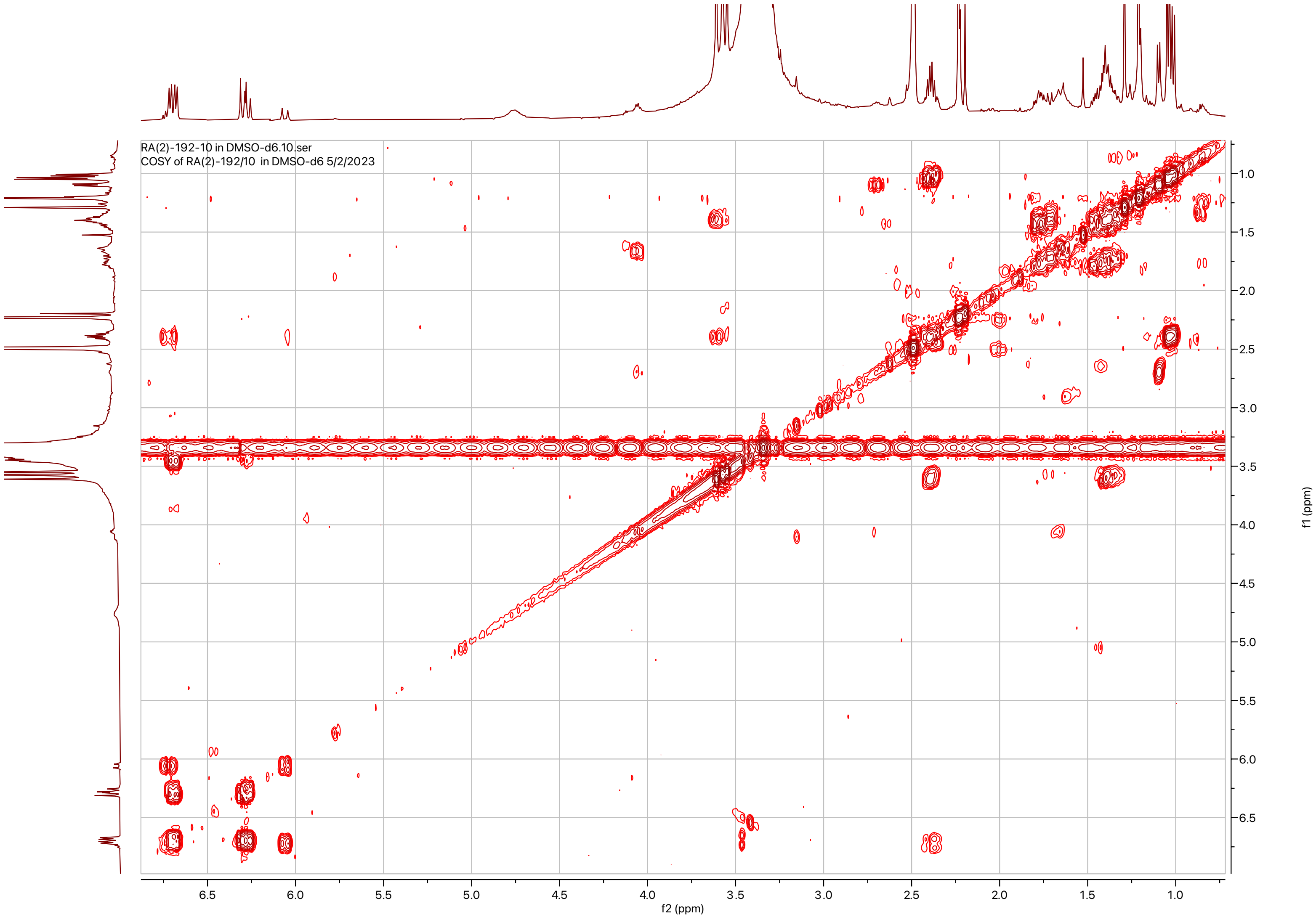

Figure S42. HR ESI MS of the mixture of Diacarnusin D (**4**) and the transformation products **4a** and **4b**

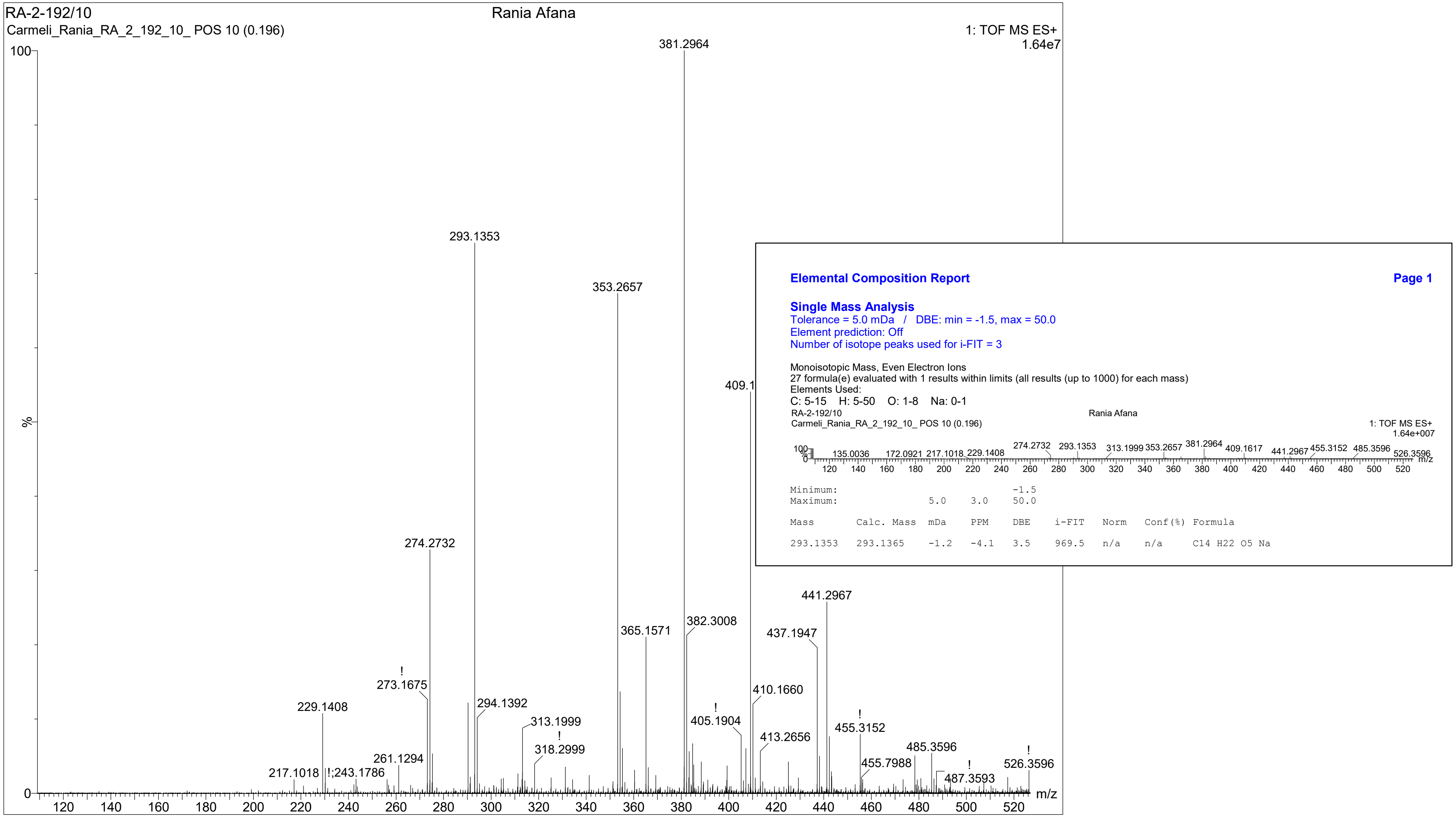

Elemental Composition Report — Page 1

Single Mass Analysis
Tolerance = 5.0 mDa / DBE: min = -1.5, max = 50.0
Element prediction: Off
Number of isotope peaks used for i-FIT = 3

Monoisotopic Mass, Even Electron Ions
27 formula(e) evaluated with 1 results within limits (all results (up to 1000) for each mass)
Elements Used:
C: 5-15 H: 5-50 O: 1-8 Na: 0-1

Minimum: -1.5
Maximum: 5.0 3.0 50.0

Mass	Calc. Mass	mDa	PPM	DBE	i-FIT	Norm	Conf(%)	Formula
293.1353	293.1365	-1.2	-4.1	3.5	969.5	n/a	n/a	C14 H22 O5 Na

Figure S43. HR ESI LCMS of the mixture of Diacarnusin D (**4**) and the transformation products **4a** and **4b**

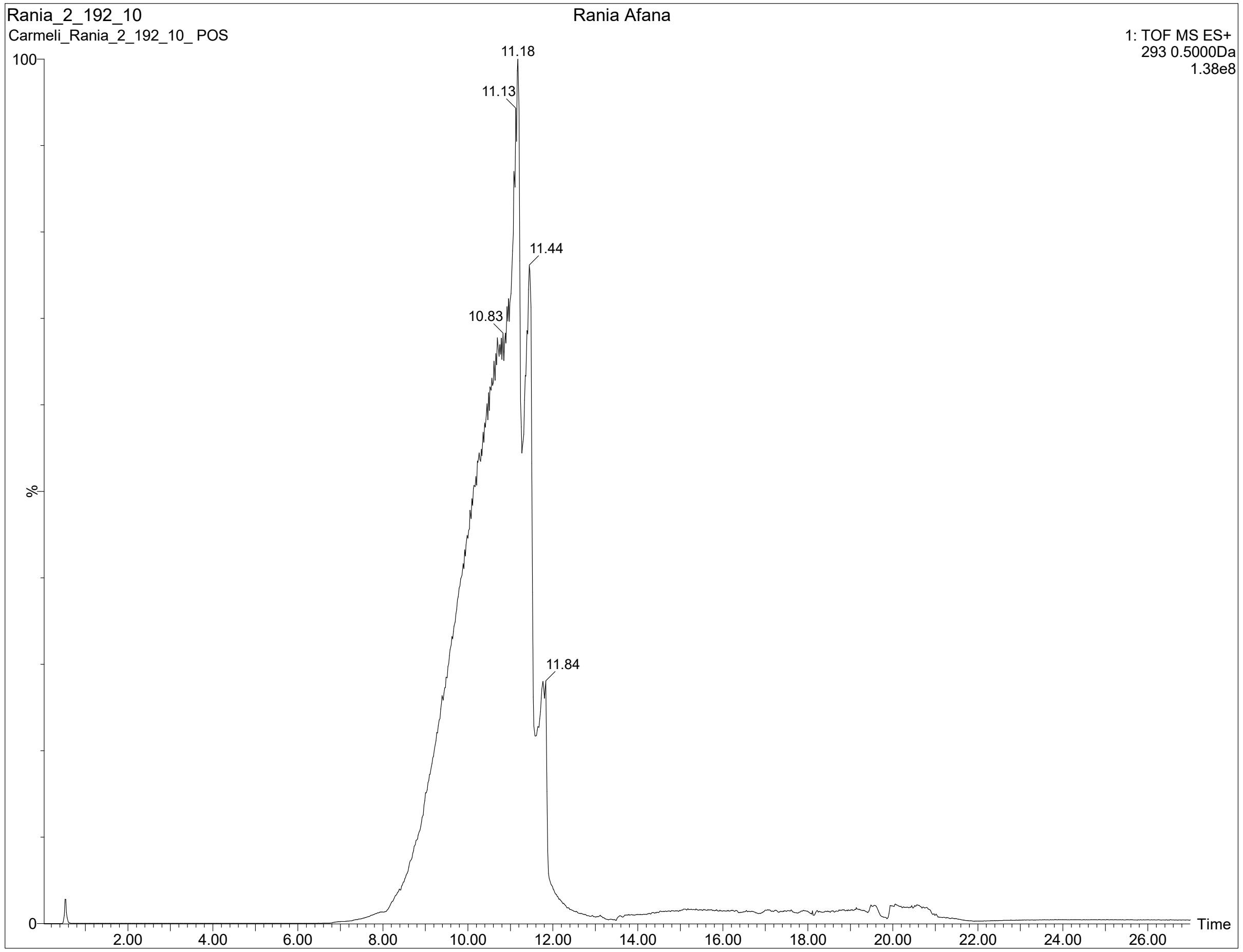

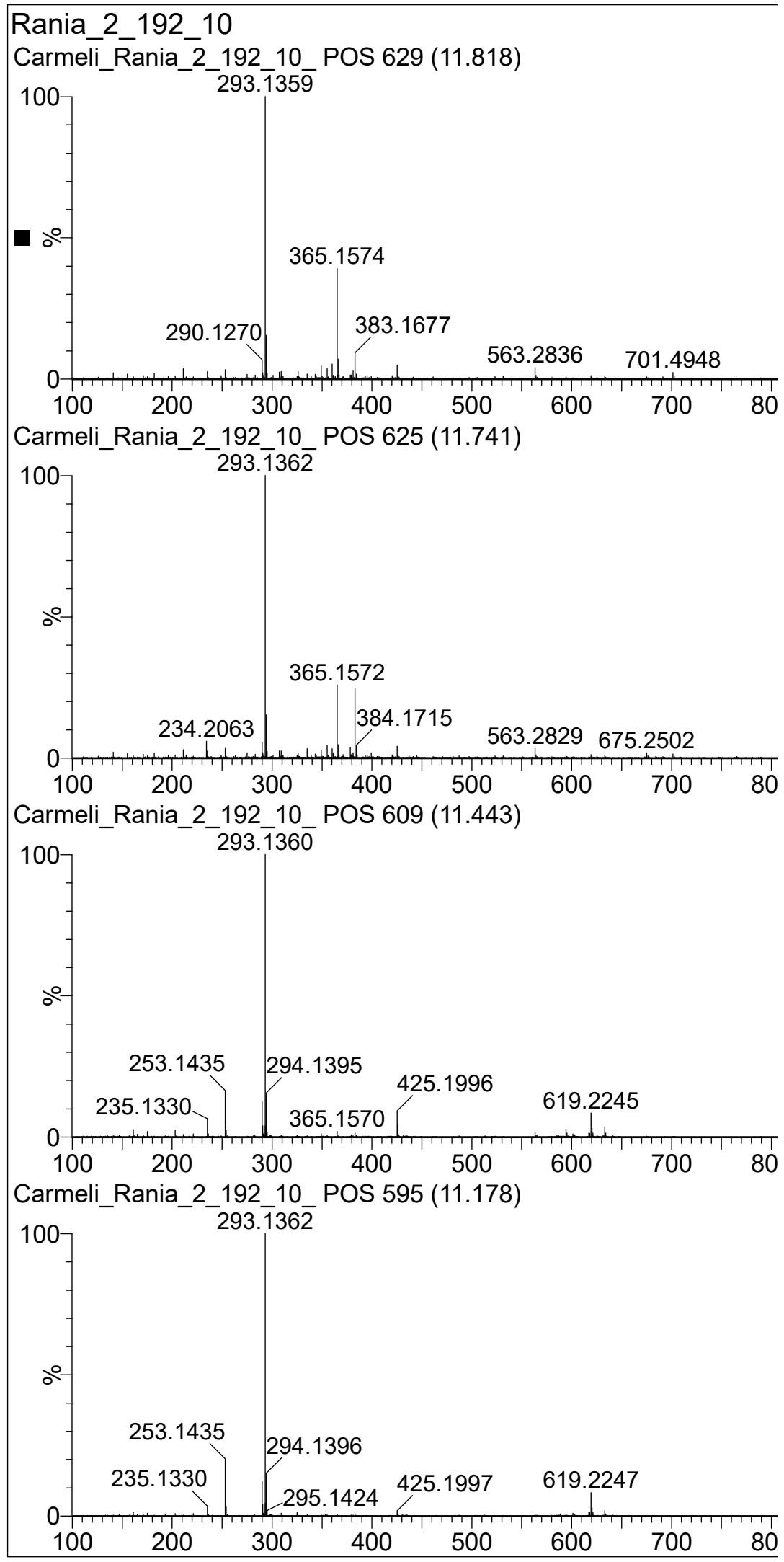

Table S7. NMR data of Diacarnusin D (**4**) in DMSO-d_6[a]

Position	δ_C, type[b]	δ_H, multiplicity, J in Hz	HMBC correlations[c]	COSY correlations
1	173.6, C		H_3-1,12	
2	41.9, CH_2	2.70, dq (6.1, 7.1)	H-3, H_3-12	H-3, H_3-12
3	80.9, CH	4.06, q (6.1)	H_2-5, H_3-12	H-2, H_2-4
4	22.5, CH_2	1.67, m	H_2-5	H-3, H_2-5
5	31.0, CH_2	1.64, m	H-3, H_3-13	H_2-4, H_3-13
6	79.7, C		H2-4,7, H_3-13	
7	41.5, CH_2	2.46, m	H-9, H_3-13	H-7',8,9
		2.37, dd (14.2, 7.7)		H-7,8,9
8	142.7, CH	6.72, dt (15.9, 7.5)	H_3-11	H-7,7',9
9	134.2, CH	6.06, dt (15.9, 1.2)	H-7', H_3-11	H-7,7',8
10	198.1, C		H-8,9, H_3-11	
11	27.0, CH_3	2.20, s	H-9	
12	12.8, CH_3	1.10, d (7.1)		
13	21.3, CH_3	1.20, s	H-2	H-2
1-OCH_3	51.9, CH_3	3.61, s		

[a]500 MHz for ^{1}H, 125 MHz for ^{13}C; [b]From HSQC; [c]HMBC correlations, optimized for J_{C-H}=8 Hz, are from carbon to the indicated proton(s).

Table S8a. NMR data of Diacarnusin D major product (**4a**) in DMSO-d_6[a]

Position	δ_C, multiplicity[b]	δ_H, multiplicity, J in Hz	HMBC correlations[c]	COSY correlations
1	175.1, C		1-OCH_3, H-2, H_3-12	
2	45.4, CH	2.39, m	H_2-4, H_3-12	H-3, H_3-12
3	70.8, CH	3.61, m	H-2,4,4',5, H_3-12	H-2,4
4	30.3, CH_2	1.40, m	H-2,5,5'	H-3
5	34.5, CH_2	1.78, m	H-3,4,4',7, H_3-12	H-4,4',5'
		1.44, m		H-4,4',5
6	64.0, C		H-4,4',5,5',7, H_3-13	
7	60.8, CH	3.45, d (6.8)	H-5,5',8,9, H_3-13	H-8
8	142.6, CH	6.69, dd (16.0, 6.8)	H-7, H_3-11	H-7,9
9	133.9, CH	6.30, d (16.0)	H-7,8, H_3-11	H-7,8, H_3-11
10	197.6, C		H-8,9, H_3-11	
11	27.2, CH_3	2.23, s	H-9	
12	11.8, CH_3	1.04, d (6.9)	H-2,3	H-2
13	16.6, CH_3	1.21, s	H-5,5'	
1-OCH_3	51.5, CH_3	3.57, s		

[a]500 MHz for ^{1}H, 125 MHz for ^{13}C; [b]From HSQC; [c]Optimized for $J_{C\text{-}H}$=8 Hz.

Table S8b. NMR data of Diacarnusin D minor product (**4b**) in DMSO-d_6[a]

Position	δ_C, multiplicity[b]	δ_H, multiplicity, *J* in Hz	HMBC correlations[c]	COSY correlations
1	175.1, C		1-OCH$_3$, H-2, H$_3$-12	
2	45.1, CH	2.38, m	H$_3$-12	H-3, H$_3$-12
3	71.1, CH	3.57, m	H-2, H-5', H$_3$-12	H-2, H$_2$-4
4	34.5, CH$_2$	1.45, m	H-2, H-5'	H-3,4', H$_2$-5
		1.34, m		H-3,4,5
5	29.1, CH$_2$	1.72, m	H-2, H-4, H$_3$-13	H-5', H$_2$-4,
		1.37, m		H-5'
6	64.3, C		H-5,7,8,9, H$_2$-4, H$_3$-13	
7	61.8, CH	3.44, d (6.8)	H-8,9, H$_2$-5, H$_3$-13	H-8
8	142.5, CH	6.70, dd (16.0, 6.8)	H-7, H$_3$-11	H-7,9
9	133.8, CH	6.27, d (16.0)	H-7,8, H$_3$-11	H-7,9, H$_3$-11
10	197.6, C		H-8,9, H$_3$-11	
11	27.1, CH$_3$	2.22, s	H-9	
12	11.8, CH$_3$	1.01, d (7.0)	H-2	H-2
13	22.0, CH$_3$	1.29, s	H-5,7	
1-OCH$_3$	51.5, CH$_3$	3.55, s		

[a]500 MHz for ^{1}H, 125 MHz for ^{13}C; [b]From HSQC; [c]Optimized for $J_{C\text{-}H}$=8 Hz.

Figure S44. ^{1}H NMR (500 MHz, $CDCl_3$) spectrum of the mixture of Diacarnusins E (**5**) and F (**6**)

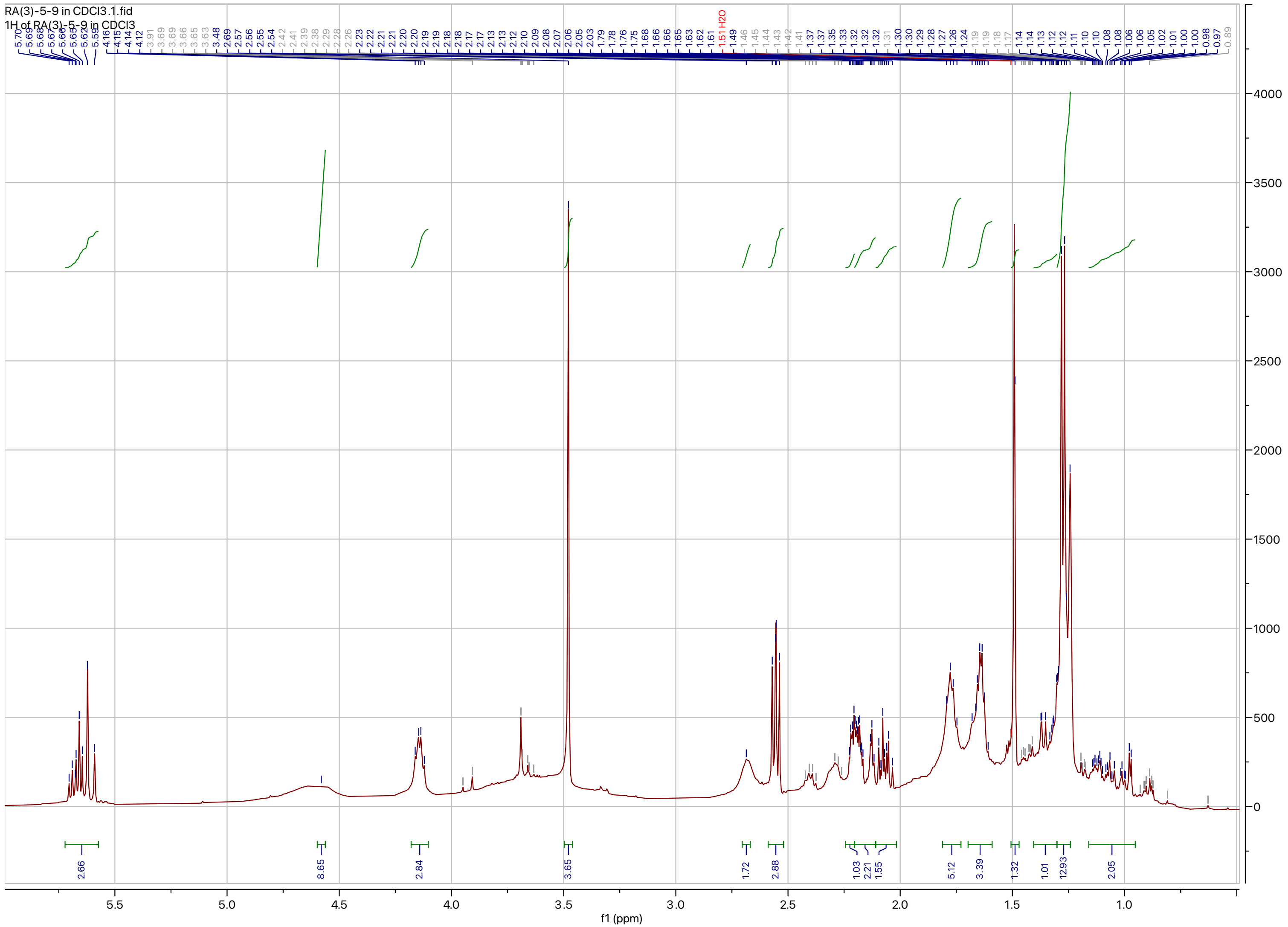

Figure S45. ^{13}C NMR (125 MHz, $CDCl_3$) spectrum of the mixture of Diacarnusins E (**5**) and F (**6**)

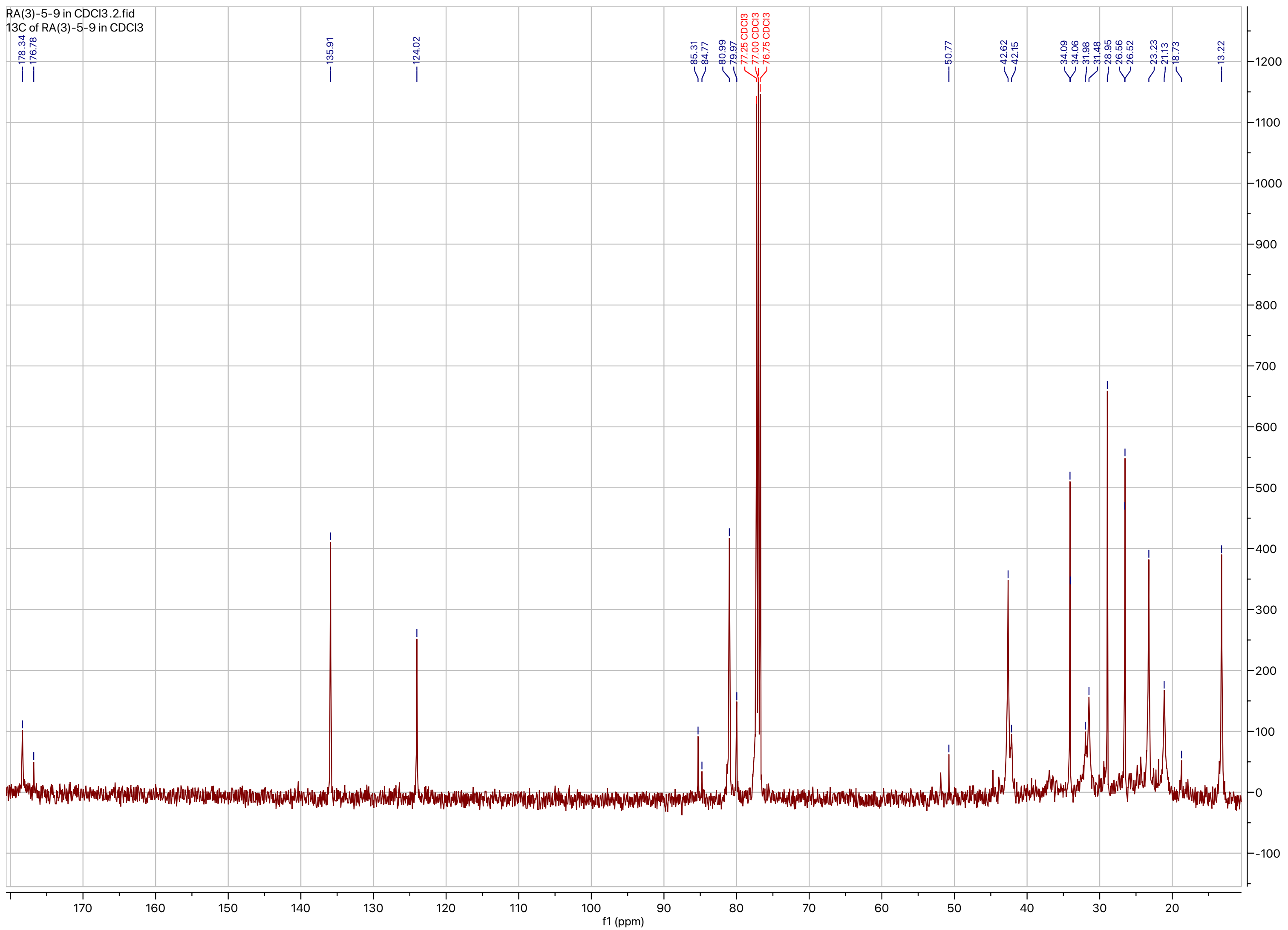

Figure S46. HSQC (500/125 MHz, $CDCl_3$) 2D spectrum of the mixture of Diacarnusins E (**5**) and F (**6**)

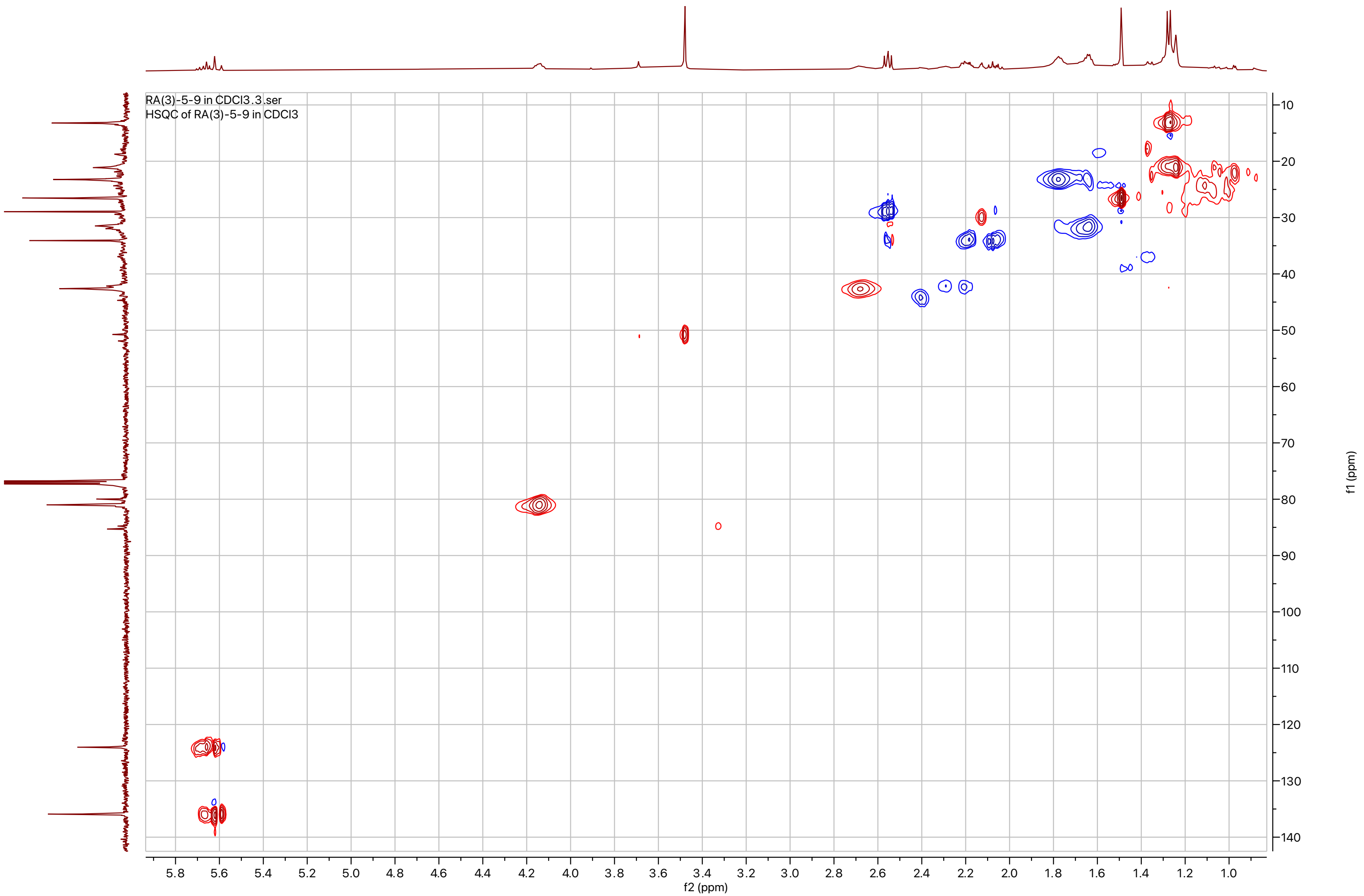

Figure S47. HMBC (500/125 MHz, $CDCl_3$) 2D spectrum of the mixture of Diacarnusins E (**5**) and F (**6**)

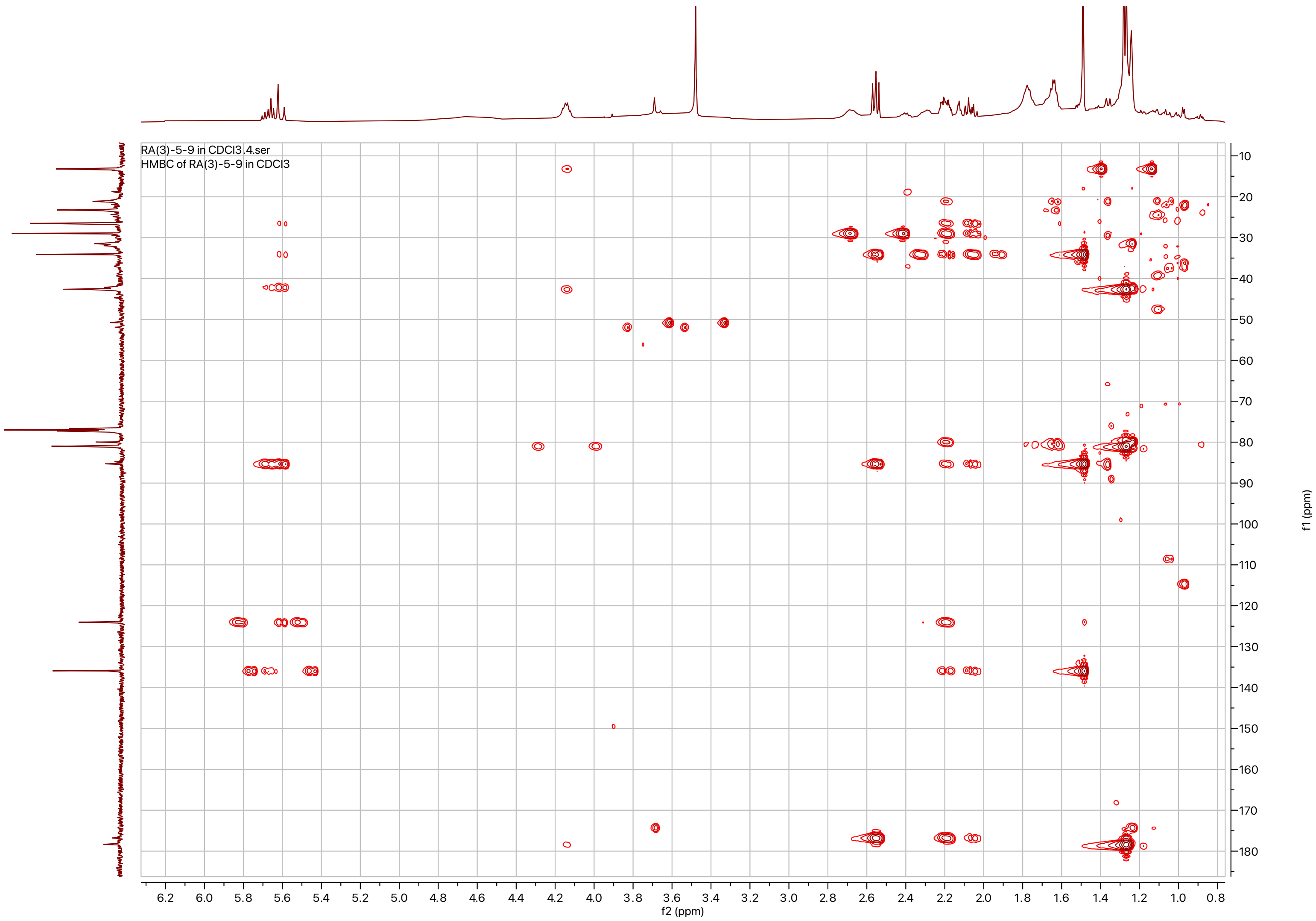

Figure S48. COSY (500 MHz, $CDCl_3$) 2D spectrum of the mixture of Diacarnusins E (**5**) and F (**6**)

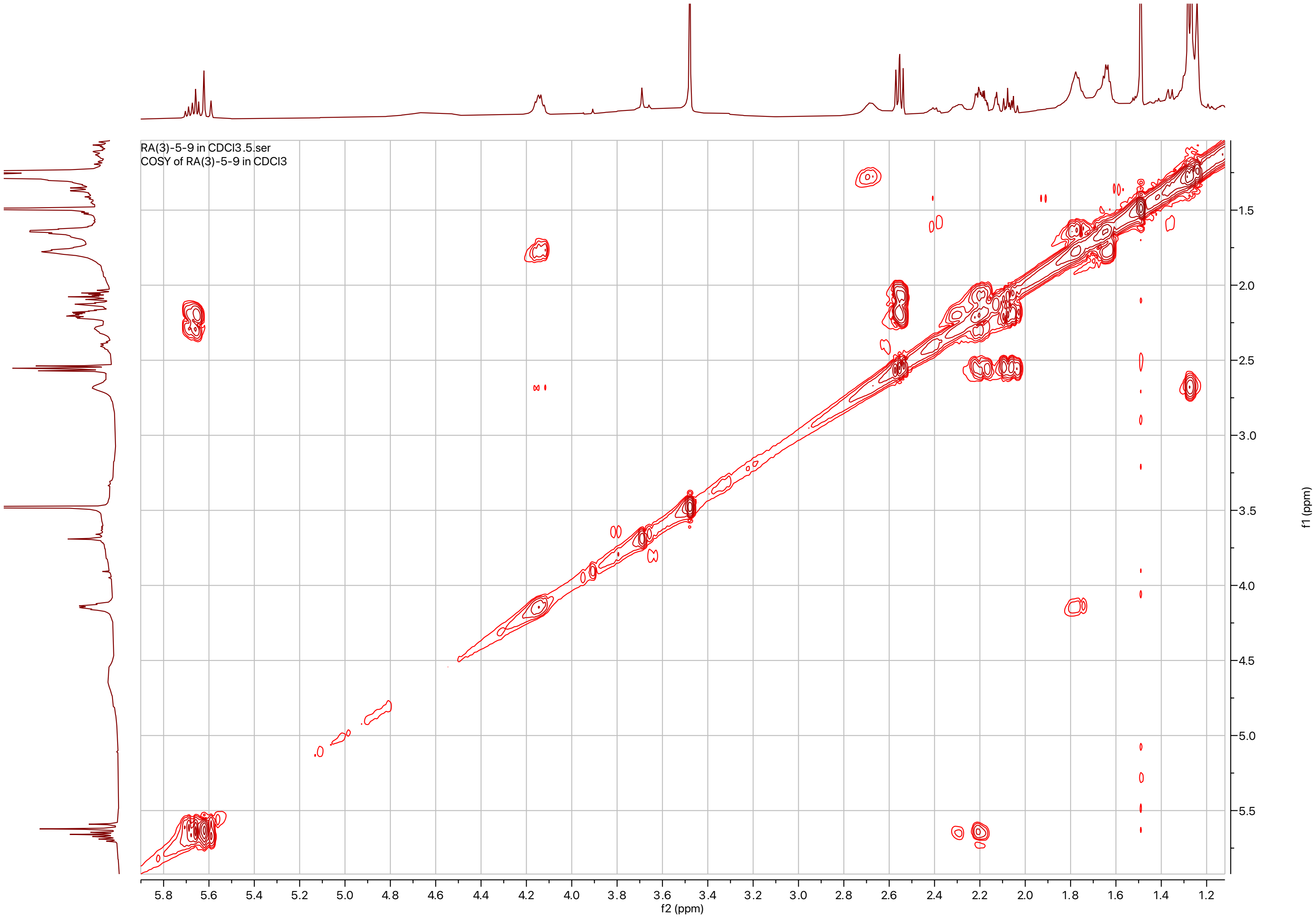

Figure S49. HR ESI MS of the mixture of Diacarnusins E (**5**) and F (**6**)

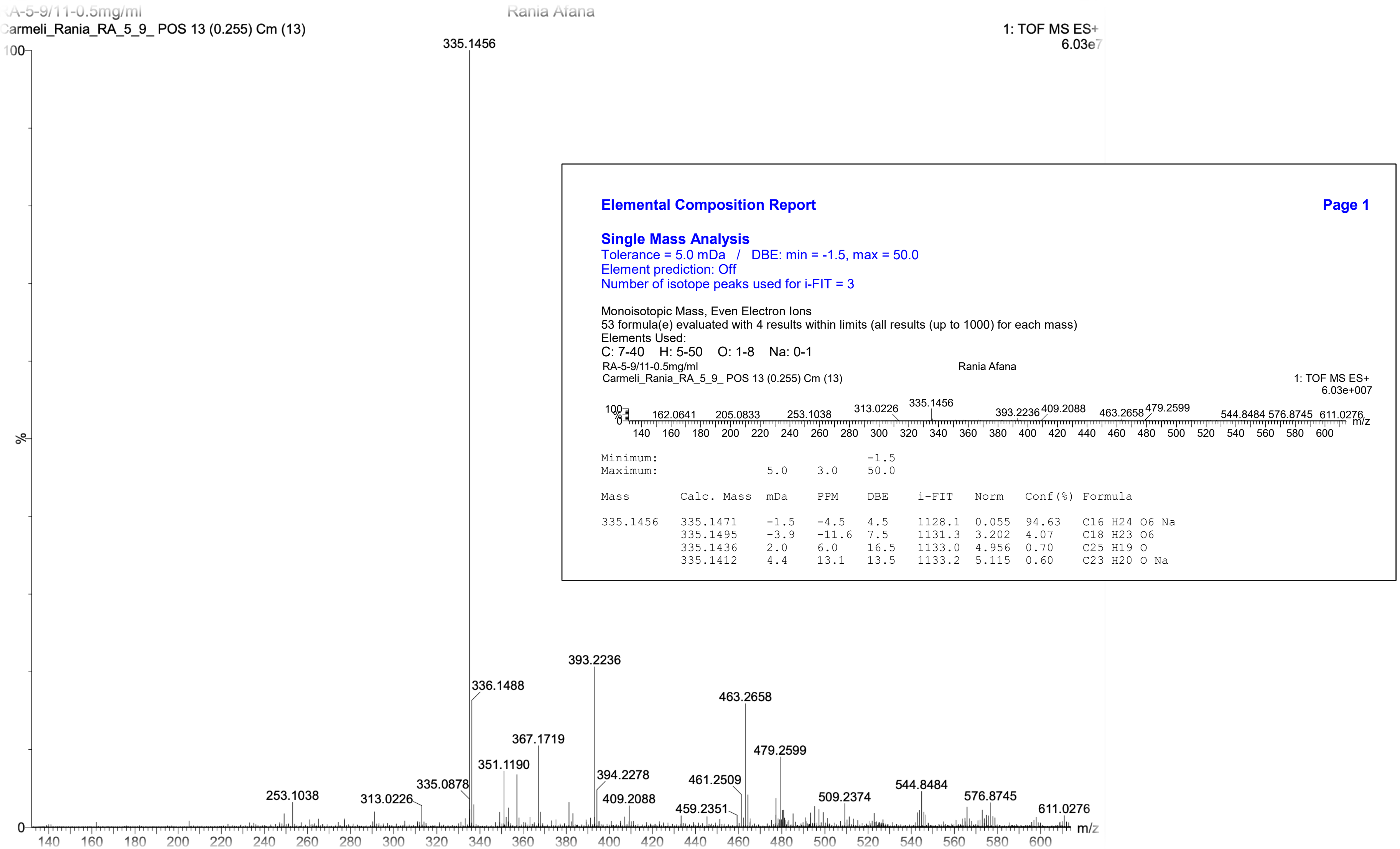

Elemental Composition Report **Page 1**

Single Mass Analysis
Tolerance = 5.0 mDa / DBE: min = -1.5, max = 50.0
Element prediction: Off
Number of isotope peaks used for i-FIT = 3

Monoisotopic Mass, Even Electron Ions
53 formula(e) evaluated with 4 results within limits (all results (up to 1000) for each mass)
Elements Used:
C: 7-40 H: 5-50 O: 1-8 Na: 0-1

Mass	Calc. Mass	mDa	PPM	DBE	i-FIT	Norm	Conf(%)	Formula
Minimum:				-1.5				
Maximum:		5.0	3.0	50.0				
335.1456	335.1471	-1.5	-4.5	4.5	1128.1	0.055	94.63	C16 H24 O6 Na
	335.1495	-3.9	-11.6	7.5	1131.3	3.202	4.07	C18 H23 O6
	335.1436	2.0	6.0	16.5	1133.0	4.956	0.70	C25 H19 O
	335.1412	4.4	13.1	13.5	1133.2	5.115	0.60	C23 H20 O Na

Table 5. NMR data of diacarnusins E (**5**) and F (**6**) in $CDCl_3$[a]

Position	δ_C, type[b]	δ_H, multiplicity, *J* in Hz	HMBC correlations[c]	COSY correlations
1	178.3, C		H-3, H_3-14	
2	42.6, CH	2.68, brm	H-3, H_3-14	H-3, H_3-14
3	81.0, CH	4.14, brdt (5.2, 7.8)	H_2-5, H_3-14	H-2, H_2-4
4	23.2, CH_2	1.77, m	H-3, H_2-5	H-3, H_2-5
5	31.5, CH_2	1.64, m	H_2-4, H-7’, H_3-15	H_2-4
6	80.0, C		H_2-5, H-7’, H_3-15	
7	42.2, CH_2	2.30, m	H-8, H-9	H-7’, H-8
7’		2.20, m		H-7, H-8
8	124.0, CH	5.67, dt (15.8, 6.9)	H-7’,9, H_3-16	H_2-7, H-9
9	135.9, CH	5.61, brd (15.8)	H-8, H_2-7,11, H_3-16	H-8
10	85.3, C (2)		H-8,9, H_2-11,12, H_3-16	
	84.8, C (1)			
11	34.09, CH_2 34.07, CH_2	2.18, m	H-9, H_3-16	H-11’, H_2-12
11’		2.07, dt (12.8, 8.6)		H-11, H_2-12
12	29.0, CH_2	2.55, dd (7.8, 8.6)	H_2-11	H_2-11
13	176.8, C		H_2-11,12	
14	13.2, CH_3	1.27, d (7.2)	H-3	H-2
15	21.1, CH_3	1.24, brs	H_2-5, H-7, H-7’	
16	26.52, CH_3	1.491, s (2)	H-9, H_2-11	
	26.56, CH_3	1.488, s (1)		

[a]500 MHz for ^{1}H, 125 MHz for ^{13}C; [b]From HSQC; [c]HMBC correlations, optimized for J_{C-H}=8 Hz, are from carbon to the indicated proton(s).

Figure S50. ^{1}H NMR (500 MHz, $CDCl_3$) spectrum of Sigmosceptrellin B (**7**)

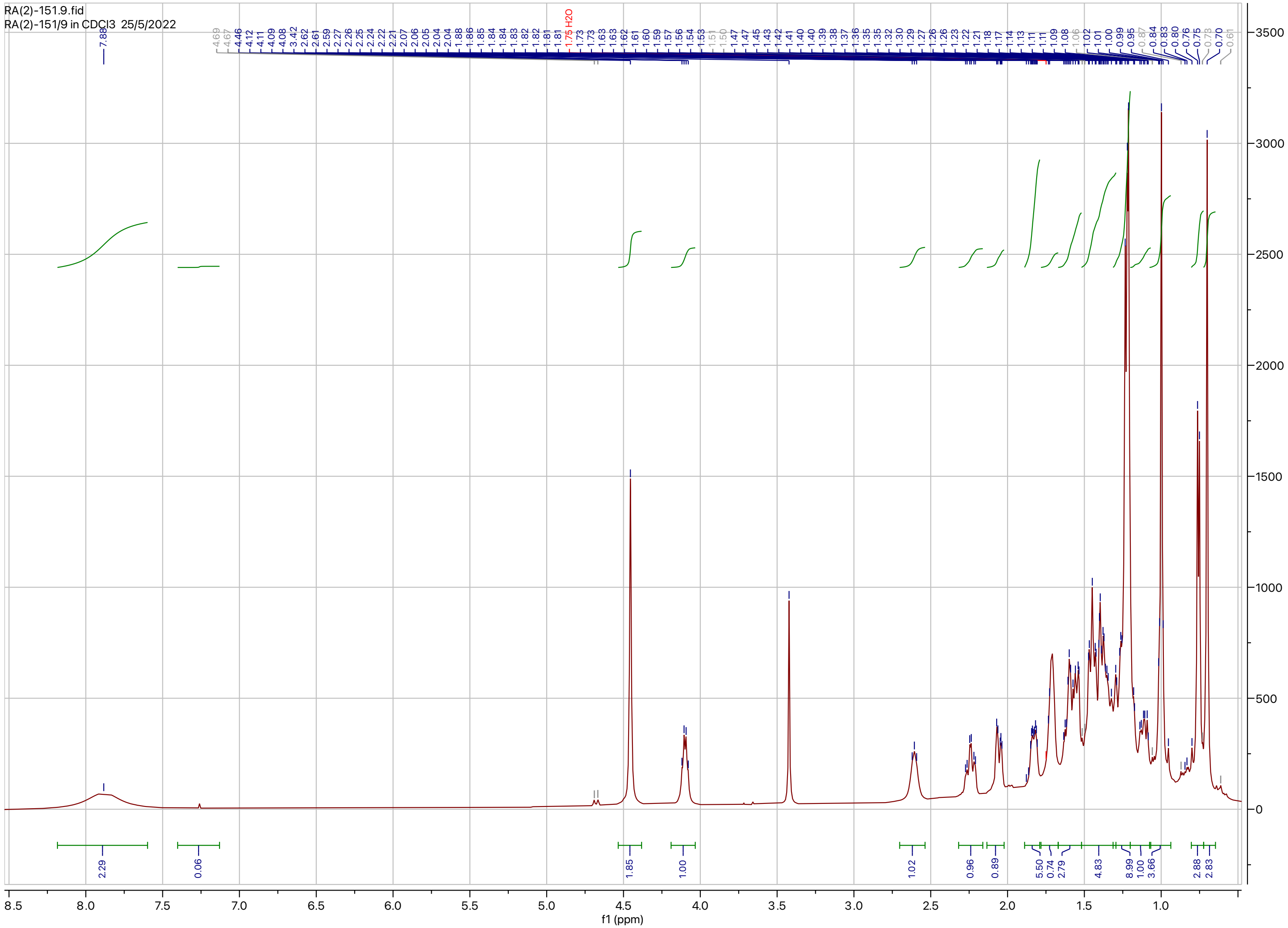

Figure S51. ^{1}H NMR (500 MHz, DMSO-d_6) spectrum of Sigmosceptrellin B (**7**)

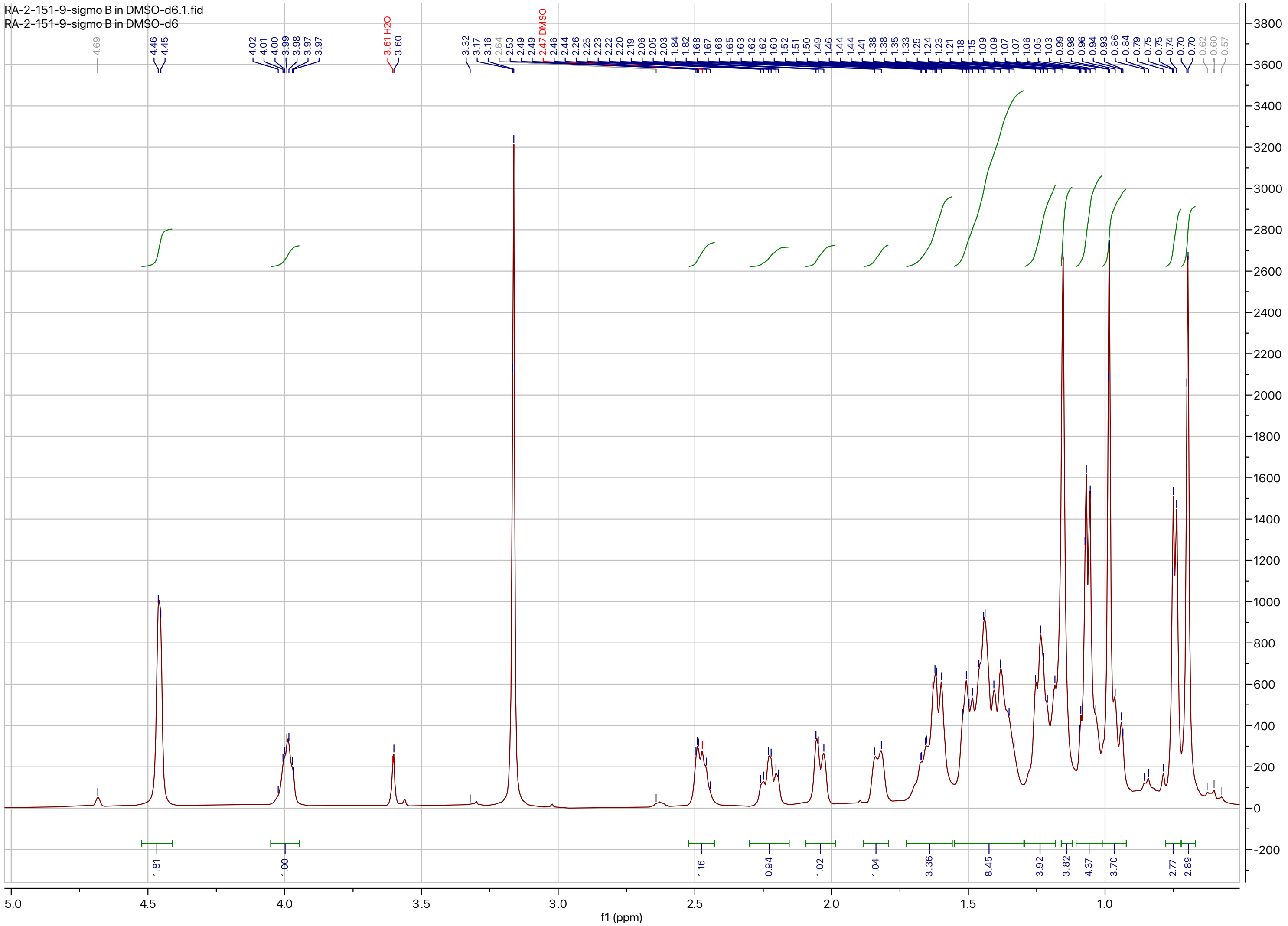

Figure S52. ^{13}C NMR (125 MHz, $CDCl_3$) spectrum of Sigmosceptrellin B (**7**)

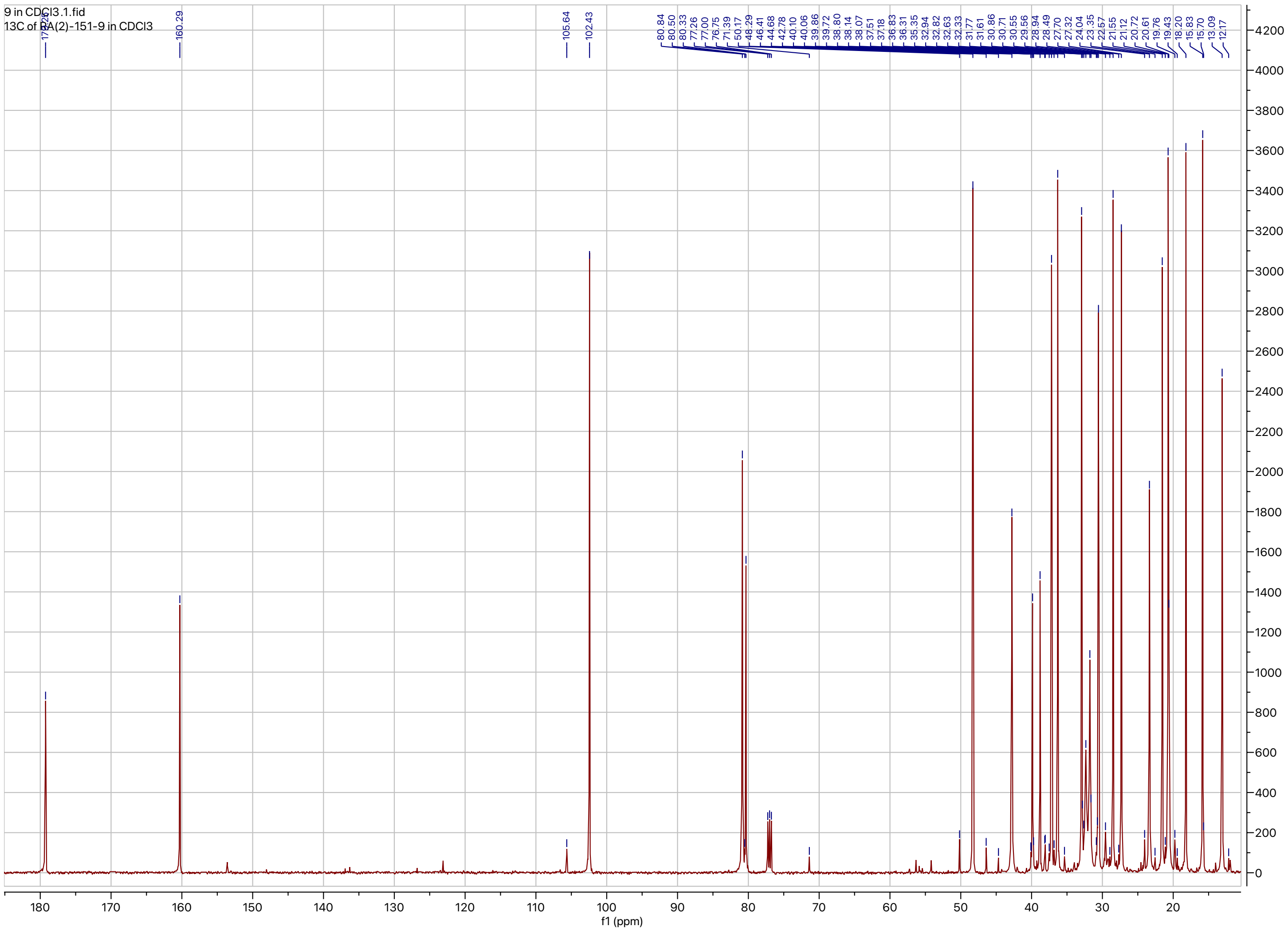

Figure S53. ^{13}C NMR (125 MHz, DMSO-d_6) spectrum of Sigmosceptrellin B (**7**)

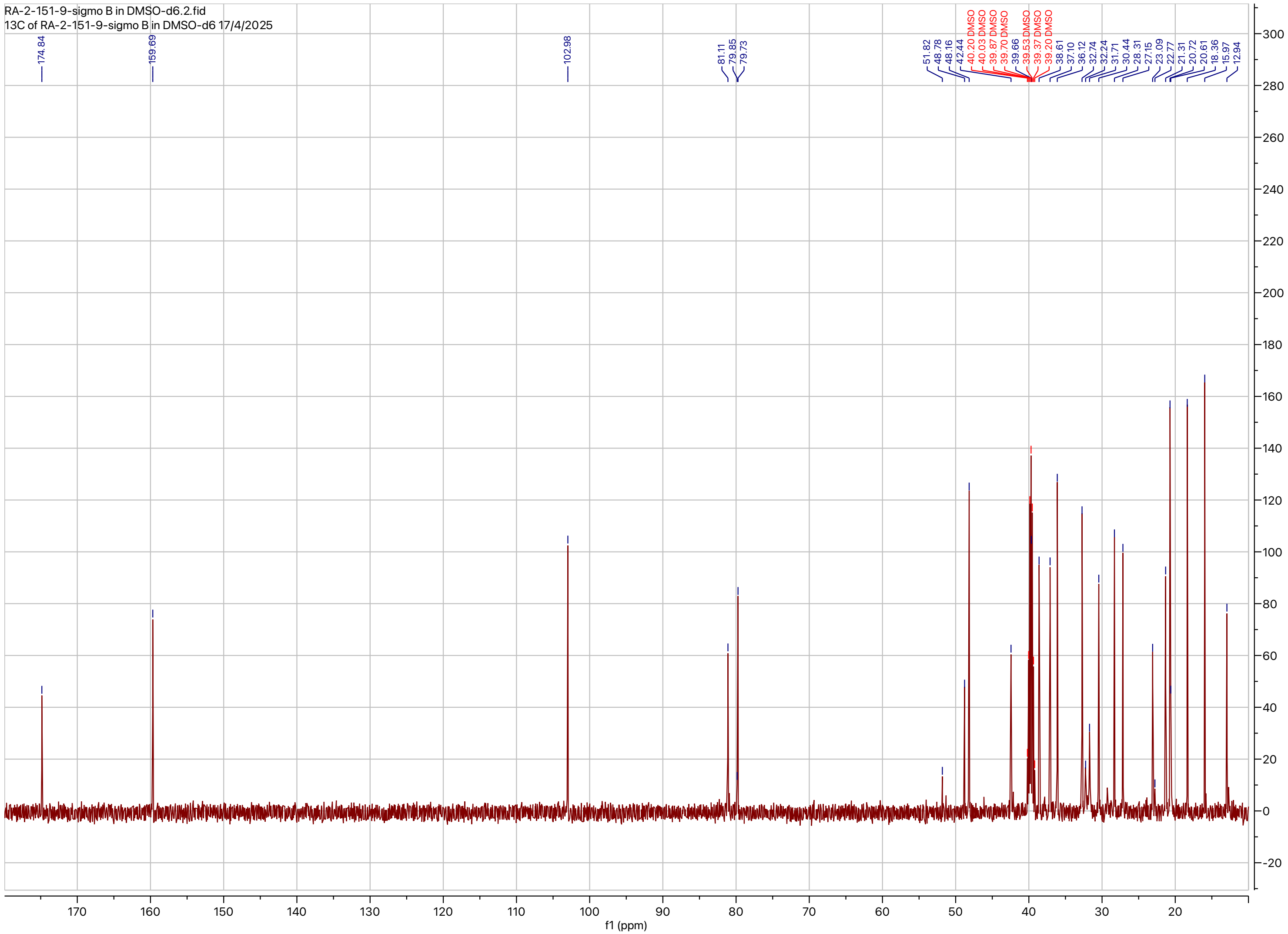

Figure S54. HSQC (500/125 MHz, $CDCl_3$) 2D spectrum of Sigmosceptrellin B (**7**)

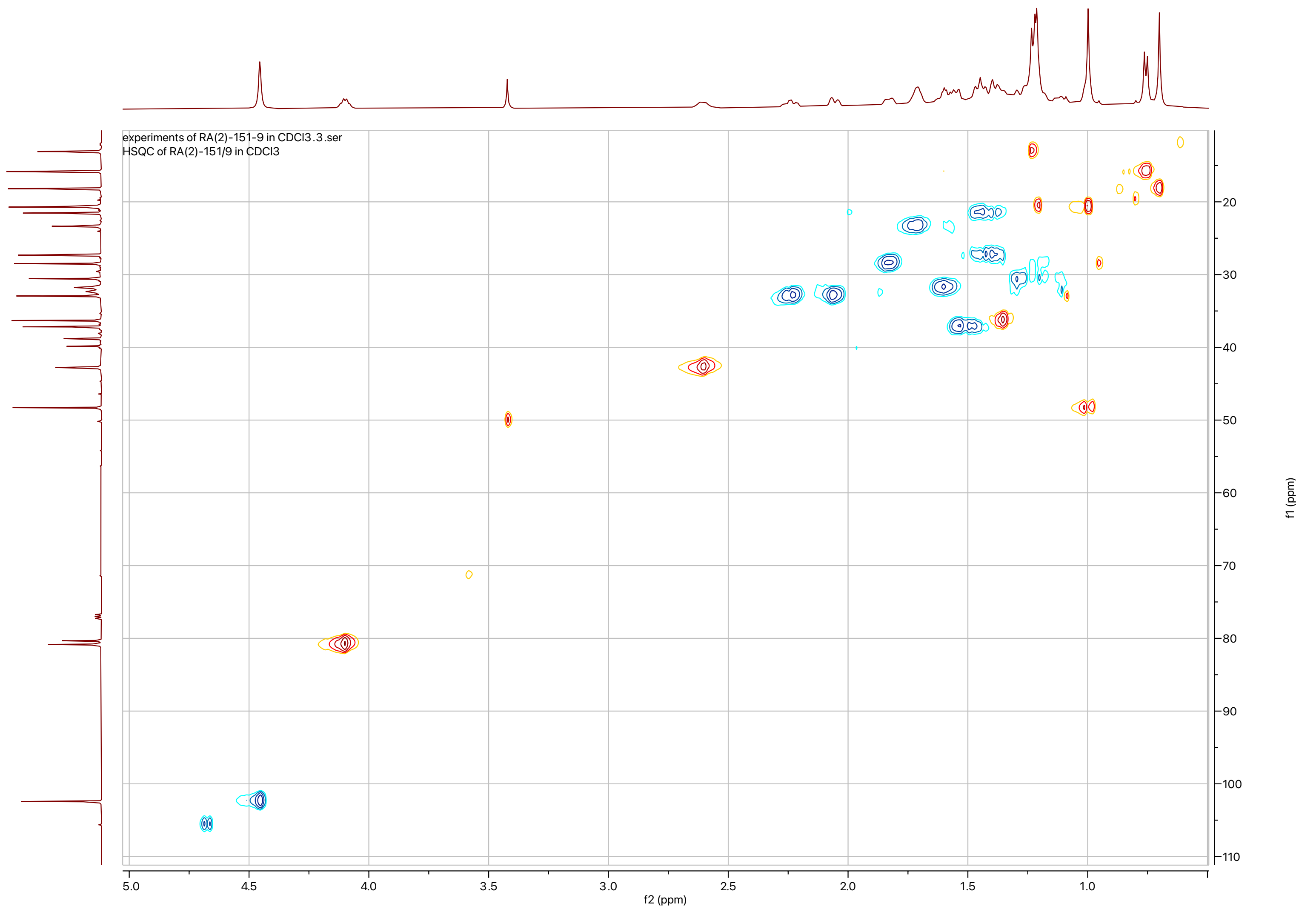

Figure S55. HSQC (500/125 MHz, DMSO-d_6) 2D spectrum of Sigmosceptrellin B (**7**)

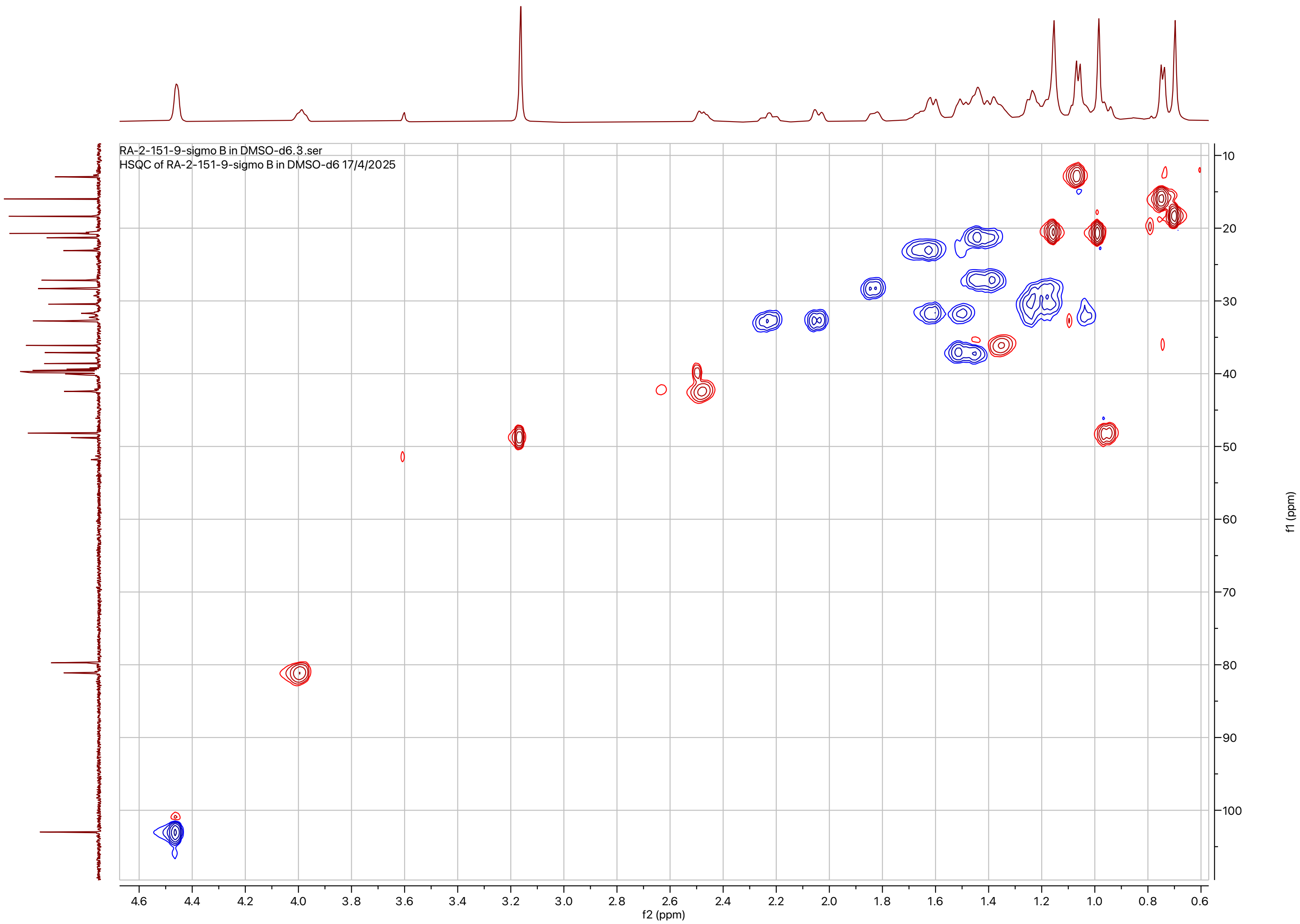

Figure S56. HMBC (500/125 MHz, $CDCl_3$) 2D spectrum of Sigmosceptrellin B (**7**)

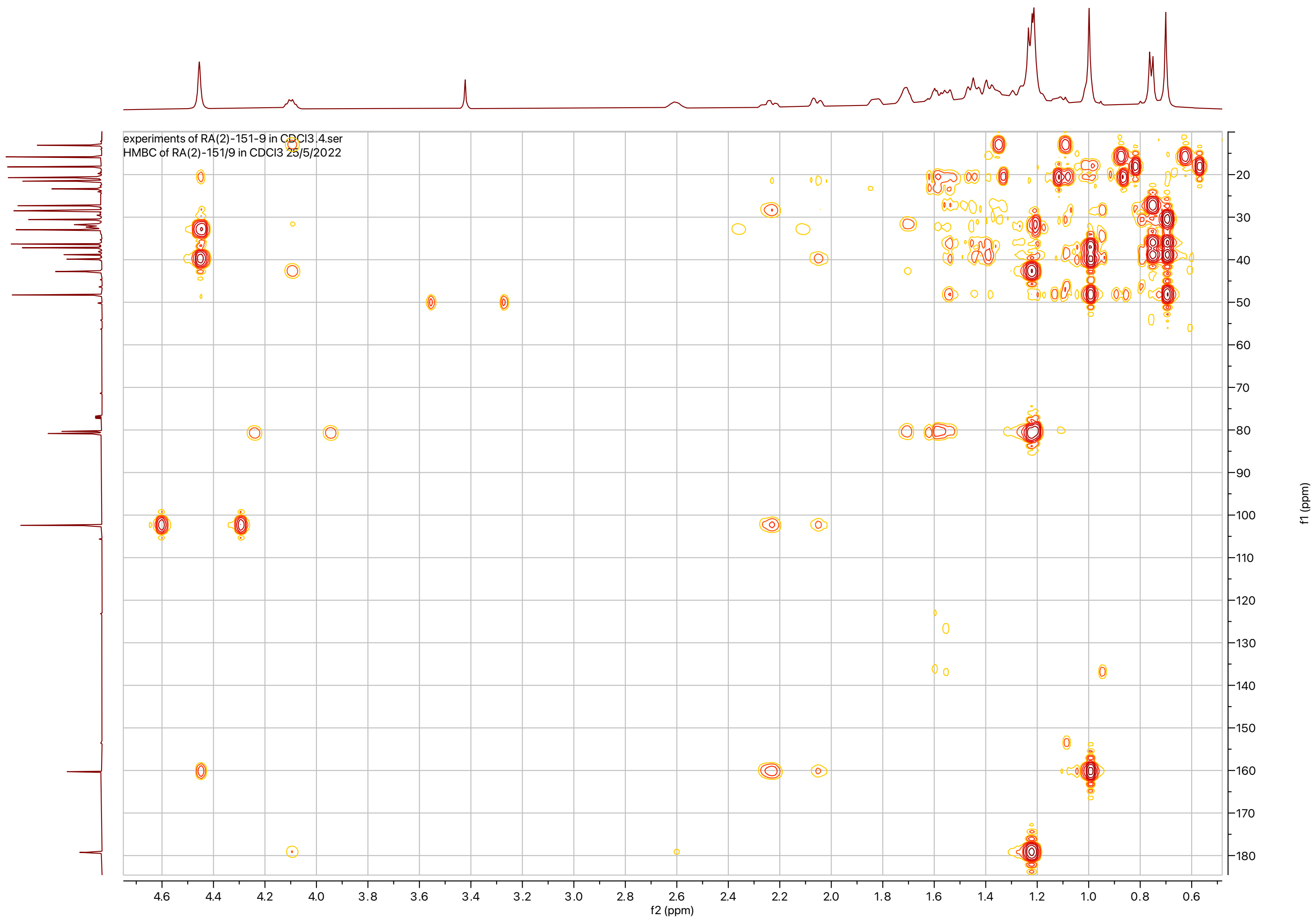

Figure S57. COSY (500 MHz, $CDCl_3$) 2D spectrum of Sigmosceptrellin B (**7**)

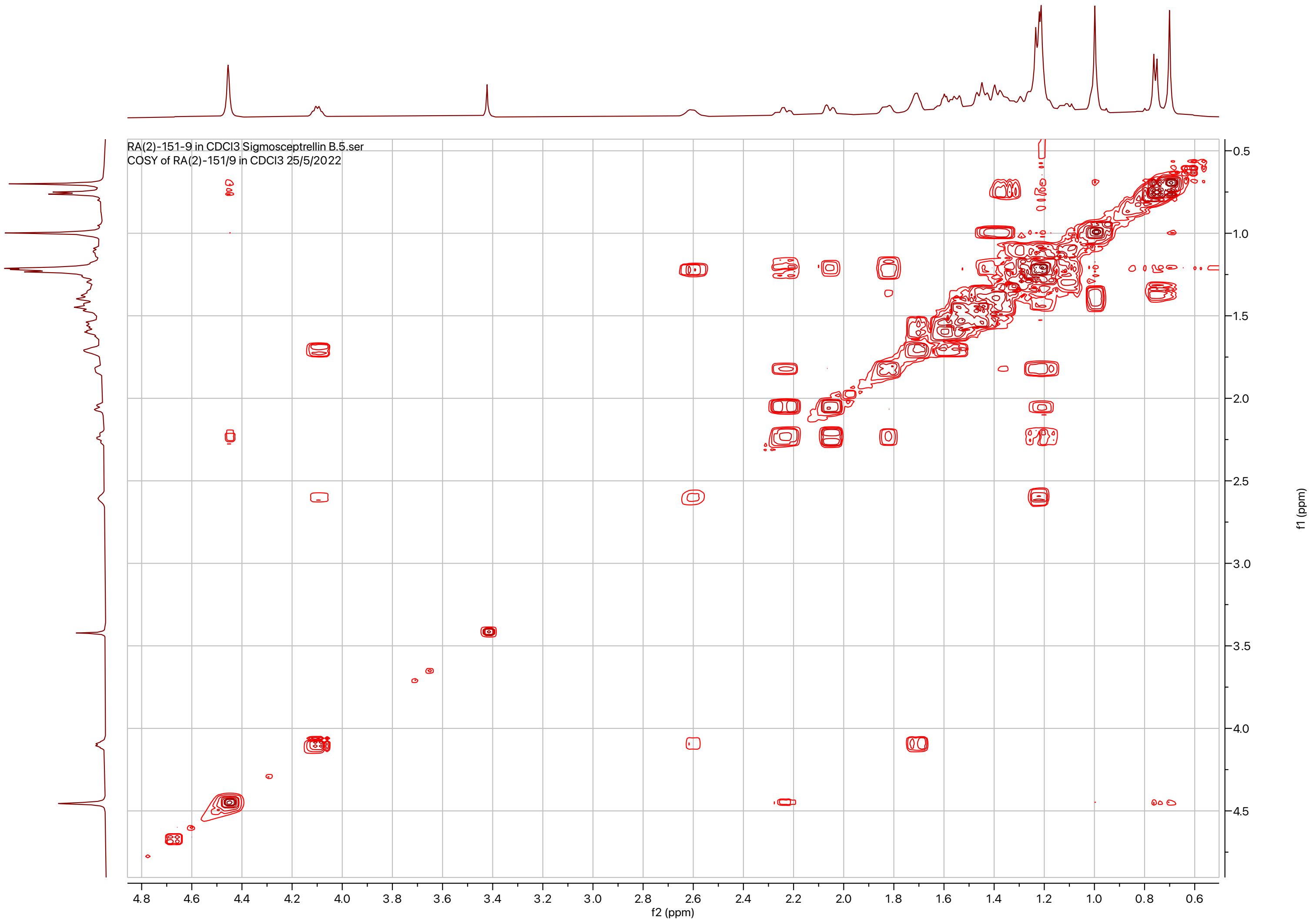

Figure S58. COSY (500 MHz, DMSO-d_6) 2D spectrum of Sigmosceptrellin B (**7**)

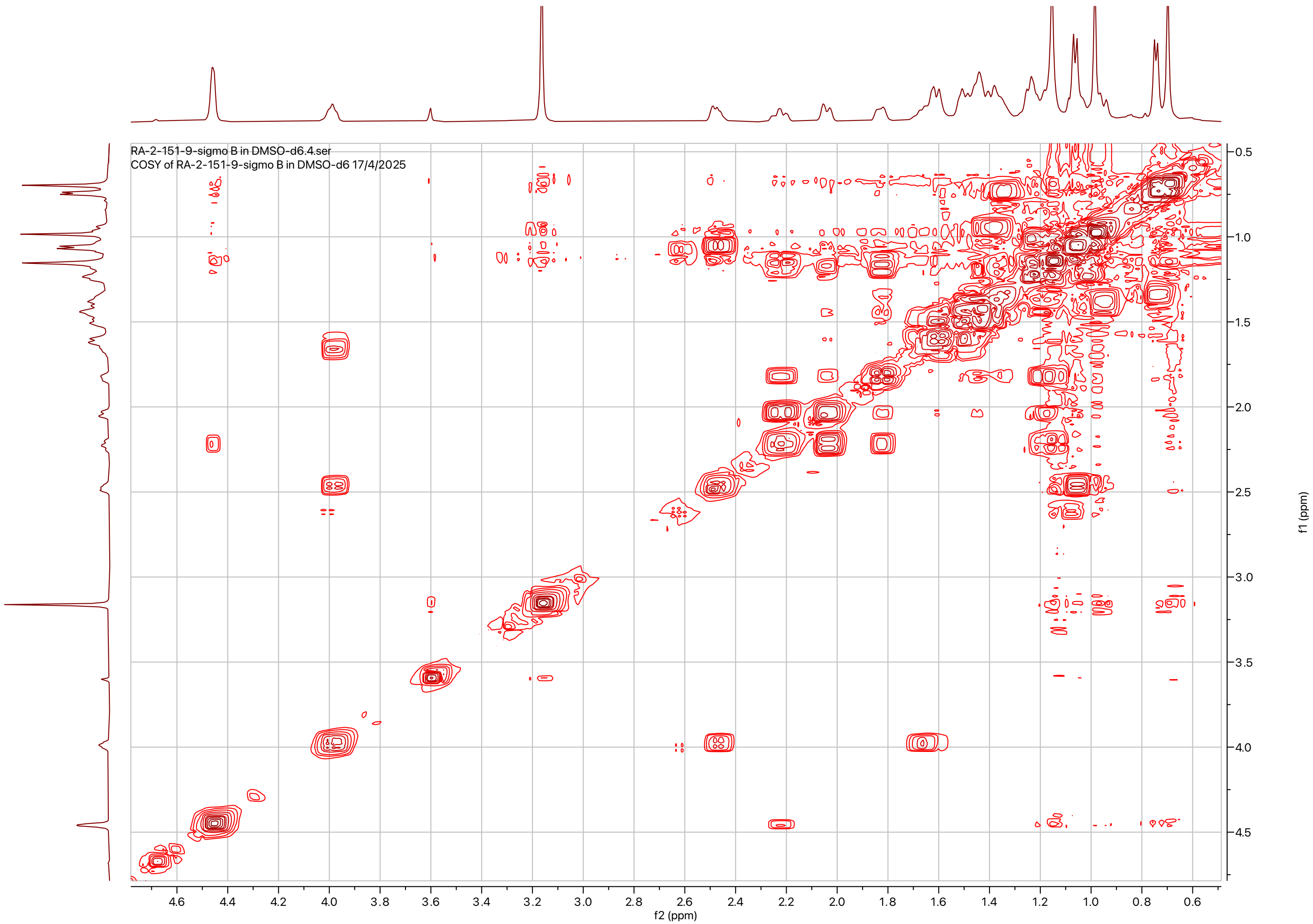

Figure S59. HR ESI MS of Sigmosceptrellin B (**7**)

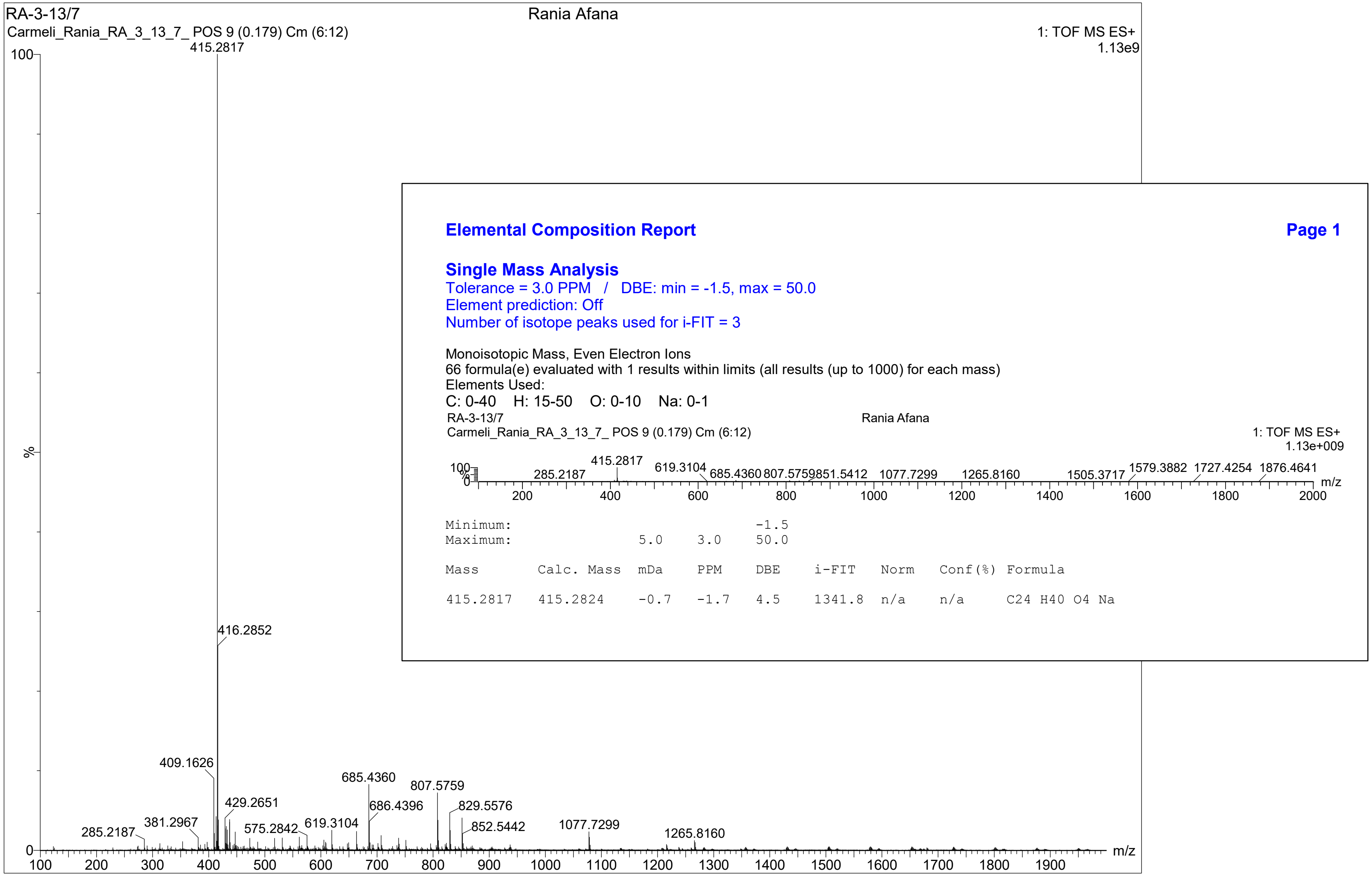

Table S10a. NMR data of Sigmosceptrellin B (**7**) in $CDCl_3$[a]

Position	δ_C, multiplicity[b]	δ_H, multiplicity, *J* in Hz	HMBC correlations[c]	COSY correlations
1	179.3, C		H-2,3, H_3-19	
2	42.8, CH	2.61, m	H-3, H_2-4, H_3-19	H-3, H_3-19
3	80.8, CH	4.10, brq	H-2,5 H_2-4, H_3-19	H-2, H_2-4
4	23.4, CH_2	1.71, brm	H-5'	H-3, H_2-5
5	31.8, CH_2	1.61, m	H-7', H_2-4, H_3-20	H-5', H_2-4
		1.58, m		H-5, H_2-4
6	80.3, C		H-7, H_2-4,5, H_3-20	
7	32.3, CH_2	1.29, m	H-5', H_3-20	
		1.21, m		
8	30.6, CH_2	1.28, m	H-10, H_3-21	
		1.20, m		
9	38.8, C		H-8,11', H_3-21,22	
10	36.3, CH	1.36, m	H-8,11, H_2-12, H_3-21,22	H_3-22
11	27.3, CH_2	1.43, m	H-10, H_2-12, H_3-22	H-18
		1.39, m		H-18
12	37.2, CH_2	1.54, m	H-18, H_2-12	
		1.46, m		
13	39.9, C		H-18, H_2-11,12,15,24	
14	160.3, C		H-18, H_2-15,24, 1.11	
15	32.9, CH_2	2.24, dt	H_2-24, H-16', H_3-21	H-15', H_2-16,24
		2.06, brd		H-15, H_2-16
16	28.5, CH_2	1.83, brd	H_2-15,17	H-16', H_2-15,17
		1.22, brd		H-16, 17', H_2-15
17	21.6, CH_2	1.45, t	H_2-15	
		1.39, brd		
18	48.3, CH	1.00, d	H_2-12,17,24, H_3-21	H_3-17
19	13.1, CH_3	1.23, d	H-3	H-2
20	20.6, CH_3	1.21, s	H-5'	
21	18.2, CH_3	0.70, s	H-18	

22	15.8, CH_3	0.76, d	H_2-17	H-10
23	20.7, CH_3	1.00, s	H-18, H_2-12,24	
24	102.4, CH_2	4.45, s	H_2-15	H-15

[a]500 MHz for ^{1}H, 125 MHz for ^{13}C; [b]From HSQC; [c]Optimized for $J_{C\text{-}H}$=8 Hz.

Table S10b. NMR data of Sigmospterellin B (**7**) in DMSO-d_6[a]

Position	δ_C, multiplicity[b]	δ_H, multiplicity	COSY correlations
1	174.8, C		
2	42.4, CH	2.47, brt	H-3, H_3-19
3	81.1, CH	3.99, dt	H-2, H_2-4
4	23.1, CH_2	1.66, m	H-3,4’,5’
		1.62, m	H-3,4,5’
5	31.7, CH_2	1.61, m	H-4,5’
		1.49, m	H-4,5
6	79.7, C		
7	32.2, CH_2	1.24, m	H-7’,8’
		1.04, m	H-7
8	30.4, CH_2	1.24, m	H-8’
		1.18, m	H-7,8
9	38.6, C		
10	36.1, CH	1.34, m	H-11
11	27.2, CH_2	1.44, m	H-10,11’
		1.39, m	H-11
12	37.1, CH_2	1.50, m	H-12’
		1.45, m	H-12
13	39.7, C		
14	159.7, C		
15	32.7, CH_2	2.22, dt	H-15’, H_2-16,24
		2.04, brd	H-15, H_2-16
16	28.3, CH_2	1.83, brd	H-15,16’,17
		1.16, m	H-15,16,17’
17	21.3, CH_2	1.44, m	H-16,17’
		1.39, m	H-16’,17
18	48.2, CH	0.95, brd	
19	12.9, CH_3	1.06, d	H-2
20	20.6, CH_3	1.15, s	

21	18.4, CH_3	0.70, s	
22	16.0, CH_3	0.74, d	H-10
23	20.7, CH_3	0.98, s	
24	103.0, CH_2	4.46, s	H-15

[a]500 MHz for ^{1}H, 125 MHz for ^{13}C; [b]From HSQC.

Figure S60. ^{1}H NMR (500 MHz, $CDCl_3$) spectrum of Sigmosceptrellin B Methyl Ester (**8**)

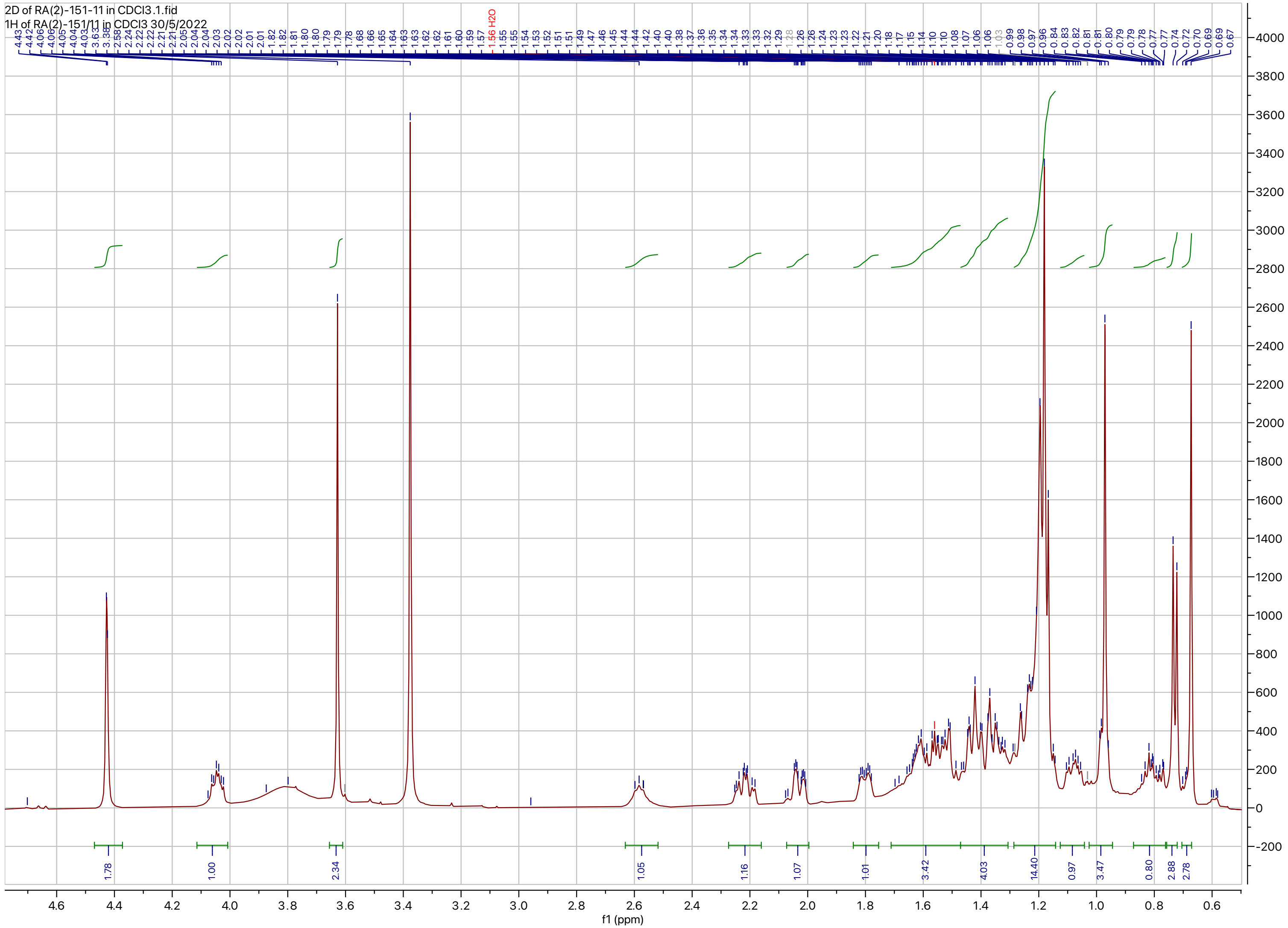

Figure S61. ^{13}C NMR (125 MHz, $CDCl_3$) spectrum of Sigmosceptrellin B Methyl Ester (**8**)

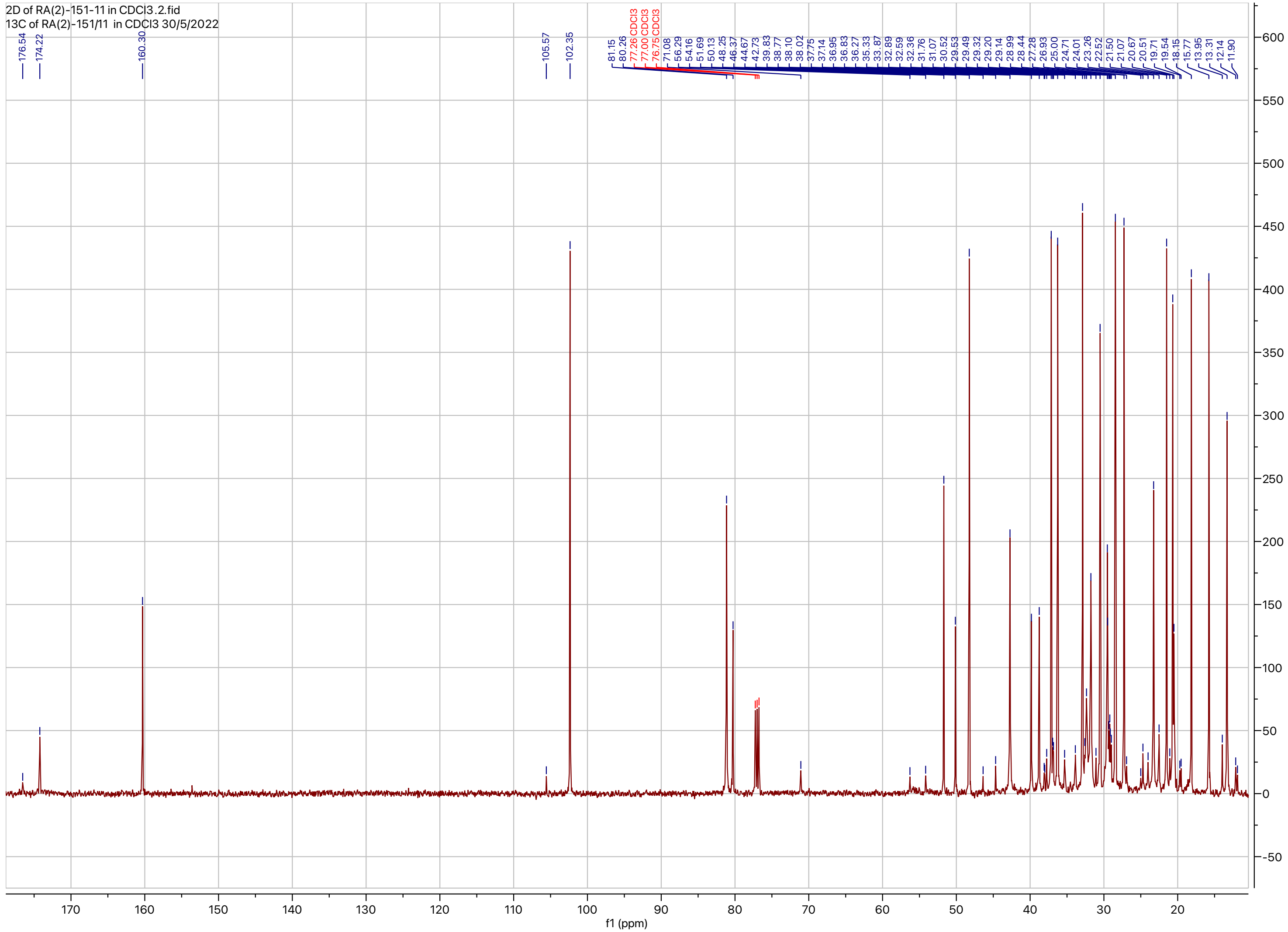

Figure S62. HSQC (500/125 MHz, $CDCl_3$) 2D spectrum of Sigmosceptrellin B Methyl Ester (**8**)

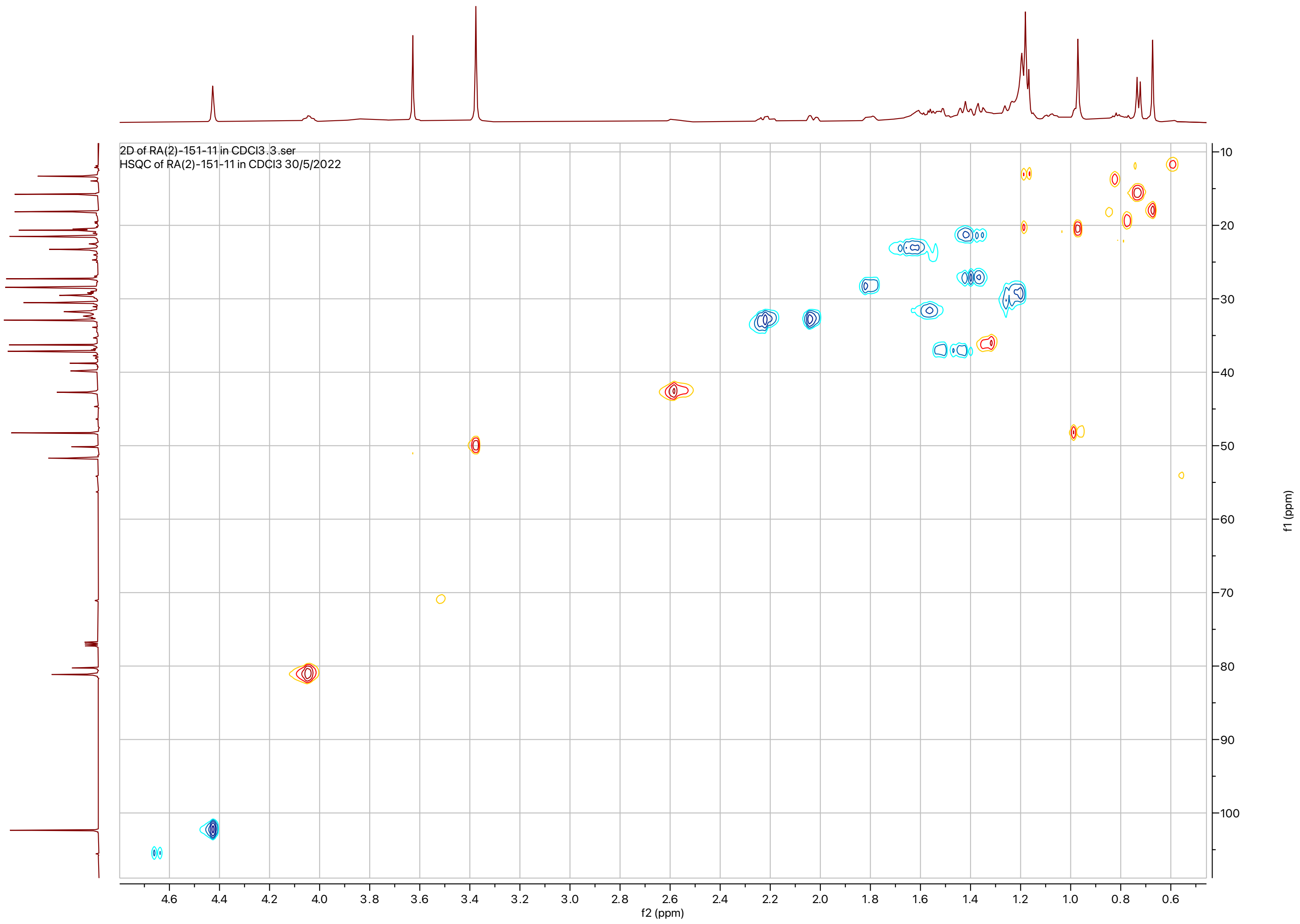

Figure S63. HMBC (500/125 MHz, $CDCl_3$) 2D spectrum of Sigmosceptrellin B Methyl Ester (**8**)

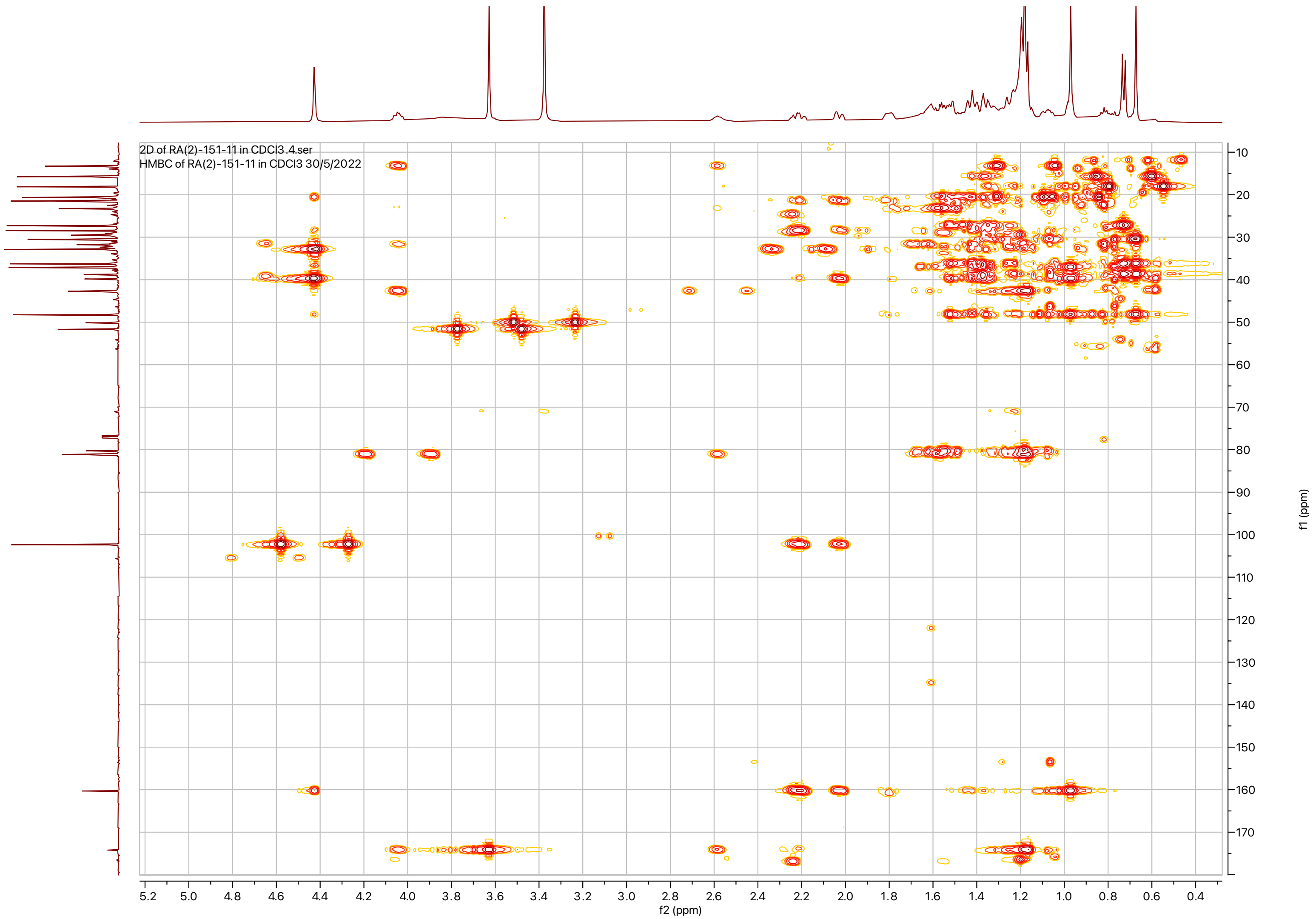

Figure S64. COSY (500 MHz, $CDCl_3$) 2D spectrum of Sigmosceptrellin B Methyl Ester (**8**)

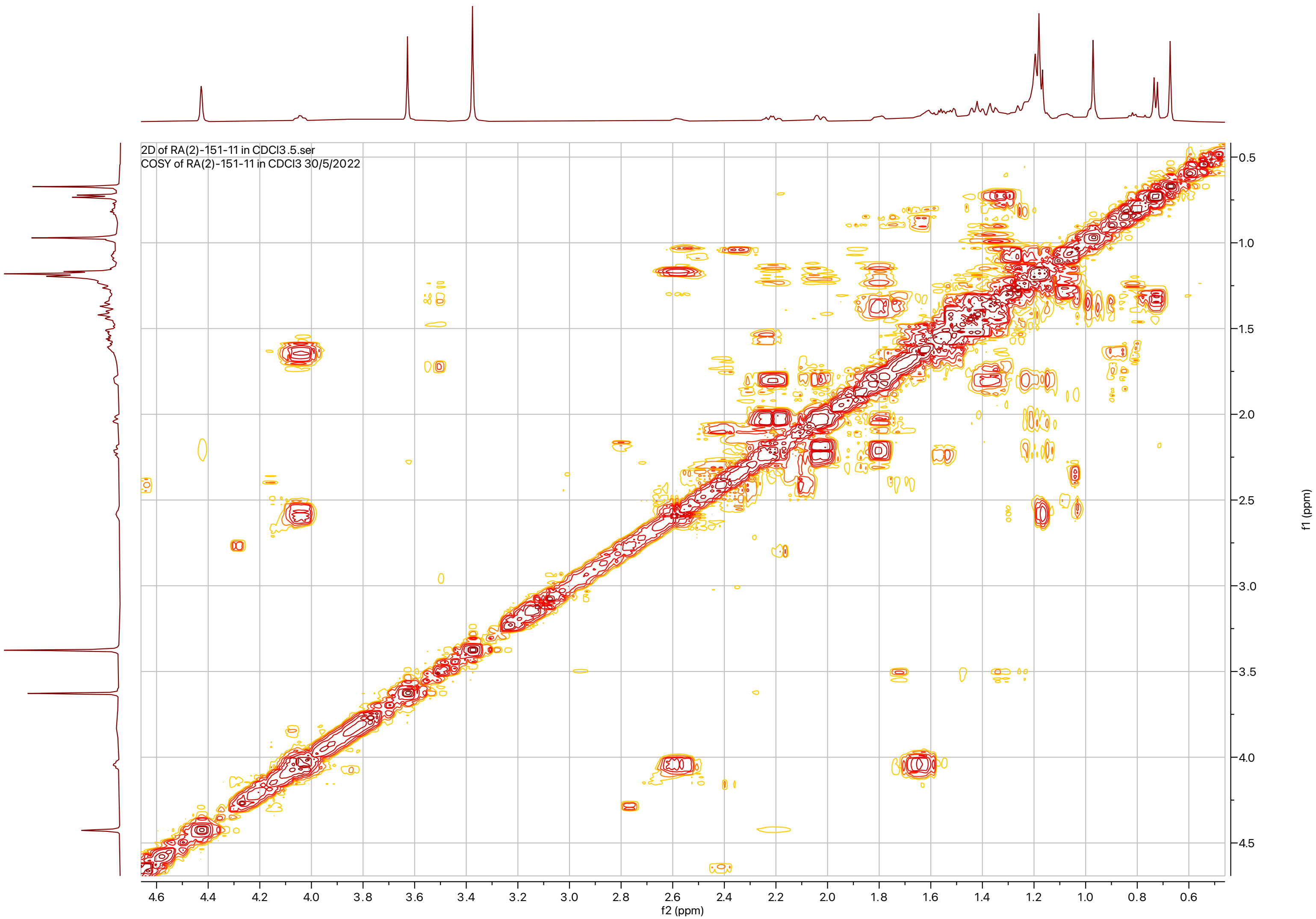

Figure S65. HR ESI MS of Sigmosceptrellin B Methyl Ester (**8**)

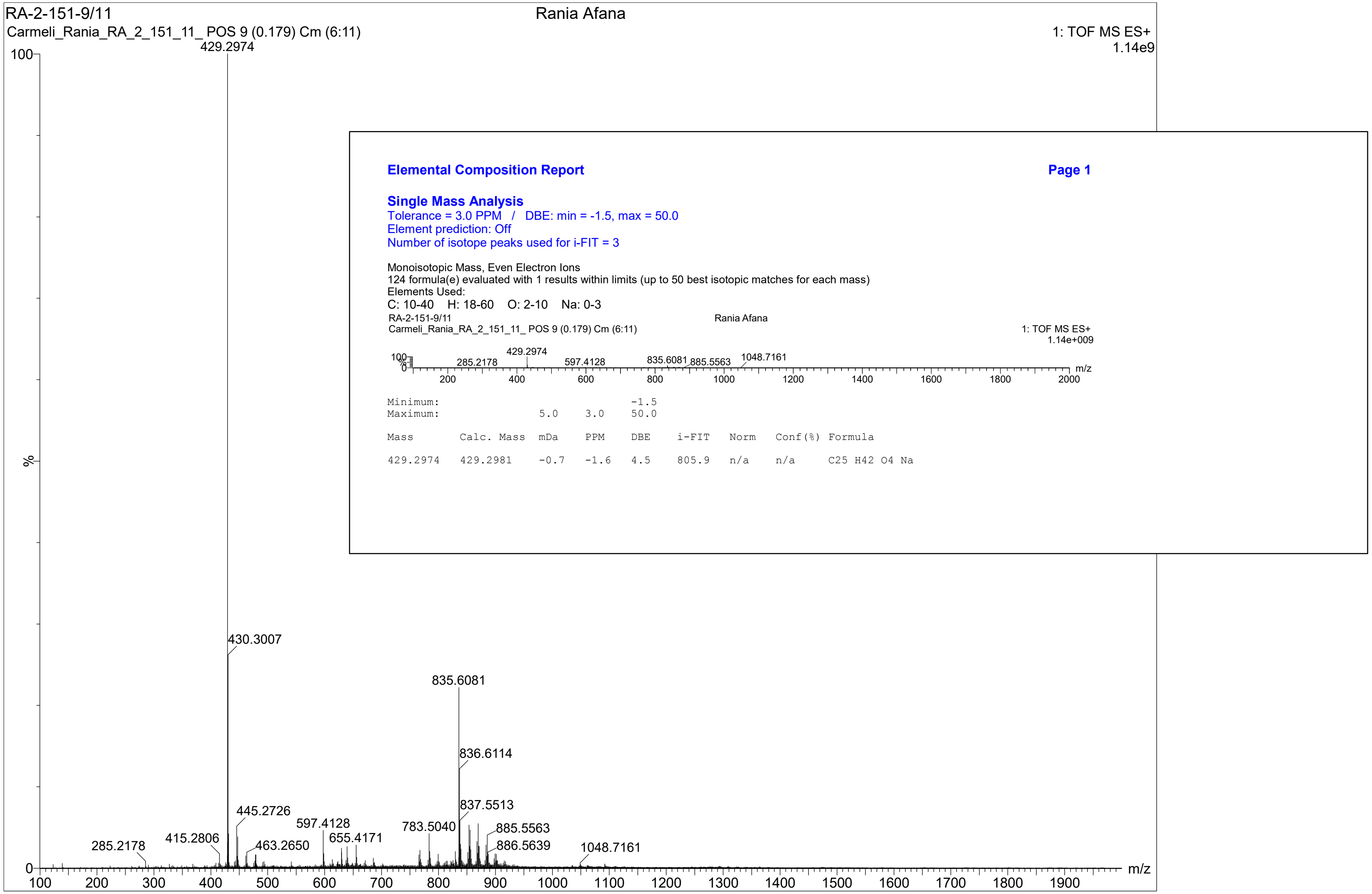

Table S11. NMR data of Sigmosceptrellin B methyl ester (**8**) in $CDCl_3$[a]

Position	δ_C, multiplicity[b]	δ_H, multiplicity, *J* in Hz	HMBC correlations[c]	COSY correlations
1	174.2, C		H-2,3, H_3-19,25	
2	42.7, CH	2.58, dq (8.0, 6.9)	H-3,4’, H_3-19	H-3, H_3-19
3	81.2, CH	4.04, dt (4.0, 8.0)	H-2, H_2-5, H_3-19	H-2, H_2-4
4	23.3, CH_2	1.66, m	H-2, H_2-5	H-3, H_2-5
		1.62, m		H-3, H_2-5
5	31.8, CH_2	1.56, m	H-3,8, H_2-4, H_3-20	H-8, H_2-4, H_3-20
6	80.3, C		H-4’, H_2-5, H_3-20	
7	29.5, CH_2	1.20, m	H_3-20	
8	30.6, CH_2	1.25,	H-10, H_3-21	
		1.15		
9	38.8, C		H_2-11, H_3-21,22	
10	36.3, CH	1.33, m	H-8,12, H_2-11, H_3-21,22	H_3-22
11	27.3, CH_2	1.42, m	H-8,10, H_2-12, H_3-22	
		1.35, m		
12	37.1, CH_2	1.51, m	1.66, 1.62, 1.38, H-18	
		1.43, m		
13	39.7, C		H-11’,12,16,18, H_2-15,24	
14	160.3, C		H-18, H_2-15,16,17,24,	
15	32.9, CH_2	2.21, dt (5.0, 13.3)	H_2-17,24	H-15’, H_2-16,24
		2.03, brd (13.3)		H-15, H_2-16
16	28.4, CH_2	1.80, m	H_2-15,17	H-16’,17’, H_2-15
		1.19, m		H-16,17, H_2-15
17	21.5, CH_2	1.42, m	H-12,16, H_2-15	
		1.37, m		
18	48.3, CH	0.97, m	H-16, H_2-12,24, H_3-21,23	
19	13.3, CH_3	1.17, d (6.9)	H-2,3	H-2
20	20.5, CH_3	1.18, s	H_2-5	
21	18.2, CH_3	0.67, s	H-8,10, H_3-23	
22	15.8, CH_3	0.73, d (6.6)	H-17’	H-10

23	20.7, CH_3	0.97, s	H-17’, H_2-24	H-17’
24	102.4, CH_2	4.43, brs	H_2-15	H-15
25	51.7, CH_3	3.62, s		

[a]500 MHz for ^{1}H, 125 MHz for ^{13}C; [b]From HSQC; [c]Optimized for J_{C-H}=8 Hz.

Figure S66. ^{1}H NMR (500 MHz, DMSO-d_6) spectrum of Diacarperoxide A (**9**)

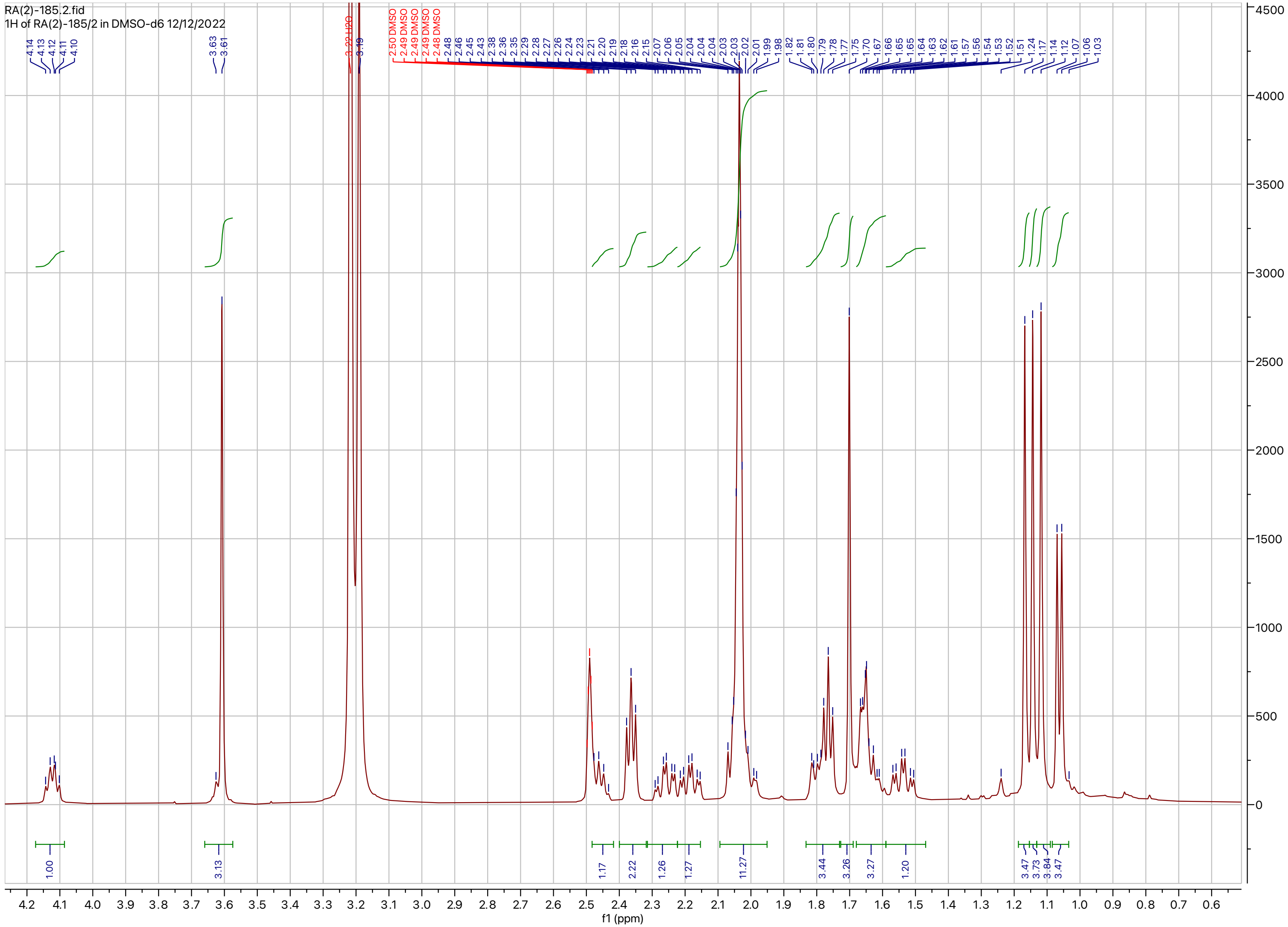

Figure S67. ^{13}C NMR (125 MHz, DMSO-d_6) spectrum of Diacarperoxide A (**9**)

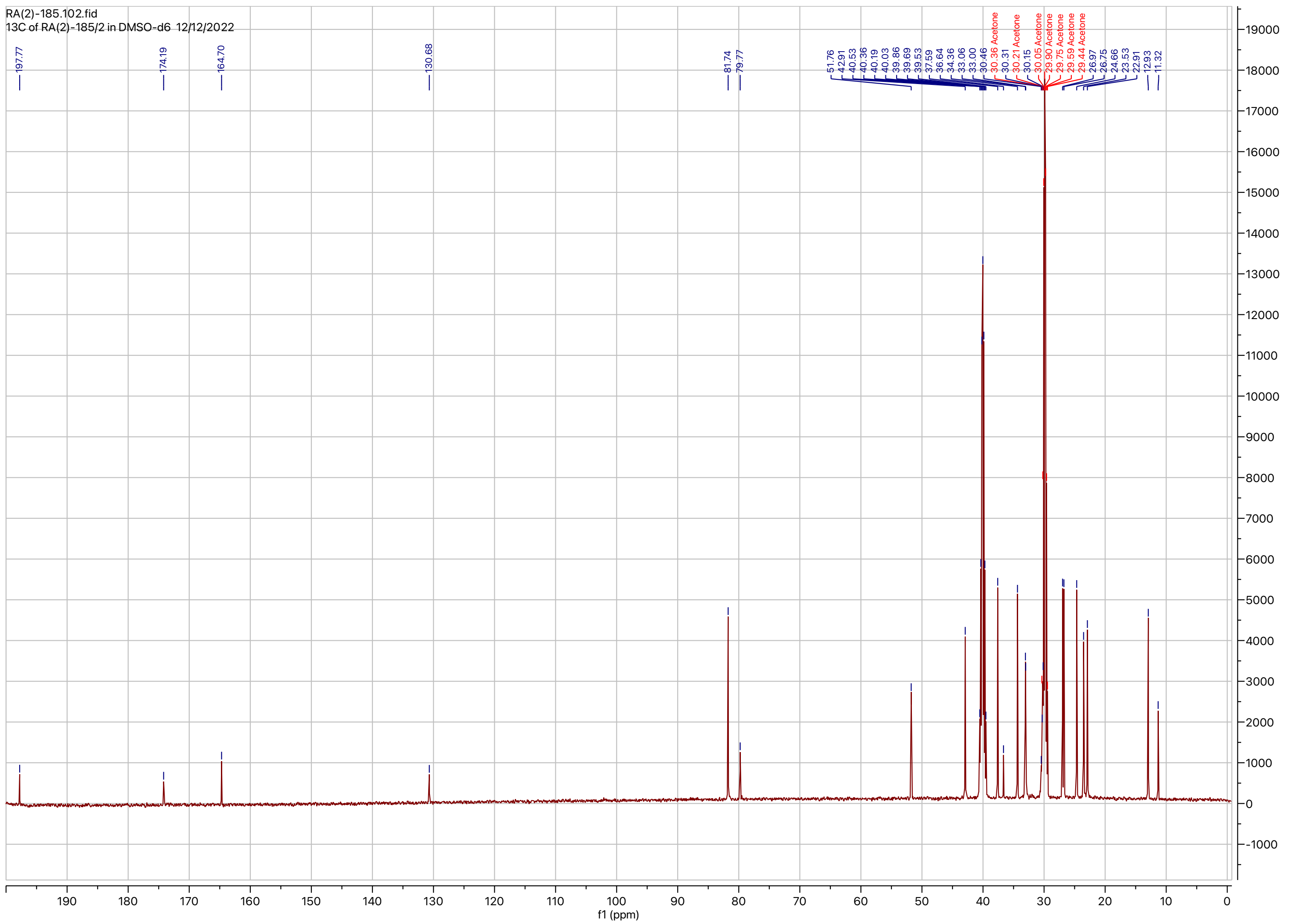

Figure S68. HSQC (500/125 MHz, DMSO-d_6) 2D spectrum of Diacarperoxide A (**9**)

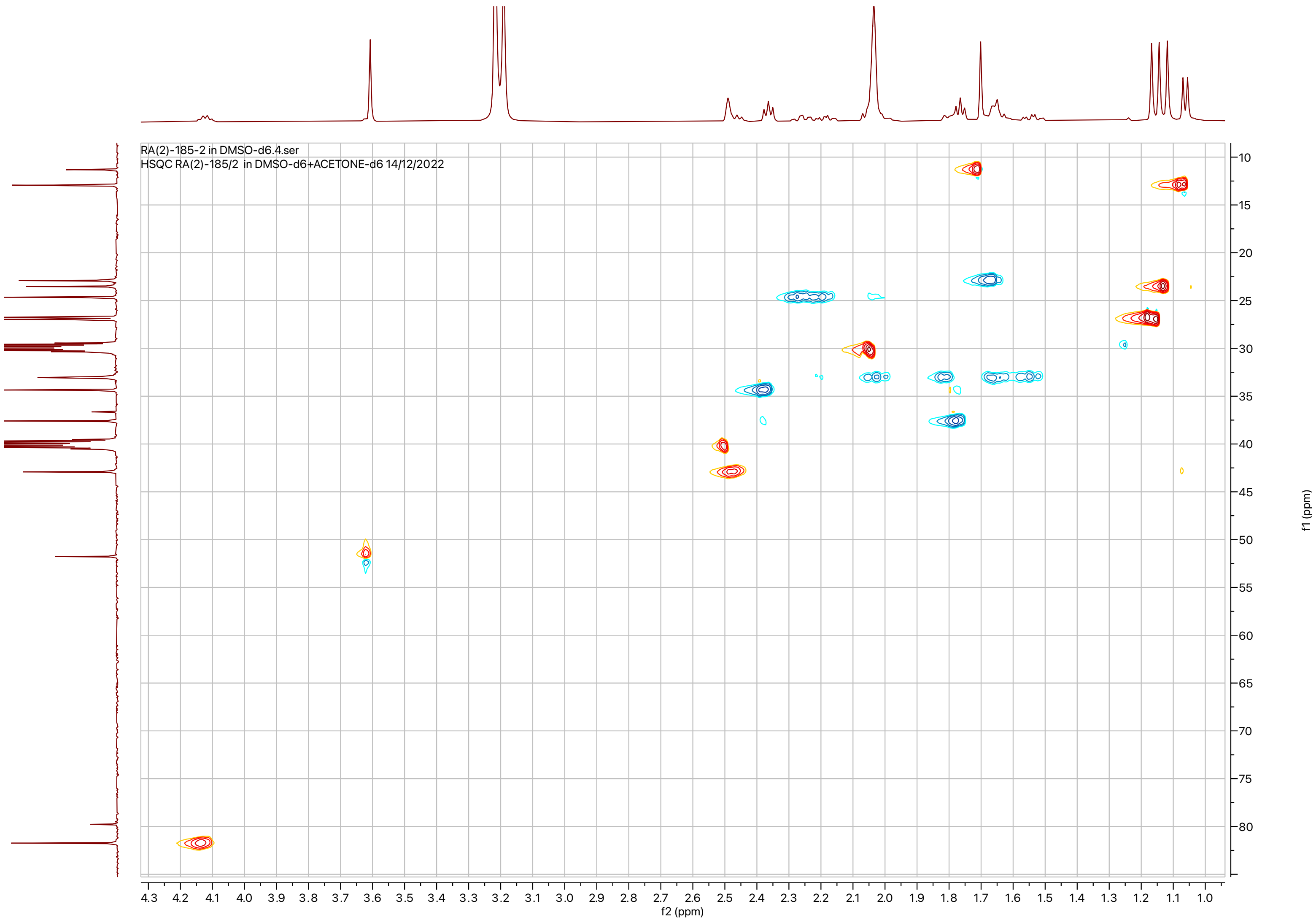

Figure S69. HMBC (500/125 MHz, DMSO-d_6) 2D spectrum of Diacarperoxide A (**9**)

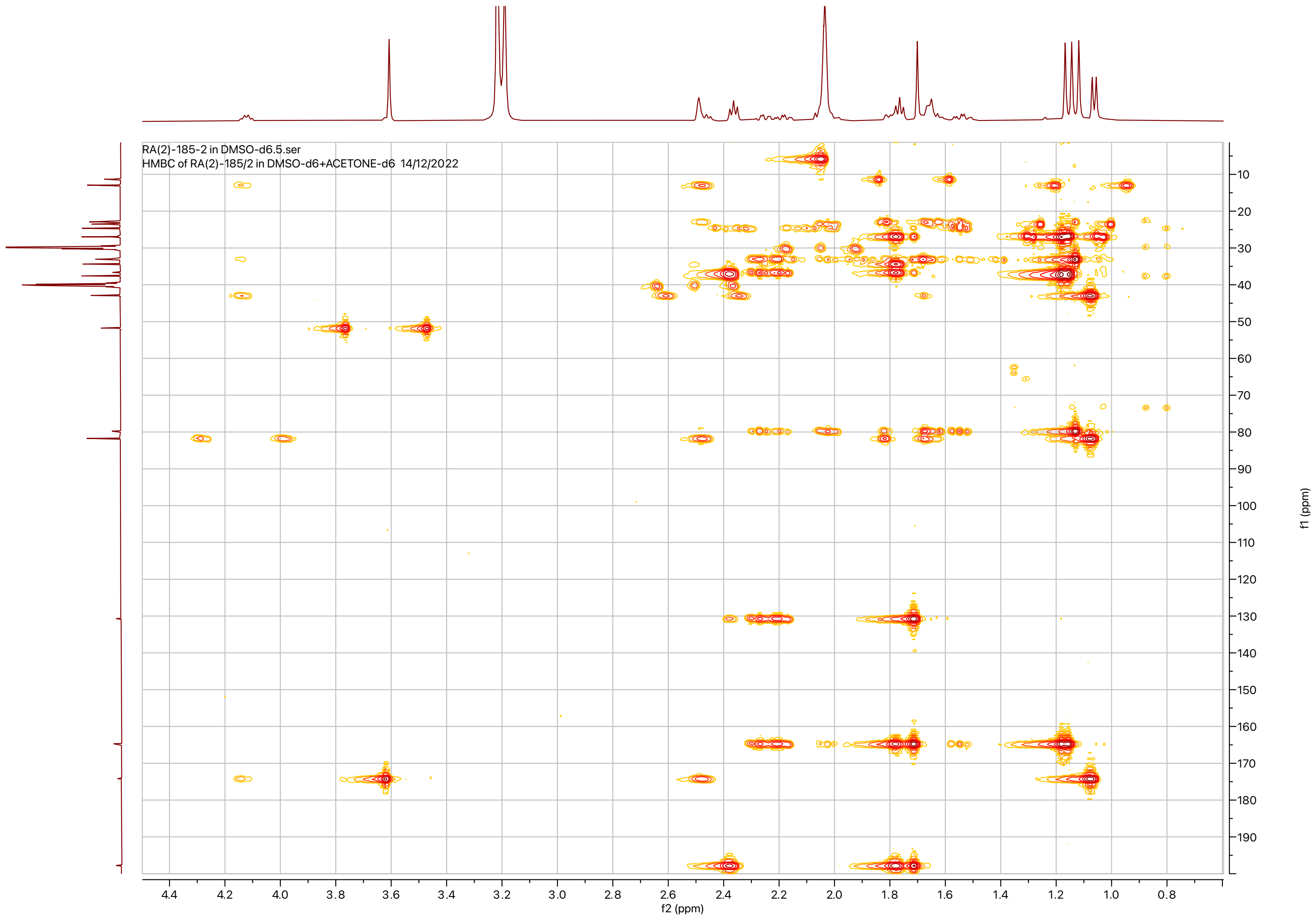

Figure S70. COSY (500 MHz, DMSO-d_6) 2D spectrum of Diacarperoxide A (**9**)

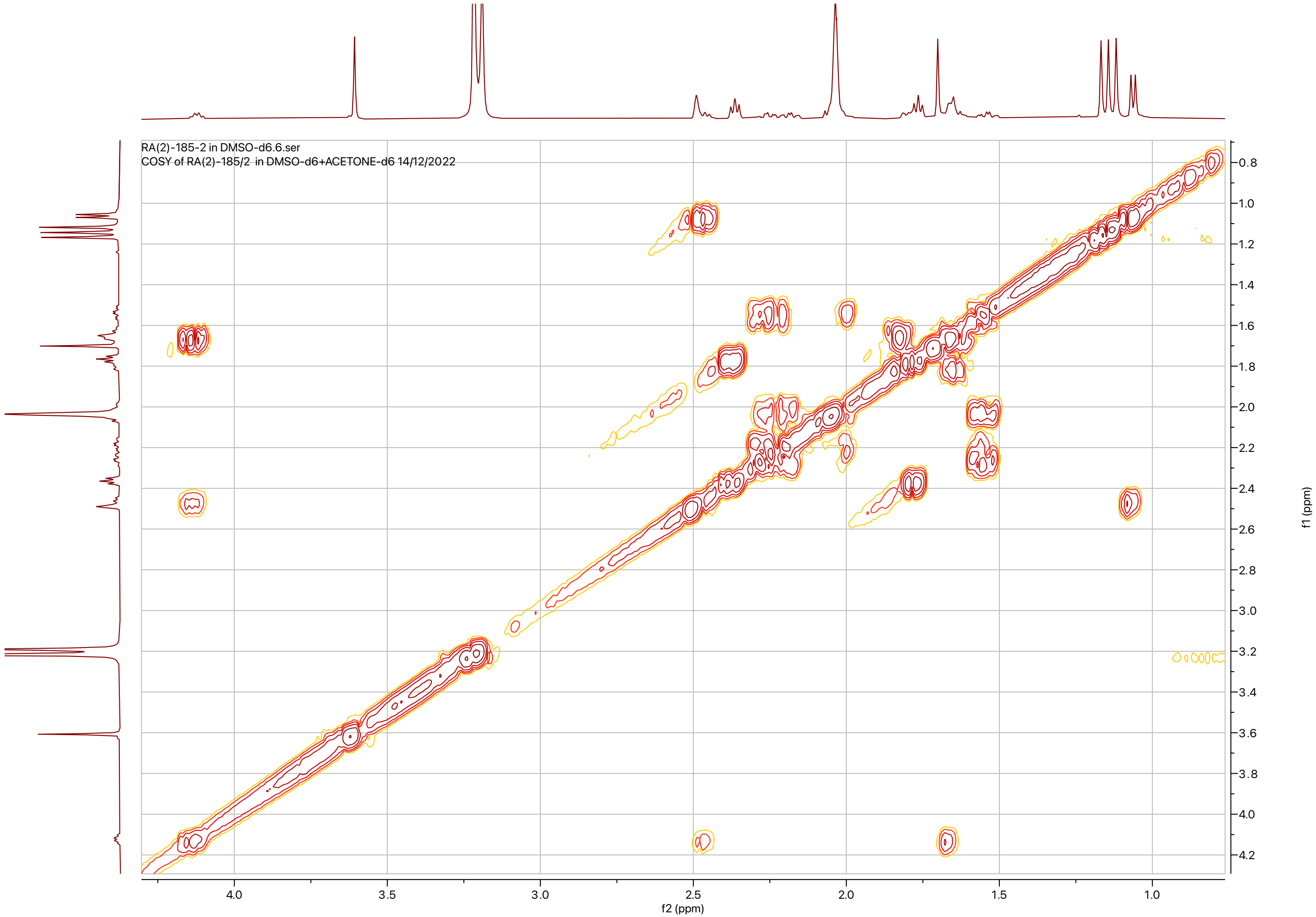

Figure S71. HR ESI MS of Diacarperoxide A (**9**)

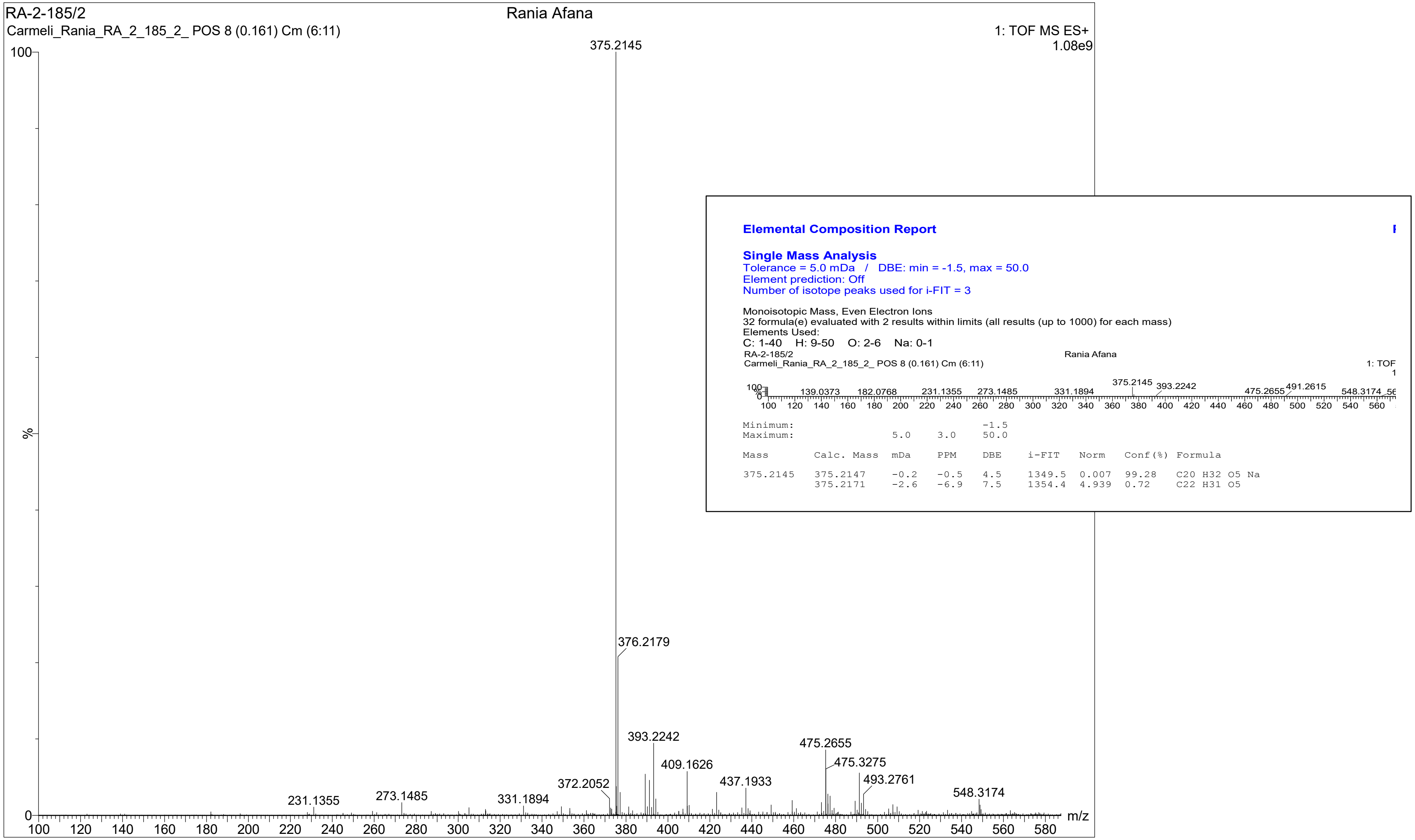

Table S12. NMR Data of Diacarperoxide A (**9**) in DMSO-d_6[a]

Position	δ_C, multiplicity[b]	δ_H, multiplicity, J in Hz	HMBC correlations[c]	COSY correlations	ROSEY correlations
1	173.7, C		H-2,3, H_3-15,20		
2	42.0, CH	2.69, dq, (7.0, 6.8)	H-3, H_3-15	H-3, H_3-15	H-3, H_2-4, H_3-15
3	80.9, CH	4.05, m	H-2, H_2-4,5, H_3-15	H-2, H_2-4	H-2, H_2-4, H_3-15
4	22.6, CH_2	1.66, m	H-2, H_2-5	H-3, H_2-4	H-3
5	31.2, CH_2	1.69, m	H_2-4,7, H_3-16	H_2-5	H_2-7
6	79.9, C		H_2-4,5,7,8, H_3-16		
7	37.4, CH_2	1.55, m	H_2-8, H_3-16	H_2-8	
		1.49, m		H_2-8	
8	23.9, CH_2	2.19, t (8.4)	H-7’	H_2-7	H_2-5, H_3-16,18,19
9	164.9, C		H_2-8,13, H_3-17,18,19		
10	130.2, C		2.35, H_2-8, H_3-17		
11	197.7, C		H_2-12,13, H_3-17		
12	34.0, CH_2	2.35, t (6.8)	H_2-13	H_2-13	H_3-18,19
13	37.0, CH_2	1.72, t (6.8)	H_2-12, H_3-18,19	H_2-12	H_3-18,19
14	36.4, C		H_2-8, H_2-13, H_3-18,19		
15	12.8, CH_3	1.10, d (6.8)	H-2,3	H-2	H-2,3
16	20.5, CH_3	1.25, s	H-7, H_2-5		H_2-5,8
17	11.4, CH_3	1.64, s			H-7’, H_2-8, H_3-18,19
18	26.7, CH_3	1.11, s	H_2-13, H_3-19		H_2-8,12,13
19	26.8, CH_3	1.11, s	H_2-13, H_2-18		H_2-8,12,13
20	51.9, CH_3	3.61, s			

[a]500 MHz for ^{1}H and 125 MHz for ^{13}C. [b]Assigned by HSQC experiment; [c]Optimized for $J_{C\text{-}H}$=8 Hz.

Figure S72. ^{1}H NMR (500 MHz, $CDCl_3$) spectrum of Methyl Diacarnoate A (**10**)

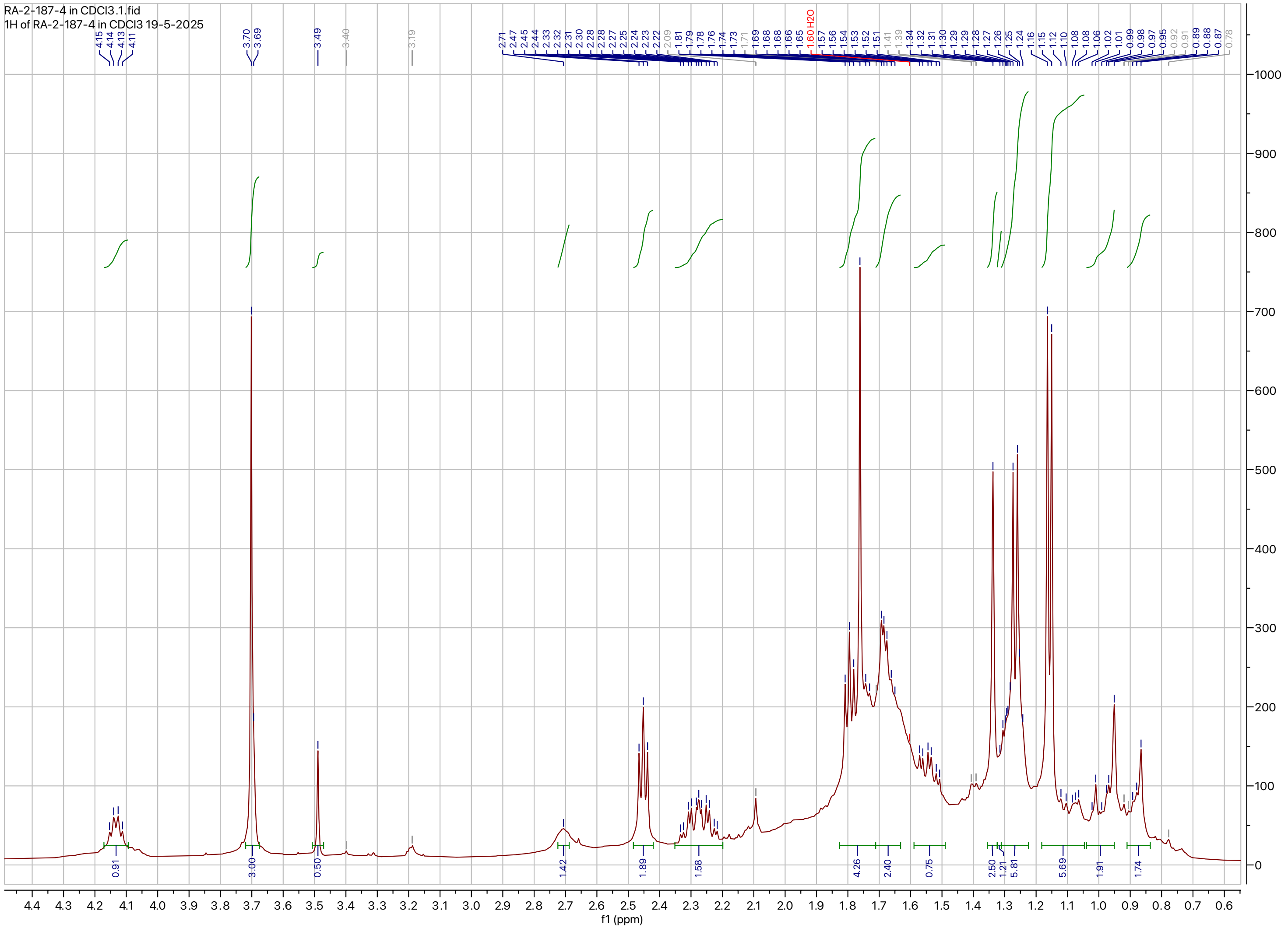

Figure S73. ^{13}C NMR (125 MHz, $CDCl_3$) spectrum of Methyl Diacarnoate A (**10**)

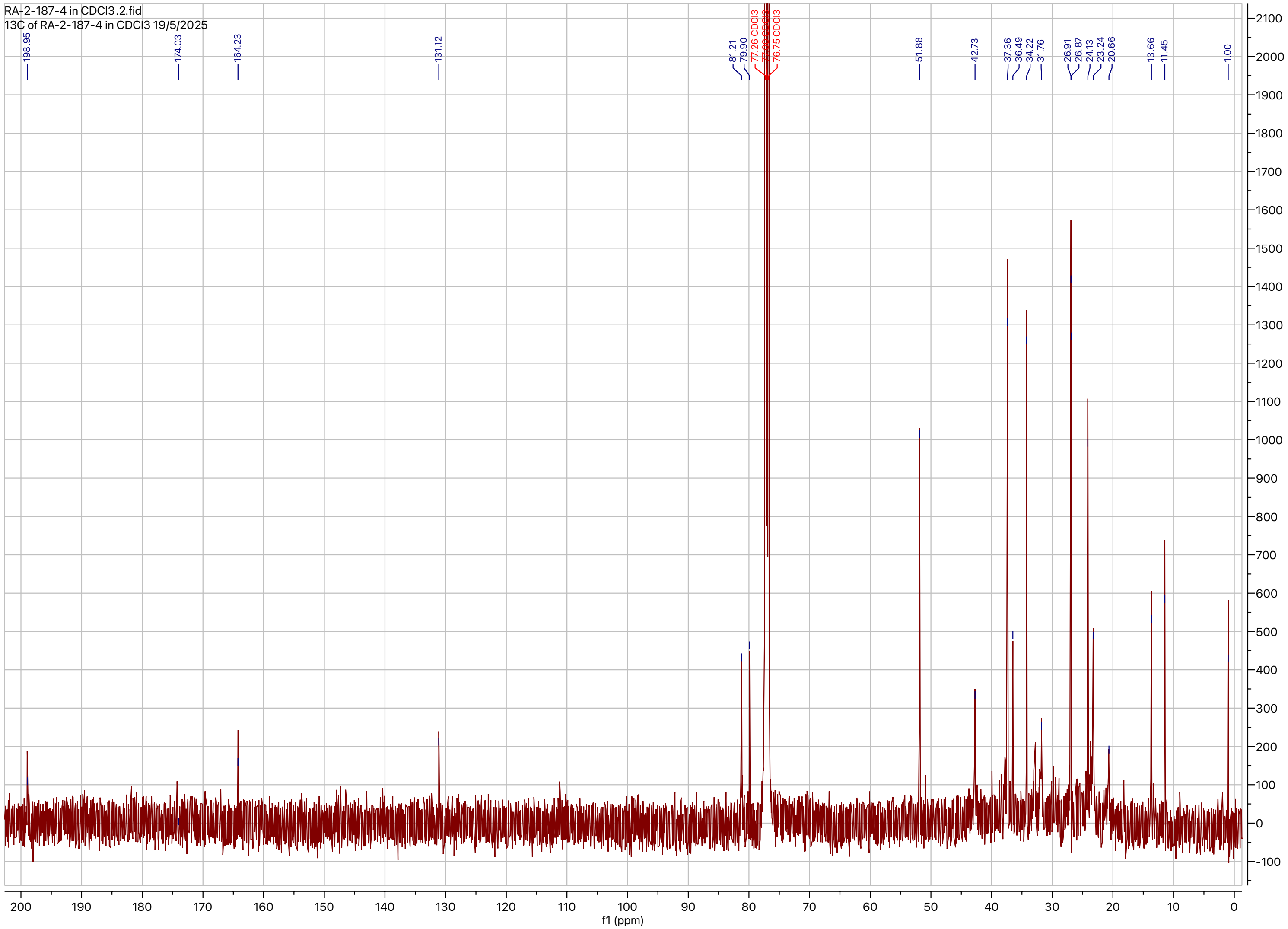

Figure S74. HSQC (500/125 MHz, $CDCl_3$) 2D spectrum of Methyl Diacarnoate A (**10**)

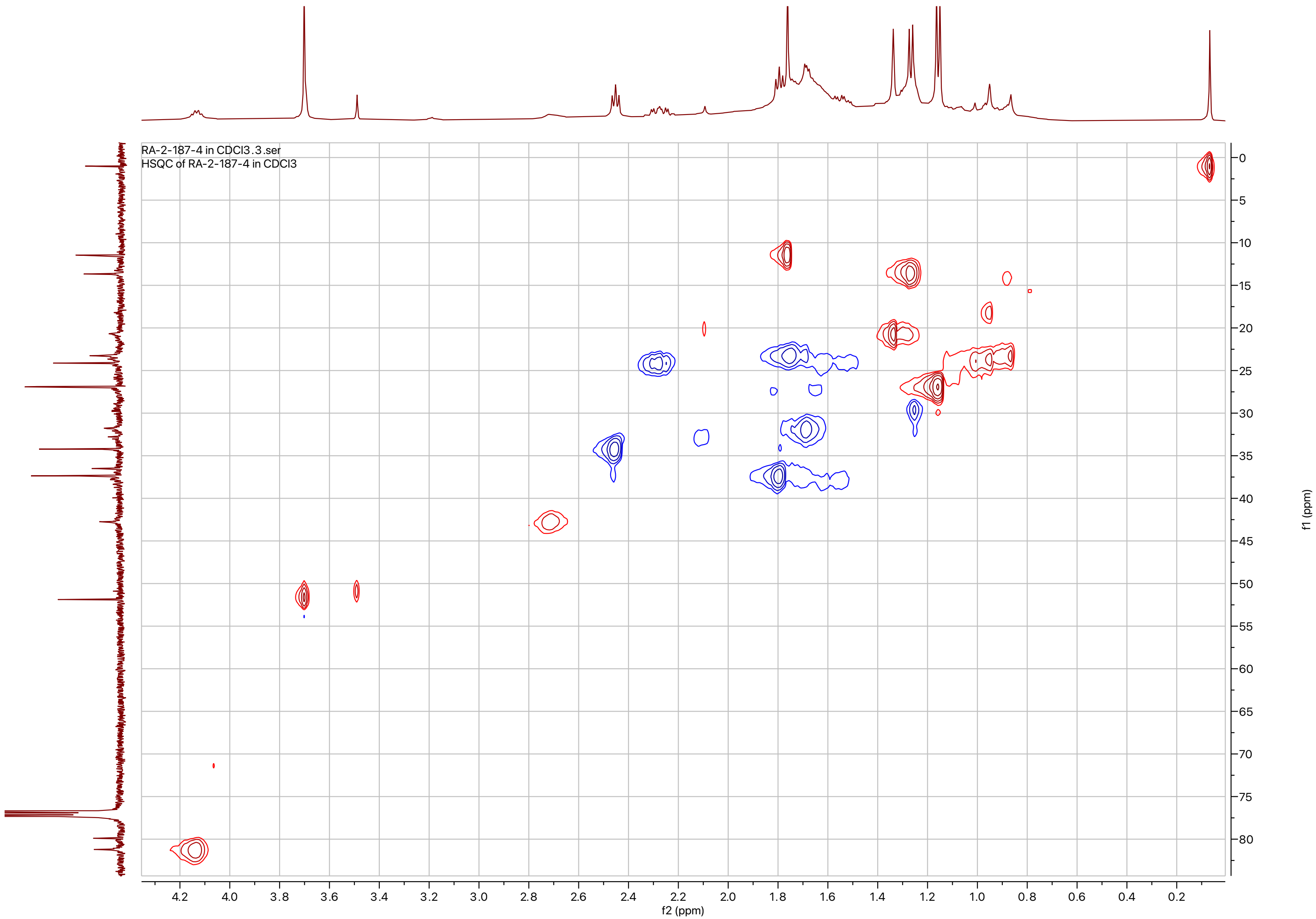

Figure S75. COSY (500 MHz, $CDCl_3$) 2D spectrum of Methyl Diacarnoate A (**10**)

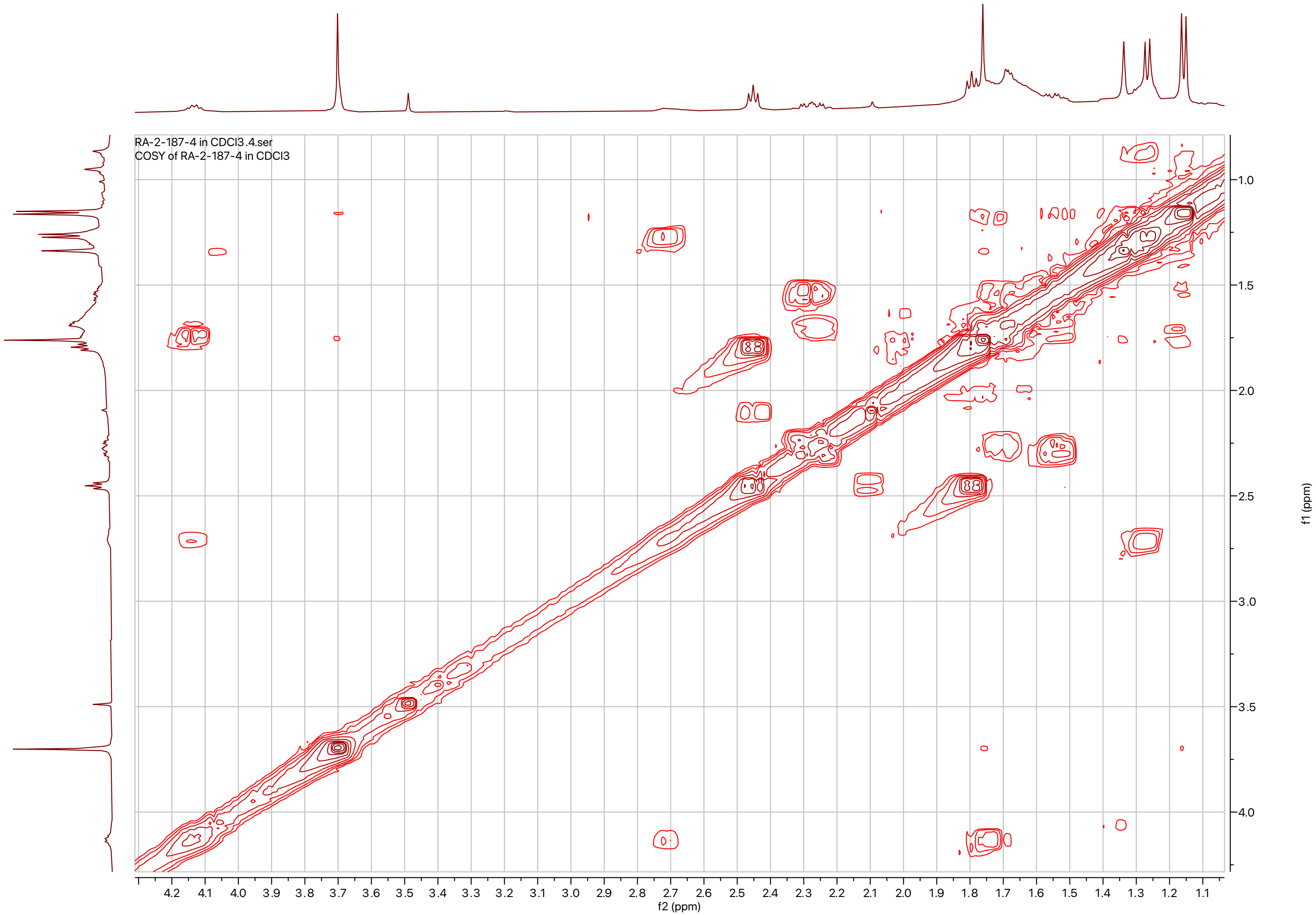

Figure S76. HR ESI MS of Methyl Diacarnoate A (**10**)

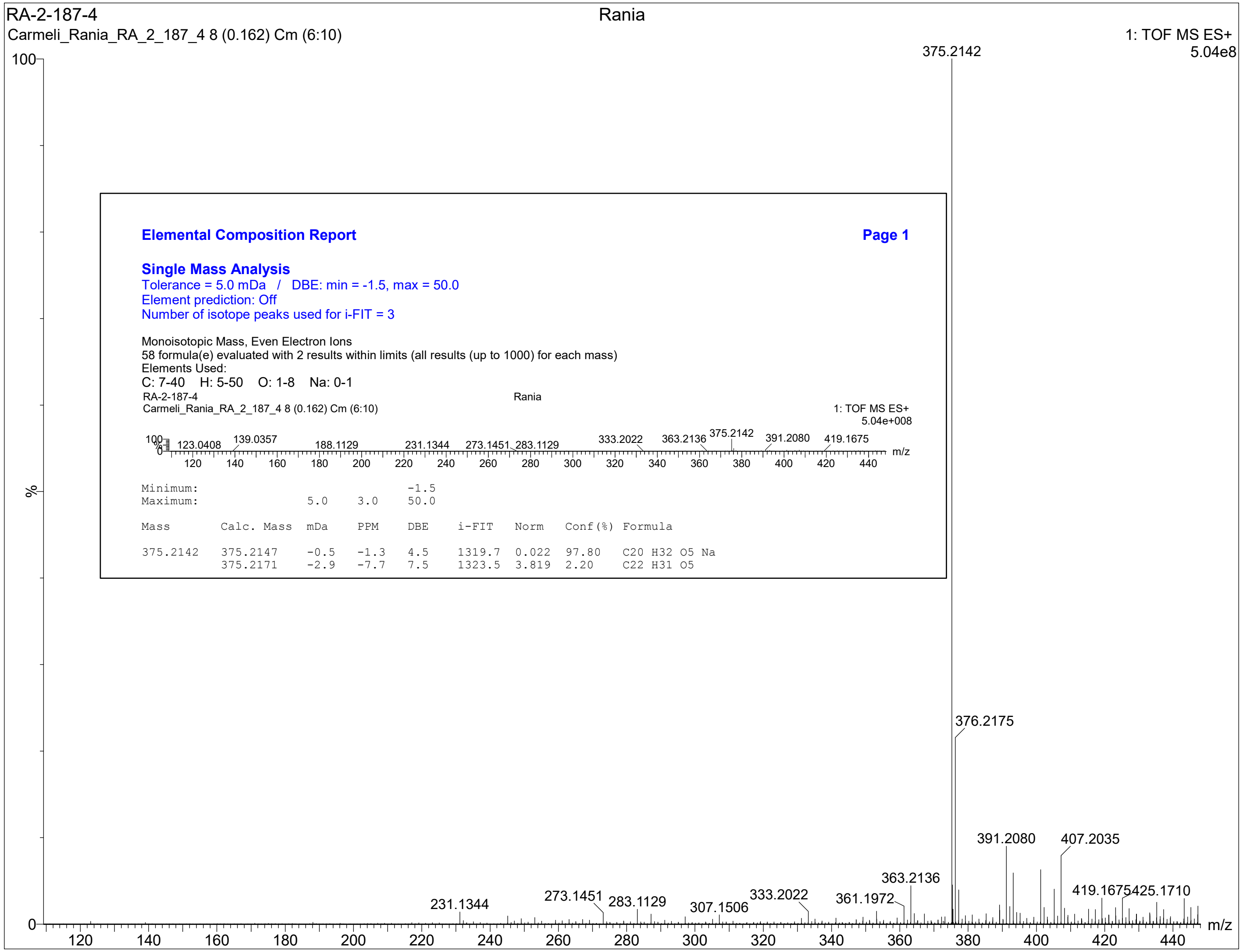

Table S13. NMR data of Methyl Diacarnoate A (**10**) in $CDCl_3$[a]

Position	δ_C, multiplicity[b]	δ_H, multiplicity, *J* in Hz	COSY correlations
1	174.0, C		
2	42.7, CH	2.70, m	H-3, H_3-15
3	81.2, CH	4.13, ddd (7.2, 6.7, 6.3)	H-2, H_2-4
4	23.2, CH_2	1.75, m	H_2-5
5	31.8, CH_2	1.68, m	H_2-4
6	79.9, C		
7	37.7, CH_2	1.70, m	H-7’, H_2-8
		1.54, dt (4.9, 13.2)	H-7, H_2-8
8	24.1, CH_2	2.30, dt (4.9, 12.6)	H-8’, H_2-7
		2.25, dt (4.9, 12.6)	H-8, H_2-7
9	164.2, C		
10	131.1, C		
11	199.0, C		
12	34.2, CH_2	2.45, t (6.8)	H_2-13
13	37.4, CH_2	1.79, t (6.8)	H_2-12
14	36.5, C		
15	13.7, CH_3	1.27, d (7.2)	H-2
16	20.7, CH_3	1.34, brs	
17	11.5, CH_3	1.76, s	
18	26.9, CH_3	1.16, s	
19	26.9, CH_3	1.15, s	
20	51.9, CH_3	3.70, s	

[a]500 MHz for ^1H and 125 MHz for ^{13}C. [b]Assigned by HSQC experiment.

Figure S77. ^{1}H NMR (500 MHz, $CDCl_3$) spectrum of (-)-*ent*-Muqubilone (**11**)

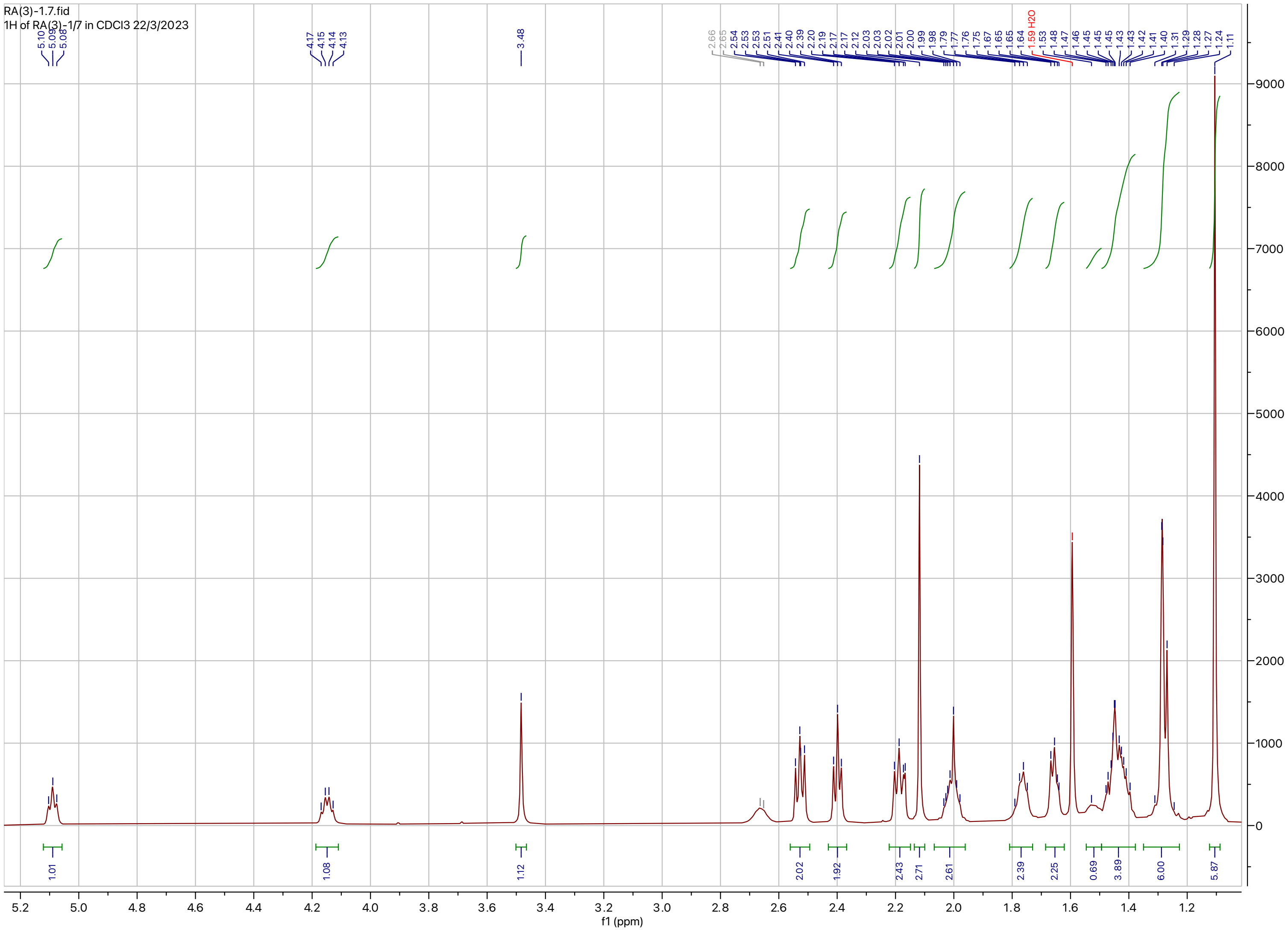

Figure S78. ^{13}C NMR (125 MHz, CDCl$_3$) spectrum of (-)-*ent*-Muqubilone (**11**)

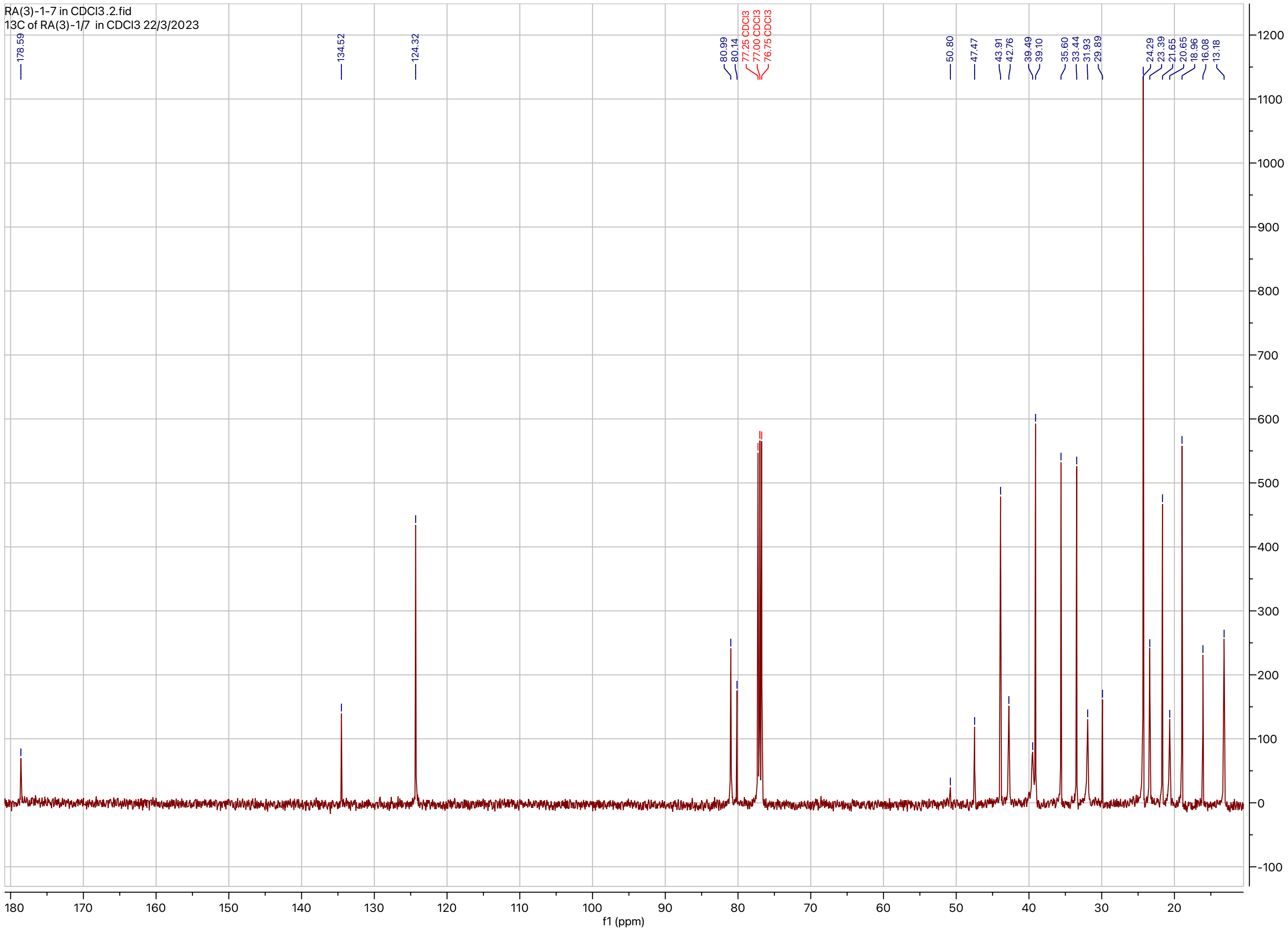

Figure S79. HSQC (500/125 MHz, $CDCl_3$) 2D spectrum of (-)-*ent*-Muqubilone (**11**)

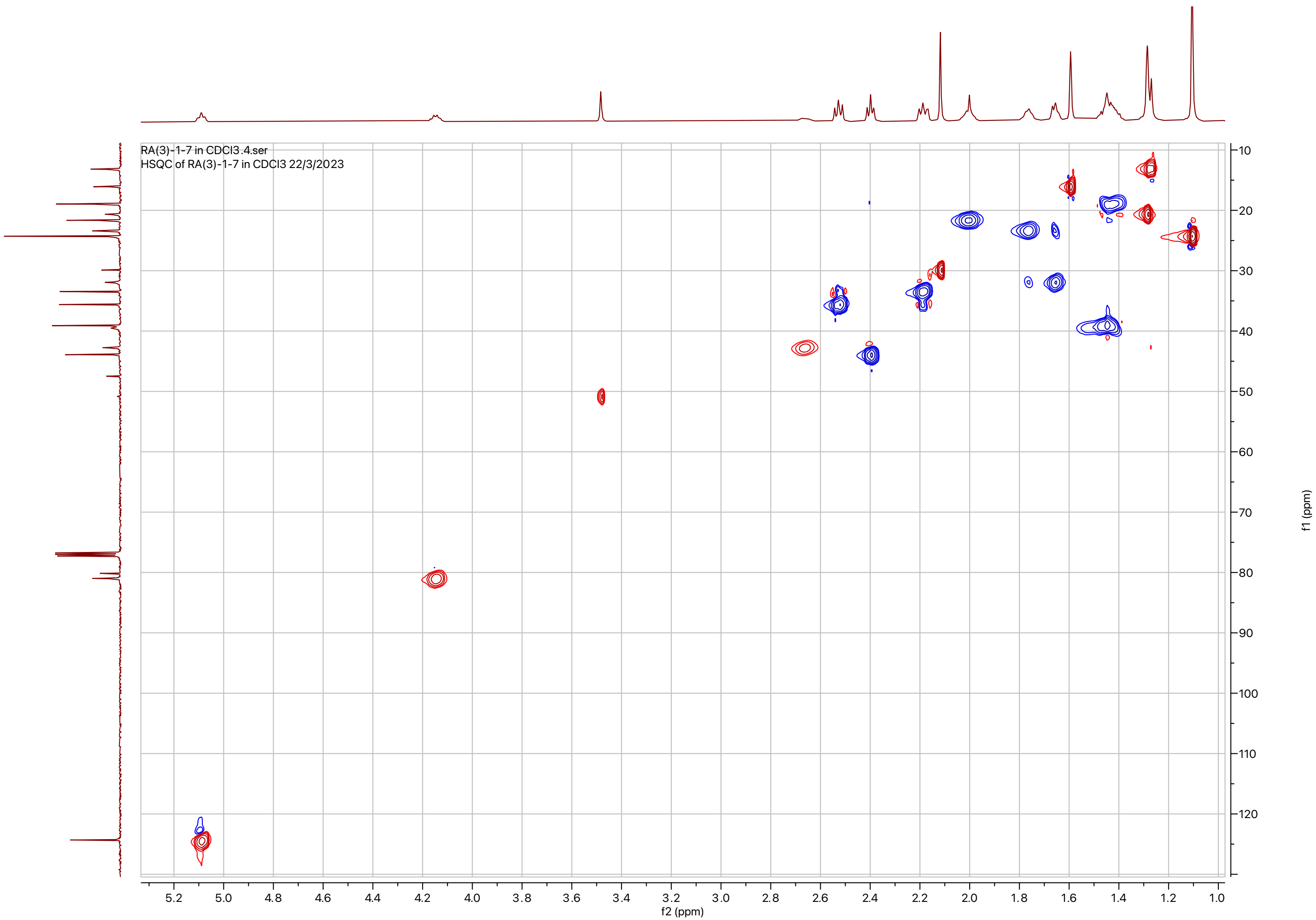

Figure S80. HMBC (500/125 MHz, $CDCl_3$) 2D spectrum of (-)-*ent*-Muqubilone (**11**)

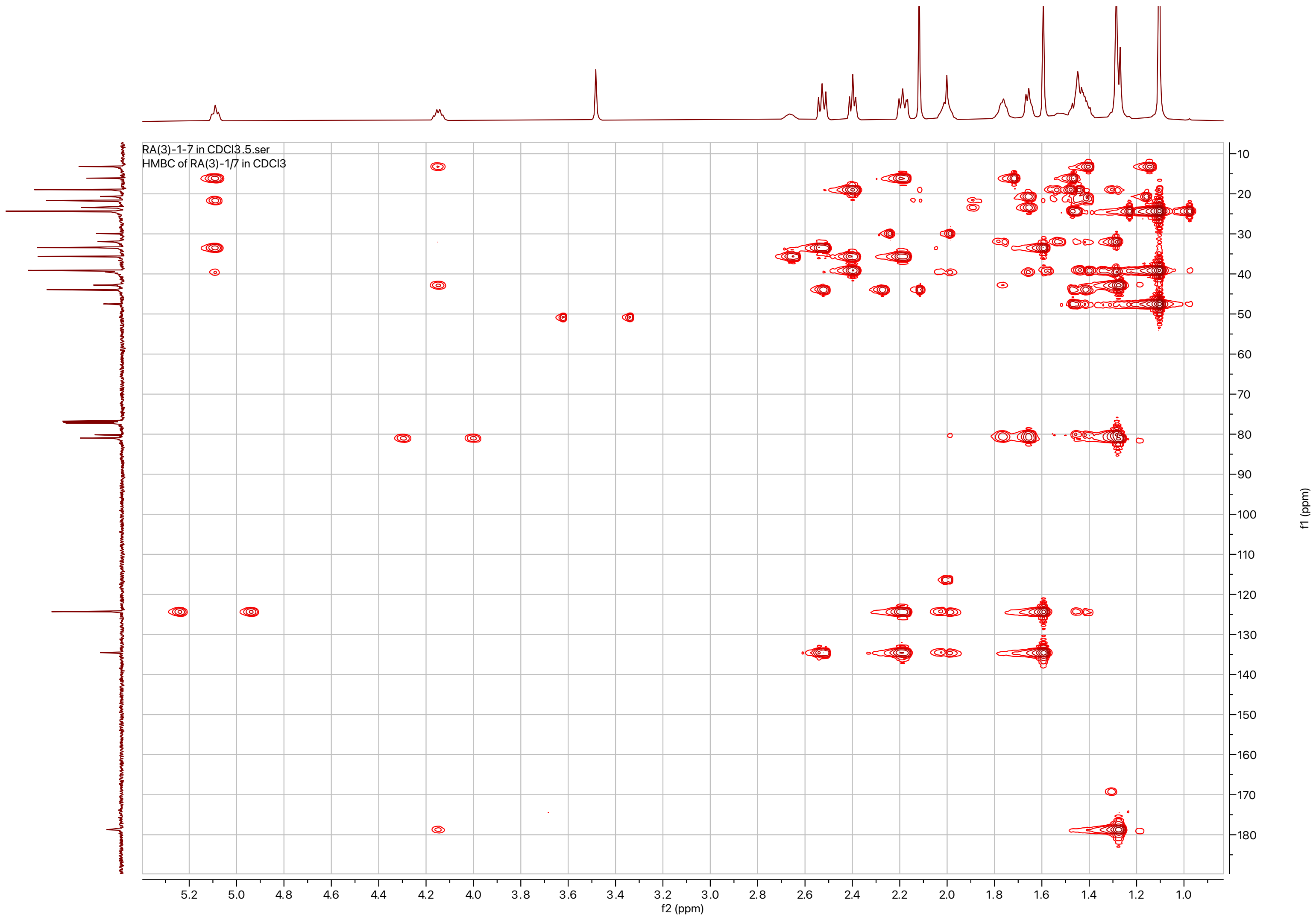

Figure S81. COSY (500 MHz, $CDCl_3$) 2D spectrum of (-)-*ent*-Muqubilone (**11**)

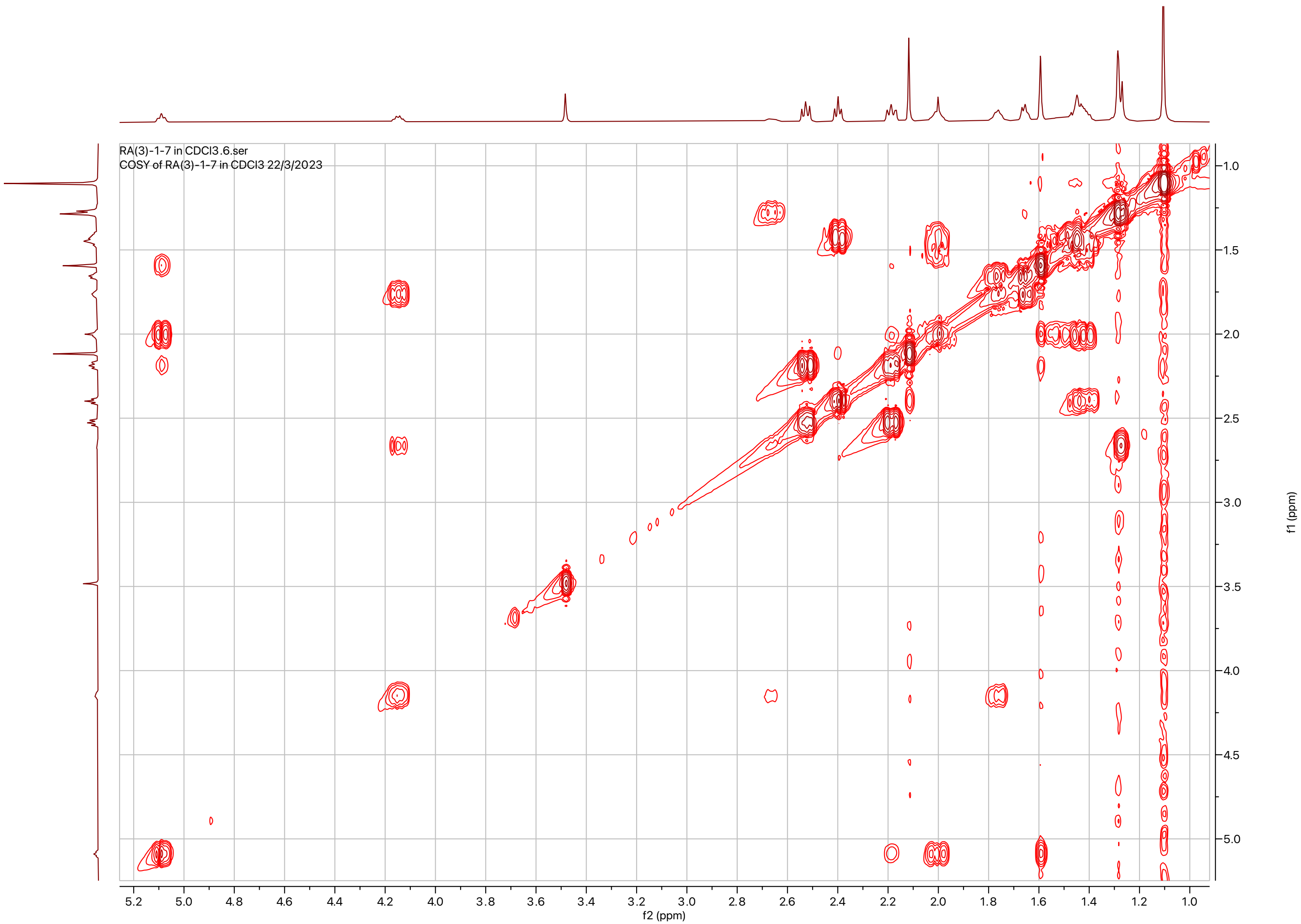

Figure S82. HR ESI MS of (-)-*ent*-Muqubilone (**11**)

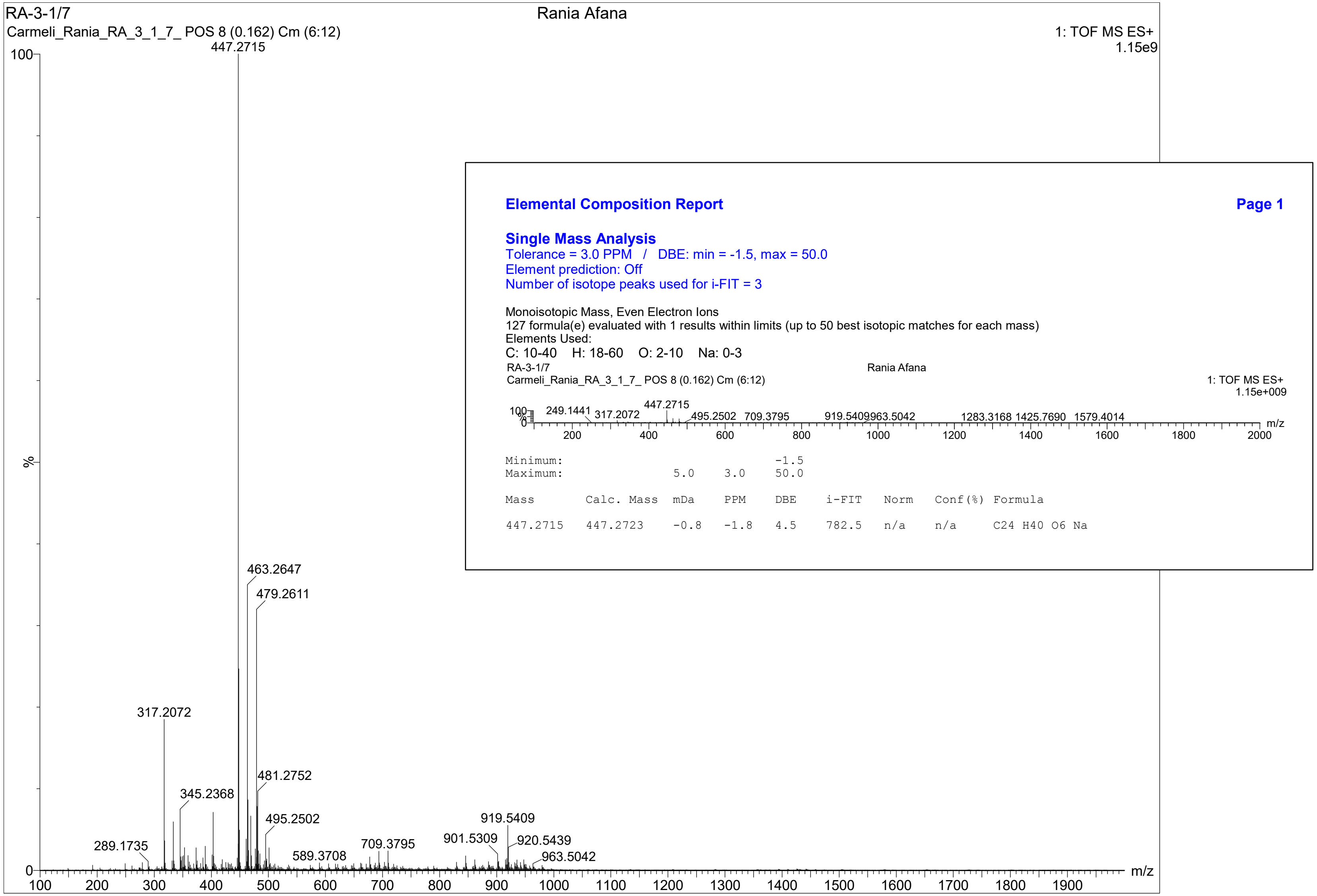

Table S14. NMR Data of (-)-*ent*-Muqubilone (**11**) in $CDCl_3$[a]

Position	δ_C, multiplicity[b]	δ_H, multiplicity, J in Hz	HMBC correlations[c]	COSY correlations
1	178.8, C		H_3-20	
2	42.8, CH	2.66, brdq (6.2, 7.2)	H-3, H_3-20	H-3, H_3-20
20	13.2, CH_3	1.28, d (7.2)	H-3	H-2,3
3	81.0, CH	4.15, brddd (7.3, 6.8, 6.3)	H_2-4,5, H_3-20	H-2, H_2-4
4	23.4, CH_2	1.76, m	H_2-5	H-3, H_2-5
5	31.9, CH_2	1.65, m	H_2-7, H_3-21	H_2-4
6	80.2, C		H_2-4,5,7, H_3-21	
21	20.7, CH_3	1.29, s	H_2-5,7	
7	39.5, CH_2	1.52, m	H-9, H_2-5,8 H_3-21	H-7’, H_2-8
		1.42, m		H-7, H_2-8
8	21.7, CH_2	2.01, brm	H-9, H_2-7	H-9, H_2-16, H_3-22
9	124.3, CH	5.09, t (6.8)	H_2-8,11 H_3-22	H_2-8,11 H_3-22
10	134.5, C		H_2-8,11,12, H_3-22	
22	16.1, CH_3	1.59, brs	H-9, H_2-11	H-9 H_2-11, H_3-23,24
11	33.5, CH_2	2.19, dd (7.9, 7.5)	H-9, H_2-12, H_3-22	H-9, H_2-8,12,17, H_3-22
12	35.6, CH_2	2.53, dd (7.9, 7.5)	H_2-11	H_2-11
13	215.2, C		H_2-11,12,15, H_3-23,24	
14	47.5, C		H_2-15, H_3-23,24	
23	24.3, CH_3 x2	1.10, s x2	H_2-15, H_3-23,24	H_2-17, H_3-22
24	24.3, CH_3 x2	1.10, s x2	H_2-15, H_3-23,24	H_2-17, H_3-22
15	39.1, CH_2	1.45, m	H-16’, H_2-15,17, H_3-23,24	
16	19.0, CH_2	1.45, m	H_2-15,17 H_3-19,23,24	H_2-8,17
		1.40, m		H_2-8
17	43.9, CH_2	2.40, t (6.8, 6.2)	H_3-19, H_2-15,16	H_3-19, H_2-16,17, H_3-23,24
18	208.7, C		H-16’, H_2-17, H_3-19	
19	29.9, CH_3	2.12, brs		H_2-17

[a]500 MHz for ^{1}H and 125 MHz for ^{13}C. [b]Assigned by HSQC experiment.

Figure S83. Concentration-response cytotoxicity curves of Diacarnusin A (**1**) on the different cell lines

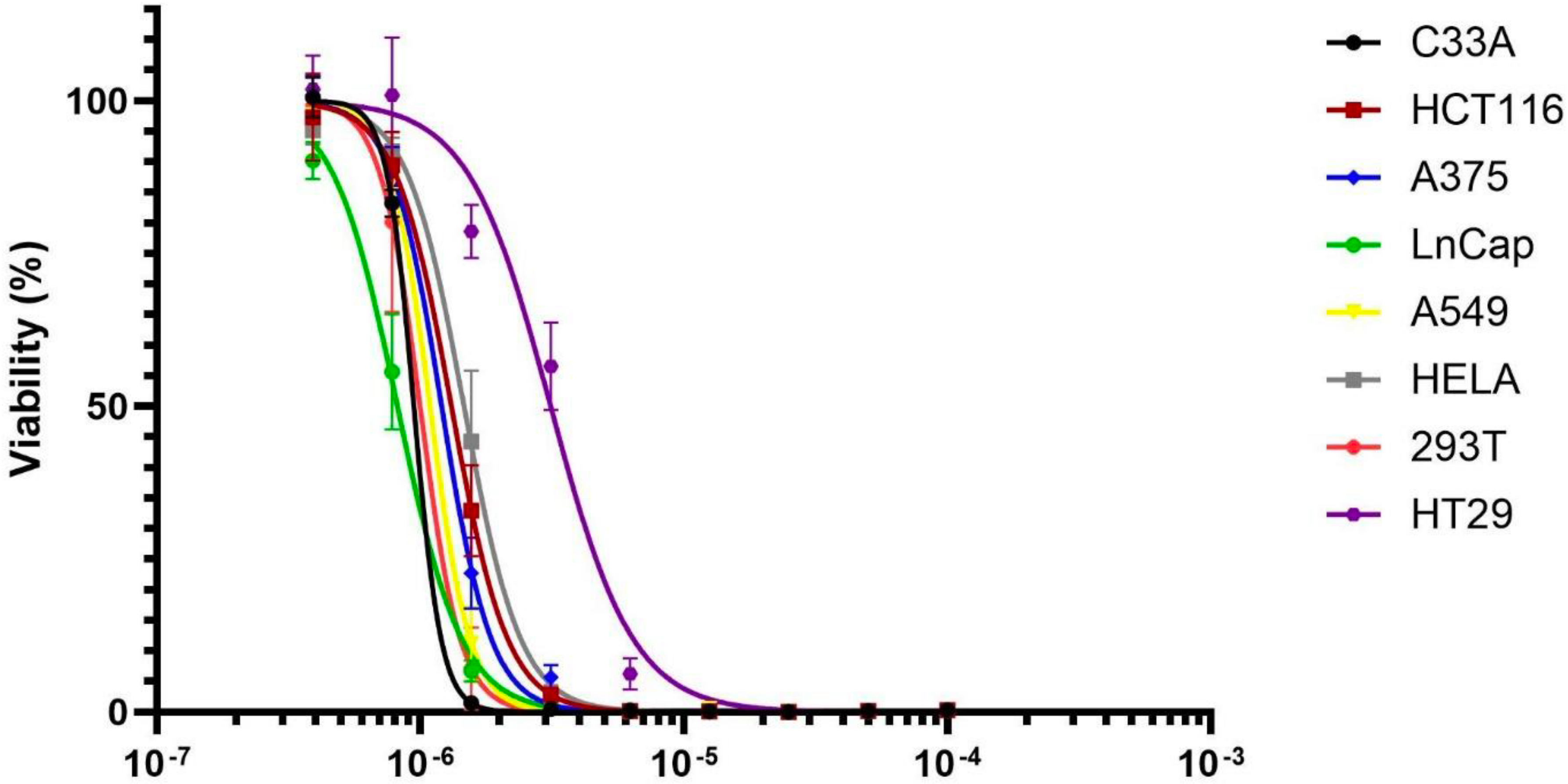

Diacarnusin A (**1**) [M]

Figure S84. Concentration-response cytotoxicity curves of Methyl Diacarnoate A (**10**) on the different cell lines

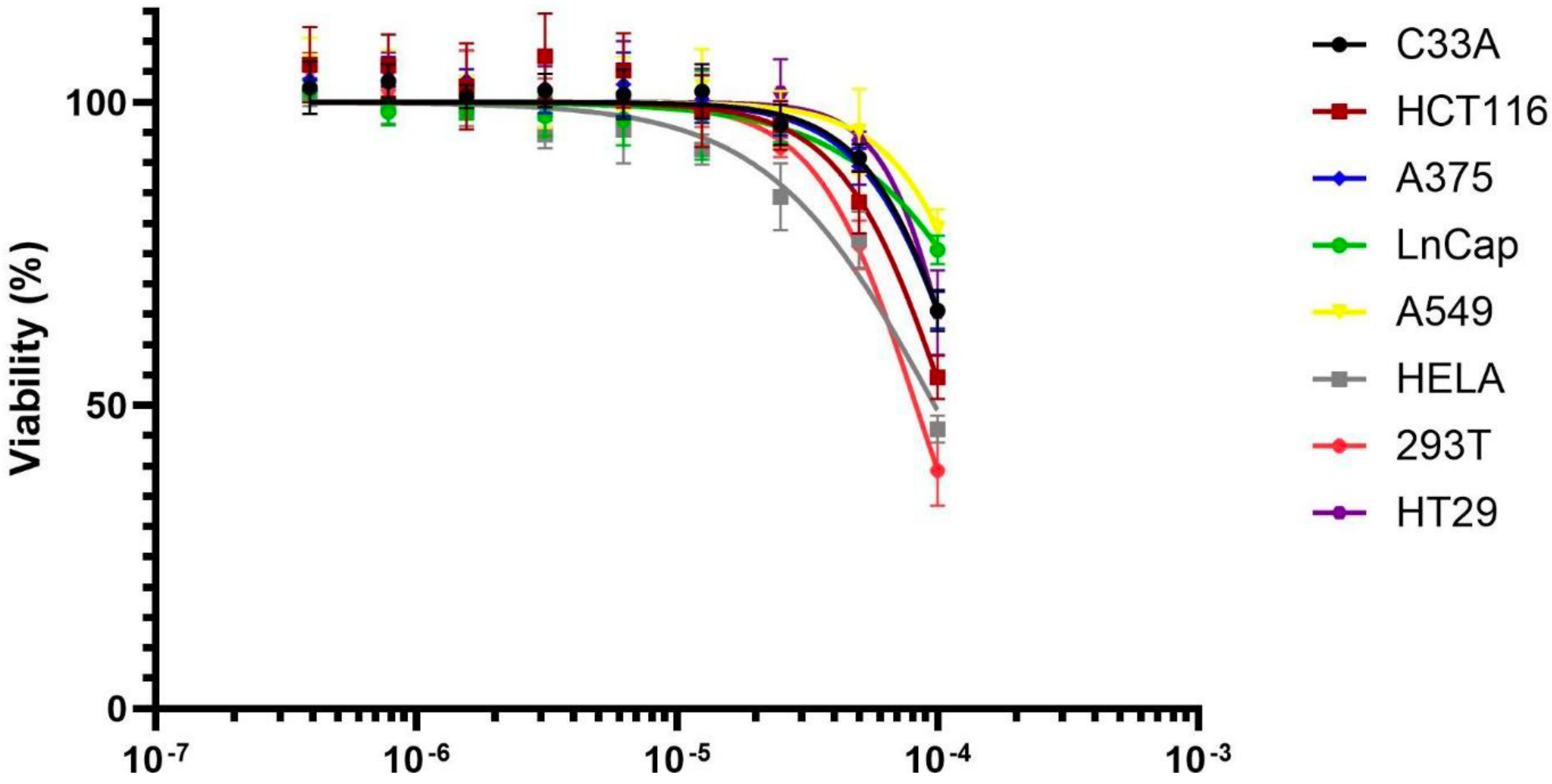

Diacarnusin B (**2**) [M]

Figure S85. Concentration-response cytotoxicity curves of Diacarnusins E&F (**5&6**) on the different cell lines

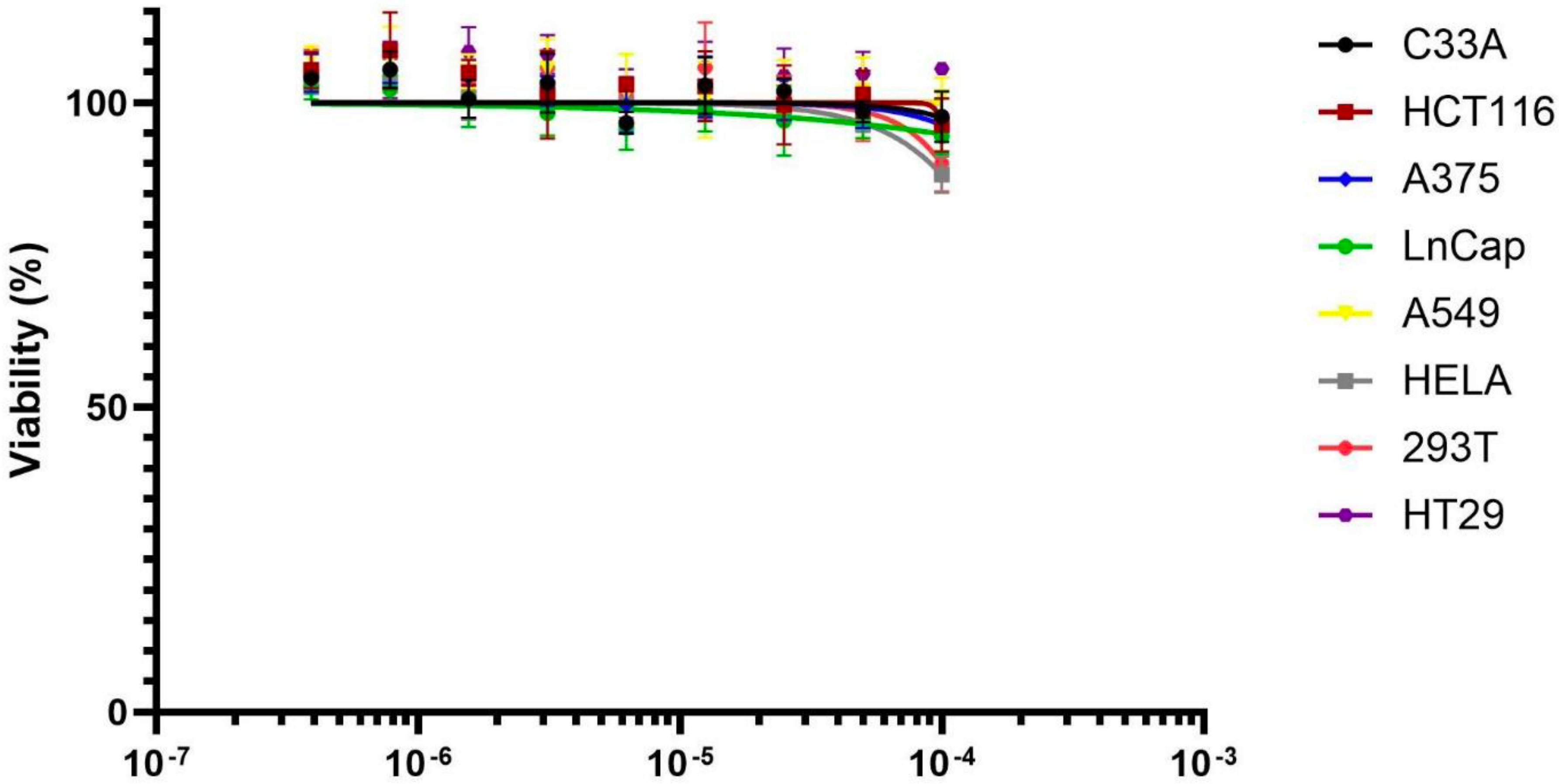

Diacarnusins E & F (**5&6**) [M]

Figure S86. Concentration-response cytotoxicity curves of Sigmosceptrellin B (**7**) on the different cell lines

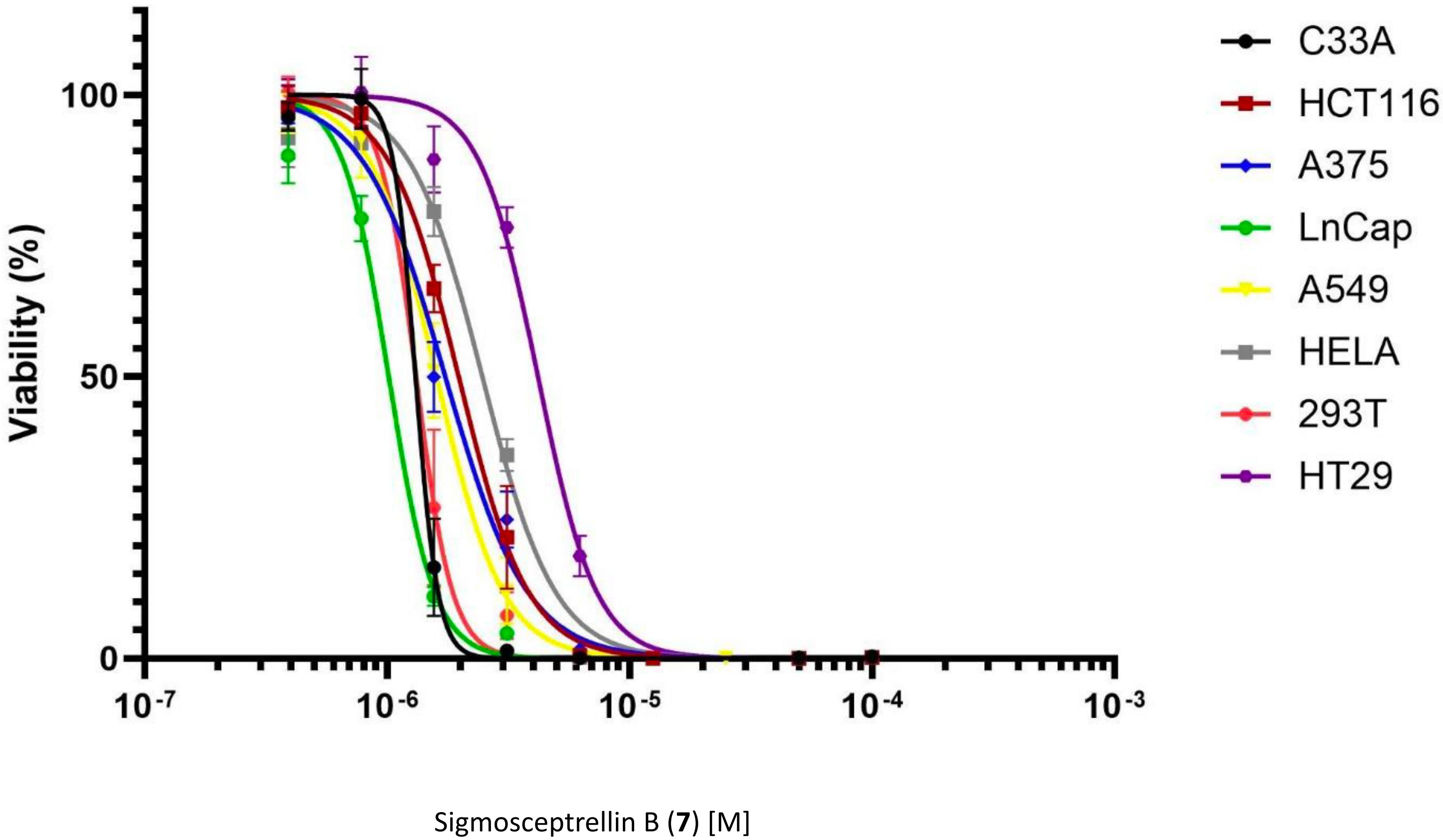

Figure S87. Concentration-response cytotoxicity curves of Sigmosceptrellin B methyl ester (**8**) on the different cell lines

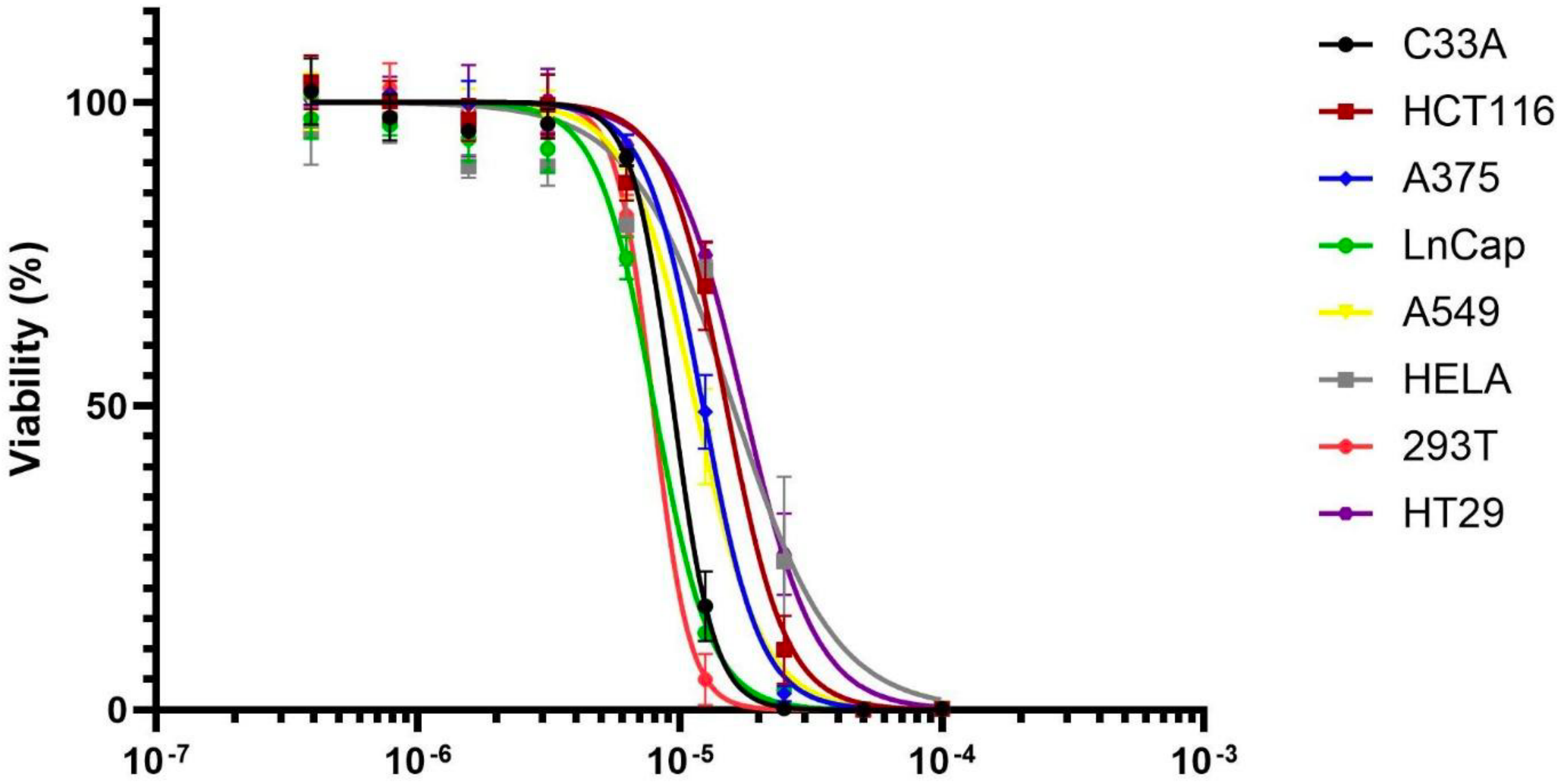

Sigmosceptrellin B methyl ester (**8**) [M]

Figure S88. Concentration-response cytotoxicity curves of Diacarperoxide A (**9**) on the different cell lines

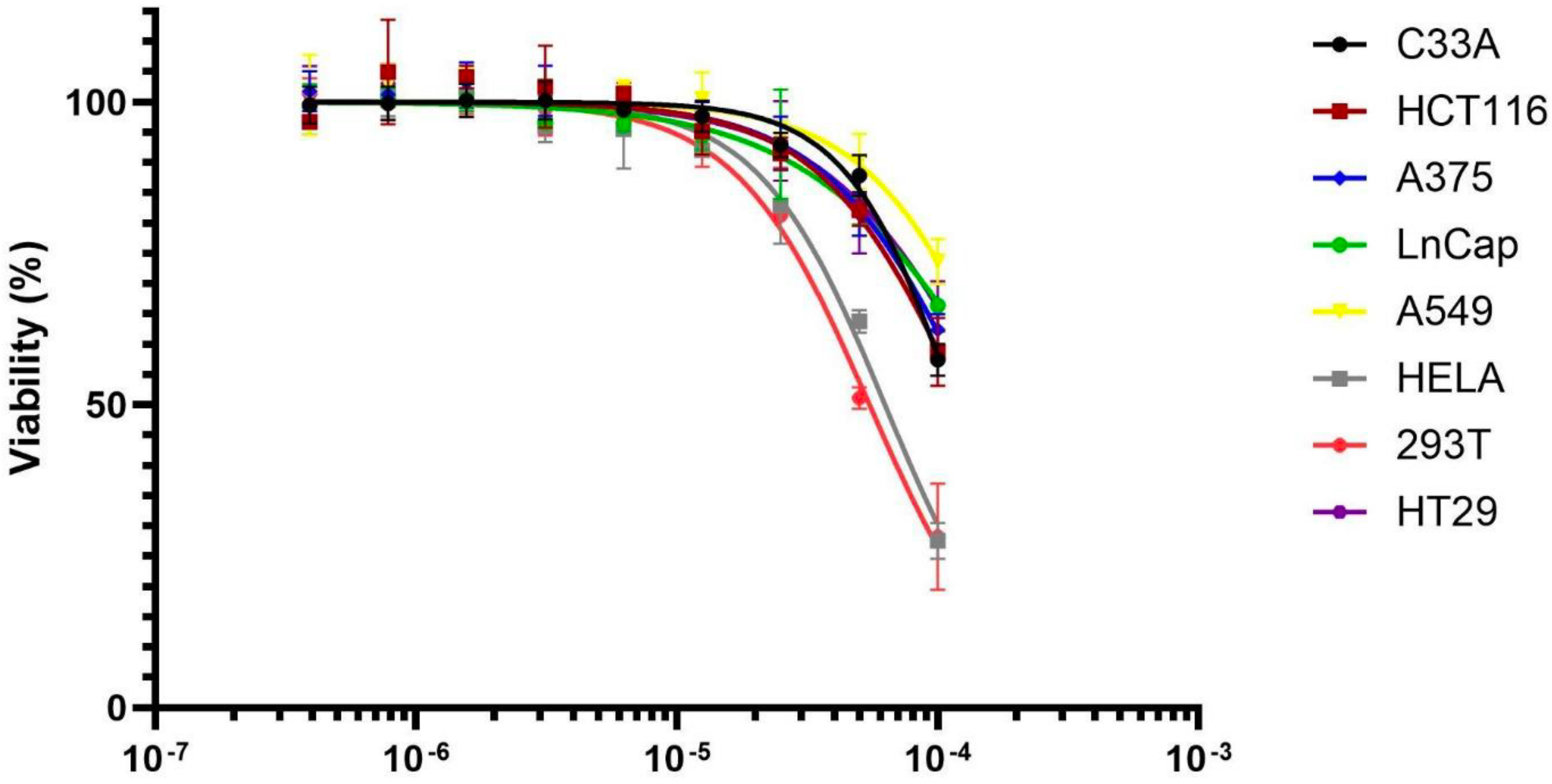

Diacarperoxide A (**9**) [M]

Figure S89. Concentration-response cytotoxicity curves of Methyl Diacarnoate A (**10**) on the different cell lines

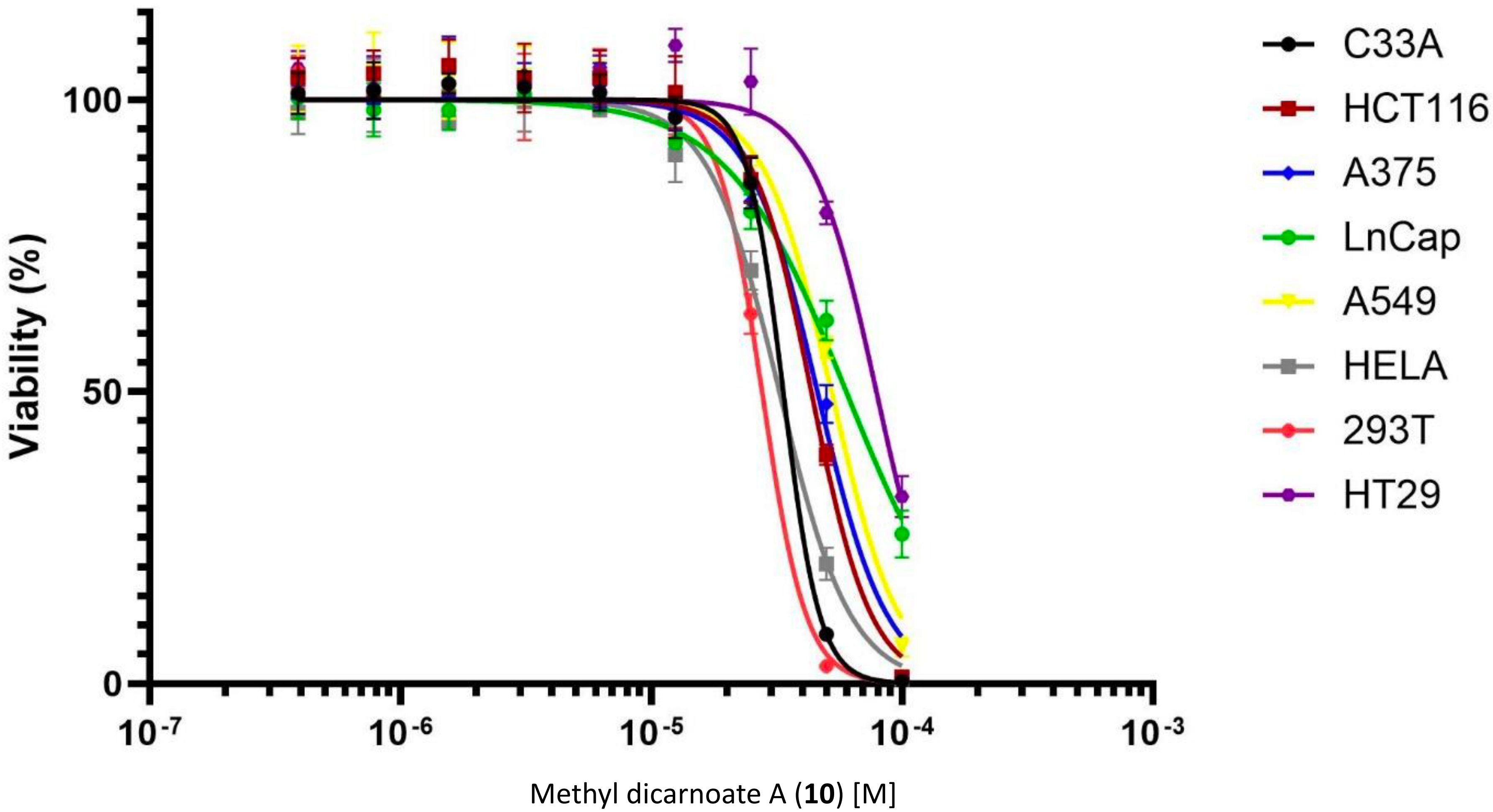

Figure S90. Concentration-response cytotoxicity curves of (-)-ent-Muqubilone (**11**) on the different cell lines

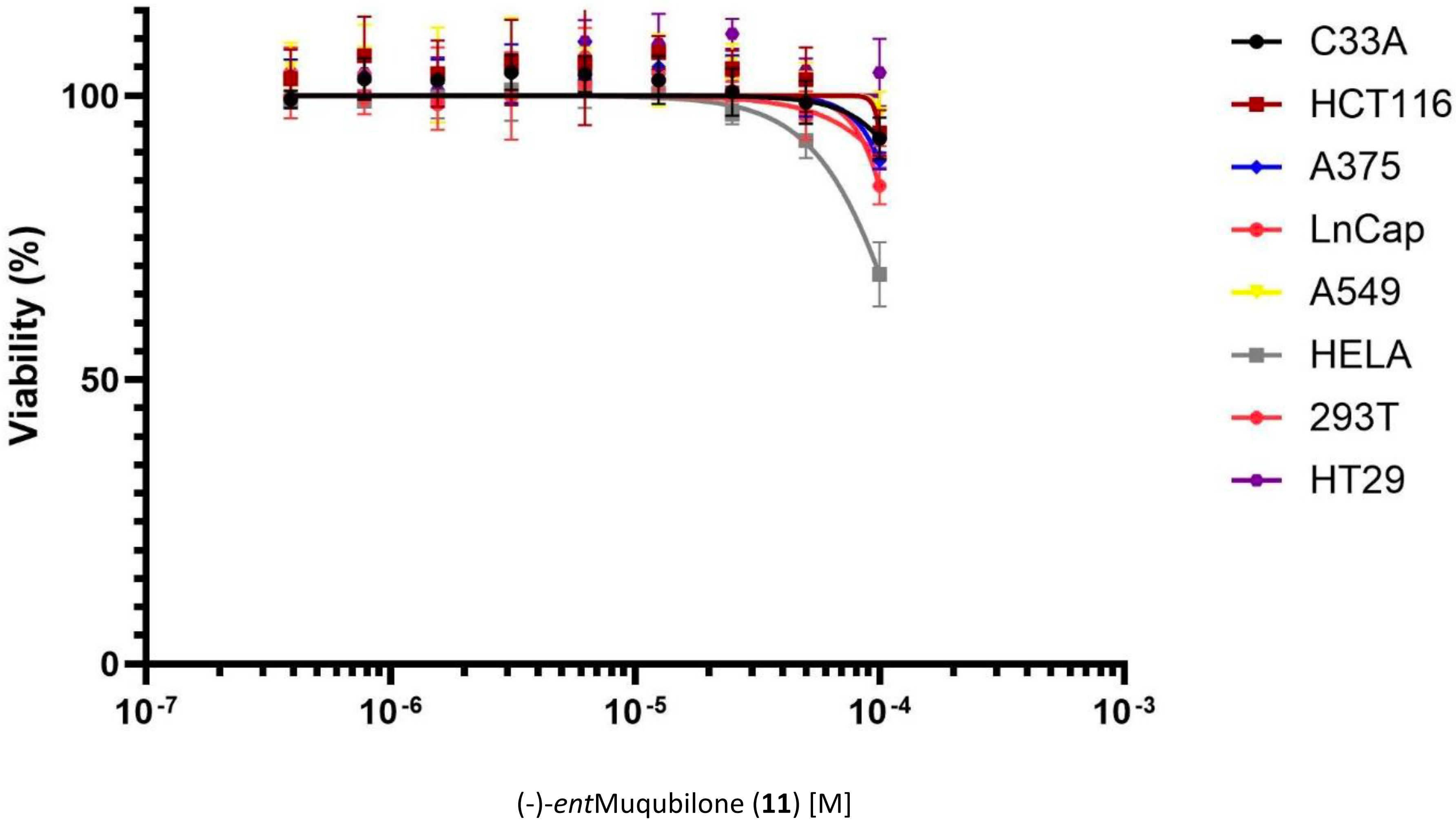

Supplement: Supplementary file 1 [file np6c00510_si_001.pdf]
